# Solvent-Mediated Tunable Regiodivergent C6- and N1-Alkylations of 2,3-Disubstituted Indoles with *p*-Quinone Methides

**DOI:** 10.1021/acs.joc.2c02937

**Published:** 2023-02-13

**Authors:** Douaa Adris, Yunus Taskesenligil, Volkan Akyildiz, Selcuk Essiz, Nurullah Saracoglu

**Affiliations:** † Department of Chemistry, Faculty of Sciences, Atatürk University, Erzurum 25240, Türkiye; ‡ Department of Medical Services and Techniques, Vocational School of Health Services, Hakkari University, Hakkari 30000, Türkiye

## Abstract

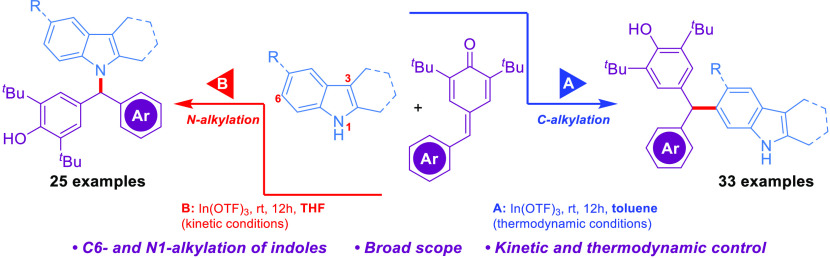

Indium-catalyzed, solvent-enabled regioselective C6- or N1-alkylations of 2,3-disubstituted indoles with *para*-quinone methides are developed under mild conditions. Notably, highly selective and switchable alkylations were selectively achieved by adjusting the reaction conditions. Moreover, scalability and further transformations of the alkylation products are demonstrated, and this operationally simple methodology is amenable to the late-stage C6-functionalization of the indomethacin drug. The reaction pathways were explained with the support of experimental and density functional theory studies.

## Introduction

**Figure 1:**
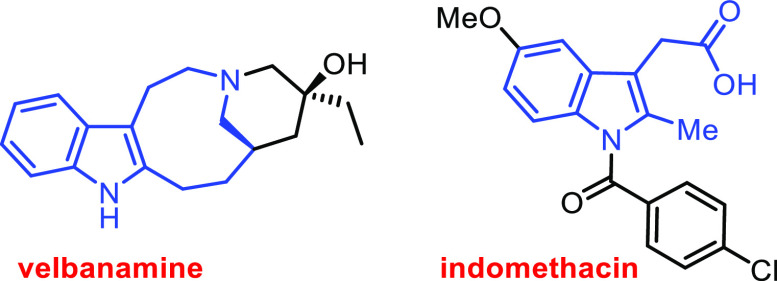
Representative compounds with biologically active 2,3-disubstituted/fused indole motifs.

Indole is one of the most significant heteroaromatic structural units found in numerous bioactive natural products, medicinal compounds, and synthetic compounds and plays an important role in their bioactivity.^1^ Particularly, the substructures of 2,3-disubstituted indole skeletons (as fused or substitutions) are medicinally and synthetically valuable scaffolds and present as a core structural motif in numerous pharmaceutically relevant compounds.^2−5^ Velbanamine and a small subgroup of tetracyclic natural bases belonging to the Iboga family are representative fused indole substructure examples (Figure 1).^6^

The 2,3-disubstituted indole scaffold is also a basic structural motif for the nonsteroidal anti-inflammatory drug (NSAID) indomethacin.^7^ Therefore, there is interest in the development of efficient methods to access 2,3-disubstituted indoles. Most of these methods for functionalized 2,3-disubstituted/fused indoles include the architecture of the parent heterocycle by cyclization via different transition metals and metal-free catalysis conditions using phenylhydrazine, aniline, and nitrobenzene as main substrates.^8^ Other approaches to these functionalized indoles involve derivatization of the indole nucleus via cross-coupling methodology or direct C–H/N–H activation.^9^

**Scheme 1:**
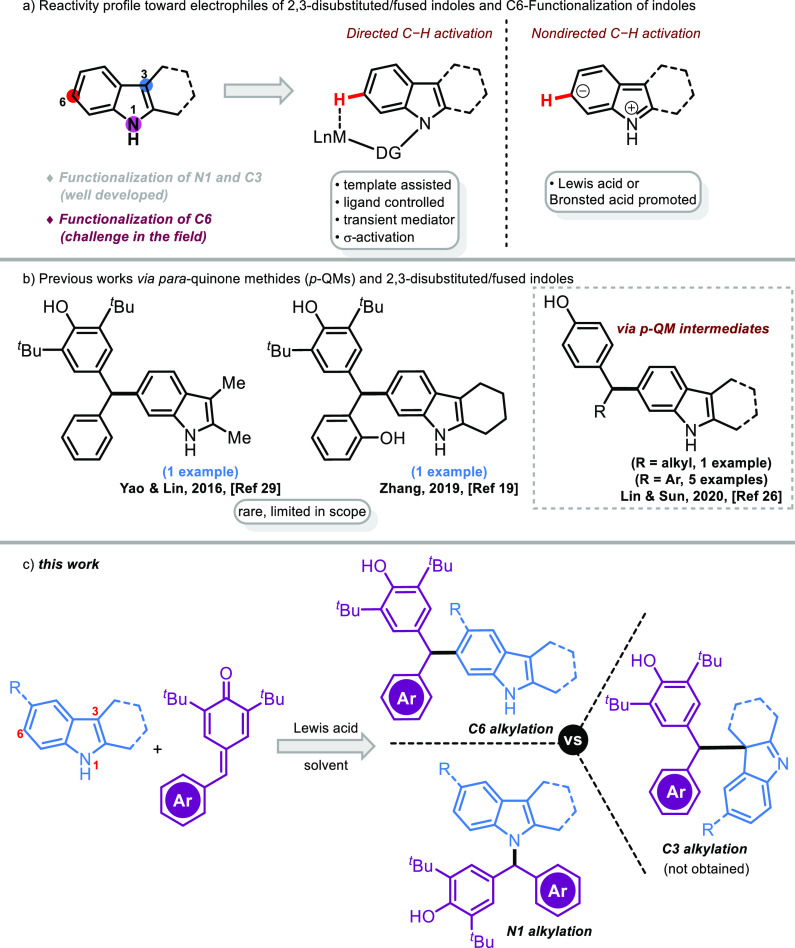
Context of the Work

Meanwhile, N1, C3, and C6 positions of 2,3-disubstituted indoles may exhibit mainly three types of nucleophilic behavior toward electrophiles due to their electronic nature (Scheme 1a). The C3- and N1-positions of 2,3-disubstituted indoles have been well documented as the most reactive sites among the three positions (N1, C3, C6), and the majority of their reactions were focused on the C3- and N1-positions.^10−12^ Furthermore, 2,3-disubstituted/fused indoles can couple with electrophiles to permit Friedel–Crafts-type remote C6-functionalization, which is relatively more difficult (Scheme 1a). In this context, significant progress has been recently achieved via remote C6–H functionalization of indoles. Limited elegant approaches, such as template-assisted C6-olefination,^13^ ligand-controlled C6-borylation,^14^ C6-arylation via a transient mediator,^15^ and C6-alkylation with σ-activation,^16^ are available for the remote C(sp^2^)–H activation (Scheme 1a, middle side). In addition, Lewis and Bronsted acid promoted Friedel–Crafts-type reactions were also reported for the C6–H alkylation (Scheme 1a, right side).^17−23^ Within this scope, Zheng, You, and co-workers, pioneers in the field, first described scandium triflate-catalyzed direct C6 alkylation of 2,3-disubstituted indoles with aziridines via Friedel–Crafts-type functionalization.^17^ Subsequently, Shi and co-workers reported another pioneering study via chemo- and regiospecific C6-alkylation of 2,3-disubstituted indoles using 3-indolylmethanols with organocatalysts.^18^ Also, Zhang and co-workers described chiral phosphoric acid (CPA)-catalyzed remote C6-enantioselective C–H functionalization of 2,3-disubstituted indoles with isatin-derived *N*-Boc ketimine through the dual H-bonds and π–π interaction strategy.^19^ However, the enantioselective C6-functionalization of 2,3-disubstituted indoles with benzofuran-derived azadienes using chiral phosphoric acid led to a range of enantioenriched heterotriarylmethanes.^20^ Recently, electrophiles such as *o*-hydroxybenzyl alcohol,^21^ trifluoromethylated 3-indolylmethanol,^22^ and carbene^23^ have been utilized in the Friedel–Crafts-type C6-alkylations of 2,3-disubstituted indoles.

*p*-Quinone methides (*p*-QMs) have attracted considerable attention as powerful acceptors in 1,6-nucleophilic conjugate addition reactions with a wide range of carbon and heteroatom nucleophiles.^24^ To date, indoles have been also tested as donor molecules on this 1,6-conjugate addition platform to access triarylmethanes.^25,26^ Despite those elegant advancements using this concept, the synthesis of triarylmethanes on the benzenoid ring of the indole motif is still challenging and there are a limited number of examples.^27,28^ In 2016, Yao, Lin, and co-workers disclosed a single example (in a yield of 66%, its structure was drawn incorrectly in the article) of C6-alkylation of 2,3-dimethylindole with *p*-QM under metal-free conditions (Scheme 1b).^29^ Later, Zhang and co-workers reported another single example (in a yield of 83%, 62% ee) of CPA-catalyzed remote C6-enantioselective C–H alkylation of a 2,3-fused indole with a *p*-QM.^19^ In 2020, Lin, Sun, and co-workers reported the synthesis of an unsymmetrical diarylmethane (1 example) and triarylmethanes (5 examples) through *p*-TsOH-catalyzed 1,6-conjugate addition of 2,3-dimethyl/fused indoles to in situ generated *p*-QM intermediates (Scheme 1b).^26^ Consequently, the preceding examples of the C6-functionalization of indoles with *p*-QMs suffered from limited substrate scope (in terms of both indoles and *p*-QMs). However, the development of efficient protocols for the chemoselective functionalization through both C6–H and N1–H bonds of unprotected indoles is still a difficult problem and challenge. Herein, we describe In(OTf)_3_-catalyzed divergent alkylations (from C6 and N1 positions) of unprotected 2,3-disubstituted/fused indoles with *p*-QMs (Scheme 1c). The high chemoselectivity was made feasible by a delicate choice of solvents. To our knowledge, this protocol represents the first comprehensive examples of tunable chemoselective alkylations of unprotected 2,3-disubstituted/fused indoles. The present strategy is highly tunable depending on the catalyst, temperature, and solvent employed.

## Results and Discussion

In continuation of our research interest in C–H and N–H alkylation reactions,^27^ 2,3,4,9-tetrahydro-1*H*-carbazole (**1a**) and *p*-QM **2d** were selected as the model substrates to optimize the reaction conditions (Table 1). The initial experiments were conducted in 1,2-dichloroethane (DCE) at room temperature for 12 h by using 10 different metal triflates as Lewis acid promoters (Table 1, entries 1–10). Zn(OTf)_2_ (10 mol %) and Gd(OTf)_3_ (10 mol %) were found to promote the reaction quite well, giving the N1-alkylation product **4ad** in 82 and 77% yields, respectively (entries 1 and 2), whereas Bi(OTf)_3_ (10 mol %) furnished C6-alkylation product **3ad** in an 84% yield (entry 3). Subsequently, when other Lewis acids such as Cu(OTf)_2_ and LiOTf were tested for the reaction, no alkylation products were detected, and only starting materials were recovered (entries 4 and 5). The subsequent screening of other triflates, such as scandium(III), tin(II), silver(I), ytterbium(III), and indium(III) triflates, revealed that the desired C6-alkylation product **3ad** was generated with excellent regioselectivity and yields (entries 6–10). We also checked the reaction with Bronsted acids in DCE (Table 1, entries 11 and 12). When TfOH was sufficient to promote the reaction, **3ad** was obtained in 82% yield (entry 11). In contrast, NaHSO_4_ as the solid heterogeneous acid catalyst afforded N1-alkylation product **4ad** (entry 12). Furthermore, when the reaction was performed with HFIP as a catalyst, any alkylation products were not formed (Table 1, entry 13). Notably, entries 1, 6, and 10 indicated that the catalysts such as Zn(OTf)_2_, Sc(OTf)_3_, and In(OTf)_3_ played an important role in regioselective switching. Next, to test the effect of the solvent with these catalysts, further additional optimization was conducted (Table 1, entries 14–24). Using THF and DMSO instead of DCE as a solvent for the Zn(OTf)_2_-catalyzed reaction, no product **3ad** or **4ad** was detected (entries 14 and 15). To our surprise, different solvents showed a noticeable effect on the Sc(OTf)_3_-catalyzed reaction (Table 1, entries 16 and 17). DCM showed the same effect as DCE (entry 16), while THF switched the regioselectivity for the product from **3ad** to **4ad** (in 85% yield) (entry 17). Based on these results, we further extensively screened various solvents for the indium(III)-catalyzed reaction (Table 1, entries 18–24). Interestingly, the reaction in ethyl acetate afforded **3ad** as a major product in 56% yield and with almost 2:1 regioselectivity (entry 18), while the reaction in dioxane gave **4ad** as a major product in 84% yield and with almost 10:1 regioselectivity (entry 22).

**Table 1:**
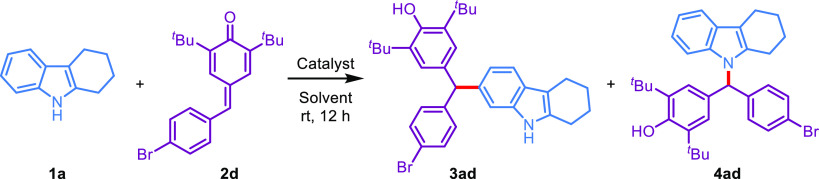
Optimization of Reaction Conditions^a^

entry	catalyst	solvent	**3ad** (yield, %)	**4ad** (yield, %)^b^
1	Zn(OTf)_2_	DCE		82
2	Gd(OTf)_3_	DCE		77
3	Bi(OTf)_3_	DCE	84	
4	Cu(OTf)_2_	DCE		
5	LiOTf	DCE		
6	Sc(OTf)_3_	DCE	84	
7	Sn(OTf)_2_	DCE	80	
8	AgOTf	DCE	72	
9	Yb(OTf)_3_	DCE	75	
10	In(OTf)_3_	DCE	87	
11	TfOH	DCE	82	
12	NaHSO_4_	DCE		81
13	HFIP	DCE		
14	Zn(OTf)_2_	THF		
15	Zn(OTf)_2_	DMSO		
16	Sc(OTf)_3_	DCM	80	
17	Sc(OTf)_3_	THF	trace	85
18	In(OTf)_3_	EtOAc	56	25
19	In(OTf)_3_	DCM	88	
20	In(OTf)_3_	MeCN	74	
21	In(OTf)_3_	CH_3_NO_2_	78	
22	In(OTf)_3_	dioxane	9	84
**23**^c^	**In(OTf)**_**3**_	**THF**	**trace**	**90**
**24**	**In(OTf)**_**3**_	**toluene**	**92**	**trace**

a Reaction conditions. For **3ad**; **1a** (0.20 mmol), catalyst (10 mol %), **2d** (0.22 mmol), solvent (2 mL).

b Isolated yield.

c For **4ad**; **1a** (0.30 mmol), catalyst (10 mol %), **2d** (0.20 mmol), solvent (2 mL).

When dichloromethane, acetonitrile, and nitromethane were applied to the reaction instead of DCE, similar results were found (entries 19–21). Significantly, the use of THF in the In(III)-catalyzed reaction system gave the best result for the *N*1-alkylation product **4ad** with a 90% yield (entry 23). We found the yield of C6-alkylation product **3ad** was improved to 92% with toluene as solvent (entry 24). Based on the above results, 10 mol % In(OTf)_3_ is the preferred catalyst for these regioselective reactions. Furthermore, the use of THF for N1-alkylation and toluene for C6-alkylation as solvent were determined to be the other optimal reaction parameters at room temperature for 12 h (entries 23 and 24).

**Scheme 2:**
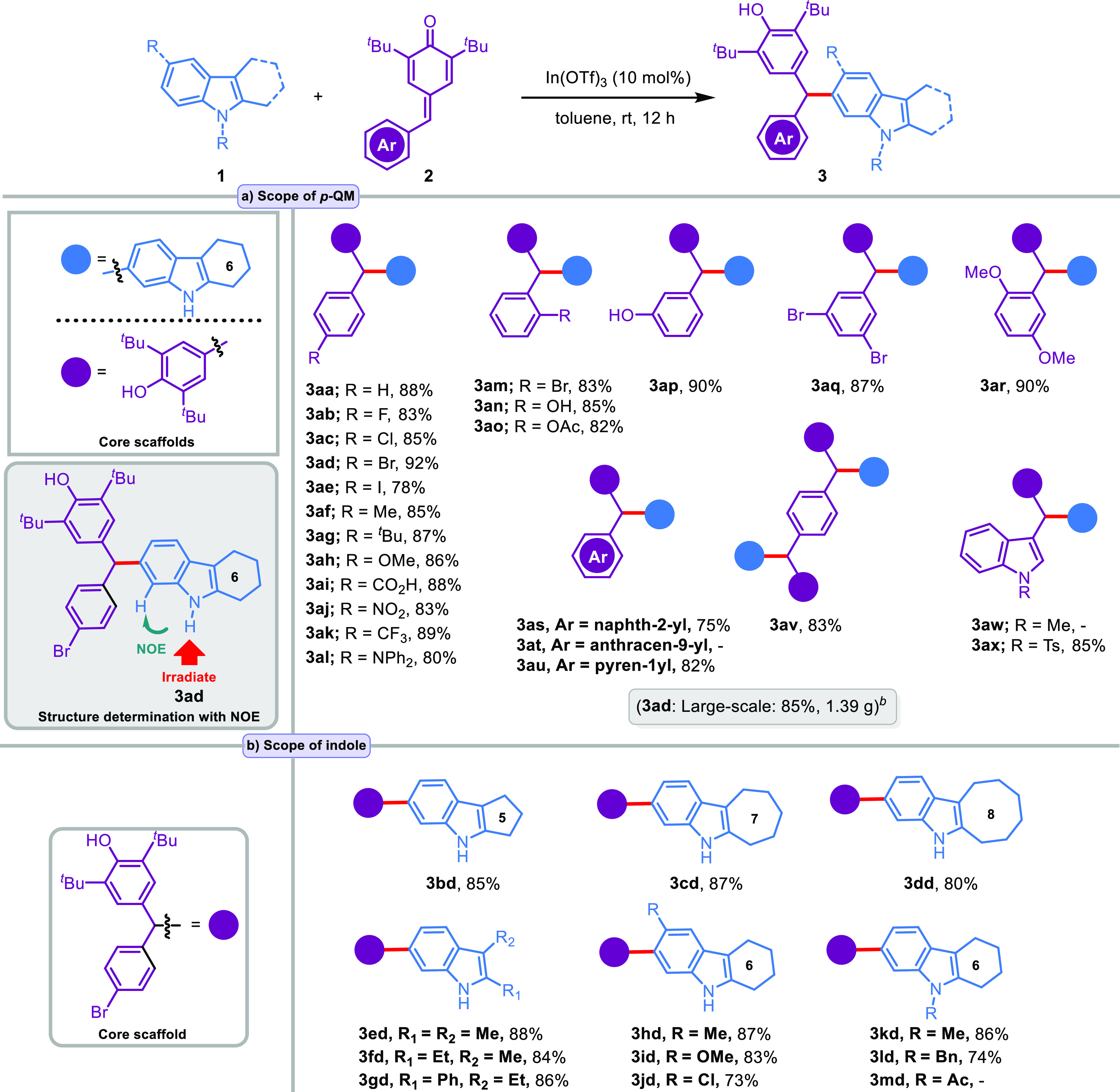
Substrate Scope for C(6)–H Alkylation Reaction conditions: **1** (0.20 mmol), In(OTf)_3_ (10 mol %), **2** (0.22 mmol), toluene (2 mL). Large-scale synthesis of **3ad** (1.39 g, 2.55 mmol).

Under the established optimized reaction conditions, we attempted to survey the substrate scope of various *p*-QM substrates **2a**–**x** for C6-alkylation of **1a** (Schemes 2a). By employing **1a** as the coupling partner, we first investigated the generality of *p*-QMs **2a**–**x**. A series of C6-alkylated products **3aa–ax** were obtained in good to excellent yields (75–92% yield) under the optimized conditions (entry 24, Table 1). To our delight, this indium(III)-catalyzed 1,6-hydroarylation reaction in toluene demonstrated a broad scope for the *p*-QM reaction partner. In the case of *p*-QM **2a** without any substituent on the phenyl ring, the reaction proceeded well and delivered the corresponding product **3aa** in 88% yield. *p*-QMs bearing a halogen group at the *para*-position of aryl ring afforded C6-alkylated indoles **3ab–ae** in good to excellent yields (78–92%). A series of *p*-QMs including both electron-donating substituents (**2f**–**h**) such as methyl, *tert*-butyl, and methoxy and electron-withdrawing substituents (**2i**–**k**) such as carboxylic acid, nitro, and trifluoromethyl at the *para*-position of the phenyl ring were well tolerated to yield the desired products (**3af–ak**) in the range of 83–89% yields under the optimal conditions. The *p*-QM **2l** bearing a diphenylamine group at the *para*-position of the aryl ring was also a suitable reaction partner for the 1,6-addition **3al** in 80% yield. Even the reactions of substrates **2m**–**r** with sterically hindered *ortho*-monosubstituted (Br, OAc, and OH), *meta*-monosubstituted (OH), *meta*/*meta*-disubstituted (Br), and *ortho-*/*meta*-disubstituted (OMe) aromatic rings proceeded efficiently and led to the corresponding products **3am–ar** in excellent yields between 82 and 90%. Further, *p*-QMs **2s** and **2u** bearing naphth-2-yl and pyren-1-yl groups were also compatible and delivered the addition products **3as** and **3au** in 75% and 82% yields, respectively, whereas *p*-QMs **2t** bearing an anthracen-9-yl group failed to yield **3at** due to the possible steric effect of the anthracene ring. On the other hand, the corresponding *p*-QM **2v** of terephthalaldehyde gave tandem double 1,6-conjugate addition product **3av** in 83% yield. Finally, *p*-QMs **2w**–**x** bearing the heterocyclic ring were used as a substrate instead of the carbocyclic aryl ring under standard conditions. First, *N*-tosylindole-substituted *p*-QM **2x** gave the desired product **3ax** in an excellent yield of 85%. Second, *N*-methylindole-substituted *p*-QM **2w** was employed as the substrate; however, no reaction occurred under the standard conditions. This result indicated that the reactivity of *N*-methylindole-substituted *p*-QM **2w** is lower than that of *N*-tosylindole-substituted *p*-QM **2x** in the conjugate addition with **1a**. We hypothesize that the electron-rich *N*-methylindole ring provides electrons by resonance, reducing the reactivity of the corresponding *p*-QM. With the optimized conditions in hand, we next evaluated the substrate scope of 2,3-disubstituted indole derivatives. As depicted in Scheme 2b, a wide range of 2,3-disubstituted indoles were suitable reaction partners for *p*-QM **2d** and provided the desired products in good to excellent yields. Pleasantly, 5-, 7-, and 8-membered fused-ring substrate triad **1b**–**d** provided C6-alkylation products **3bd–dd** with high yields and regioselectivity. Also, indoles **1e**–**g** bearing dimethyl, ethyl/methyl, and phenyl/ethyl on the 2,3-positions tolerated C6-alkylation with high efficiencies (**3ed–gd**, 84–88%). With 6-membered fused-ring indoles **1h**–**j** bearing electron-donating groups (−Me and −OMe) and an electron-withdrawing group (−Cl) on the 5-position, the expected products **3hd–jd** were obtained in high yields (73–87%). Notably, the *N*-protected 6-membered fused-ring indoles **1k**–**l** with *N*-methyl and *N*-benzyl groups also exhibited pleasing results, and the corresponding products **3kd–ld** were obtained in high yields (86% and 74%). The *N*-acetyl-protected indole **1m** failed to react with *p*-QM **2a** under the standard reaction conditions. This result can be attributed to the π-electron-withdrawing effect of the acetyl substituent. The structures of the C(6)–H alkylation products were elucidated utilizing NMR spectroscopy and high-resolution mass spectrometry (HRMS). As a representative example, the alkylation at the C6-position of **3ad** was also determined by a nuclear Overhauser effect (NOE) study. NOE correlations between the C(7)–H and N(1)-H, hydrogens (see green arrow) agree with the alkylation depicted (Scheme 2a). To demonstrate the practicality of the current protocol, a gram-scale synthesis of **3ad** was also investigated under the optimized standard reaction conditions, and the yield of the desired product was only slightly decreased with no effect on regioselectivity (Scheme 2a). This result reveals that the current transformation can be used to synthesize C6-alkylated indoles with practical usefulness.

**Scheme 3:**
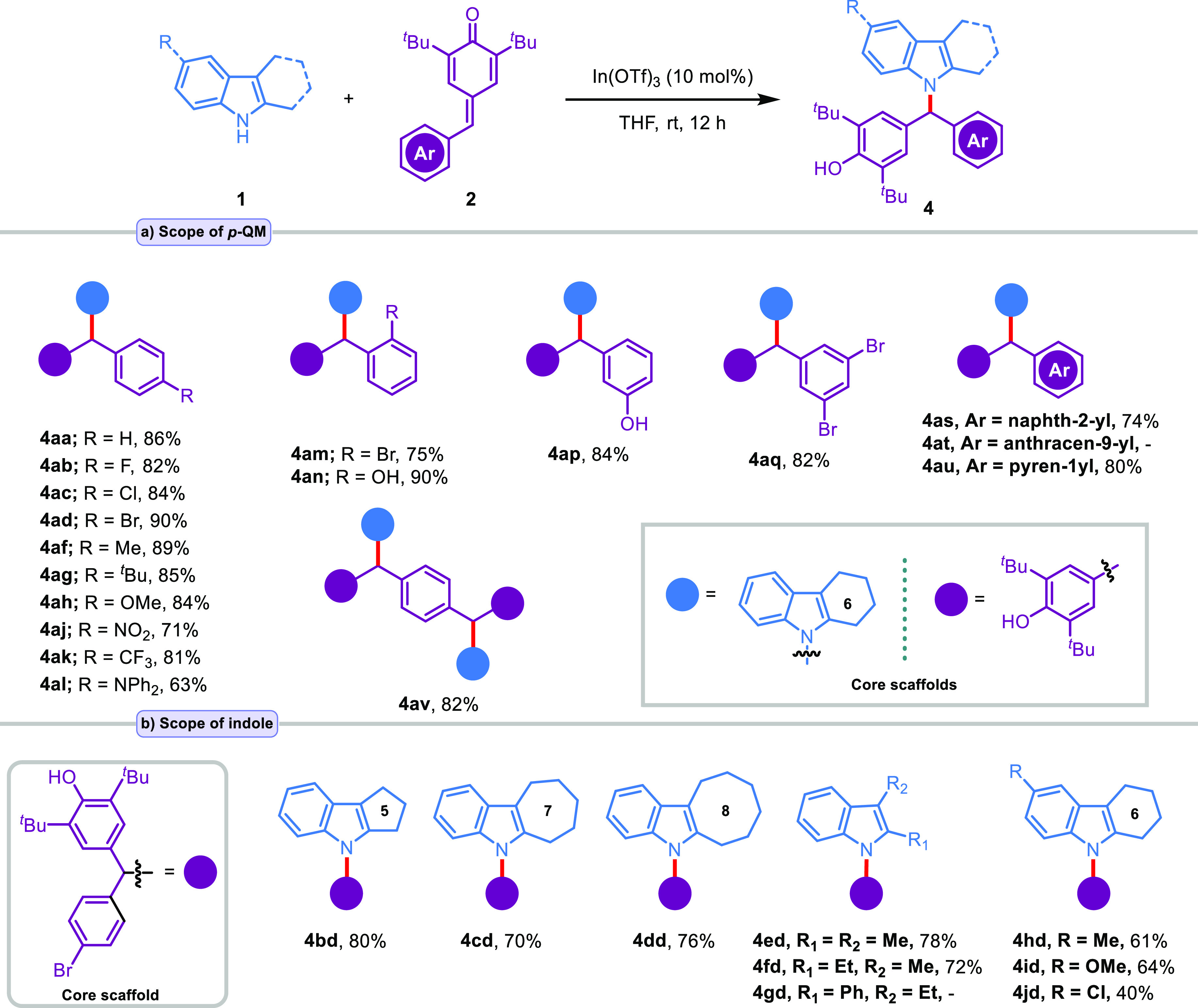
Substrate Scope for N(1)–H Alkylation Reaction conditions: **1** (0.30 mmol), In(OTf)_3_ (10 mol %), **2** (0.20 mmol), THF (2 mL).

As depicted in Scheme 3, the N(1)–H alkylation reactions of various 2,3-disubstituted/fused indoles **1b**–**j** mediated by In(OTf)_3_ in THF were also explored under the optimized conditions (Table 1, entry 23). In this context, the *N*-alkylation product **4aa** was generated and isolated in 85% yield (Scheme 3a). On the other hand, the *p*-QMs (**2b**,**c**, **2f**–**h**, and **2j**–**l**) containing different electron-donating and -withdrawing groups such as fluoro, chloro, methyl, *tert*-butyl, methoxy, nitro, trifluoromethyl, and diphenylamine at the *para*-position of the phenyl ring were suitable substrates to react with **1a** in this transformation, affording the corresponding alkylation products **4ab–ac**, **4af–ah**, and **4aj–al** in 63–90% yields.

**Scheme 4:**
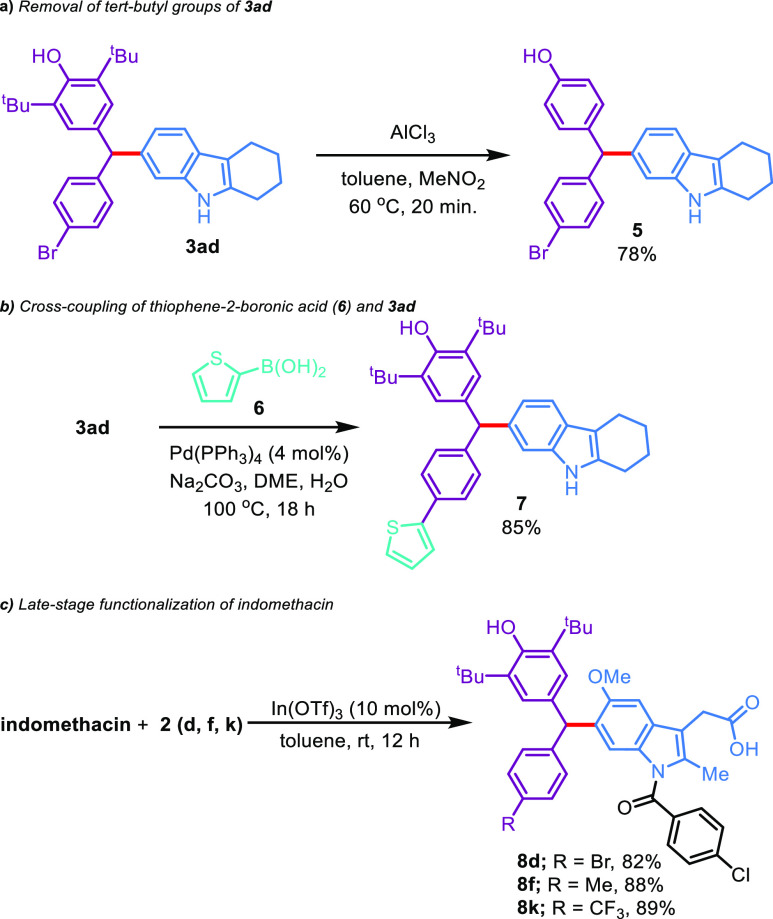
Follow-Up Chemistry

Additionally, when the electron-donating and -withdrawing *ortho*-substituted *p*-QMs **2m**–**n** were used as the substrates for the reaction, the alkylation process took place at the nitrogen atom in indole derivative **1a** to afford the corresponding products **4am–an** in 75% and 90% yields, respectively (Scheme 3a). Furthermore, the introduction of mono- and disubstitution at the *meta*-position did not significantly affect the yield of products **4ap** (84% yield) and **4aq** (82% yield) (Scheme 3a). *p*-QMs with other aromatic rings, such as naphthalene and pyrene, were also tested, giving satisfactory results. The corresponding products **4as** and **4au** were obtained in good yields (74% and 80%) (Scheme 3a). Unfortunately, in the case of anthracenyl-substituted *p*-QM **2t**, no product (**4at**) formation was observed (Scheme 3a). It is assumed that steric hindrance due to the size of the anthracene ring blocks the approach of the nucleophile. When the terephthalaldehyde-*p*-QM **2v** was used, both *p*-QM groups reacted and the desired product **4av** was obtained in 82% yield (Scheme 3a). Both the 5-, 7-, and 8-membered fused-ring-indoles **1b**–**d** and indoles **1e**–**f** including substituents such as dimethyl and ethyl/methyl on 2,3-positions can react efficiently with 4-(4-bromobenzylidene)-2,6-di-*tert*-butylcyclohexa-2,5-dien-1-one (**2d**) to give the N(1)–H alkylation products **4bd–fd** in 70–80% yields (Scheme 3b). Nevertheless, a complex mixture was obtained when **1g** was reacted with **2d** under standard conditions, and no desired product **4gd** was isolated (Scheme 3b). Notably, substrates containing methyl, methoxy, and chloride groups at the C5-position of 6-membered fused-ring-indole **2a** also provided the target products (**4hd–4jd**) in moderate yields (40–64%) (Scheme 3b). To examine the synthetic potential of the protocol, postfunctionalizations of the C6-alkylation products were also performed (Scheme 4).

Initially, the *retro*-Friedel–Crafts alkylation of **3ad** was attempted with AlCl_3_ as a *tert*-butyl acceptor in toluene/nitromethane to afford the corresponding *para*-substituted phenol **5** in 78% yield (Scheme 4a).^27^ Subsequently, bromo-substituted C6-alkylated product **3ad** was explored for cross-coupling reaction with thiophene-2-boronic acid (**6**) in the presence of Pd(PPh_3_)_4_, Na_2_CO_3_, and 1,2-dimethoxyethane:water (2:1), which afforded the corresponding product **7** with 85% yield (Scheme 4b).^30^ Finally, to demonstrate the applicability of our strategy to the late-stage functionalization of a medicinal relevant molecule, we subjected a pharmaceutically relevant drug to our C6-alkylation protocol: an NSAID indomethacin could be decorated with diarylmethane moieties (**8d**, **8f**, and **8k**) with 82–89% yields (Scheme 4c).

**Scheme 5:**
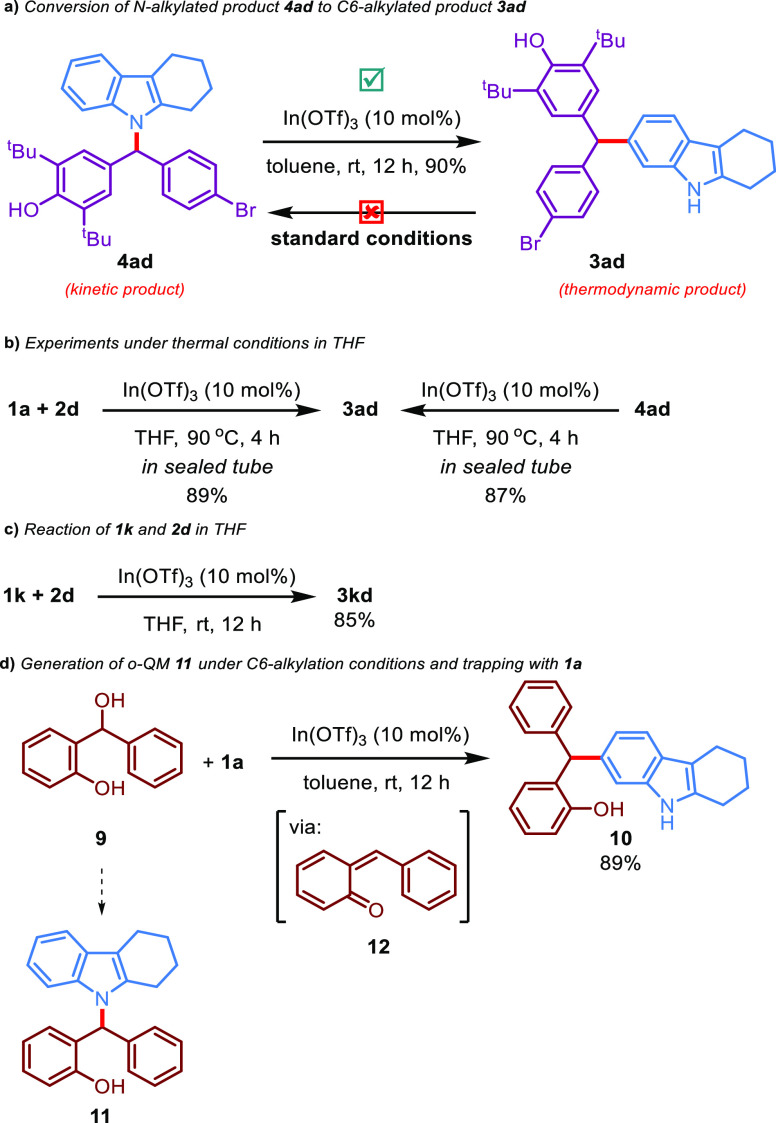
Control Experiments

To elucidate the reaction mechanism of the regioselective alkylations of 2,3-disubstituted/fused indoles with *p*-QMs, a series of control experiments were then performed (Scheme 5). For this, we tested the interconversion between these two different alkylation products **3ad** and **4ad**. When the pure **4ad** was subjected to the conditions for C6-alkylation, **3ad** was isolated in 90% yield as a sole product (Scheme 5a). On the contrary, when the pure **3ad** was subjected to the conditions for *N*-alkylation, the reaction did not proceed (Scheme 5a). Moreover, no conversion from **3ad** to **4ad** was observed, even when the *N*-alkylation reaction time was prolonged to 24 h. Under the thermal conditions in THF (Scheme 5b), the progress of the reaction was further investigated. The reaction between **1a** and **2d** with the 10 mol % In(OTf)_3_ catalyst system in THF for 4 h in a sealed tube at 90 °C surprisingly provided **3ad** in 89% yield instead of **4ad** (Scheme 5b). In addition, when the stirring of **4ad** occurred under the same catalyst system in THF for 4 h in a sealed tube at 90 °C, the conversion of **4ad** to **3ad** was observed in 87% yield (Scheme 5b). When the reaction between **2d** with *N*-methyl-protected indole **1k** for 12 h was conducted only in THF as a solvent, **3kd** was isolated in 85% yield (Scheme 5c). These control experiments disclose that the *N*-alkylated product is probably the kinetically controlled product and the C-alkylated product is the thermodynamically controlled product. Furthermore, we also investigated whether our catalyst system would work on *ortho*-quinone methides (*o*-QMs), a reactive intermediate, to access the corresponding C- and *N*-alkylated products **10** and **11** (Scheme 5d). *o*-Hydroxybenzyl alcohol **9** was selected as a representative o-QM precursor to test In(OTf)_3_-catalyzed target reactions. Gratifyingly, the use of the reaction conditions for the C-alkylation provided product **10** with 89% yield. However, an unstable or not isolable product was observed when the N-alkylation conditions were employed. These reactions indicate that the C-alkylation conditions are more suitable for the generation and trapping of the *o*-quinone methide intermediate **12**.

**Scheme 6:**
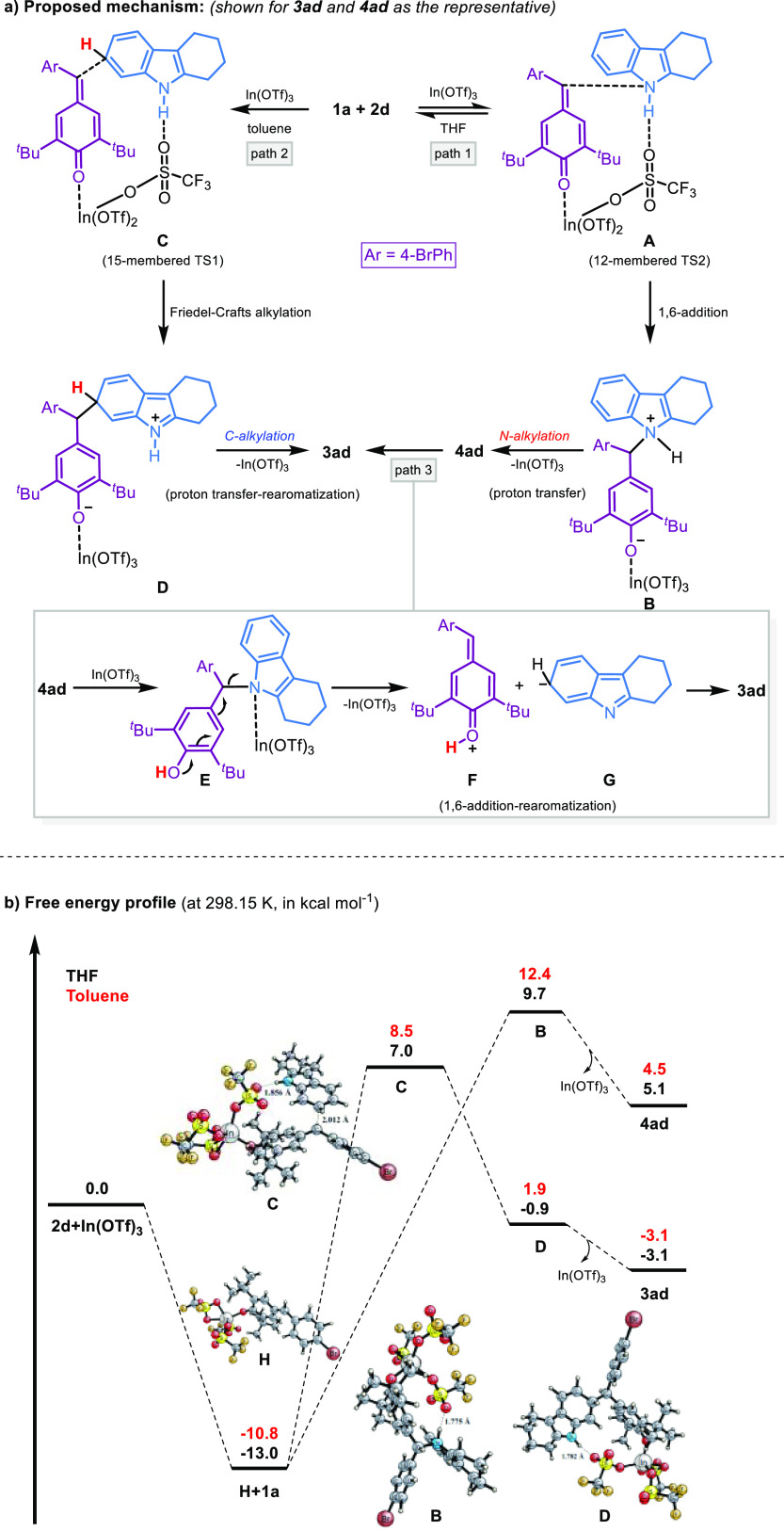
Proposed Mechanism and Gibbs Free Energy Profile

From the above experimental results, it can be seen that the solvent, Lewis acid, and temperature strongly affect the course of the reaction. Based on the results of the optimization and control experiments, together with the related reports,^26,10a^ a plausible reaction mechanism for the N- and C-alkylation was proposed, as shown in Scheme 6a. Three pathways are possible via hydrogen bonding- and metal coordination-assisted catalyst (paths 1–3). With **1a** and **2d** as representative examples, this might involve initial activation of the substrates by In(OTf)_3_ to form 12-membered transition state **A** (TS1). We assume that less membered transition state **A** facilitates the N-nucleophilic attack of **1a** to generate the zwitterionic intermediate **B** and form the C–N bond. Finally, intramolecular proton transfer from N–H and the regeneration of the In(OTf)_3_ catalyst affords the desired product **4ad** (at room temperature in THF, path 1). In contrast, pathway 2 for the C–H functionalization involves a 15-membered transition state **C** (TS2) between catalyst, donor, and acceptor compounds leading to an increase in the nucleophilicity at the C6-position of the indole ring. Next, the Friedel–Crafts-type nucleophilic addition of **1a** to **2d** generates intermediate **D**. Eventually, proton transfer, catalyst regeneration, and isomerization occur to afford the desired product **3ad** (at room temperature in toluene or in heating conditions, path 2). However, the steric crowding **4ad** would be selectively activated by In(OTf)_3_ to yield the intermediate **E**, followed by C–N bond cleavage to form the intermediates **F** and **G**, which produces **3ad** (under heating conditions or at room temperature in toluene, path c).

To gain detailed insights into the above reaction mechanism, we performed a series of DFT computations using the Gaussian 16 program^31^ to explain the experimental results, particularly the interconversion between kinetic and thermodynamic products. The DFT method, hybrid functional B3LYP, was performed using model substrates bearing the bromine group (**1a**, **2d**, **3ad**, **4ad**, intermediates, and transition states).^32−35^ The energy diagram is depicted in Scheme 6b. We further computed their vibrational frequencies to characterize each stationary structure. In all computations, Ahlrichs’ def2-TZVP basis set was utilized.^36^ The solvation model based on density (SMD) was used to evaluate the effects of toluene (ε = 2.3741) and THF (ε = 7.4257) on the computed Gibbs energies.^37^ As depicted in Scheme 6b, first, the interaction of **2d** and In(OTf)_3_ leads to complex **H** with a relative energy of −10.8 and −13.0 kcal mol^–1^ in toluene and THF, respectively. Subsequently, the nucleophilic attack by the indole **1a** bifurcates into two paths which are N1- and C6-attacks. Our computations showed that N1-alkylation proceeds apparently barrierless. For the N1-alkylation, **H** → **B**, the computed reaction-free energies are 23.2 and 22.7 kcal mol^–1^ with toluene and THF, respectively. For C6-alkylation, **H** → **D**, while the computed reaction-free energies are 12.7 and 12.1 kcal mol^–1^, the activation-free energies are 19.3 and 20.0 kcal mol^–1^ with toluene and THF, respectively. The Gibbs free energies indicated that N1-alkylation in THF has lower energy by 0.5 kcal mol^–1^ compared to N1-alkylation in toluene. Additionally, we determined that the reaction barrier for C6-alkylation in toluene is lower in energy by 0.7 kcal mol^–1^ than C6-alkylation in THF. However, the N1-functionalized product **4ad** is higher in energy compared to the C6-functionalized product **3ad** by 7.6 and 8.2 kcal mol^–1^ in toluene and THF, respectively. The DFT results are rationally explained and supported our experimental observations that the C6-alkylation is more thermodynamically favorable, while the N1-alkylation is more kinetically favorable. Remote hydrogen bonding interactions between indole NH and catalyst lower the energy of the steps and promote the formation of the intermediates or transition states.

## Conclusion

In summary, we have developed highly regiodivergent alkylation reactions of 2,3-disubstituted indoles with *p*-QMs via indium(III)-catalyst in different solvents. The reactions feature kinetic and thermodynamic control, mild conditions, a broad substrate scope, and good functional group tolerance, providing an efficient method for the synthesis of C6- and N1-alkylated indole derivatives. Essentially, the reaction parameters (such as catalyst, solvent, and temperature) are found to be crucial for achieving in receiving high regioselectivity. Experimental and DFT studies supported that the reactions proceed through kinetic and thermodynamic control. Furthermore, the synthetic utility of the current approach and the alkylation products were demonstrated by scaling up to gram-scale, removing the *tert*-butyl group, and aryl–aryl coupling. Moreover, the protocol was successful for the late-stage modification of indomethacin as a biorelevant motif.

## Experimental Section

### General Methods

Commercially available reagents and solvents were used without further purification. NMR spectra were recorded at 400 MHz (^1^H) and at 100 MHz (^13^C{^1^H}), respectively. Chemical shifts (δ) are given in parts per million (ppm). Coupling constants (*J*) are reported in hertz (Hz). Signal multiplicities are abbreviated as follows: singlet (s), doublet (d), triplet (t), quartet (q), doublet of doublets (dd), multiple (m), and broad (b). Structural assignments were made with additional information from NOE experiments. High-resolution mass spectra (HRMS) were recorded using a quadrupole time-of-flight (QTOF) spectrometry device. Column chromatography was carried out using silica gel (70–230 mesh).

### Starting Materials

Substituted indoles (**1a**–**m**), 2-(hydroxy(phenyl)methyl)phenol (**9**), and *para*-quinone methides (**2a**–**x**) were prepared following the reported procedures, respectively.^10b,12d,38,39^

### General Procedure A: The Synthesis of C6-Alkylation Products

To a solution of indole (**1a**–**m** or indomethacin) (0.20 mmol, 1.0 equiv) and *p*-QM (**2a**–**x**) (0.22 mmol, 1.1 equiv) in toluene (0.1 M) was added In(OTf)_3_ (10 mol %), and the mixture was stirred at room temperature for 12 h. After the reaction was complete (monitored by TLC), the solvent was removed under reduced pressure and the residue was purified by silica gel column chromatography using hexane/EtOAc solvent mixture as the eluent.

### Large-Scale Synthesis of **3ad**

To a solution of indole **2a** (514 mg, 3.0 mmol) and *p*-QM (**2a**–**x**) (1.23 g, 3.3 mmol) in toluene (0.1 M) was added In(OTf)_3_ (169 mg, 0.3 mmol, 10 mol %), and the mixture was stirred at room temperature for 12 h. After the reaction was complete (monitored by TLC), the solvent was removed under reduced pressure and the residue was purified by silica gel column chromatography using hexane/EtOAc (95:5) to give the compound **3ad** (1.39 g, 85% yield).

#### (±)-2,6-Di-*tert*-butyl-4-(phenyl(2,3,4,9-tetrahydro-1*H*-carbazol-7-yl)methyl)phenol **(3aa)**

Purified by column chromatography on silica gel, eluent hexane/EtOAc (95:5), orange solid (82 mg, 88% yield; mp 83–84 °C). ^1^H NMR (400 MHz, CDCl_3_) δ 7.33 (bs, 1H), 7.25 (d, *J* = 7.9 Hz, 1H), 7.17–7.12 (m, 2H), 7.09–7.02 (m, 3H), 6.88 (s, 2H), 6.83–6.77 (m, 2H), 5.46 (s, 1H), 4.97 (s, 1H), 2.62–2.51 (m, 4H), 1.81–1.72 (m, 4H), 1.27 (s, 18H). ^13^C{^1^H} NMR (100 MHz, CDCl_3_) δ 152.0, 145.8, 138.2, 135.8, 135.4, 135.0, 134.0, 129.6, 128.0, 126.2, 126.0, 125.8, 121.4, 117.3, 111.3, 109.9, 57.1, 34.4, 30.4, 23.4, 23.30, 23.26, 21.0. HRMS (ESI-TOF) *m*/*z*: calcd for C_33_H_40_NO [M + H]^+^, 466.3104; found, 466.3086.

#### (±)-2,6-Di-*tert*-butyl-4-((4-fluorophenyl)(2,3,4,9-tetrahydro-1*H*-carbazol-7-yl)methyl)phenol **(3ab)**

Purified by column chromatography on silica gel, eluent hexane/EtOAc (95:5), orange solid (80 mg, 83% yield; mp 92–93 °C). ^1^H NMR (400 MHz, CDCl_3_) δ 7.54 (bs, 1H), 7.43 (d, *J* = 8.0 Hz, 1H), 7.19–7.13 (m, 2H), 7.03–6.94 (m, 6H), 5.61 (s, 1H), 5.16 (s, 1H), 2.79–2.69 (m, 4H), 1.97–1.90 (m, 4H), 1.44 (s, 18H). ^13^C{^1^H} NMR (100 MHz, CDCl_3_) δ 161.2 (d, *J* = 243.6 Hz), 152.1, 141.52 (d, *J* = 2.3 Hz), 138.0, 135.8, 135.5 (2C), 134.9, 134.1, 130.9 (d, *J* = 7.8 Hz), 126.1, 121.3, 117.4, 114.7 (d, *J* = 21.0 Hz), 111.2, 110.0, 56.3, 34.4, 30.4, 23.4, 23.30, 23.26, 21.0. HRMS (ESI-TOF) *m*/*z*: calcd for C_33_H_39_FNO [M + H]^+^, 484.3010; found, 484.3016.

#### (±)-2,6-Di-*tert*-butyl-4-((4-chlorophenyl)(2,3,4,9-tetrahydro-1*H*-carbazol-7-yl)methyl)phenol **(3ac)**

Purified by column chromatography on silica gel, eluent hexane/EtOAc (95:5), orange solid (85 mg, 85% yield; mp 108–109 °C). ^1^H NMR (400 MHz, CDCl_3_) δ 7.56 (bs, 1H), 7.39 (d, *J* = 8.1 Hz, 1H), 7.26–7.21 (m, AA’ part of AA’BB’ system, 2H), 7.11–7.07 (m, BB’ part of AA’BB’ system, 2H), 6.98 (s, 2H), 6.93 (s, 1H), 6.91–6.87 (m, 1H), 5.55 (s, 1H), 5.13 (s, 1H), 2.75–2.69 (m, 4H), 1.91 (d, *J* = 2.5 Hz, 4H), 1.40 (s, 18H). ^13^C{^1^H} NMR (100 MHz, CDCl_3_) δ 152.1, 144.4, 137.6, 135.8, 135.5, 134.5, 134.1, 131.5, 130.9, 128.1, 126.13, 126.06, 121.2, 117.4, 111.2, 110.0, 56.4, 34.4, 30.4, 23.32, 23.28, 23.2, 21.0. HRMS (ESI-TOF) *m*/*z*: calcd for C_33_H_39_ClNO [M + H]^+^, 500.2715; found, 500.2715.

#### (±)-4-((4-Bromophenyl)(2,3,4,9-tetrahydro-1*H*-carbazol-7-yl)methyl)-2,6-di-*tert*-butylphenol **(3ad)**

Purified by column chromatography on silica gel, eluent hexane/EtOAc (95:5), orange solid (100 mg, 92% yield; mp 119–120 °C). ^1^H NMR (400 MHz, CDCl_3_) δ 7.36 (bs, 1H), 7.27–7.22 (m, 3H), 6.91–6.88 (m, 2H), 6.84 (s, 2H), 6.79–6.73 (m, 2H), 5.39 (s, 1H), 4.98 (s, 1H), 2.60–2.51 (m, 4H), 1.78–1.72 (m, 4H), 1.26 (s, 18H). ^13^C{^1^H} NMR (100 MHz, CDCl_3_) δ 152.2, 145.0, 137.5, 135.8, 135.6 (2C), 134.5, 134.2, 131.4, 131.1, 126.1, 121.2, 119.7, 117.5, 111.3, 110.0, 56.6, 34.4, 30.4, 23.4, 23.30, 23.27, 21.0. HRMS (ESI-TOF) *m*/*z*: calcd for C_33_H_38_BrNO [M ]^+^, 543.2131; found, 543.2126.

#### (±)-2,6-Di-*tert*-butyl-4-((4-iodophenyl)(2,3,4,9-tetrahydro-1*H*-carbazol-7-yl)methyl)phenol **(3ae)**

Purified by column chromatography on silica gel, eluent hexane/EtOAc (95:5), orange solid (92 mg, 78% yield; mp 114–115 °C). ^1^H NMR (400 MHz, CDCl_3_) δ 7.60–7.54 (m, 3H), 7.36 (d, *J* = 8.1 Hz, 1H), 6.95 (s, 2H), 6.91–6.85 (m, 4H), 5.49 (s, 1H), 5.10 (s, 1H), 2.73–2.67 (m, 4H), 1.92–1.85 (m, 4H), 1.37 (s, 18H).^13^C{^1^H} NMR (100 MHz, CDCl_3_) δ 152.1, 145.6, 137.4, 137.0, 135.7, 135.5, 134.3, 134.1, 131.6, 126.1, 126.0, 121.2, 117.4, 111.2, 110.0, 91.1, 56.6, 34.4, 30.4, 23.3 (2C), 23.2, 21.0. HRMS (ESI-TOF) *m*/*z*: calcd for C_33_H_39_INO [M + H]^+^, 592.2071; found, 592.2061.

#### (±)-2,6-Di-*tert*-butyl-4-((2,3,4,9-tetrahydro-1*H*-carbazol-7-yl)(*p*-tolyl)methyl)phenol **(3af)**

Purified by column chromatography on silica gel, eluent hexane/EtOAc (95:5), orange solid (82 mg, 85% yield; mp 108–109 °C). ^1^H NMR (400 MHz, CDCl_3_) δ 7.52 (bs, 1H), 7.36 (d, *J* = 8.1 Hz, 1H), 7.08–7.02 (m, 4H), 6.99 (s, 2H), 6.94 (s, 1H), 6.93–6.89 (m, 1H), 5.52 (s, 1H), 5.07 (s, 1H), 2.73–2.67 (m, 4H), 2.33 (s, 3H), 1.93–1.85 (m, 4H), 1.38 (s, 18H).^13^C{^1^H} NMR (100 MHz, CDCl_3_) δ 151.9, 142.7, 138.5, 135.8, 135.3 (2C), 135.1, 133.8, 129.4, 128.7, 126.1, 125.9, 121.4, 117.2, 111.2, 109.9, 56.7, 34.4, 30.4, 23.33, 23.28, 23.2, 21.03, 21.01. HRMS (ESI-TOF) *m*/*z*: calcd for C_34_H_42_NO [M + H]^+^, 480.3261; found, 480.3242.

#### (±)-2,6-Di-*tert*-butyl-4-((4-(*tert*-butyl)phenyl)(2,3,4,9-tetrahydro-1*H*-carbazol-7-yl)methyl)phenol **(3ag)**

Purified by column chromatography on silica gel, eluent hexane/EtOAc (95:5), orange solid (91 mg, 87% yield; mp 119–120 °C). ^1^H NMR (400 MHz, CDCl_3_) δ 7.29 (bs, 1H), 7.24 (d, *J* = 8.4 Hz, 1H), 7.18–7.14 (m, AA’ part of AA’BB’ system, 2H), 6.98–6.94 (m, BB’ part of AA’BB’ system, 2H), 6.89 (s, 2H), 6.81 (t, *J* = 3.4 Hz, 2H), 5.41 (s, 1H), 4.95 (s, 1H), 2.61–2.53 (m, 4H), 1.80–1.73 (m, 4H), 1.27 (s, 18H), 1.20 (s, 9H).^13^C{^1^H} NMR (100 MHz, CDCl_3_) δ 151.9, 148.5, 142.6, 138.5, 135.8, 135.3, 135.2, 133.8, 129.1, 126.2, 125.9, 124.9, 121.4, 117.2, 111.3, 109.9, 56.7, 34.4 (2C), 31.5, 30.4, 23.4, 23.28, 23.26, 21.0. HRMS (ESI-TOF) *m*/*z*: calcd for C_37_H_48_NO [M + H]^+^, 522.3730; found, 522.3729.

#### (±)-2,6-Di-*tert*-butyl-4-((4-methoxyphenyl)(2,3,4,9-tetrahydro-1*H*-carbazol-7-yl)methyl)phenol **(3ah)**

Purified by column chromatography on silica gel, eluent hexane/EtOAc (95:5), orange solid (85 mg, 86% yield; mp 104–105 °C). ^1^H NMR (400 MHz, CDCl_3_) δ 7.54 (s, 1H), 7.37 (d, *J* = 8.1 Hz, 1H), 7.09–7.05 (m, AA’ part of AA’BB’ system, 2H), 7.00 (s, 2H), 6.94 (s, 1H), 6.91 (d, *J* = 8.1 Hz, 1H), 6.84–6.79 (m, BB’ part of AA’BB’ system, 2H), 5.53 (s, 1H), 5.08 (s, 1H), 3.80 (s, 3H), 2.74–2.67 (m, 4H), 1.90 (s, 4H), 1.39 (s, 18H). ^13^C{^1^H} NMR (100 MHz, CDCl_3_) δ 157.6, 151.9, 138.6, 138.0, 135.8, 135.3 (2C), 133.9, 130.4, 126.1, 125.9, 121.3, 117.2, 113.4, 111.2, 109.9, 56.2, 55.2, 34.4, 30.4, 23.34, 23.29, 23.2, 21.0. HRMS (ESI-TOF) *m*/*z*: calcd for C_34_H_42_NO_2_ [M + H]^+^, 496.3210 found, 496.3213.

#### (±)-4-((3,5-Di-*tert*-butyl-4-hydroxyphenyl)(2,3,4,9-tetrahydro-1*H*-carbazol-7-yl)methyl)benzoic Acid **(3ai)**

Purified by column chromatography on silica gel, eluent hexane/EtOAc (95:5), orange solid (90 mg, 88% yield; mp 146–147 °C). ^1^H NMR (400 MHz, CDCl_3_) δ 8.07–7.91 (m, 3H), 7.58 (s, 1H), 7.36 (d, *J* = 8.1 Hz, 1H), 7.25–7.17 (m, 2H), 6.97–6.90 (m, 3H), 6.86 (d, *J* = 8.0 Hz, 1H), 5.59 (s, 1H), 5.10 (s, 1H), 2.68 (s, 4H), 1.87 (s, 4H), 1.35 (s, 18H). ^13^C{^1^H} NMR (100 MHz, CDCl_3_) δ 172.2, 152.4, 137.3, 136.0, 135.7, 134.4, 134.3, 130.6, 130.2, 129.8, 126.4, 126.3, 125.7, 121.4, 117.7, 111.4, 110.2, 57.3, 34.6, 30.6, 23.50, 23.49, 23.4, 21.2. HRMS (ESI-TOF) *m*/*z*: calcd for C_34_H_40_NO_3_ [M + H]^+^, 510.3003; found, 510.3006.

#### (±)-2,6-Di-*tert*-butyl-4-((4-nitrophenyl)(2,3,4,9-tetrahydro-1*H*-carbazol-7-yl)methyl)phenol **(3aj)**

Purified by column chromatography on silica gel, eluent hexane/EtOAc (95:5), orange solid (85 mg, 83% yield; mp 103–104 °C). ^1^H NMR (400 MHz, CDCl_3_) δ 8.16–8.10 (m, AA’ part of AA’BB’ system, 2H), 7.66 (s, 1H), 7.41 (d, *J* = 8.1 Hz, 1H), 7.35–7.30 (m, BB’ part of AA’BB’ system, 2H), 6.97–6.94 (m, 3H), 6.87 (dd, *J* = 8.1, 1.3 Hz, 1H), 5.66 (s, 1H), 5.16 (s, 1H), 2.72 (t, *J* = 5.7 Hz, 4H), 1.95–1.86 (m, 4H), 1.39 (s, 18H). ^13^C{^1^H} NMR (100 MHz, CDCl_3_) δ 153.8, 152.4, 146.2, 136.3, 135.8, 134.5, 133.5, 130.3, 126.5, 126.0, 123.3 (2C), 121.0, 117.7, 111.2, 110.1, 57.0, 34.4, 30.3, 23.3 (2C), 23.2, 21.0. HRMS (ESI-TOF) *m*/*z*: calcd for C_33_H_39_N_2_O_3_ [M + H]^+^, 511.2955; found, 511.2940.

#### (±)-2,6-Di-*tert*-butyl-4-((2,3,4,9-tetrahydro-1*H*-carbazol-7-yl)(4-(trifluoromethyl)phenyl)methyl)phenol **(3ak)**

Purified by column chromatography on silica gel, eluent hexane/EtOAc (95:5), yellow solid (95 mg, 89% yield; mp 112–113 °C). ^1^H NMR (400 MHz, CDCl_3_) δ 7.58 (bs, 1H), 7.52–7.49 (m, AA’ part of AA’BB’ system, 2H), 7.38 (d, *J* = 8.1 Hz, 1H), 7.29–7.24 (m, BB’ part of AA’BB’ system, 2H), 6.95 (s, 2H), 6.92 (s, 1H), 6.87 (dd, *J* = 8.1, 1.2 Hz, 1H), 5.60 (s, 1H), 5.11 (s, 1H), 2.74–2.67 (m, 4H), 1.93–1.84 (m, 4H), 1.37 (s, 18H). ^13^C{^1^H} NMR (100 MHz, CDCl_3_) δ 152.3, 150.0, 137.1, 135.8, 135.6, 134.3, 134.1, 129.8, 128.1 (q, *J* = 32.2 Hz), 126.3, 126.1, 124.9 (q, *J* = 4.2 Hz), 124.5 (q, *J* = 271.5 Hz), 121.2, 117.6, 111.3, 110.0, 56.9, 34.4, 30.4, 23.31, 23.28, 23.2, 21.0. HRMS (ESI-TOF) *m*/*z*: calcd for C_34_H_39_F_3_NO [M + H]^+^, 534.2978; found, 534.2951.

#### (±)-2,6-Di-*tert*-butyl-4-((4-(diphenylamino)phenyl)(2,3,4,9-tetrahydro-1*H*-carbazol-7-yl)methyl)phenol **(3al)**

Purified by column chromatography on silica gel, eluent hexane/EtOAc (95:5), orange solid (101 mg, 80% yield; mp 115–116 °C). ^1^H NMR (400 MHz, CDCl_3_) δ 7.36 (s, 1H), 7.26 (d, *J* = 7.9 Hz, 1H), 7.12–7.07 (m, 4H), 6.97–6.92 (m, 6H), 6.90–6.82 (m, 8H), 5.42 (s, 1H), 4.96 (s, 1H), 2.61–2.53 (m, 4H), 1.80–1.72 (m, 4H), 1.28 (s, 18H). ^13^C{^1^H} NMR (100 MHz, CDCl_3_) δ 152.0, 148.0, 145.5, 140.6, 138.3, 135.8, 135.32, 135.29, 134.0, 130.4, 129.2, 126.2, 126.1, 124.5, 123.7, 122.3, 121.4, 117.3, 111.3, 110.0, 56.5, 34.4, 30.4, 23.4, 23.33, 23.28, 21.1. HRMS (ESI-TOF) *m*/*z*: calcd for C_45_H_49_N_2_O [M + H]^+^, 633.3839; found, 633.3814.

#### (±)-4-((2-Bromophenyl)(2,3,4,9-tetrahydro-1*H*-carbazol-7-yl)methyl)-2,6-di-*tert*-butylphenol **(3am)**

Purified by column chromatography on silica gel, eluent hexane/EtOAc (95:5), yellow solid (90 mg, 83% yield; mp 102–103 °C). ^1^H NMR (400 MHz, CDCl_3_) δ 7.59–7.55 (m, 1H), 7.51 (bs, 1H), 7.38 (d, *J* = 8.1 Hz, 1H), 7.22–7.17 (m, 1H), 7.09–7.03 (m, 2H), 6.96 (s, 2H), 6.92 (s, 1H), 6.89 (dd, *J* = 8.1, 1.3 Hz, 1H), 5.98 (s, 1H), 5.11 (s, 1H), 2.74–2.68 (m, 4H), 1.93–1.86 (m, 4H), 1.39 (s, 18H). ^13^C{^1^H} NMR (100 MHz, CDCl_3_) δ 152.1, 145.0, 136.7, 135.8, 135.3, 134.0, 133.6, 132.8, 131.5, 127.5, 127.0, 126.4, 126.1, 125.6, 121.4, 117.3, 111.5, 110.0, 56.1, 34.4, 30.4, 23.32, 23.29, 23.2, 21.0. HRMS (ESI-TOF) *m*/*z*: calcd for C_33_H_38_BrNO [M]^+^, 543.2131; found, 543.2139.

#### (±)-2,6-Di-*tert*-butyl-4-((2-hydroxyphenyl)(2,3,4,9-tetrahydro-1*H*-carbazol-7-yl)methyl)phenol **(3an)**(19)

Purified by column chromatography on silica gel, eluent hexane/EtOAc (95:5), orange solid (82 mg, 85% yield; mp 123–124 °C). ^1^H NMR (400 MHz, CDCl_3_) δ 7.45 (s, 1H), 7.29 (d, *J* = 8.0 Hz, 1H), 7.08–6.97 (m, 2H), 6.91 (s, 2H), 6.87 (s, 1H), 6.83 (d, *J* = 8.1 Hz, 1H), 6.75–6.71 (m, 3H), 5.53 (s, 1H), 5.02 (s, 1H), 2.61–2.53 (m, 4H), 1.81–1.72 (m, 4H), 1.27 (s, 18H). ^13^C{^1^H} NMR (100 MHz, CDCl_3_) δ 154.0, 152.5, 136.0, 135.9, 135.6, 134.4, 132.9, 131.8, 130.4, 127.7, 126.6, 126.0, 120.9, 120.5, 117.9, 116.5, 111.0, 110.0, 51.9, 34.4, 30.4, 23.29, 23.26, 23.2, 21.0. HRMS (ESI-TOF) *m*/*z*: calcd for C_33_H_40_NO_2_ [M + H]^+^, 482.3054; found, 482.3031.

#### (±)-2-((3,5-Di-*tert*-butyl-4-hydroxyphenyl)(2,3,4,9-tetrahydro-1*H*-carbazol-7-yl)methyl)phenyl Acetate **(3ao)**

Purified by column chromatography on silica gel, eluent hexane/EtOAc (95:5), orange solid (86 mg, 82% yield; mp 153–154 °C). ^1^H NMR (400 MHz, CDCl_3_) δ 7.61 (s, 1H), 7.41 (d, *J* = 8.0 Hz, 1H), 7.29–7.24 (m, 1H), 7.15–7.08 (m, 2H), 6.98–6.91 (m, 5H), 5.70 (s, 1H), 5.12 (s, 1H), 2.76–2.68 (m, 4H), 1.95 (s, 3H), 1.94–1.84 (m, 4H), 1.40 (s, 18H). ^13^C{^1^H} NMR (100 MHz, CDCl_3_) δ 168.9, 152.0, 148.7, 137.6, 136.4, 135.8, 135.4, 134.0, 133.8, 130.7, 126.9, 126.2, 125.9, 125.5, 122.7, 121.2, 117.4, 111.3, 109.9, 51.4, 34.3, 30.3, 23.32, 23.26, 23.2, 21.0, 20.6. HRMS (ESI-TOF) *m*/*z*: calcd for C_35_H_42_NO_3_ [M + H]^+^, 524.3159; found, 524.3160.

#### (±)-2,6-Di-*tert*-butyl-4-((3-hydroxyphenyl)(2,3,4,9-tetrahydro-1*H*-carbazol-7-yl)methyl)phenol **(3ap)**

Purified by column chromatography on silica gel, eluent hexane/EtOAc (95:5), orange solid (87 mg, 90% yield; mp 139–140 °C). ^1^H NMR (400 MHz, CDCl_3_) δ 7.42 (s, 1H), 7.39 (d, *J* = 8.1 Hz, 1H), 7.13 (t, *J* = 7.8 Hz, 1H), 7.02 (s, 2H), 6.95–6.90 (m, 2H), 6.75 (d, *J* = 7.6 Hz, 1H), 6.68–6.63 (m, 1H), 6.55 (s, 1H), 5.52 (s, 1H), 5.12 (s, 1H), 2.75–2.63 (m, 4H), 1.94–1.86 (m, 4H), 1.41 (s, 18H). ^13^C{^1^H} NMR (100 MHz, CDCl_3_) δ 155.4, 152.0, 147.7, 137.9, 135.8, 135.4, 134.8, 134.1, 129.1, 126.2, 126.0, 122.1, 121.4, 117.2, 116.6, 112.8, 111.4, 109.8, 56.9, 34.4, 30.4, 23.3, 23.2 (2C), 21.0. HRMS (ESI-TOF) *m*/*z*: calcd for C_33_H_40_NO_2_ [M + H]^+^, 482.3054; found, 482.3057.

#### (±)-2,6-Di-*tert*-butyl-4-((3,5-dibromophenyl)(2,3,4,9-tetrahydro-1*H*-carbazol-7-yl)methyl)phenol **(3aq)**

Purified by column chromatography on silica gel, eluent hexane/EtOAc (95:5), orange solid (108 mg, 87% yield; mp 115–116 °C). ^1^H NMR (400 MHz, CDCl_3_) δ 7.62 (s, 1H), 7.55 (t, *J* = 1.7 Hz, 1H), 7.43 (d, *J* = 8.1 Hz, 1H), 7.30 (d, *J* = 1.7 Hz, 2H), 6.98 (s, 2H), 6.95 (s, 1H), 6.90 (dd, *J* = 8.1, 1.3 Hz, 1H), 5.52 (s, 1H), 5.18 (s, 1H), 2.78–2.71 (m, 4H), 1.98–1.89 (m, 4H), 1.43 (s, 18H). ^13^C{^1^H} NMR (100 MHz, CDCl_3_) δ 152.4, 149.9, 136.4, 135.8, 135.7, 134.4, 133.4, 131.6, 131.3, 126.4, 126.0, 122.6, 121.1, 117.7, 111.2, 110.1, 56.6, 34.4, 30.4, 23.3 (2C), 23.2, 21.0. HRMS (ESI-TOF) *m*/*z*: calcd for C_33_H_37_Br_2_NO [M]^+^, 621.1236; found, 621.1242.

#### (±)-2,6-Di-*tert*-butyl-4-((2,5-dimethoxyphenyl)(2,3,4,9-tetrahydro-1*H*-carbazol-7-yl)methyl)phenol **(3ar)**

Purified by column chromatography on silica gel, eluent hexane/EtOAc (95:5), orange solid (95 mg, 90% yield; mp 108–109 °C). ^1^H NMR (400 MHz, CDCl_3_) δ 7.49 (s, 1H), 7.34 (d, *J* = 8.0 Hz, 1H), 6.99 (s, 2H), 6.93 (s, 1H), 6.90 (d, *J* = 8.0 Hz, 1H), 6.81 (d, *J* = 8.8 Hz, 1H), 6.72 (dd, *J* = 8.8, 3.0 Hz, 1H), 6.62–6.57 (m, 1H), 5.93 (s, 1H), 5.06 (s, 1H), 3.67 (s, 3H), 3.64 (s, 3H), 2.73–2.65 (m, 4H), 1.92–1.85 (m, 4H), 1.38 (s, 18H). ^13^C{^1^H} NMR (100 MHz, CDCl_3_) δ 153.4, 151.8, 151.7, 137.8, 135.9, 135.8, 135.1, 134.7, 133.6, 126.2, 125.9, 121.3, 117.2, 117.1, 112.0, 111.1, 111.0, 109.8, 56.6, 55.6, 49.7, 34.3, 30.4, 23.4, 23.2 (2C), 21.0. HRMS (ESI-TOF) *m*/*z*: calcd for C_35_H_44_NO_3_ [M + H]^+^, 526.3316; found, 526.3300.

#### (±)-2,6-Di-*tert*-butyl-4-(naphthalen-2-yl(2,3,4,9-tetrahydro-1*H*-carbazol-7-yl)methyl)phenol **(3as)**

Purified by column chromatography on silica gel, eluent hexane/EtOAc (95:5), orange solid (77 mg, 75% yield; mp 113–114 °C). ^1^H NMR (400 MHz, CDCl_3_) δ 7.83–7.79 (m, 1H), 7.75–7.70 (m, 2H), 7.54 (s, 1H), 7.51 (s, 1H), 7.45–7.41 (m, 2H), 7.40–7.33 (m, 2H), 7.06 (s, 2H), 6.99–6.94 (m, 2H), 5.73 (s, 1H), 5.11 (s, 1H), 2.73–2.67 (m, 4H), 1.94–1.86 (m, 4H), 1.38 (s, 18H). ^13^C{^1^H} NMR (100 MHz, CDCl_3_) δ 152.0, 143.4, 137.9, 135.8, 135.4, 134.7, 134.0, 133.4, 132.0, 128.5, 127.9, 127.6, 127.5, 127.4, 126.3, 126.0, 125.7, 125.3, 121.5, 117.3, 111.4, 110.0, 57.2, 34.4, 30.4, 23.33, 23.29, 23.2, 21.0. HRMS (ESI-TOF) *m*/*z*: calcd for C_37_H_42_NO [M + H]^+^, 516.3261; found, 516.3260.

#### (±)-2,6-Di-*tert*-butyl-4-(pyren-1-yl(2,3,4,9-tetrahydro-1*H*-carbazol-7-yl)methyl)phenol **(3au)**

Purified by column chromatography on silica gel, eluent hexane/EtOAc (95:5), orange solid (97 mg, 82% yield; mp 153–154 °C). ^1^H NMR (400 MHz, CDCl_3_) δ 8.40 (d, *J* = 9.4 Hz, 1H), 8.18–8.07 (m, 3H), 8.04 (s, 2H), 8.01–7.95 (m, 2H), 7.66 (d, *J* = 8.0 Hz, 1H), 7.40 (d, *J* = 8.1 Hz, 1H), 7.33 (s, 1H), 7.10 (s, 2H), 7.03–6.98 (m, 1H), 6.89 (s, 1H), 6.67 (s, 1H), 5.14 (s, 1H), 2.74–2.67 (m, 2H), 2.64–2.56 (m, 2H), 1.89–1.82 (m, 4H), 1.39 (s, 18H). ^13^C{^1^H} NMR (100 MHz, CDCl_3_) δ 152.1, 139.8, 138.3, 135.9, 135.5, 135.1, 133.9, 131.4, 130.8, 129.8, 129.1, 127.9, 127.6, 127.3, 126.8, 126.6, 126.0, 125.8, 125.1, 124.9 (2C), 124.8, 124.5, 124.2, 121.6, 117.4, 111.6, 109.9, 53.5, 34.4, 30.4, 23.3, 23.21, 23.18, 21.0. HRMS (APCI-TOF) *m*/*z*: calcd for C_43_H_43_NO [M]^+^, 591.3496; found, 591.3462.

#### (±)-4,4′-(1,4-Phenylenebis((2,3,4,9-tetrahydro-1*H*-carbazol-7-yl)methylene))bis(2,6-di-*tert*-butylphenol) **(3av)**

Purified by column chromatography on silica gel, eluent hexane/EtOAc (95:5), orange solid (142 mg, 83% yield; mp 179–180 °C). ^1^H NMR (400 MHz, CDCl_3_) δ 7.50–7.44 (m, 2H), 7.38–7.33 (m, 2H), 7.07–7.03 (m, 4H), 7.00 (s, 4H), 6.95–6.89 (m, 4H), 5.54 (s, 2H), 5.09–5.05 (m, 2H), 2.74–2.66 (m, 8H), 1.92–1.84 (m, 8H), 1.39 (s, 36H). ^13^C{^1^H} NMR (100 MHz, CDCl_3_) δ 151.9, 143.01, 142.95, 138.42, 138.40, 135.8, 135.3, 135.25, 135.21, 133.8, 129.1, 126.2, 125.9, 121.4, 117.1, 111.3, 109.9, 56.7, 34.4, 30.4, 23.34, 23.27, 23.2, 21.0 (Due to a mixture of diastereomer, all ^13^C{^1^H} NMR signals could not be resolved). HRMS (ESI-TOF) *m*/*z*: calcd for C_60_H_73_N_2_O_2_ [M + H]^+^, 853.5667; found, 853.5642.

#### (±)-2,6-Di-*tert*-butyl-4-((2,3,4,9-tetrahydro-1*H*-carbazol-7-yl)(1-tosyl-1*H*-indol-3-yl)methyl)phenol **(3ax)**

Purified by column chromatography on silica gel, eluent hexane/EtOAc (95:5), orange solid (112 mg, 85% yield; mp 140–141 °C). ^1^H NMR (400 MHz, CDCl_3_) δ 8.06 (d, *J* = 8.2 Hz, 1H), 7.78–7.72 (m, AA’ part of AA’BB’ system, 2H), 7.55 (s, 1H), 7.41 (d, *J* = 8.0 Hz, 1H), 7.32–7.23 (m, 3H), 7.16 (d, *J* = 7.7 Hz, 1H), 7.10–7.01 (m, 5H), 6.97 (d, *J* = 8.0 Hz, 1H), 5.58 (s, 1H), 5.16 (s, 1H), 2.79–2.68 (m, 4H), 2.40 (s, 3H), 1.93 (d, *J* = 4.5 Hz, 4H), 1.41 (s, 18H). ^13^C{^1^H} NMR (100 MHz, CDCl_3_) δ 152.2, 144.6, 135.9 (2C), 135.7, 135.5, 135.3, 134.1, 133.5, 131.0, 129.8, 128.9, 126.8, 126.4, 125.6, 125.4, 124.5, 123.1, 121.0, 120.7, 117.5, 113.8, 110.7, 109.9, 48.7, 34.4, 30.4, 23.33, 23.27, 23.2, 21.6, 21.0. HRMS (ESI-TOF) *m*/*z*: calcd for C_42_H_47_N_2_O_3_S [M + H]^+^, 659.3302; found, 659.3300.

#### (±)-4-((4-Bromophenyl)(1,2,3,4-tetrahydrocyclopenta[*b*]indol-6-yl)methyl)-2,6-di-*tert*-butylphenol **(3bd)**

Purified by column chromatography on silica gel, eluent hexane/EtOAc (95:5), orange solid (451 mg, 85% yield; mp 102–103 °C). ^1^H NMR (400 MHz, CDCl_3_) δ 7.70 (s, 1H), 7.41–7.34 (m, 3H), 7.06–7.00 (m, 2H), 6.97–6.93 (m, 3H), 6.89–6.86 (m, 1H), 5.51 (s, 1H), 5.11 (s, 1H), 2.86–2.80 (m, 4H), 2.57–2.49 (m, 2H), 1.38 (s, 18H). ^13^C{^1^H} NMR (100 MHz, CDCl_3_) δ 152.1, 144.9, 143.7, 141.2, 137.0, 135.5, 134.4, 131.3, 131.0, 126.0, 123.1, 121.6, 119.7, 119.6, 118.1, 112.1, 56.4, 34.4, 30.4, 28.7, 25.9, 24.5. HRMS (ESI-TOF) *m*/*z*: calcd for C_32_H_36_BrNO [M]^+^, 529.1959; found, 529.1979.

#### (±)-4-((4-Bromophenyl)(5,6,7,8,9,10-hexahydrocyclohepta[*b*]indol-3-yl)methyl)-2,6-di-*tert*-butylphenol **(3cd)**

Purified by column chromatography on silica gel, eluent hexane/EtOAc (95:5), orange solid (97 mg, 87% yield; mp 111–112 °C). ^1^H NMR (400 MHz, CDCl_3_) δ 7.58 (bs, 1H), 7.41–7.35 (m, 3H), 7.05–7.00 (m, 2H), 6.96 (s, 2H), 6.91–6.86 (m, 2H), 5.51 (s, 1H), 5.11 (s, 1H), 2.83–2.78 (m, 4H), 1.92–1.87 (m, 2H), 1.81–1.75 (m, 4H), 1.38 (s, 18H). ^13^C{^1^H} NMR (100 MHz, CDCl_3_) δ 152.1, 144.9, 137.4, 137.1, 135.4, 134.34, 134.27, 131.3, 131.0, 127.6, 126.0, 121.1, 119.6, 117.4, 113.6, 111.0, 56.4, 34.4, 31.8, 30.4, 29.6, 28.7, 27.5, 24.8. HRMS (ESI-TOF) *m*/*z*: calcd for C_34_H_41_BrNO [M + H]^+^, 558.2366; found, 558.2341.

#### (±)-4-((4-Bromophenyl)(6,7,8,9,10,11-hexahydro-5*H*-cycloocta[*b*]indol-3-yl)methyl)-2,6-di-*tert*-butylphenol **(3dd)**

Purified by column chromatography on silica gel, eluent hexane/EtOAc (95:5), yellow solid (92 mg, 80% yield; mp 106–107 °C). ^1^H NMR (400 MHz, CDCl_3_) δ 7.58 (s, 1H), 7.43–7.37 (m, 3H), 7.08–7.04 (m, 2H), 6.98 (s, 2H), 6.92–6.88 (m, 2H), 5.52 (s, 1H), 5.13 (s, 1H), 2.90–2.80 (m, 4H), 1.78–1.72 (m, 4H), 1.50–1.45 (m, 4H), 1.40 (s, 18H). ^13^C{^1^H} NMR (100 MHz, CDCl_3_) δ 152.1, 145.0, 137.0, 135.5, 135.4, 135.1, 134.3, 131.3, 131.0, 127.0, 126.0, 121.0, 119.6, 117.3, 111.5, 111.0, 56.5, 34.4, 34.3, 30.4, 29.5, 29.4, 26.1, 25.9, 22.3. HRMS (ESI-TOF) *m*/*z*: calcd for C_35_H_42_BrNO [M]^+^, 571.2444; found, 571.2451.

#### (±)-4-((4-Bromophenyl)(2,3-dimethyl-1*H*-indol-6-yl)methyl)-2,6-di-*tert*-butylphenol **(3ed)**

Purified by column chromatography on silica gel, eluent hexane/EtOAc (95:5), yellow solid (91 mg, 88% yield; mp 119–120 °C). ^1^H NMR (400 MHz, CDCl_3_) δ 7.58 (s, 1H), 7.40–7.35 (m, 3H), 7.04–7.00 (m, 2H), 6.95 (s, 2H), 6.90–6.86 (m, 2H), 5.51 (s, 1H), 5.11 (s, 1H), 2.33 (s, 3H), 2.22 (s, 3H), 1.38 (s, 18H). ^13^C{^1^H} NMR (100 MHz, CDCl_3_) δ 151.0, 143.8, 136.3, 134.3, 134.1, 133.2, 130.2, 130.0, 129.5, 126.6, 125.0, 120.0, 118.6, 116.6, 109.8, 105.8, 55.3, 33.3, 29.3, 10.5, 7.4. HRMS (ESI-TOF) *m*/*z*: calcd for C_31_H_36_BrNO [M]^+^, 517.1975; found, 517.1978.

#### (±)-4-((4-Bromophenyl)(2-ethyl-3-methyl-1*H*-indol-6-yl)methyl)-2,6-di-*tert*-butylphenol **(3fd)**

Purified by column chromatography on silica gel, eluent hexane/EtOAc (95:5), white solid (89 mg, 84% yield; mp 93–94 °C). ^1^H NMR (400 MHz, CDCl_3_) δ 7.62 (bs, 1H), 7.42–7.35 (m, 3H), 7.05–7.00 (m, 2H), 6.95 (s, 2H), 6.91–6.86 (m, 2H), 5.51 (s, 1H), 5.10 (s, 1H), 2.74 (q, *J* = 7.6 Hz, 2H), 2.23 (s, 3H), 1.38 (s, 18H), 1.26 (t, *J* = 7.6 Hz, 3H).^13^C{^1^H} NMR (100 MHz, CDCl_3_) δ 152.1, 144.9, 137.4, 136.4, 135.5, 135.2, 134.3, 131.3, 131.0, 127.8, 126.0, 121.1, 119.6, 117.7, 111.0, 106.0, 56.5, 34.4, 30.4, 19.4, 14.0, 8.4. HRMS (ESI-TOF) *m*/*z*: calcd for C_32_H_39_BrNO [M + H]^+^, 532.2210; found, 532.2197.

#### (±)-4-((4-Bromophenyl)(3-ethyl-2-phenyl-1*H*-indol-6-yl)methyl)-2,6-di-*tert*-butylphenol **(3gd)**

Purified by column chromatography on silica gel, eluent hexane/EtOAc (95:5), light yellow solid (102 mg, 86% yield; mp 99–100 °C). ^1^H NMR (400 MHz, CDCl_3_) δ 7.88 (s, 1H), 7.57–7.51 (m, 3H), 7.48–7.43 (m, 2H), 7.41–7.32 (m, 3H), 7.07–7.02 (m, 2H), 7.00–6.91 (m, 4H), 5.53 (s, 1H), 5.10 (s, 1H), 2.90 (q, *J* = 7.4 Hz, 2H), 1.38 (s, 18H), 1.34 (t, *J* = 7.4 Hz, 3H). ^13^C{^1^H} NMR (100 MHz, CDCl_3_) δ 152.2, 144.6, 138.8, 136.1, 135.6, 134.1, 133.7, 133.4, 131.3, 131.1, 128.8, 127.7, 127.42, 127.38, 126.0, 121.6, 119.7, 118.9, 115.3, 111.4, 56.5, 34.4, 30.4, 17.9, 15.6. HRMS (ESI-TOF) *m*/*z*: calcd for C_37_H_41_BrNO [M + H]^+^, 594.2366; found, 594.2361.

#### (±)-4-((4-Bromophenyl)(6-methyl-2,3,4,9-tetrahydro-1*H*-carbazol-7-yl)methyl)-2,6-di-*tert*-butylphenol **(3hd)**

Purified by column chromatography on silica gel, eluent hexane/EtOAc (95:5), white solid (97 mg, 87% yield; mp 131–132 °C). ^1^H NMR (400 MHz, CDCl_3_) δ 7.48 (bs, 1H), 7.38–7.32 (m, 2H), 7.24 (s, 1H), 6.97–6.93 (m, 2H), 6.85 (s, 2H), 6.64 (s, 1H), 5.60 (s, 1H), 5.09 (s, 1H), 2.71–2.66 (m, 4H), 2.26 (s, 3H), 1.92–1.83 (m, 4H), 1.36 (s, 18H). ^13^C{^1^H} NMR (100 MHz, CDCl_3_) δ 152.0, 144.0, 136.4, 135.4, 134.3, 134.2, 134.0, 131.4, 131.0, 127.5, 126.3, 126.2, 119.5, 118.8, 111.6, 109.5, 53.3, 34.3, 30.4, 23.34, 23.27, 23.2, 21.0, 20.3. HRMS (ESI-TOF) *m*/*z*: calcd for C_34_H_41_BrNO [M + H]^+^, 558.2366; found, 558.2367.

#### (±)-4-((4-Bromophenyl)(6-methoxy-2,3,4,9-tetrahydro-1*H*-carbazol-7-yl)methyl)-2,6-di-*tert*-butylphenol **(3id)**

Purified by column chromatography on silica gel, eluent hexane/EtOAc (95:5), yellow solid (95 mg, 83% yield; mp 129–130 °C). ^1^H NMR (400 MHz, CDCl_3_) δ 7.48 (bs, 1H), 7.35–7.30 (m, 2H), 7.00–6.96 (m, 2H), 6.93–6.88 (m, 3H), 6.66 (s, 1H), 5.83 (s, 1H), 5.08 (s, 1H), 3.71 (s, 3H), 2.72–2.66 (m, 4H), 1.94–1.86 (m, 4H), 1.37 (s, 18H). ^13^C{^1^H} NMR (100 MHz, CDCl_3_) δ 152.0, 151.9, 144.7, 135.3, 134.5, 134.3, 131.1, 130.8, 130.2, 128.3, 126.32, 126.29, 119.2, 112.1, 109.8, 99.5, 56.4, 49.8, 34.4, 30.4, 23.4 (2C), 23.2, 21.1. HRMS (ESI-TOF) *m*/*z*: calcd for C_34_H_41_BrNO_2_ [M + H]^+^, 574.2315; found, 574.2325.

#### (±)-4-((4-Bromophenyl)(6-chloro-2,3,4,9-tetrahydro-1*H*-carbazol-7-yl)methyl)-2,6-di-*tert*-butylphenol **(3jd)**

Purified by column chromatography on silica gel, eluent hexane/EtOAc (95:5), white solid (85 mg, 73% yield; mp 231–232 °C). ^1^H NMR (400 MHz, CDCl_3_) δ 7.59 (bs, 1H), 7.46 (s, 1H), 7.38–7.34 (m, 2H), 6.98–6.94 (m, 2H), 6.87 (s, 2H), 6.74 (s, 1H), 5.89 (s, 1H), 5.11 (s, 1H), 2.71–2.63 (m, 4H), 1.92–1.84 (m, 4H), 1.36 (s, 18H). ^13^C{^1^H} NMR (100 MHz, CDCl_3_) δ 152.2, 143.3, 135.6, 135.5, 134.5, 134.2, 133.5, 131.2, 131.0, 127.4, 126.3, 125.7, 119.7, 118.3, 112.7, 109.8, 53.2, 34.4, 30.4, 23.2, 23.1, 23.0, 20.8. HRMS (ESI-TOF) *m*/*z*: calcd for C_33_H_38_BrClNO [M + H]^+^, 578.1820; found, 578.1802.

#### (±)-4-((4-Bromophenyl)(9-methyl-2,3,4,9-tetrahydro-1*H*-carbazol-7-yl)methyl)-2,6-di-*tert*-butylphenol **(3kd)**

Purified by column chromatography on silica gel, eluent hexane/EtOAc (95:5), yellow solid (96 mg, 86% yield; mp 78–79 °C). ^1^H NMR (400 MHz, CDCl_3_) δ 7.39–7.35 (m, 3H), 7.04–7.01 (m, 2H), 6.98–6.96 (m, 3H), 6.86–6.82 (m, 1H), 5.53 (s, 1H), 5.10 (s, 1H), 3.53 (s, 3H), 2.74–2.68 (m, 4H), 1.96–1.84 (m, 4H), 1.38 (s, 18H). ^13^C{^1^H} NMR (100 MHz, CDCl_3_) δ 152.1, 145.1, 136.8, 135.8, 135.4, 134.4, 131.2 (2C), 131.0, 126.0, 125.5, 120.6, 119.6, 117.4, 109.3, 109.0, 56.7, 34.4, 30.4, 28.9, 23.2 (2C), 22.1, 21.1. HRMS (ESI-TOF) *m*/*z*: calcd for C_34_H_41_BrNO [M + H]^+^, 558.2366; found, 558.2370.

#### (±)-4-((9-Benzyl-2,3,4,9-tetrahydro-1*H*-carbazol-7-yl)(4-bromophenyl)methyl)-2,6-di-*tert*-butylphenol **(3ld)**

Purified by column chromatography on silica gel, eluent hexane/EtOAc (95:5), orange solid (94 mg, 74% yield; mp 183–184 °C). ^1^H NMR (400 MHz, CDCl_3_) δ 7.44 (d, *J* = 8.0 Hz, 1H), 7.39–7.34 (m, 2H), 7.30–7.24 (m, 3H), 7.01–6.95 (m, 4H), 6.94–6.88 (m, 4H), 5.50 (s, 1H), 5.22–5.11 (m, 2H), 5.10 (s, 1H), 2.81–2.75 (m, 2H), 2.69–2.61 (m, 2H), 1.96–1.86 (m, 4H), 1.36 (s, 18H). ^13^C{^1^H} NMR (100 MHz, CDCl_3_) δ 152.0, 145.0, 138.2, 137.2, 136.7, 135.7, 135.4, 134.3, 131.2, 131.0, 128.7, 127.2, 126.2, 126.0, 125.8, 121.0, 119.6, 117.5, 109.8, 109.7, 56.6, 46.2, 34.3, 30.3, 23.3, 23.2, 22.2, 21.2. HRMS (ESI-TOF) *m*/*z*: calcd for C_40_H_45_BrNO [M + H]^+^, 634.2679; found, 634.2651.

### General Procedure B: The Synthesis of N1-Alkylation Products

To a solution of indole (**1a**–**m**) (0.30 mmol, 1.5 equiv) and *p*-QM (**2a**–**v**) (0.20 mmol, 1.0 equiv) in THF (0.1 M) was added In(OTf)_3_ (10 mol %), and the mixture was stirred at room temperature for 12 h. After the reaction was complete (monitored by TLC), the solvent was removed under reduced pressure. The residue was dissolved in DCM (10 mL) and the organic phase was washed with water (3 × 10 mL). The aqueous phase was extracted three times with DCM (10 mL). The organic phases were combined, dried over Na_2_SO_4_, and concentrated under reduced pressure. The crude product was purified by silica gel column chromatography using hexane/EtOAc solvent mixture as the eluent.

#### (±)-2,6-Di-*tert*-Butyl-4-(phenyl(1,2,3,4-tetrahydro-9*H*-carbazol-9-yl)methyl)phenol **(4aa)**

Purified by column chromatography on silica gel, eluent hexane/EtOAc (98:2), yellow solid (80 mg, 86% yield; mp 81–82 °C). ^1^H NMR (400 MHz, CDCl_3_) δ 7.51 (d, *J* = 7.7 Hz, 1H), 7.35–7.28 (m, 3H), 7.18–7.13 (m, 2H), 7.06 (t, *J* = 7.2 Hz, 1H), 7.00 (s, 2H), 6.98–6.95 (m, 1H), 6.91 (d, *J* = 8.2 Hz, 1H), 6.85 (s, 1H), 5.24 (s, 1H), 2.83–2.77 (m, 2H), 2.51–2.45 (m, 2H), 1.90–1.83 (m, 4H), 1.39–1.36 (m, 18H). ^13^C{^1^H} NMR (100 MHz, CDCl_3_) δ 153.1, 140.9, 136.7, 136.4, 135.7, 129.9, 128.3, 128.2, 128.0, 127.2, 125.4, 120.4, 118.5, 117.5, 111.2, 110.4, 62.2, 34.4, 30.3, 23.9, 23.7, 23.1, 21.3. HRMS (ESI-TOF) *m*/*z*: calcd for C_33_H_40_NO [M + H]^+^, 466.3104; found, 466.3075.

#### (±)-2,6-Di-*tert*-butyl-4-((4-fluorophenyl)(1,2,3,4-tetrahydro-9*H*-carbazol-9-yl)methyl)phenol **(4ab)**

Purified by column chromatography on silica gel, eluent hexane/EtOAc (98:2), yellow solid (79 mg, 82% yield; mp 76–77 °C). ^1^H NMR (400 MHz, CDCl_3_) δ 7.48 (d, *J* = 7.6 Hz, 1H), 7.10–6.93 (m, 8H), 6.85 (d, *J* = 8.3 Hz, 1H), 6.78 (s, 1H), 5.22 (s, 1H), 2.79–2.75 (m, 2H), 2.47–2.43 (m, 2H), 1.87–1.82 (m, 4H), 1.35 (s, 18H). ^13^C{^1^H} NMR (100 MHz, CDCl_3_) δ 161.9 (d, *J* = 245.9 Hz), 153.2, 136.7 (d, *J* = 3.2 Hz), 136.6, 136.2, 135.8, 129.8 (d, *J* = 8.1 Hz), 129.6, 128.0, 125.3, 120.4, 118.6, 117.5, 115.2 (d, *J* = 21.4 Hz), 111.1, 110.6, 61.5, 34.4, 30.3, 23.9, 23.6, 23.0, 21.2. HRMS (ESI-TOF) *m*/*z*: calcd for C_33_H_39_FNO [M + H]^+^, 484.3010; found, 484.2987.

#### (±)-2,6-Di-*tert*-butyl-4-((4-chlorophenyl)(1,2,3,4-tetrahydro-9*H*-carbazol-9-yl)methyl)phenol **(4ac)**

Purified by column chromatography on silica gel, eluent hexane/EtOAc (98:2), yellow solid (84 mg, 84% yield; mp 92–93 °C). ^1^H NMR (400 MHz, CDCl_3_) δ 7.48 (d, *J* = 7.7 Hz, 1H), 7.29–7.24 (m, 2H), 7.06–7.01 (m, 3H), 6.98–6.93 (m, 3H), 6.86 (d, *J* = 8.2 Hz, 1H), 6.76 (s, 1H), 5.23 (s, 1H), 2.81–2.74 (m, 2H), 2.48–2.41 (m, 2H), 1.87–1.79 (m, 4H), 1.35 (s, 18H). ^13^C{^1^H} NMR (100 MHz, CDCl_3_) δ 153.2, 139.5, 136.6, 136.1, 135.9, 133.0, 129.6, 129.3, 128.5, 128.1, 125.3, 120.5, 118.7, 117.6, 111.1, 110.7, 61.6, 34.4, 30.3, 23.9, 23.7, 23.0, 21.2. HRMS (ESI-TOF) *m*/*z*: calcd for C_33_H_38_ClNO [M]^+^, 499.2636; found, 499.2613

#### (±)-2,6-Di-*tert*-butyl-4-((4-chlorophenyl)(1,2,3,4-tetrahydro-9*H*-carbazol-9-yl)methyl)phenol **(4ad)**

Purified by column chromatography on silica gel, eluent hexane/EtOAc (98:2), yellow solid (98 mg, 90% yield; mp 93–94 °C). ^1^H NMR (400 MHz, CDCl_3_) δ 7.48 (d, *J* = 7.7 Hz, 1H), 7.29–7.24 (m, 2H), 7.06–7.01 (m, 3H), 6.98–6.93 (m, 3H), 6.86 (d, *J* = 8.2 Hz, 1H), 6.76 (s, 1H), 5.23 (s, 1H), 2.81–2.74 (m, 2H), 2.48–2.41 (m, 2H), 1.87–1.79 (m, 4H), 1.35 (s, 18H). ^13^C{^1^H} NMR (100 MHz, CDCl_3_) δ 153.2, 139.5, 136.6, 136.1, 135.9, 133.0, 129.6, 129.3, 128.5, 128.1, 125.3, 120.5, 118.7, 117.6, 111.1, 110.7, 61.6, 34.4, 30.3, 23.9, 23.7, 23.0, 21.2. HRMS (ESI-TOF) *m*/*z*: calcd for C_33_H_39_BrNO [M + H]^+^, 544.2210; found, 544.2226.

#### (±)-2,6-Di-*tert*-butyl-4-((1,2,3,4-tetrahydro-9*H*-carbazol-9-yl)(p-tolyl)methyl)phenol **(4af)**

Purified by column chromatography on silica gel, eluent hexane/EtOAc (98:2), yellow solid (85 mg, 89% yield; mp 94–95 °C). ^1^H NMR (400 MHz, CDCl_3_) δ 7.51 (d, *J* = 7.7 Hz, 1H), 7.15–7.11 (m, AA’ part of AA’BB’ system, 2H), 7.07–7.03 (m, 3H), 7.00 (s, 2H), 6.98–6.92 (m, 2H), 6.82 (s, 1H), 5.22 (s, 1H), 2.84–2.78 (m, 2H), 2.55–2.46 (m, 2H), 2.37 (s, 3H), 1.87 (s, 4H), 1.38 (s, 18H). ^13^C{^1^H} NMR (100 MHz, CDCl_3_) δ 153.0, 137.8, 136.8, 136.7, 136.4, 135.7, 130.0, 129.0, 128.2, 128.0, 125.3, 120.3, 118.4, 117.4, 111.2, 110.3, 61.9, 34.4, 30.3, 23.9, 23.7, 23.1, 21.3, 21.2. HRMS (ESI-TOF) *m*/*z*: calcd for C_34_H_42_NO [M + H]^+^, 480.3261; found, 480.3231.

#### (±)-2,6-Di-*tert*-butyl-4-((4-(*tert*-butyl)phenyl)(1,2,3,4-tetrahydro-9*H*-carbazol-9-yl)methyl)phenol **(4ag)**

Purified by column chromatography on silica gel, eluent hexane/EtOAc (98:2), yellow solid (89 mg, 85% yield; mp 93–94 °C). ^1^H NMR (400 MHz, CDCl_3_) δ 7.48 (d, *J* = 7.7 Hz, 1H), 7.34–7.28 (m, AA’ part of AA’BB’ system, 2H), 7.06–7.00 (m, 3H), 6.97–6.89 (m, 4H), 6.78 (s, 1H), 5.19 (s, 1H), 2.81–2.75 (m, 2H), 2.52–2.40 (m, 2H), 1.88–1.80 (m, 4H), 1.34 (s, 18H), 1.32 (s, 9H). ^13^C{^1^H} NMR (100 MHz, CDCl_3_) δ 153.0, 150.1, 137.8, 136.7, 136.4, 135.6, 130.1, 128.0, 127.9, 125.3, 125.1, 120.2, 118.4, 117.4, 111.3, 110.2, 61.8, 34.5, 34.3, 31.4, 30.3, 23.8, 23.7, 23.1, 21.3. HRMS (ESI-TOF) *m*/*z*: calcd for C_37_H_47_NO [M]^+^, 521.3652; found, 521.3632.

#### (±)-2,6-Di-*tert*-butyl-4-((4-methoxyphenyl)(1,2,3,4-tetrahydro-9*H*-carbazol-9-yl)methyl)phenol **(4ah)**

Purified by column chromatography on silica gel, eluent hexane/EtOAc (98:2), yellow solid (83 mg, 84% yield; mp 84–85 °C). ^1^H NMR (400 MHz, CDCl_3_) δ 7.48 (d, *J* = 7.7 Hz, 1H), 7.06–7.01 (m, 3H), 6.97–6.89 (m, 4H), 6.86–6.81 (m, 2H), 6.78 (s, 1H), 5.20 (s, 1H), 3.80 (s, 3H), 2.80–2.74 (m, 2H), 2.53–2.40 (m, 2H), 1.88–1.80 (m, 4H), 1.35 (s, 18H). ^13^C{^1^H} NMR (100 MHz, CDCl_3_) δ 158.6, 153.0, 136.7, 136.3, 135.7, 133.0, 130.1, 129.4, 128.0, 125.2, 120.3, 118.4, 117.4, 113.6, 111.2, 110.3, 61.6, 55.2, 34.3, 30.3, 23.9, 23.7, 23.1, 21.3. HRMS (ESI-TOF) *m*/*z*: calcd for C_34_H_42_NO_2_ [M + H]^+^, 496.3210; found, 496.3193.

#### (±)-2,6-Di-*tert*-butyl-4-((4-nitrophenyl)(1,2,3,4-tetrahydro-9H-carbazol-9-yl)methyl)phenol **(4aj)**

Purified by column chromatography on silica gel, eluent hexane/EtOAc (98:2), yellow solid (73 mg, 71% yield; mp 90–91 °C). ^1^H NMR (400 MHz, CDCl_3_) δ 8.18–8.13 (m, 2H), 7.49 (d, *J* = 7.7 Hz, 1H), 7.29–7.25 (m, 2H), 7.07–7.03 (m, 1H), 6.98–6.93 (m, 3H), 6.84 (s, 1H), 6.80 (d, *J* = 8.2 Hz, 1H), 5.28 (s, 1H), 2.79–2.75 (m, 2H), 2.48–2.41 (m, 2H), 1.88–1.82 (m, 4H), 1.35 (s, 18H). ^13^C{^1^H} NMR (100 MHz, CDCl_3_) δ 153.6, 148.5, 147.1, 136.4, 136.2, 135.9, 129.0, 128.4, 128.2, 125.4, 123.6, 120.8, 119.0, 117.8, 111.2, 110.9, 61.8, 34.4, 30.2, 23.8, 23.6, 22.9, 21.2. HRMS (ESI-TOF) *m*/*z*: calcd for C_33_H_39_N_2_O_3_ [M + H]^+^, 511.2955; found, 511.2947.

#### (±)-2,6-Di-*tert*-butyl-4-((1,2,3,4-tetrahydro-9*H*-carbazol-9-yl)(4-(trifluoromethyl)phenyl)methyl)phenol **(4ak)**

Purified by column chromatography on silica gel, eluent hexane/EtOAc (98:2), yellow solid (86 mg, 81% yield; mp 86–87 °C). ^1^H NMR (400 MHz, CDCl_3_) δ 7.47–7.41 (m, AA’ part of AA’BB’ system, 2H), 7.38 (d, *J* = 7.7 Hz, 1H), 7.14–7.08 (m, BB’ part of AA’BB’ system, 2H), 6.93 (t, *J* = 7.3 Hz, 1H), 6.88–6.82 (m, 3H), 6.76–6.71 (m, 2H), 5.14 (s, 1H), 2.70–2.64 (m, 2H), 2.36–2.30 (m, 2H), 1.77–1.70 (m, 4H), 1.24 (s, 18H). ^13^C{^1^H} NMR (100 MHz, CDCl_3_) δ 153.4, 145.1, 136.6, 136.1, 136.0, 129.5 (q, *J* = 32.5 Hz), 129.0, 128.5, 128.1, 125.4, 125.3 (q, *J* = 3.3 Hz), 124.2 (q, *J* = 272.0 Hz), 120.6, 118.8, 117.7, 111.0, 110.9, 61.8, 34.4, 30.2, 23.9, 23.6, 23.0, 21.2. HRMS (ESI-TOF) *m*/*z*: calcd for C_34_H_39_F_3_NO [M + H]^+^, 534.2978; found, 534.2973.

#### (±)-2,6-Di-*tert*-butyl-4-((4-(diphenylamino)phenyl)(1,2,3,4-tetrahydro-9*H*-carbazol-9-yl)methyl)phenol **(4al)**

Purified by column chromatography on silica gel, eluent hexane/EtOAc (98:2), yellow solid (80 mg, 63% yield; mp 75–76 °C). ^1^H NMR (400 MHz, CDCl_3_) δ 7.46 (d, *J* = 7.6 Hz, 1H), 7.25–7.20 (m, 4H), 7.06 (d, *J* = 7.6 Hz, 4H), 7.01–6.95 (m, 8H), 6.93 (s, 2H), 6.89 (d, *J* = 8.0 Hz, 1H), 6.75 (s, 1H), 5.17 (s, 1H), 2.79–2.72 (m, 2H), 2.48–2.42 (m, 2H), 1.87–1.80 (m, 4H), 1.33 (s, 18H). ^13^C{^1^H} NMR (100 MHz, CDCl_3_) δ 153.2, 147.9, 147.0, 136.9, 136.5, 135.8, 135.3, 130.3, 129.4, 129.2, 128.2, 125.4, 124.3, 124.0, 122.9, 120.5, 118.6, 117.6, 111.4, 110.5, 61.9, 34.6, 30.5, 24.1, 23.9, 23.3, 21.4. HRMS (ESI-TOF) *m*/*z*: calcd for C_45_H_49_N_2_O [M + H]^+^, 633.3839; found, 633.3844.

#### (±)-4-((2-Bromophenyl)(1,2,3,4-tetrahydro-9*H*-carbazol-9-yl)methyl)-2,6-di-*tert*-butylphenol **(4am)**

Purified by column chromatography on silica gel, eluent hexane/EtOAc (98:2), yellow solid (82 mg, 75% yield; mp 82–83 °C). ^1^H NMR (400 MHz, CDCl_3_) δ 7.60 (dd, *J* = 7.6, 1.4 Hz, 1H), 7.47 (d, *J* = 7.7 Hz, 1H), 7.22–7.13 (m, 2H), 7.02 (t, *J* = 7.4 Hz, 1H), 6.96 (s, 1H), 6.93–6.86 (m, 2H), 6.81 (s, 2H), 6.69 (d, *J* = 8.3 Hz, 1H), 5.22 (s, 1H), 2.79–2.74 (m, 2H), 2.48–2.41 (m, 1H), 2.32–2.25 (m, 1H), 1.85–1.77 (m, 4H), 1.34–1.32 (m, 18H). ^13^C{^1^H} NMR (100 MHz, CDCl_3_) δ 153.2, 140.3, 137.0, 136.6, 135.8, 133.0, 130.1, 129.0, 128.6, 128.0, 127.6, 125.3, 124.3, 120.5, 118.5, 117.4, 111.0, 110.6, 62.6, 34.3, 30.3, 23.72, 23.68, 23.0, 21.3. HRMS (ESI-TOF) *m*/*z*: calcd for C_33_H_39_BrNO [M + H]^+^, 544.2210; found, 544.2184.

#### (±)-2,6-Di-*tert*-butyl-4-((2-hydroxyphenyl)(1,2,3,4-tetrahydro-9*H*-carbazol-9-yl)methyl)phenol **(4an)**

Purified by column chromatography on silica gel, eluent hexane/EtOAc (98:2), yellow solid (87 mg, 90% yield; mp 83–84 °C). ^1^H NMR (400 MHz, CDCl_3_) δ 7.48 (d, *J* = 7.7 Hz, 1H), 7.10–6.93 (m, 8H), 6.85 (d, *J* = 8.2 Hz, 1H), 6.78 (s, 1H), 5.21 (s, 1H), 2.76 (s, 2H), 2.44 (s, 2H), 1.88–1.80 (m, 4H), 1.34 (s, 18H). ^13^C{^1^H} NMR (100 MHz, CDCl_3_) δ 153.2, 136.69, 136.66, 136.6, 136.2, 135.8, 129.9, 129.8, 129.6, 128.0, 125.2, 120.4, 118.6, 117.5, 115.3, 115.1, 111.1, 110.6, 61.5, 34.4, 30.2, 23.9, 23.6, 23.0, 21.2. HRMS (ESI-TOF) *m*/*z*: calcd for C_33_H_40_NO_2_ [M + H]^+^, 482.3054; found, 482.3051.

#### (±)-2,6-Di-*tert*-butyl-4-((3-hydroxyphenyl)(1,2,3,4-tetrahydro-9*H*-carbazol-9-yl)methyl)phenol **(4ap)**

Purified by column chromatography on silica gel, eluent hexane/EtOAc (98:2), yellow solid (81 mg, 84% yield; mp 98–99 °C). ^1^H NMR (400 MHz, CDCl_3_) δ 7.46 (d, *J* = 7.7 Hz, 1H), 7.16 (t, *J* = 7.9 Hz, 1H), 7.01 (t, *J* = 7.3 Hz, 1H), 6.97–6.90 (m, 3H), 6.85 (d, *J* = 8.2 Hz, 1H), 6.71 (d, *J* = 9.9 Hz, 3H), 6.50 (s, 1H), 5.19 (s, 1H), 4.76 (s, 1H), 2.78–2.72 (m, 2H), 2.48–2.42 (m, 2H), 1.86–1.78 (m, 4H), 1.33 (s, 18H). ^13^C{^1^H} NMR (100 MHz, CDCl_3_) δ 155.6, 153.1, 142.8, 136.6, 136.3, 135.7, 129.6, 129.5, 128.0, 125.4, 120.8, 120.4, 118.5, 117.4, 115.0, 114.2, 111.2, 110.4, 61.9, 34.3, 30.3, 23.8, 23.6, 23.0, 21.2. HRMS (APCI-TOF) *m*/*z*: calcd for C_33_H_40_NO_2_ [M + H]^+^, 482.3054; found, 482.3045.

#### (±)-2,6-Di-*tert*-butyl-4-((3,5-dibromophenyl)(1,2,3,4-tetrahydro-9*H*-carbazol-9-yl)methyl)phenol **(4aq)**

Purified by column chromatography on silica gel, eluent hexane/EtOAc (98:2), yellow solid (102 mg, 82% yield; mp 96–97 °C). ^1^H NMR (400 MHz, CDCl_3_) δ 7.68–7.59 (m, 1H), 7.50 (d, *J* = 7.7 Hz, 1H), 7.26–7.23 (m, 2H), 7.08–6.97 (m, 2H), 6.91–6.87 (m, 3H), 6.73 (s, 1H), 5.25 (s, 1H), 2.81–2.74 (m, 2H), 2.47–2.39 (m, 2H), 1.90–1.82 (m, 4H), 1.35 (s, 18H). ^13^C{^1^H} NMR (100 MHz, CDCl_3_) δ 153.4, 145.1, 136.5, 136.0, 135.8, 133.1, 130.1, 128.22, 128.15, 125.2, 123.0, 120.8, 118.9, 117.8, 111.2, 110.8, 61.2, 34.4, 30.2, 23.9, 23.6, 23.0, 21.2. HRMS (ESI-TOF) *m*/*z*: calcd for C_33_H_37_Br_2_NO [M]^+^, 621.1236; found, 621.1220.

#### (±)-2,6-Di-*tert*-butyl-4-(naphthalen-2-yl(1,2,3,4-tetrahydro-9*H*-carbazol-9-yl)methyl)phenol **(4as)**

Purified by column chromatography on silica gel, eluent hexane/EtOAc (98:2), yellow solid (76 mg, 74% yield; mp 101–102 °C). ^1^H NMR (400 MHz, CDCl_3_) δ 7.87–7.82 (m, 1H), 7.78 (d, *J* = 8.6 Hz, 1H), 7.76–7.71 (m, 1H), 7.54–7.44 (m, 4H), 7.33 (dd, *J* = 8.6, 1.4 Hz, 1H), 7.07–7.02 (m, 3H), 7.00–6.93 (m, 3H), 5.24 (s, 1H), 2.84–2.77 (m, 2H), 2.56–2.41 (m, 2H), 1.88–1.81 (m, 4H), 1.36 (s, 18H). ^13^C{^1^H} NMR (100 MHz, CDCl_3_) δ 153.2, 138.5, 136.8, 136.4, 135.8, 133.2, 132.7, 129.6, 128.2, 128.05, 128.01, 127.6, 126.9, 126.6, 126.1, 126.0, 125.4, 120.4, 118.6, 117.5, 111.1, 110.5, 62.3, 34.4, 30.3, 24.0, 23.7, 23.0, 21.3. HRMS (ESI-TOF) *m*/*z*: calcd for C_37_H_42_NO [M + H]^+^, 516.3261; found, 516.3244.

#### (±)-2,6-Di-*tert*-butyl-4-(pyren-1-yl(1,2,3,4-tetrahydro-9*H*-carbazol-9-yl)methyl)phenol **(4au)**

Purified by column chromatography on silica gel, eluent hexane/EtOAc (98:2), orange solid (94 mg, 80% yield; mp 135–136 °C). ^1^H NMR (400 MHz, CDCl_3_) δ 8.20 (d, *J* = 7.5 Hz, 1H), 8.16 (d, *J* = 7.5 Hz, 1H), 8.11–7.98 (m, 6H), 7.75 (s, 1H), 7.50 (d, *J* = 7.7 Hz, 1H), 7.46 (d, *J* = 8.0 Hz, 1H), 7.06–7.01 (m, 1H), 6.98–6.87 (m, 4H), 5.21 (s, 1H), 2.79–2.73 (m, 2H), 2.31–2.23 (m, 1H), 2.12–2.05 (m, 1H), 1.76–1.66 (m, 4H), 1.28 (s, 18H). ^13^C{^1^H} NMR (100 MHz, CDCl_3_) δ 153.2, 137.2, 136.7, 135.9, 134.3, 131.4, 130.9, 130.7, 130.3, 129.1, 128.2, 128.0, 127.5, 127.4, 126.6, 126.0, 125.5, 125.4, 125.3, 124.82, 124.75, 124.73, 123.1, 120.7, 118.6, 117.5, 110.8, 110.6, 60.5, 34.4, 30.3, 24.2, 23.7, 22.9, 21.3. HRMS (APCI-TOF) *m*/*z*: calcd for C_43_H_45_NO [M]^+^, 591.3496; found, 591.3484.

#### (±)-4,4′-(1,4-Phenylenebis((1,2,3,4-tetrahydro-9*H*-carbazol-9-yl)methylene))bis(2,6-di-*tert*-butylphenol) **(4av)**

Purified by column chromatography on silica gel, eluent hexane/EtOAc (98:2), yellow solid (146 mg, 82% yield; mp 76–77 °C). ^1^H NMR (400 MHz, CDCl_3_) δ 7.52–7.46 (m, 2H), 7.09–7.02 (m, 6H), 6.99–6.93 (m, 6H), 6.91–6.87 (m, 2H), 6.81 (s, 2H), 5.22 (s, 1H), 5.20 (s, 1H), 2.81–2.75 (m, 4H), 2.49–2.43 (m, 4H), 1.88–1.81 (m, 8H), 1.37 (s, 18H), 1.35 (s, 18H). ^13^C{^1^H} NMR (100 MHz, CDCl_3_) δ 153.1, 140.0, 136.7, 136.30, 136.28, 135.73, 129.70, 129.7, 128.2, 128.0, 125.4, 125.3, 120.38, 120.35, 118.5, 117.5, 111.2, 111.1, 110.44, 110.40, 62.0, 61.9, 34.4, 30.3, 24.0, 23.7, 23.1, 23.0, 21.3 (Due to a mixture of diastereomer, all ^13^C{^1^H} NMR signals could not be resolved). HRMS (ESI-TOF) *m*/*z*: calcd for C_60_H_73_N_2_O_2_ [M + H]^+^, 853.5667; found, 853.5646.

#### (±)-4-((4-Bromophenyl)(2,3-dihydrocyclopenta[*b*]indol-4(1*H*)-yl)methyl)-2,6-di-*tert*-butylphenol **(4bd)**

Purified by column chromatography on silica gel, eluent hexane/EtOAc (98:2), yellow solid (85 mg, 80% yield; mp 96–97 °C). ^1^H NMR (400 MHz, CDCl_3_) δ 7.49–7.41 (m, 3H), 7.24–7.19 (m, 1H), 6.97–6.92, 7.11–7.04 (m, 2H), (m, 4H), 6.75 (s, 1H), 5.26 (s, 1H), 2.80 (t, *J* = 6.8 Hz, 2H), 2.39–2.30 (m, 2H), 2.24–2.16 (m, 1H), 2.03–1.95 (m, 1H), 1.37 (s, 18H). ^13^C{^1^H} NMR (100 MHz, CDCl_3_) δ 153.5, 145.7, 141.4, 140.1, 135.9, 131.5, 129.7, 129.5, 125.8, 124.6, 121.3, 120.2, 119.5, 119.3, 118.4, 110.4, 62.5, 34.4, 30.3, 28.7, 27.3, 24.0. HRMS (ESI-TOF) *m*/*z*: calcd for C_32_H_36_BrNO [M]^+^, 529.1975; found, 529.1953.

#### (±)-4-((4-Bromophenyl)(7,8,9,10-tetrahydrocyclohepta[*b*]indol-5(6*H*)-yl)methyl)-2,6-di-*tert*-butylphenol **(4cd)**

Purified by column chromatography on silica gel, eluent hexane/EtOAc (98:2), yellow solid (78 mg, 70% yield; mp 90–91 °C). ^1^H NMR (400 MHz, CDCl_3_) δ 7.51 (d, *J* = 7.8 Hz, 1H), 7.44–7.39 (m, AA’ part of AA’BB’ system, 2H), 7.08–7.03 (m, 1H), 7.02–6.99 (m, BB’ part of AA’BB’ system, 2H), 6.98–6.93 (m, 4H), 6.90 (s, 1H), 5.22 (s, 1H), 2.91–2.80 (m, 2H), 2.72–2.57 (m, 2H), 1.85–1.71 (m, 4H), 1.51–1.46 (m, 2H), 1.35 (s, 18H). ^13^C{^1^H} NMR (100 MHz, CDCl_3_) δ 153.1, 140.1, 139.8, 136.1, 135.9, 131.4, 129.8, 129.3, 128.4, 125.0, 121.1, 120.2, 118.7, 117.4, 115.6, 110.6, 61.4, 34.4, 31.6, 30.3, 28.2, 28.1, 26.8, 24.2. HRMS (ESI-TOF) *m*/*z*: calcd for C_34_H_40_BrNO [M]^+^, 557.2288; found, 557.2278.

#### (±)-4-((4-Bromophenyl)(6,7,8,9,10,11-hexahydro-5*H*-cycloocta[*b*]indol-5-yl)methyl)-2,6-di-*tert*-butylphenol **(4dd)**

Purified by column chromatography on silica gel, eluent hexane/EtOAc (98:2), light yellow solid (87 mg, 76% yield; mp 93–94 °C). ^1^H NMR (400 MHz, CDCl_3_) δ 7.52 (d, *J* = 7.8 Hz, 1H), 7.43–7.38 (m, 2H), 7.04–6.98 (m, 3H), 6.95 (s, 2H), 6.88 (t, *J* = 7.6 Hz, 1H), 6.79 (s, 1H), 6.75 (d, *J* = 8.3 Hz, 1H), 5.20 (s, 1H), 2.90–2.82 (m, 4H), 1.73–1.67 (m, 2H), 1.47–1.34 (m, 6H), 1.31 (s, 18H). ^13^C{^1^H} NMR (100 MHz, CDCl_3_) δ 153.2, 139.7, 137.2, 136.2, 135.9, 132.9, 131.4, 129.8, 129.5, 125.2, 121.1, 120.2, 118.5, 117.5, 113.3, 111.7, 61.5, 34.3, 30.7, 30.2, 29.1, 26.4, 25.7, 23.7, 23.2. HRMS (ESI-TOF) *m*/*z*: calcd for C_35_H_42_BrNO [M]^+^, 571.2444; found, 571.2440.

#### (±)-4-((4-Bromophenyl)(2,3-dimethyl-1*H*-indol-1-yl)methyl)-2,6-di-*tert*-butylphenol **(4ed)**

Purified by column chromatography on silica gel, eluent hexane/EtOAc (98:2), yellow solid (81 mg, 78% yield; mp 87–88 °C). ^1^H NMR (400 MHz, CDCl_3_) δ 7.50 (d, *J* = 7.8 Hz, 1H), 7.45–7.39 (m, 2H), 7.05–6.97 (m, 5H), 6.94–6.89 (m, 1H), 6.78–6.72 (m, 2H), 5.23 (s, 1H), 2.28 (s, 3H), 2.24 (s, 3H), 1.34 (s, 18H). ^13^C{^1^H} NMR (100 MHz, CDCl_3_) δ 153.2, 140.0, 136.2, 135.9 (2C), 133.0, 131.5, 129.9, 129.3, 125.3, 121.2, 120.4, 118.6, 117.8, 111.4, 107.9, 62.1, 34.4, 30.2, 11.6, 9.0. HRMS (ESI-TOF) *m*/*z*: calcd for C_31_H_36_BrNO [M]^+^, 517.1975; found, 517.1966.

#### (±)-4-((4-Bromophenyl)(2-ethyl-3-methyl-1*H*-indol-1-yl)methyl)-2,6-di-*tert*-butylphenol **(4fd)**

Purified by column chromatography on silica gel, eluent hexane/EtOAc (98:2), yellow solid (77 mg, 72% yield; mp 78–79 °C). ^1^H NMR (400 MHz, CDCl_3_) δ 7.49 (d, *J* = 7.9 Hz, 1H), 7.43–7.38 (m, 2H), 7.04–7.00 (m, 5H), 6.90–6.84 (m, 1H), 6.73 (s, 1H), 6.68 (d, *J* = 8.3 Hz, 1H), 5.20 (s, 1H), 2.76 (qd, *J* = 7.5, 1.9 Hz, 2H), 2.29 (s, 3H), 1.32 (s, 18H), 1.04 (t, *J* = 7.5 Hz, 3H). ^13^C{^1^H} NMR (100 MHz, CDCl_3_) δ 153.2, 139.9, 139.0, 136.0, 135.9, 131.4, 129.8, 129.44, 129.38, 125.2, 121.2, 120.4, 118.6, 117.8, 112.0, 107.2, 61.8, 34.3, 30.2, 18.6, 14.5, 8.8. HRMS (ESI-TOF) *m*/*z*: calcd for C_32_H_38_BrNO [M]^+^, 531.2131; found, 531.2120.

#### (±)-4-((4-Bromophenyl)(6-methyl-1,2,3,4-tetrahydro-9*H*-carbazol-9-yl)methyl)-2,6-di-*tert*-butylphenol **(4hd)**

Purified by column chromatography on silica gel, eluent hexane/EtOAc (98:2), yellow solid (68 mg, 61% yield; mp 89–90 °C). ^1^H NMR (400 MHz, CDCl_3_) δ 7.44–7.38 (m, 2H), 7.27 (s, 1H), 6.98–6.94 (m, 4H), 6.80–6.69 (m, 3H), 5.22 (s, 1H), 2.77–2.71 (m, 2H), 2.45–2.40 (m, 5H), 1.86–1.79 (m, 4H), 1.35 (s, 18H). ^13^C{^1^H} NMR (100 MHz, CDCl_3_) δ 153.2, 140.1, 136.2, 135.8, 134.9, 131.4, 129.9, 129.4, 128.3, 127.8, 125.4, 122.0, 121.1, 117.4, 110.7, 110.2, 61.6, 34.4, 30.3, 23.9, 23.6, 23.0, 21.4, 21.2. HRMS (ESI-TOF) *m*/*z*: calcd for C_34_H_40_BrNO [M]^+^, 552.2288; found, 552.2267.

#### (±)-4-((4-Bromophenyl)(6-methoxy-1,2,3,4-tetrahydro-9*H*-carbazol-9-yl)methyl)-2,6-di-*tert*-butylphenol **(4id)**

Purified by column chromatography on silica gel, eluent hexane/EtOAc (98:2), yellow solid (73 mg, 64% yield; mp 103–104 °C). ^1^H NMR (400 MHz, CDCl_3_) δ 7.43–7.39 (m, 2H), 6.98–6.95 (m, 2H), 6.95–6.93 (m, 3H), 6.70–6.66 (m, 2H), 6.59 (dd, *J* = 8.8, 2.5 Hz, 1H), 5.23 (s, 1H), 3.84 (s, 3H), 2.74–2.70 (m, 2H), 2.48–2.43 (m, 2H), 1.87–1.81 (m, 4H), 1.34 (s, 18H). ^13^C{^1^H} NMR (100 MHz, CDCl_3_) δ 153.5, 153.2, 140.1, 136.9, 135.9, 131.7, 131.4, 129.9, 129.3, 128.4, 125.3, 121.2, 111.9, 110.3, 109.9, 100.0, 61.7, 55.8, 34.4, 30.3, 23.8, 23.6, 23.0, 21.3. HRMS (ESI-TOF) *m*/*z*: calcd for C_34_H_40_BrNO_2_ [M]^+^, 573.2237; found, 573.2225.

#### (±)-4-((4-Bromophenyl)(6-chloro-1,2,3,4-tetrahydro-9*H*-carbazol-9-yl)methyl)-2,6-di-*tert*-butylphenol **(4jd)**

Purified by column chromatography on silica gel, eluent hexane/EtOAc (98:2), yellow solid (46 mg, 40% yield; mp 79–80 °C). ^1^H NMR (400 MHz, CDCl_3_) δ 7.43–7.38 (m, 3H), 6.95–6.91 (m, 2H), 6.89 (s, 2H), 6.87–6.83 (m, 1H), 6.67–6.61 (m, 2H), 5.23 (s, 1H), 2.71–2.66 (m, 2H), 2.47–2.42 (m, 2H), 1.86–1.78 (m, 4H), 1.32 (s, 18H). ^13^C{^1^H} NMR (100 MHz, CDCl_3_) δ 153.3, 139.6, 137.7, 136.0, 134.8, 131.5, 129.9, 129.2, 128.8, 125.1, 124.4, 121.4, 120.5, 117.2, 112.1, 110.4, 61.8, 34.3, 30.2, 23.6, 23.4, 22.8, 21.0. HRMS (ESI-TOF) *m*/*z*: calcd for C_33_H_37_BrClNO [M]^+^, 577.1742; found, 577.1746.

### Synthetic Applications

#### (±)-4-((4-Bromophenyl)(2,3,4,9-tetrahydro-1*H*-carbazol-7-yl)methyl)phenol **(5)**

To a solution of the compound **3ad** (109 mg, 0.2 mmol) in toluene (0.02 M) at rt was added a solution of AlCl_3_ (240 mg, 1.8 mmol) in MeNO_2_ (2.25 M) in one portion. The mixture was immediately heated to 60 °C in a preheated oil bath and maintained at this temperature for 10 min. The mixture was subsequently cooled and poured into a separatory funnel containing ice and Et_2_O (1:1). The layers were separated, and the aqueous layer was extracted with Et_2_O (3 × 20 mL). The combined organic layers were dried over Na_2_SO_4_, filtered, and concentrated in vacuo. The crude product was purified by column chromatography using hexane/EtOAc (90:10) to give product **5**. Broken white solid (67 mg, 78% yield; mp 122–123 °C). ^1^H NMR (400 MHz, CDCl_3_) δ 7.54 (bs, 1H), 7.42–7.34 (m, 3H), 7.03–6.94 (m, 4H),6.88 (s, 1H), 6.85 (dd, *J* = 8.1, 1.3 Hz, 1H), 6.75–6.69 (m, BB’ part of AA’BB’ system, 2H), 5.54 (s, 1H), 4.83 (bs, 1H), 2.74–2.63 (m, 4H), 2.00–1.76 (m, 4H). ^13^C{^1^H} NMR (100 MHz, CDCl_3_) δ 154.1, 144.4, 136.9, 136.6, 135.9, 134.5, 131.4, 131.3, 130.7, 126.4, 121.2, 120.0, 117.7, 115.3, 111.3, 110.1, 55.7, 23.5, 23.4, 23.3, 21.1. HRMS (ESI-TOF) *m*/*z*: calcd for C_25_H_22_BrNO [M]^+^, 431.0879; found, 431.0864.

#### (±)-2,6-Di-*tert*-butyl-4-((2,3,4,9-tetrahydro-1*H*-carbazol-7-yl)(4-(thiophene-2-yl)phenyl)methyl)phenol **(7)**

Compound **3ad** (109 mg, 0.20 mmol) and thiophene-2-boronic acid (**6**) (31 mg, 0.24 mmol) were dissolved in 1,2-dimethoxyethane/water (15 mL, 2:1 v/v), and Na_2_CO_3_ (42 mg, 0.40 mmol) was added. After degassing, Pd(PPh_3_)_4_ (5 mg, 4 μmol) was added, and the mixture was boiled at 100 °C for 18 h. Then the mixture was cooled to room temperature and the solution was extracted with DCM (3 × 10 mL). The organic phases were combined, washed with water (3 × 10 mL), dried over Na_2_SO_4_, and concentrated under reduced pressure. The crude material was purified via silica gel column chromatography using hexane/EtOAc (95:5) to give the compound **7**. Orange solid (93 mg, 85% yield; mp 129–130 °C). ^1^H NMR (400 MHz, CDCl_3_) δ 7.55 (bs, 1H), 7.52–7.48 (m, AA’ part of AA’BB’ system, 2H), 7.36 (d, *J* = 8.1 Hz, 1H), 7.27 (dd, *J* = 3.6, 1.0 Hz, 1H), 7.24 (dd, *J* = 5.2, 1.0 Hz, 1H), 7.18–7.10 (m, BB’ part of AA’BB’ system, 2H) 7.06 (dd, *J* = 5.2, 3.6 Hz, 1H), 7.00 (s, 2H), 6.95 (s, 1H), 6.91 (dd, *J* = 8.1, 1.3 Hz, 1H), 5.55 (s, 1H), 5.07 (s, 1H), 2.70 (t, *J* = 5.7 Hz, 4H), 1.95–1.82 (m, 4H), 1.37 (s, 18H). ^13^C{^1^H} NMR (100 MHz, CDCl_3_) δ 152.2, 145.4, 144.8, 138.1, 135.9, 135.6, 134.8, 134.1, 132.0, 130.1, 128.1, 126.24, 126.22, 125.7, 124.4, 122.8, 121.5, 117.5, 111.4, 110.1, 56.9, 34.5, 30.5, 23.5, 23.4, 23.3, 21.1. HRMS (ESI-TOF) *m*/*z*: calcd for C_37_H_41_NOS [M]^+^, 547.2903; found, 547.2878.

#### (±)-2-(6-((4-Bromophenyl)(3,5-di-*tert*-butyl-4-hydroxyphenyl)methyl)-1-(4-chlorobenzoyl)-5-methoxy-2-methyl-1*H*-indol-3-yl)acetic Acid **(8d)**

Compound **8d** was synthesized by following general procedure A. Purified by column chromatography on silica gel, eluent DCM/MeOH (99:1), yellow solid (120 mg, 82% yield; mp 199–200 °C). ^1^H NMR (400 MHz, CDCl_3_) δ 7.56–7.48 (m, AA’ part of AA’BB’ system, 2H), 7.35–7.30 (m, BB’ part of AA’BB’ system, 2H), 7.30–7.27 (m, AA’ part of AA’BB’ system, 2H), 6.90 (s, 1H), 6.77–6.72 (m, BB’ part of AA’BB’ system, 2H), 6.70 (s, 2H), 6.32 (s, 1H), 5.69 (s, 1H), 5.02 (s, 1H), 3.72 (s, 3H), 3.70 (s, 2H), 2.43 (s, 3H), 1.32 (s, 18H). ^13^C{^1^H} NMR (100 MHz, CDCl_3_) δ 176.5, 168.3, 154.0, 152.1, 143.9, 139.4, 136.2, 135.5, 133.7, 133.3, 131.1, 131.04, 131.00, 130.5, 130.1, 129.1, 128.6, 125.8, 119.7, 116.1, 111.7, 99.8, 56.3, 49.4, 34.4, 30.5, 30.2, 13.2. HRMS (ESI-TOF) *m*/*z*: calcd for C_40_H_42_BrClNO_5_ [M + H]^+^, 730.1929; found, 730.1915.

#### (±)-2-(1-(4-Chlorobenzoyl)-6-((3,5-di-*tert*-butyl-4-hydroxyphenyl)(*p*-tolyl)methyl)-5-methoxy-2-methyl-1*H*-indol-3-yl)acetic Acid **(8f)**

Compound **8f** was synthesized by following general procedure A. Purified by column chromatography on silica gel, eluent DCM/MeOH (99:1), white solid (117 mg, 88% yield; mp 128–129 °C). ^1^H NMR (400 MHz, CDCl_3_) δ 7.58–7.50 (m, AA’ part of AA’BB’ system, 2H), 7.32–7.27 (m, BB’ part of AA’BB’ system, 2H), 7.03–6.95 (m, AA’ part of AA’BB’ system, 2H), 6.92 (s, 1H), 6.80–6.76 (m, BB’ part of AA’BB’ system, 2H), 6.75 (s, 2H), 6.39 (s, 1H), 5.75 (s, 1H), 5.00 (bs, 1H), 3.74 (s, 3H), 3.70 (s, 2H), 2.46 (s, 3H), 2.34 (s, 3H), 1.34 (s, 18H).^13^C{^1^H} NMR (100 MHz, CDCl_3_) δ 177.3, 168.4, 154.1, 151.9, 141.5, 139.3, 136.0, 135.2, 135.0, 134.2, 133.6, 131.2, 131.1, 130.5, 129.2, 129.0, 128.7, 128.4, 125.8, 116.2, 111.8, 99.8, 56.4, 49.4, 34.4, 30.5, 30.3, 21.2, 13.1. HRMS (ESI-TOF) *m*/*z*: calcd for C_41_H_45_ClNO_5_ [M + H]^+^, 666.2981; found, 666.2972.

#### (±)-2-(1-(4-Chlorobenzoyl)-6-((3,5-di-*tert*-butyl-4-hydroxyphenyl)(4 (trifluoromethyl)phenyl)methyl)-5-methoxy-2-methyl-1*H*-indol-3-yl)acetic Acid **(8k)**

Compound **8k** was synthesized by following general procedure A. Purified by column chromatography on silica gel, eluent DCM/MeOH (99:1), broken white solid (128 mg, 89% yield; mp 179–180 °C). ^1^H NMR (400 MHz, CDCl_3_) δ 7.56–7.49 (m, AA’ part of AA’BB’ system, 2H), 7.47–7.41 (m, AA’ part of AA’BB’ system, 2H), 7.34–7.28 (m, BB’ part of AA’BB’ system, 2H), 7.04–6.97 (m, BB’ part of AA’BB’ system, 2H), 6.92 (s, 1H), 6.73 (s, 2H), 6.35 (s, 1H), 5.80 (s, 1H), 5.05 (s, 1H), 3.73 (s, 3H), 3.71 (s, 2H), 2.44 (s, 3H), 1.34 (s, 18H). ^13^C{^1^H} NMR (100 MHz, CDCl_3_) δ 177.1, 168.2, 153.9, 152.1, 148.9, 139.3, 136.2, 135.5, 133.6, 132.9, 131.0, 130.3, 129.6, 129.4, 129.0, 128.7, 127.9 (q, *J* = 32.1 Hz), 125.7, 124.8 (q, *J* = 3.4 Hz). 124.4 (q, *J* = 271.5 Hz), 116.0, 111.6, 99.7, 56.1, 49.8, 34.3, 30.4, 30.1, 13.1. HRMS (ESI-TOF) *m*/*z*: calcd for C_41_H_42_ClF_3_NO_5_ [M + H]^+^, 720.2698; found, 720.2691.

### Control Experiment

#### (±)-2-(Phenyl(2,3,4,9-tetrahydro-1*H*-carbazol-7-yl)methyl)phenol **(10)**(26)

To a solution of indole (**1a)** (34.2 mg, 0.20 mmol) and 2-(hydroxy(phenyl)methyl)phenol (**9)** (44.0 mg, 0.22 mmol) in toluene (0.1 M), In(OTf)_3_ (11.2 mg, 0.02 mmol, 10 mol %) was added, and the mixture was stirred at room temperature for 12h. After the reaction was complete (monitored by TLC), the solvent was removed under reduced pressure and the residue was purified by silica gel column chromatography using hexane/EtOAc (80:20) solvent mixture to give the compound **10**. White solid (63 mg, 89% yield; mp 74–75 °C). ^1^H NMR (400 MHz, CDCl_3_) δ 7.53 (bs, 1H), 7.43 (d, *J* = 8.5 Hz, 1H), 7.38–7.26 (m, 3H), 7.21–7.13 (m, 3H), 6.98–6.93 (m, 2H), 6.90–6.81 (m, 3H), 5.80 (s, 1H), 4.95 (bs, 1H), 2.77–2.62 (m, 4H), 1.97–1.84 (m, 4H). ^13^C{^1^H} NMR (100 MHz, CDCl_3_) δ 153.8, 143.2, 135.9, 135.0, 134.7, 131.2, 130.6, 129.6, 128.6, 127.9, 126.8, 126.6, 121.1, 120.7, 118.1, 116.4, 111.2, 110.1, 51.5, 23.35, 23.33, 23.2, 21.0.

## Data Availability

The data underlying this study are available in the published article and its online Supporting Information.

## Supporting Information Available

^1^H NMR, ^13^C NMR and HRMS spectra for all compounds; computational details (PDF)
