# Supplementary material for: Solvent-Mediated Tunable Regiodivergent C6- and N1-Alkylations of 2,3-Disubstituted Indoles with p-Quinone Methides
Source: J Org Chem. 2023 Feb 13;88(5):3132–47. doi: 10.1021/acs.joc.2c02937 (PMC9990074; doi:10.1021/acs.joc.2c02937)
Supplement: Supplementary file 1 — jo2c02937_si_001.pdf [file jo2c02937_si_001.pdf]

Supporting Information

Solvent-Mediated Tunable Regiodivergent C6- and N1-Alkylations of 2,3-Disubstituted Indoles with *p*-Quinone Methides

Douaa Adris,[a] Yunus Taskesenligil,[a] Volkan Akyildiz,[a] Selcuk Essiz,[b] and Nurullah Saracoglu[a,*]

[a]Department of Chemistry, Faculty of Sciences, Atatürk University, Erzurum 25240, Türkiye

[b]Department of Medical Services and Techniques, Vocational School of Health Services, Hakkari University, Hakkari, 30000, Türkiye

*E-mail: nsarac@atauni.edu.tr

Table of Contents

1. NOE Experiment for **3ad** **S2**
2. ^{1}H NMR (400 MHz) and $^{13}C\{^{1}H\}$ NMR (100 MHz) Spectra of Compounds **S3**
3. HRMS Spectra of Compounds **S67**
4. Computational Details **S88**

1. NOE Experiment for 3ad

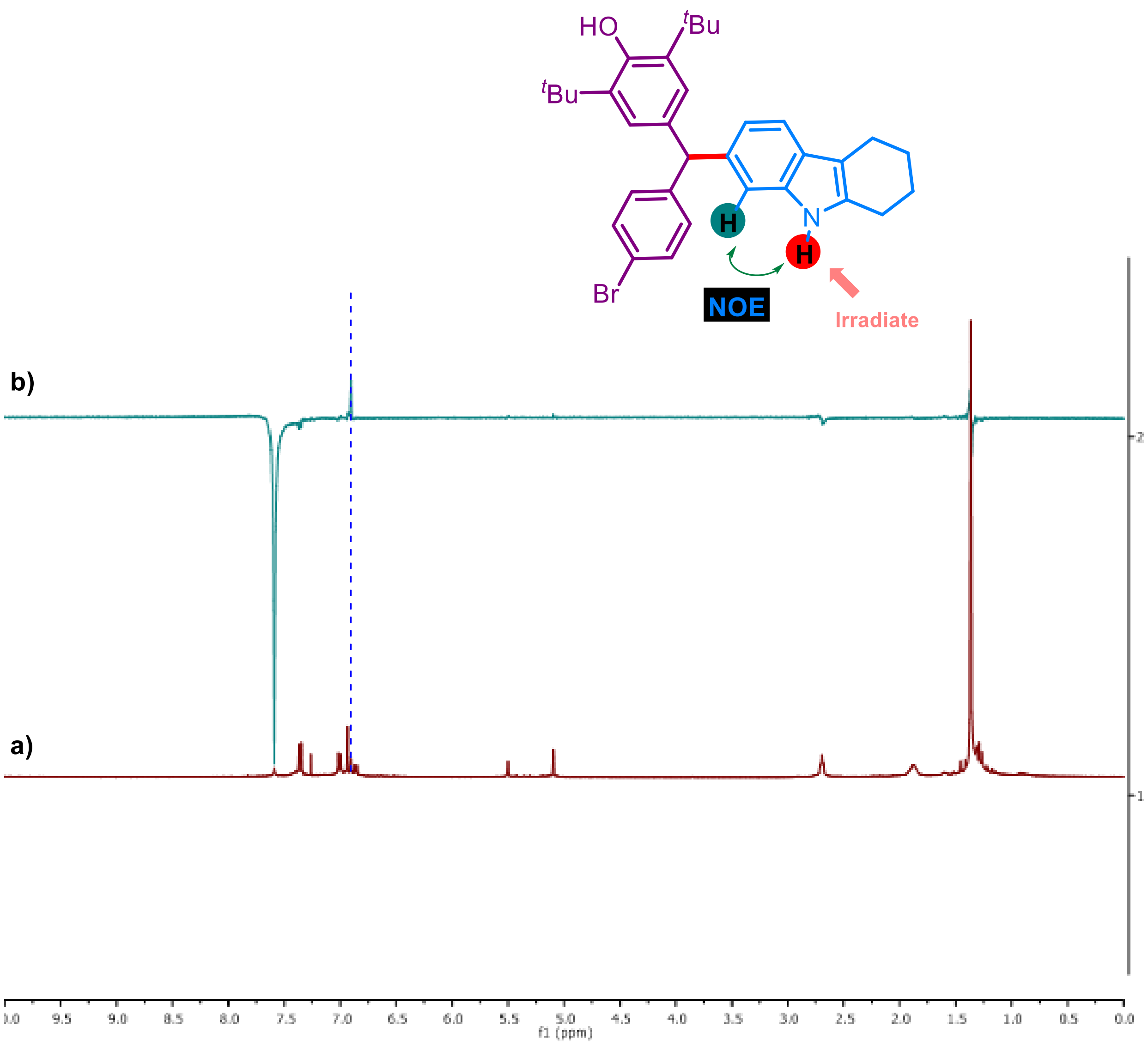

^{1}H NMR spectrum (a) and ^{1}H NOE NMR spectrum (b) of 4-((4-bromophenyl)(2,3,4,9-tetrahydro-1*H*-carbazol-7-yl)methyl)-2,6-di-*tert*-butylphenol (**3ad**) (400 MHz, $CDCl_3$).

There is NOE correlation between NH and C7-H, which is assigned to be **3ad**.

2. ^{1}H NMR (400 MHz) and $^{13}C\{^{1}H\}$ NMR (100 MHz) Spectra of Compounds

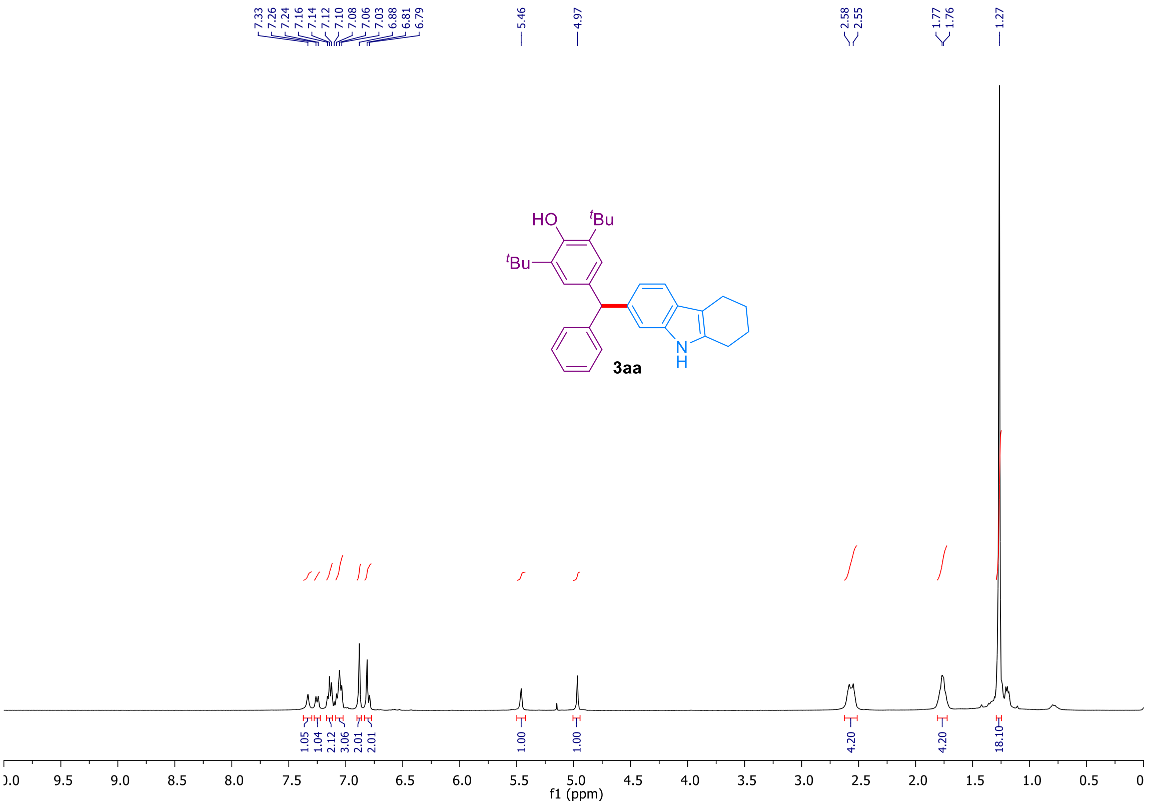

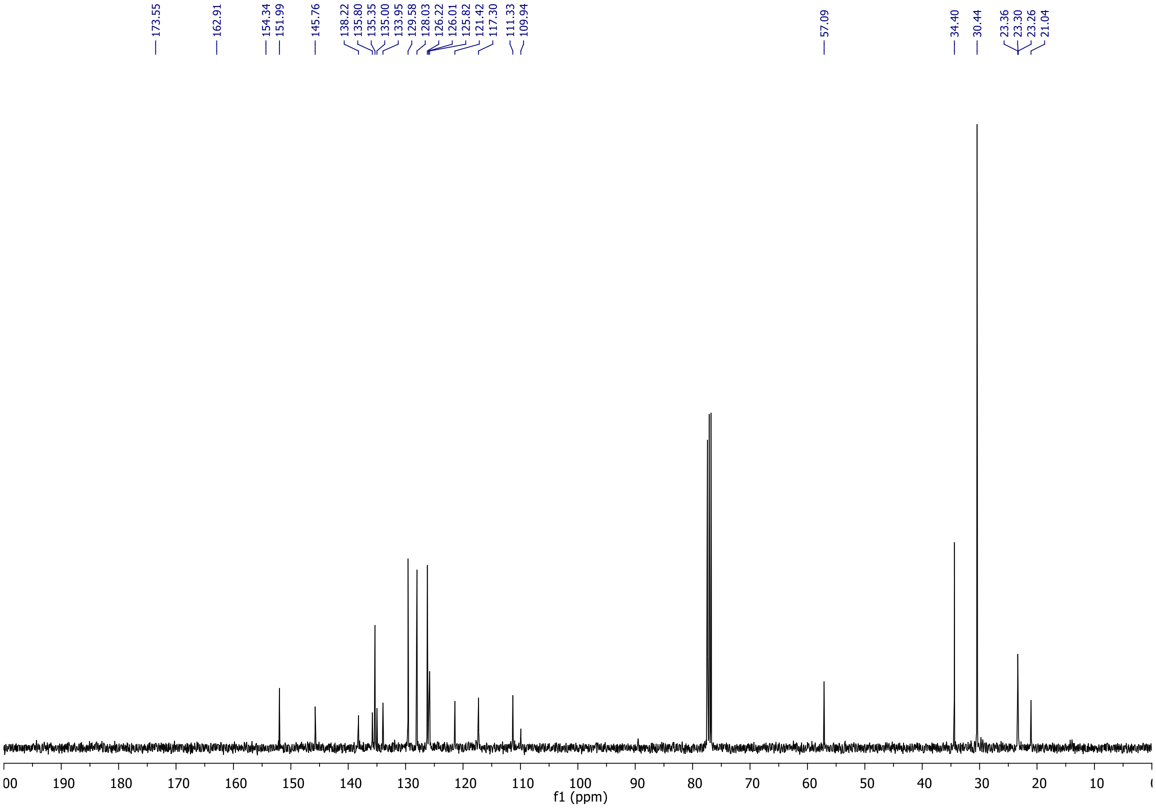

^{1}H NMR (400 MHz) and $^{13}C\{^{1}H\}$ NMR (100 MHz) spectra of **3aa** ($CDCl_3$)

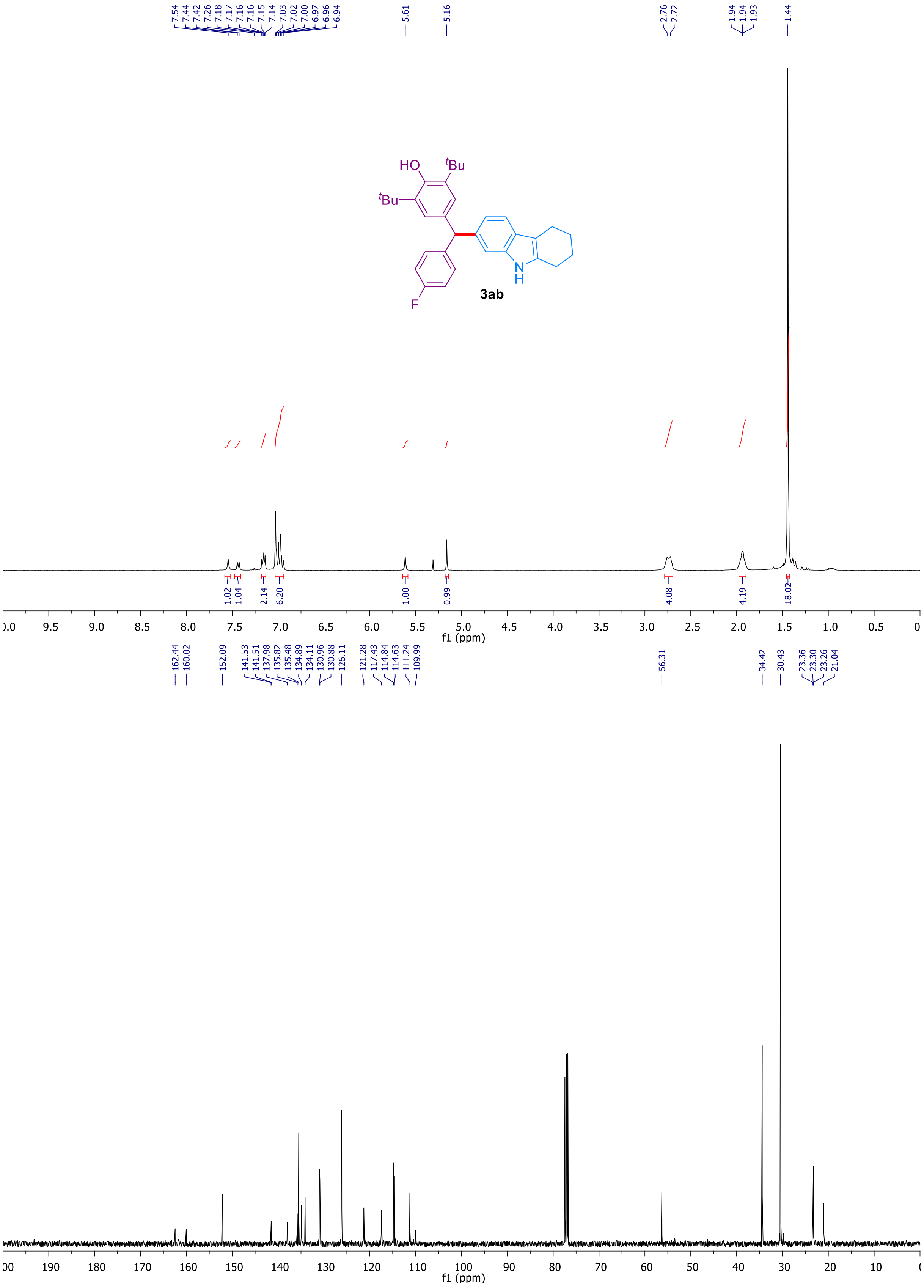

^{1}H NMR (400 MHz) and $^{13}C\{^{1}H\}$ NMR (100 MHz) spectra of **3ab** ($CDCl_3$)

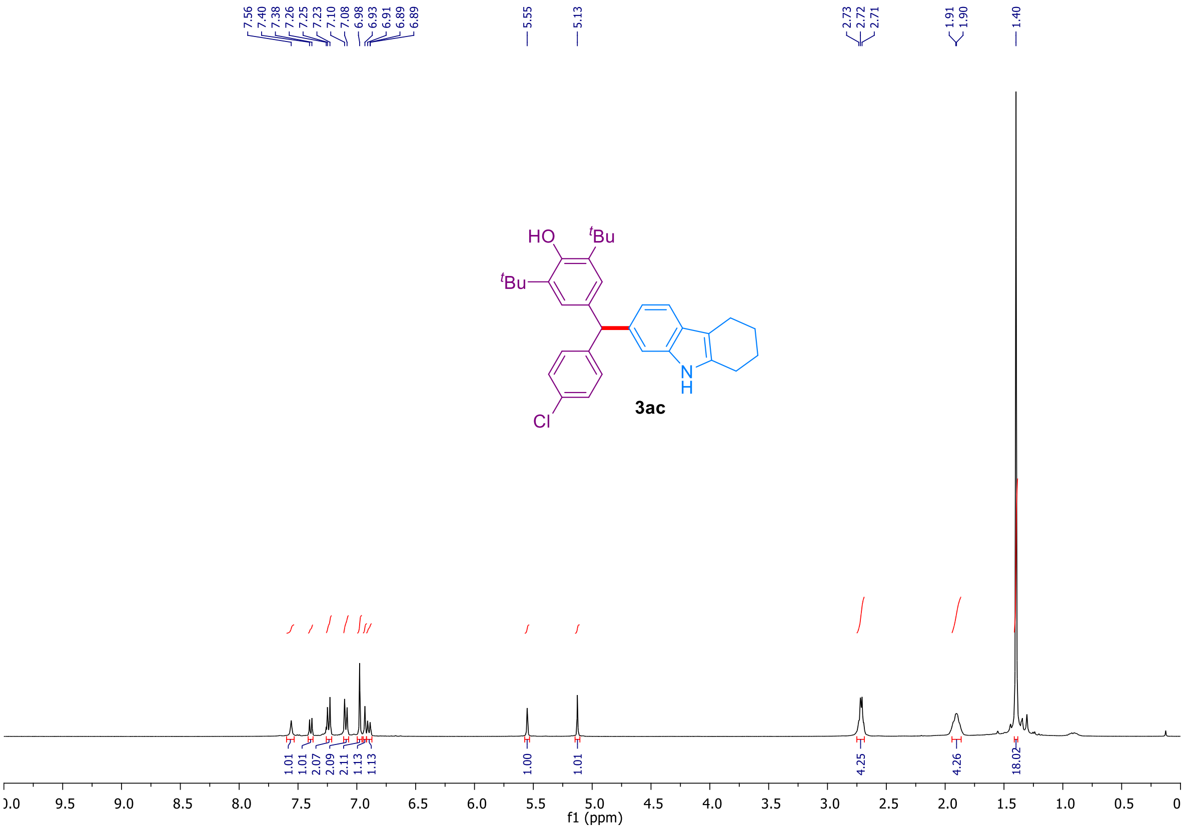

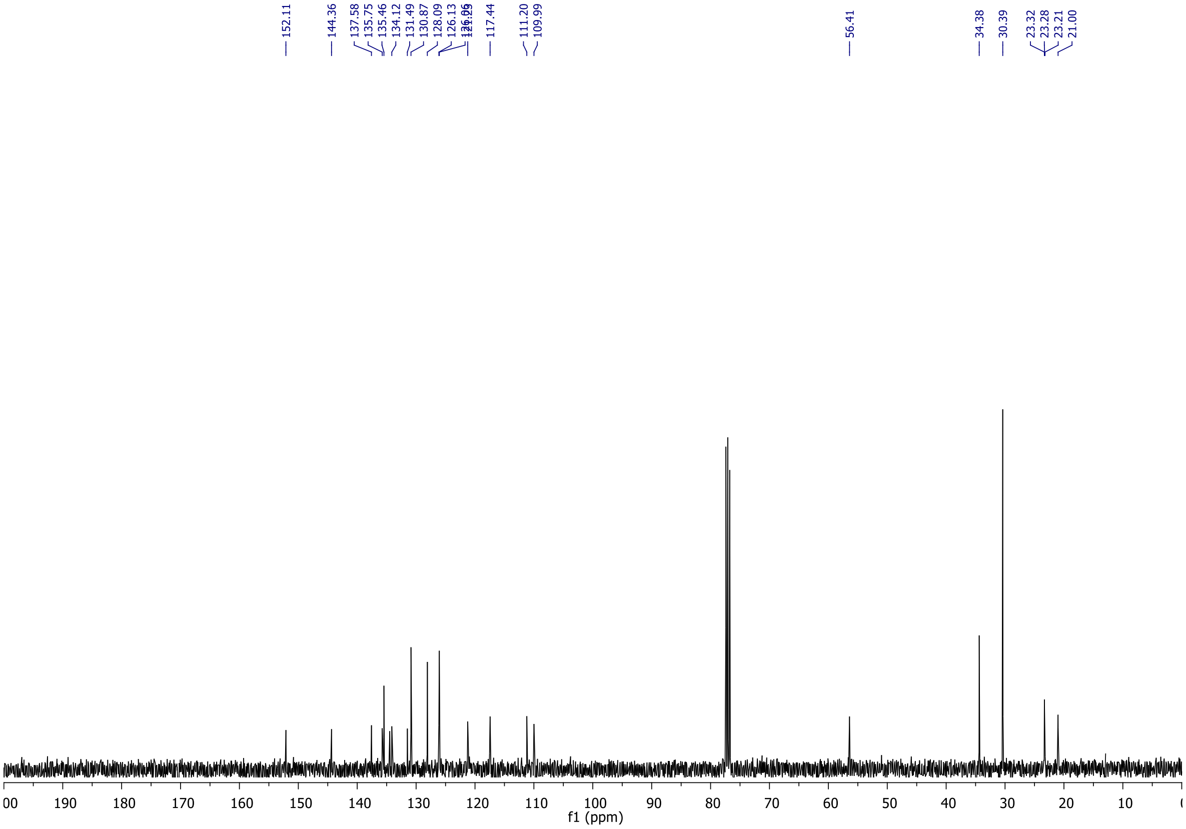

^{1}H NMR (400 MHz) and $^{13}C\{^{1}H\}$ NMR (100 MHz) spectra of **3ac** ($CDCl_3$)

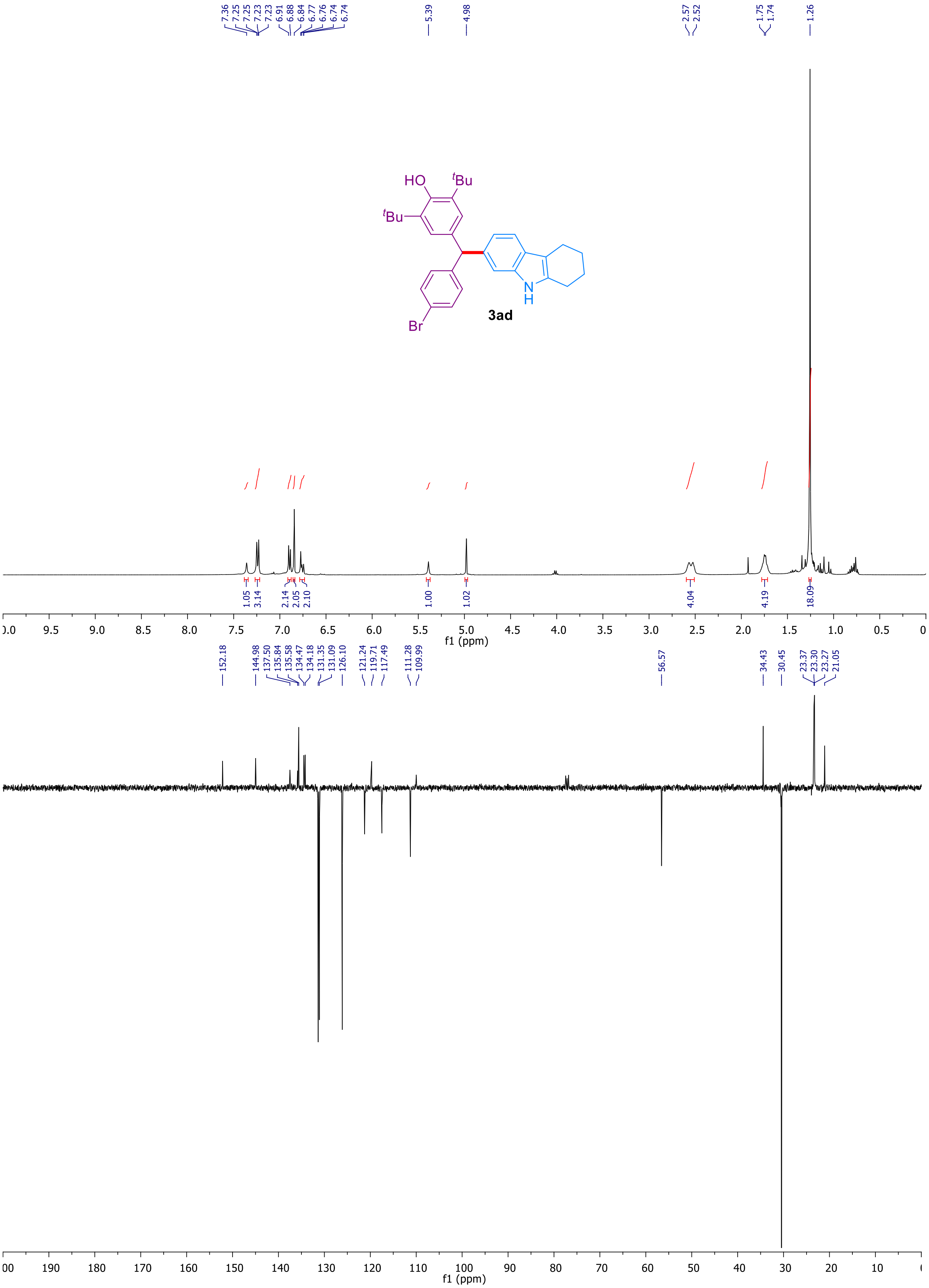

^{1}H NMR (400 MHz) and ^{13}C{^{1}H} NMR (100 MHz) spectra of **3ad** ($CDCl_3$)

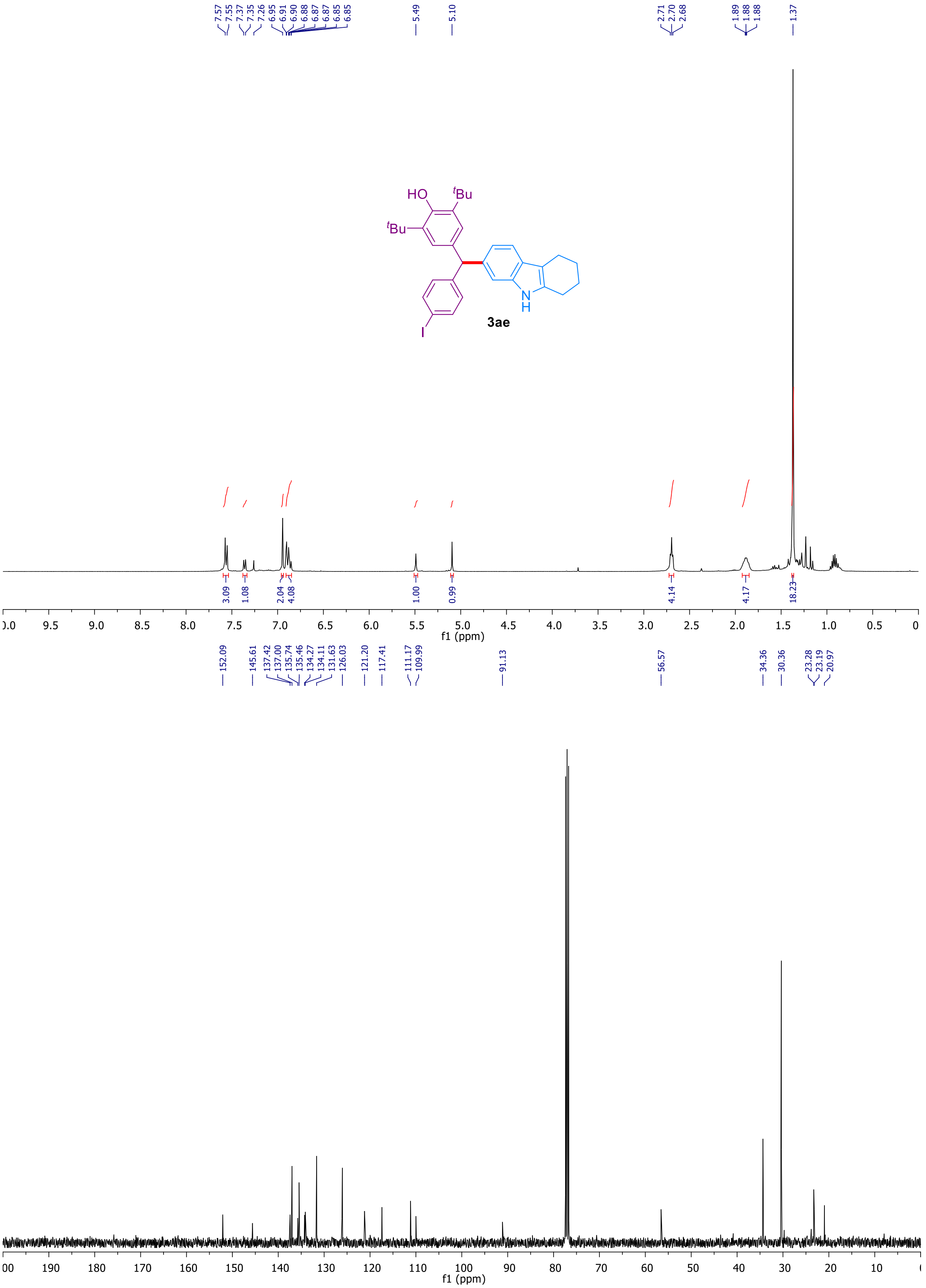

^{1}H NMR (400 MHz) and ^{13}C{^{1}H} NMR (100 MHz) spectra of **3ae** ($CDCl_3$)

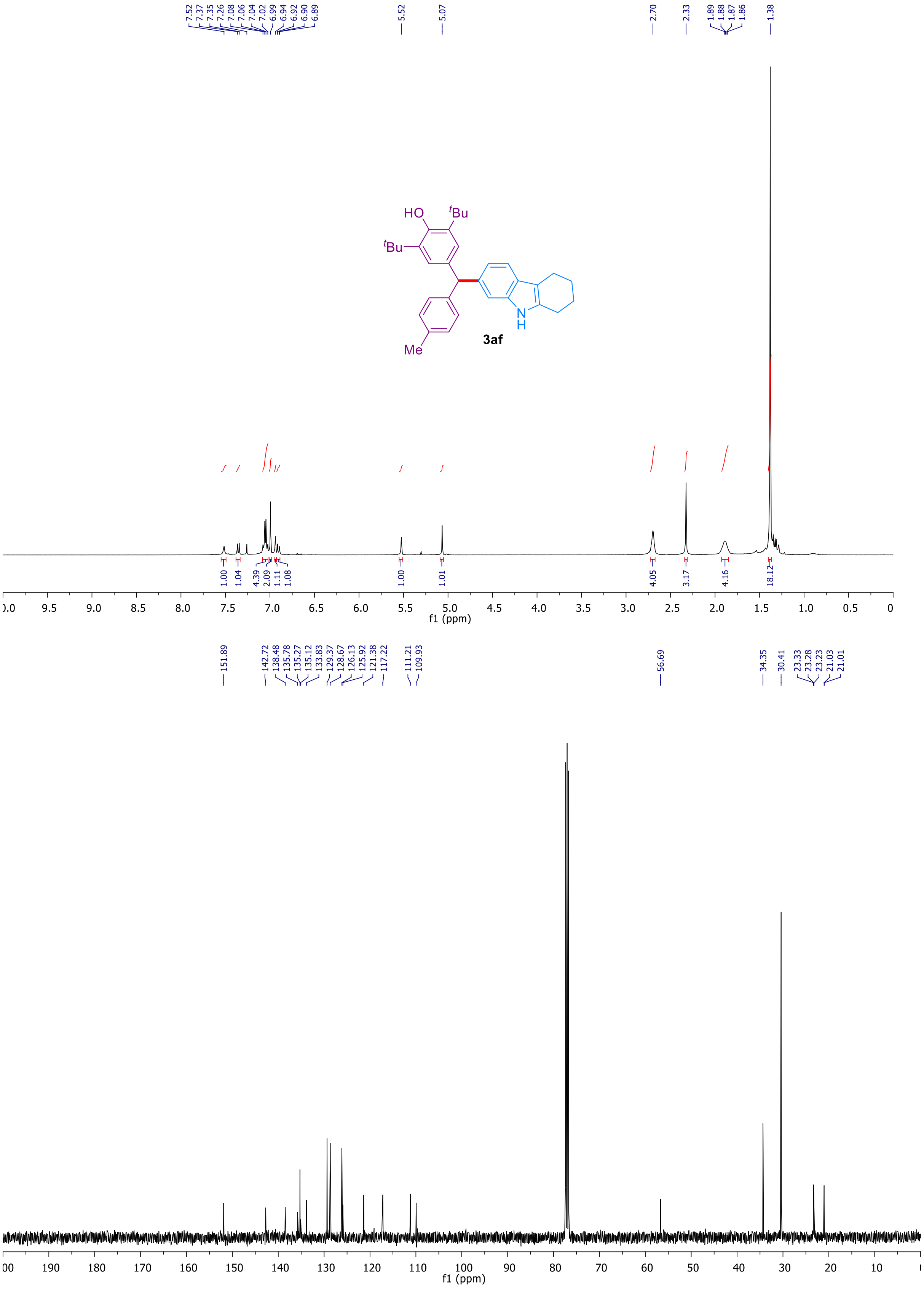

^{1}H NMR (400 MHz) and $^{13}C\{^{1}H\}$ NMR (100 MHz) spectra of **3af** ($CDCl_3$)

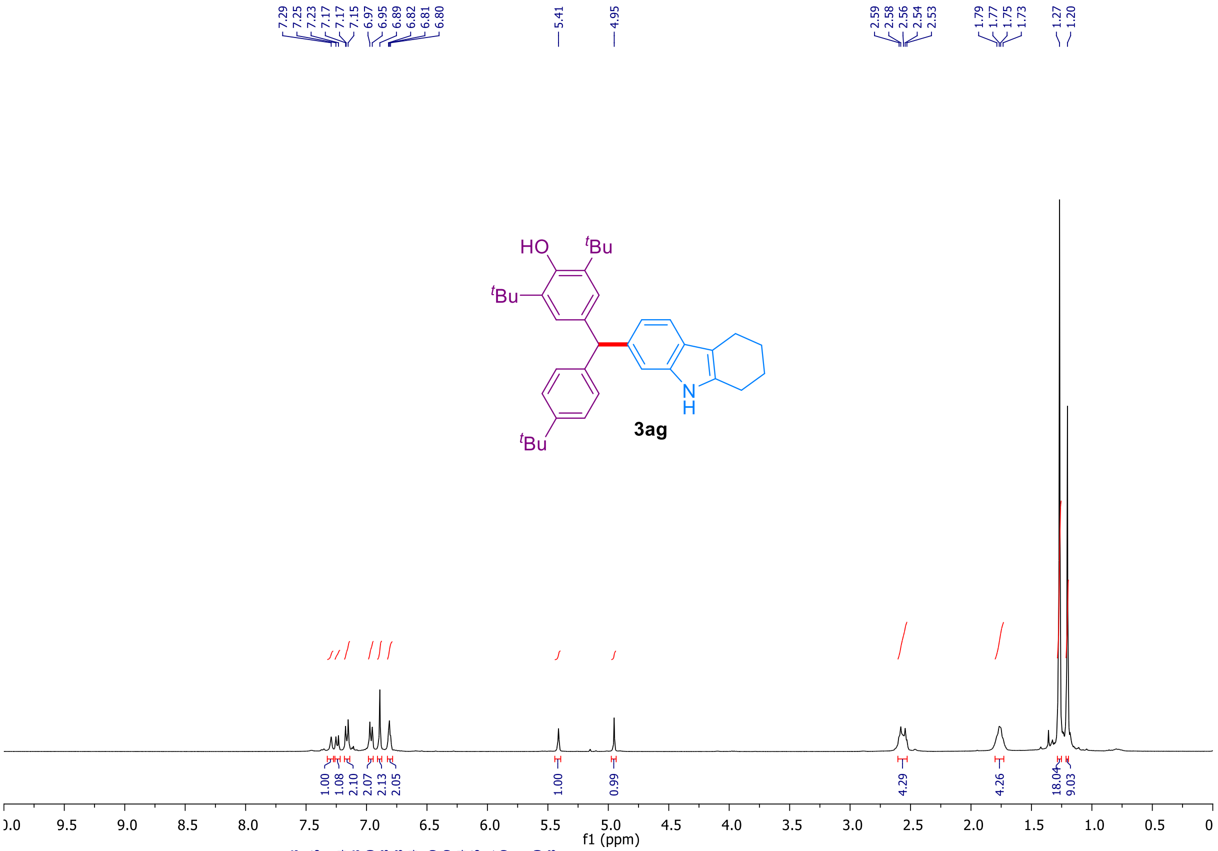

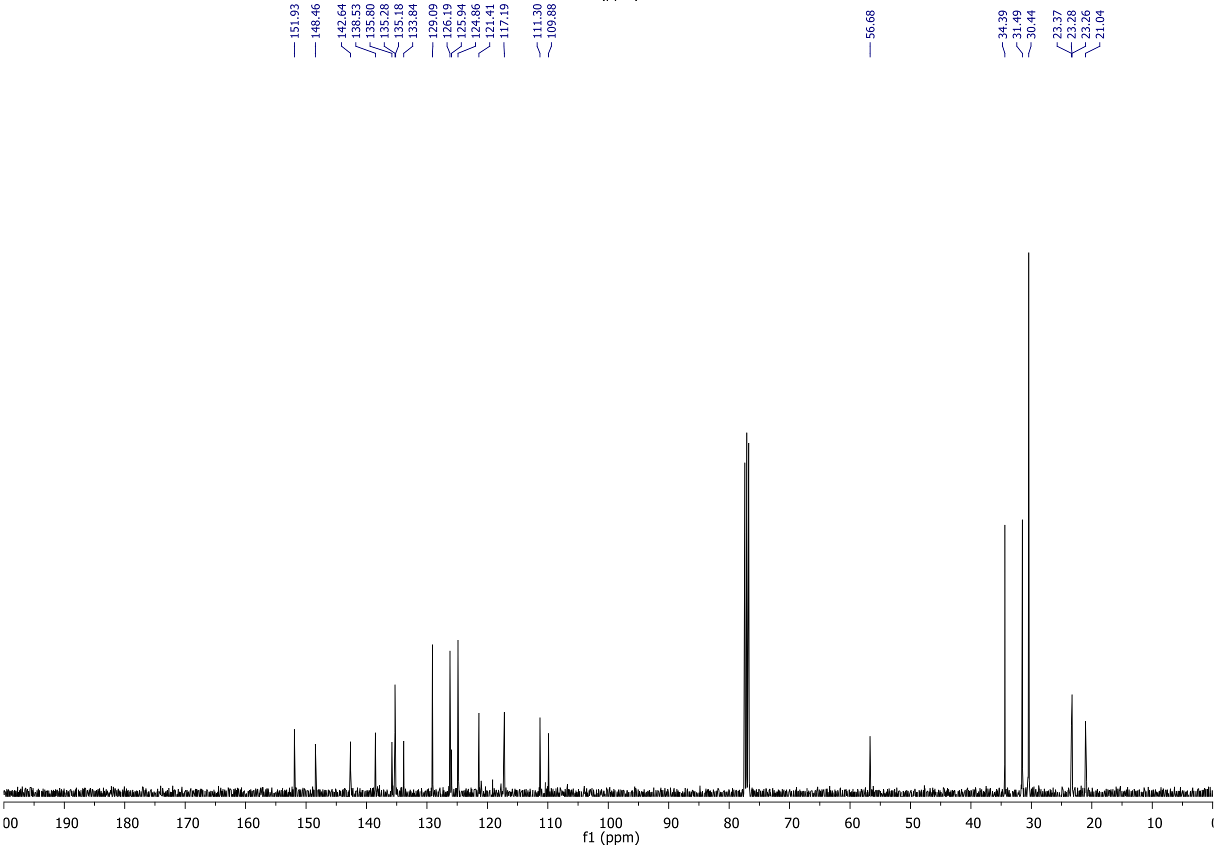

^{1}H NMR (400 MHz) and $^{13}C\{^{1}H\}$ NMR (100 MHz) spectra of **3ag** ($CDCl_3$)

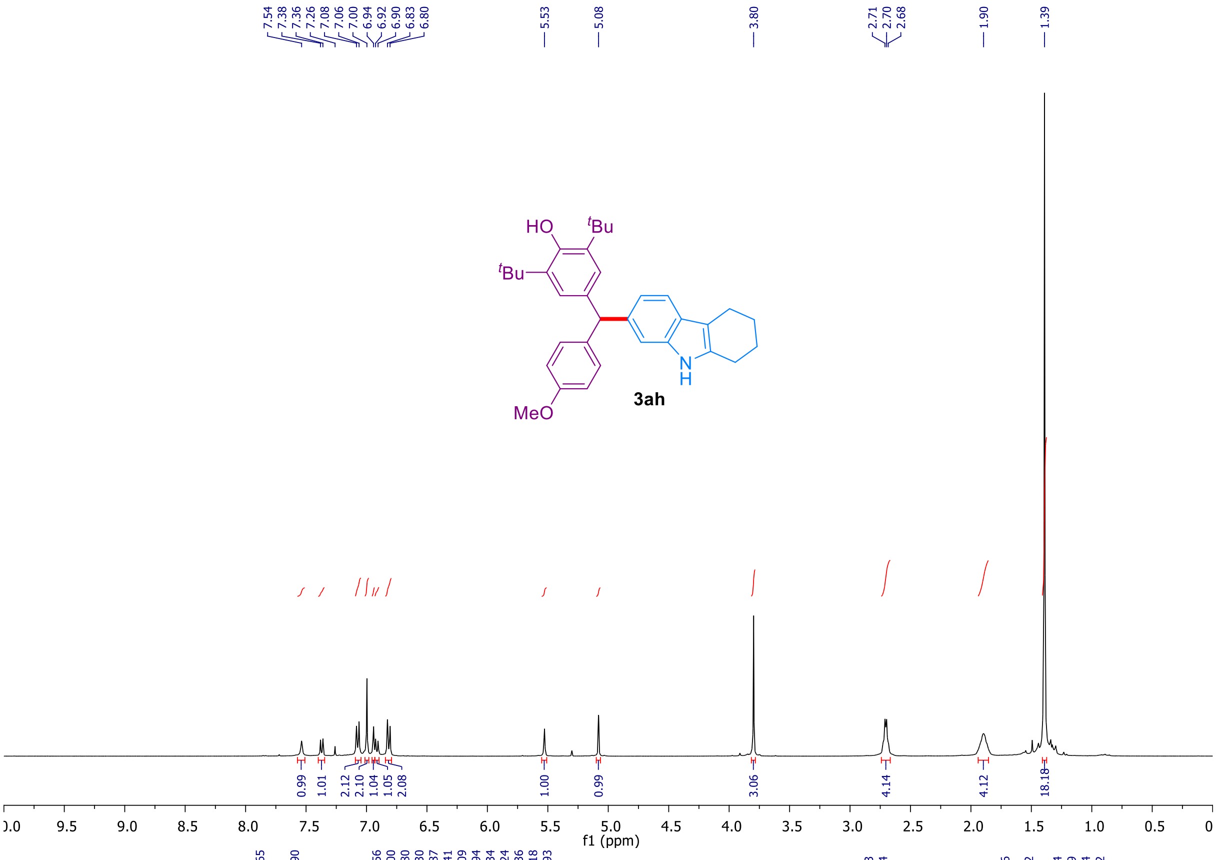

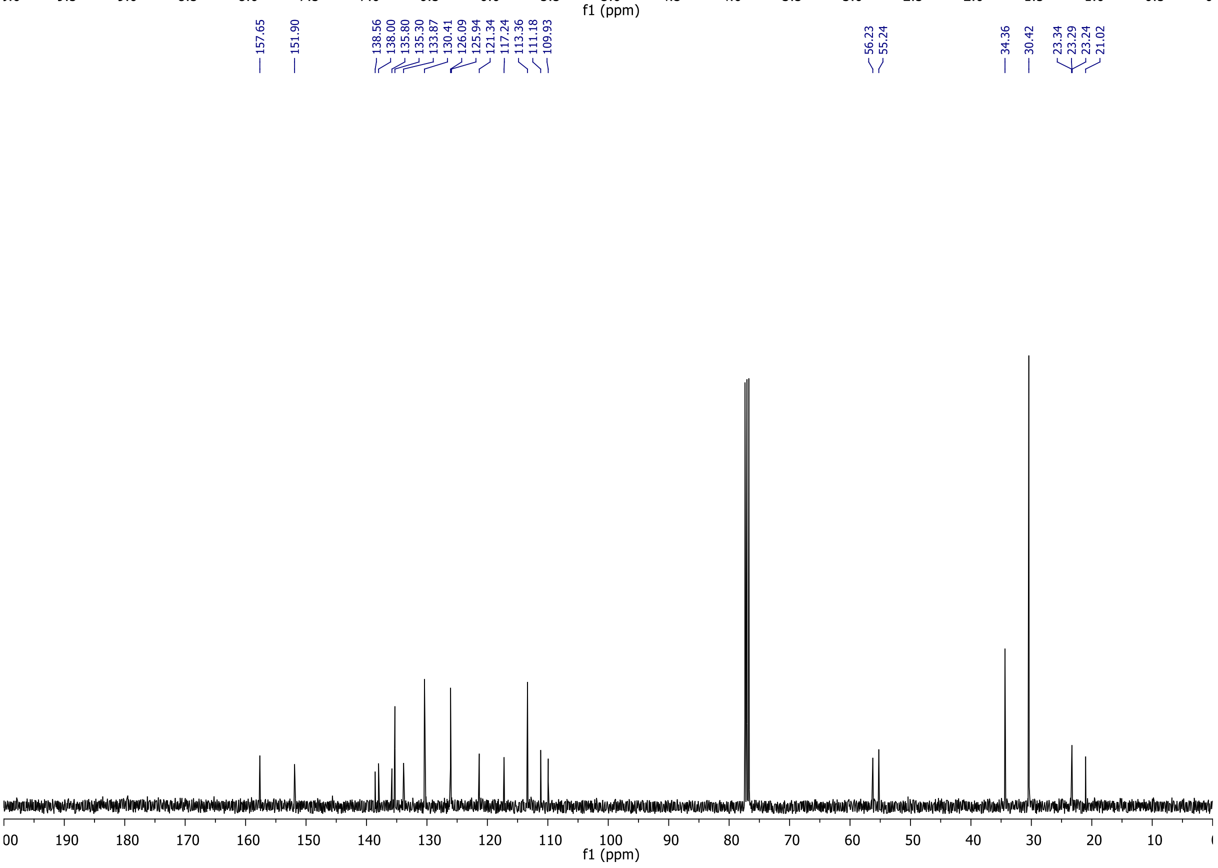

^{1}H NMR (400 MHz) and $^{13}C\{^{1}H\}$ NMR (100 MHz) spectra of **3ah** ($CDCl_3$)

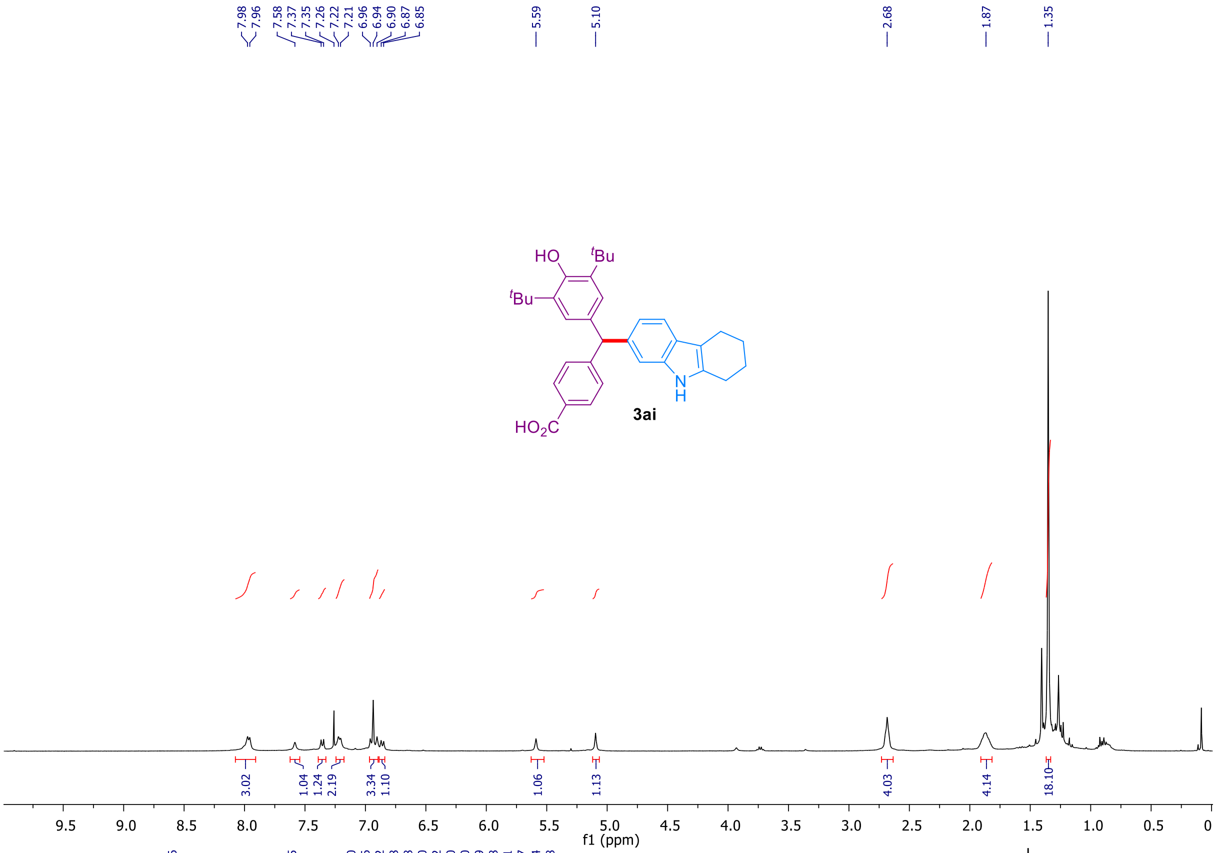

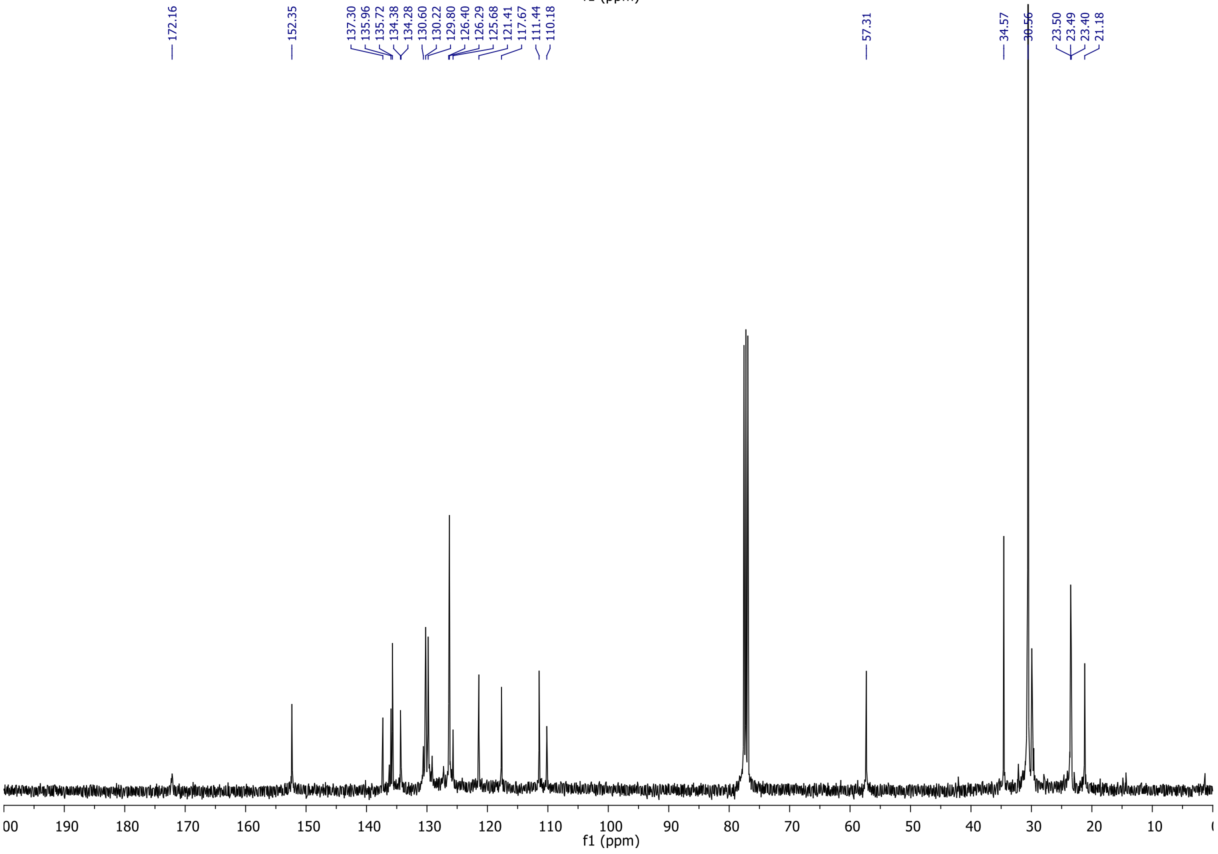

^{1}H NMR (400 MHz) and ^{13}C{^{1}H} NMR (100 MHz) spectra of **3ai** ($CDCl_3$)

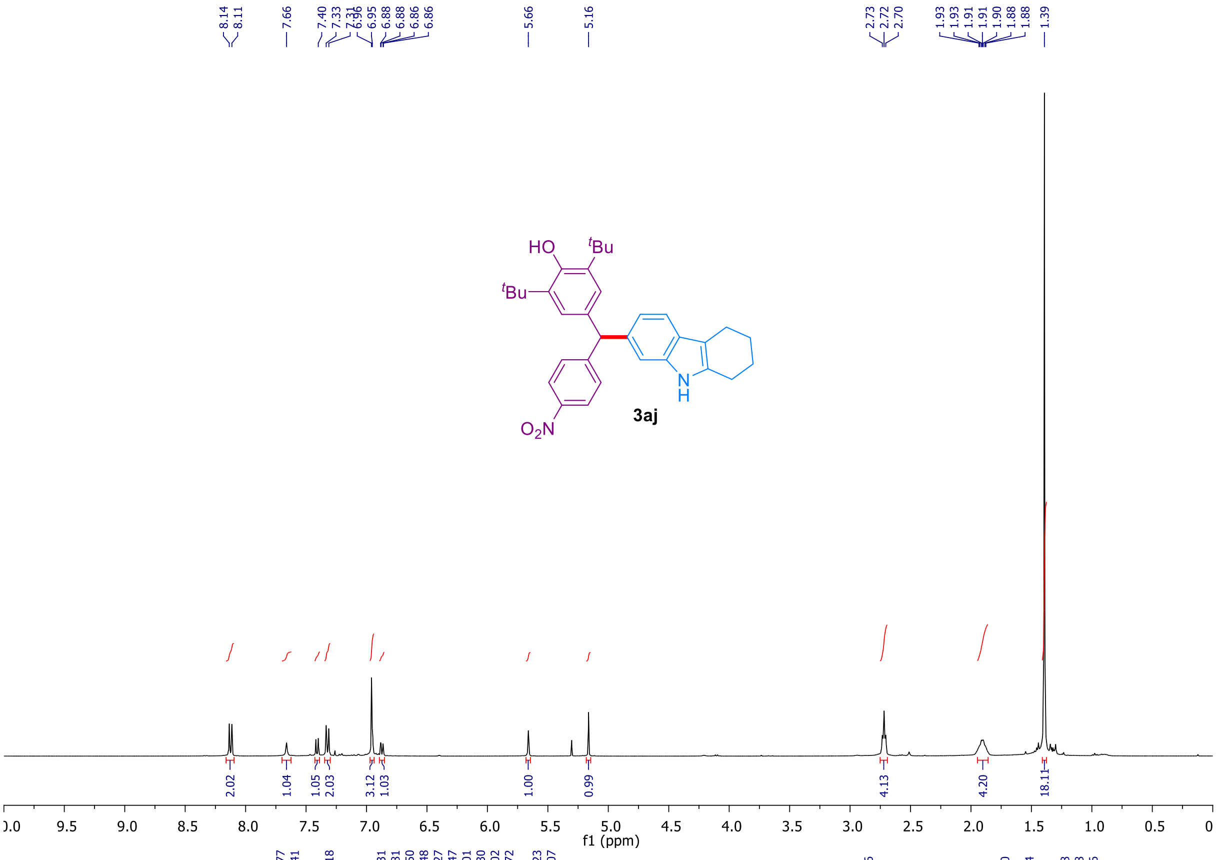

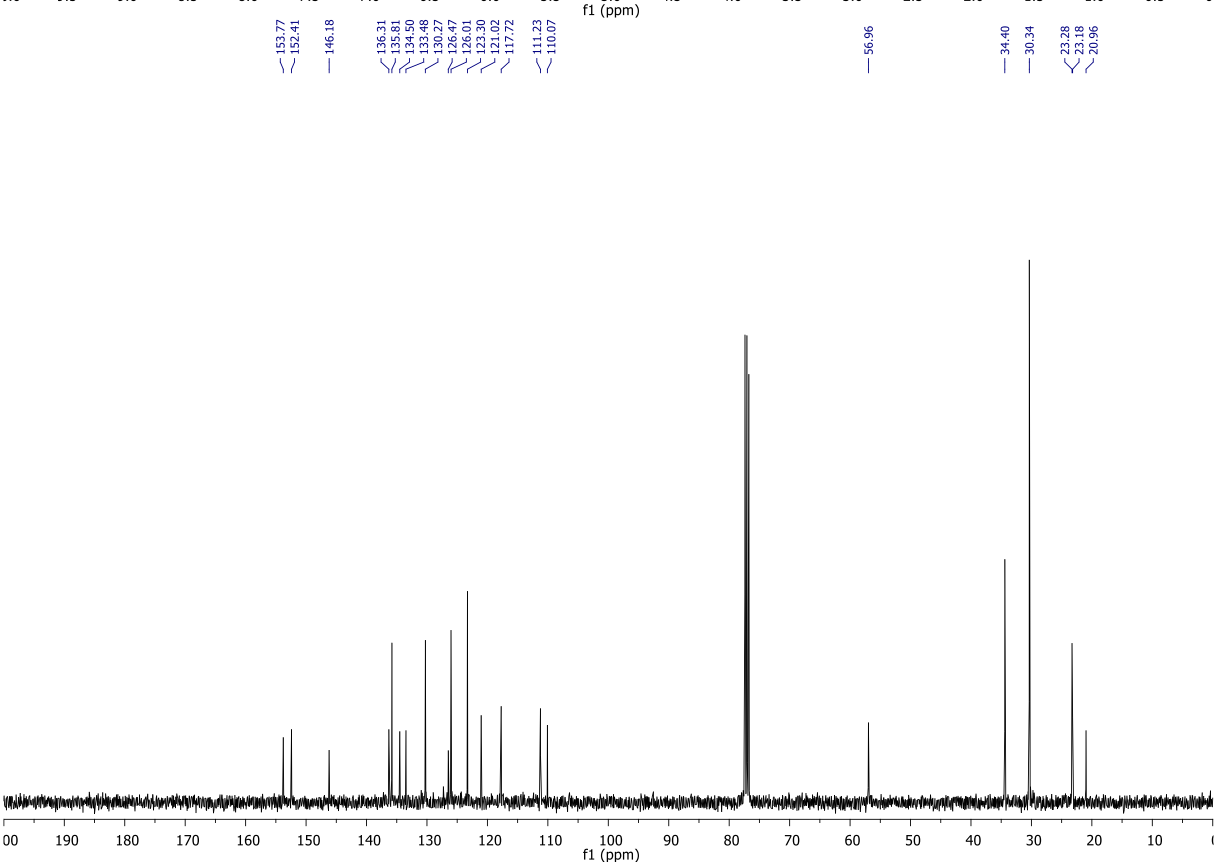

^{1}H NMR (400 MHz) and ^{13}C{^{1}H} NMR (100 MHz) spectra of **3aj** ($CDCl_3$)

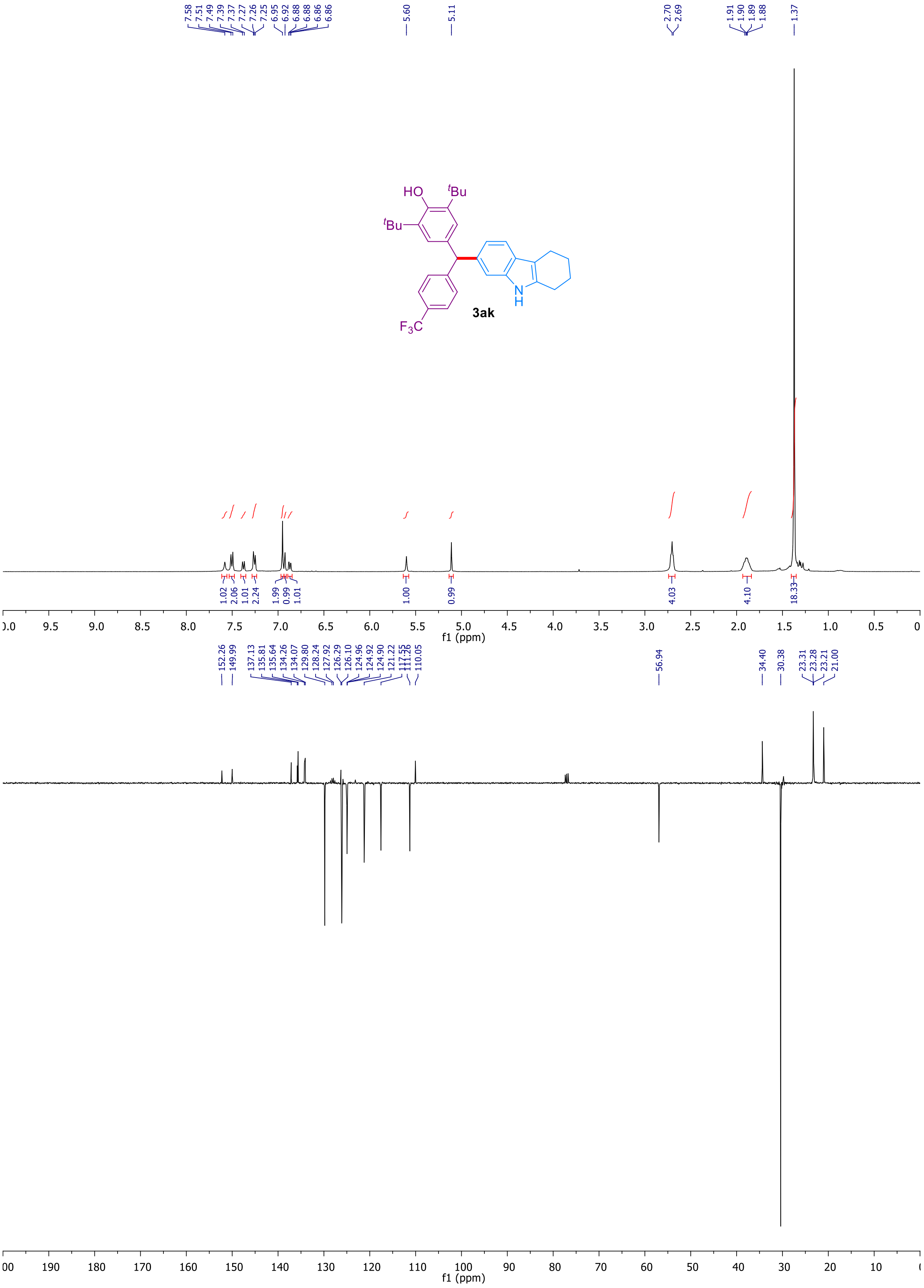

^{1}H NMR (400 MHz) and $^{13}C\{^{1}H\}$ NMR (100 MHz) spectra of **3ak** ($CDCl_3$)

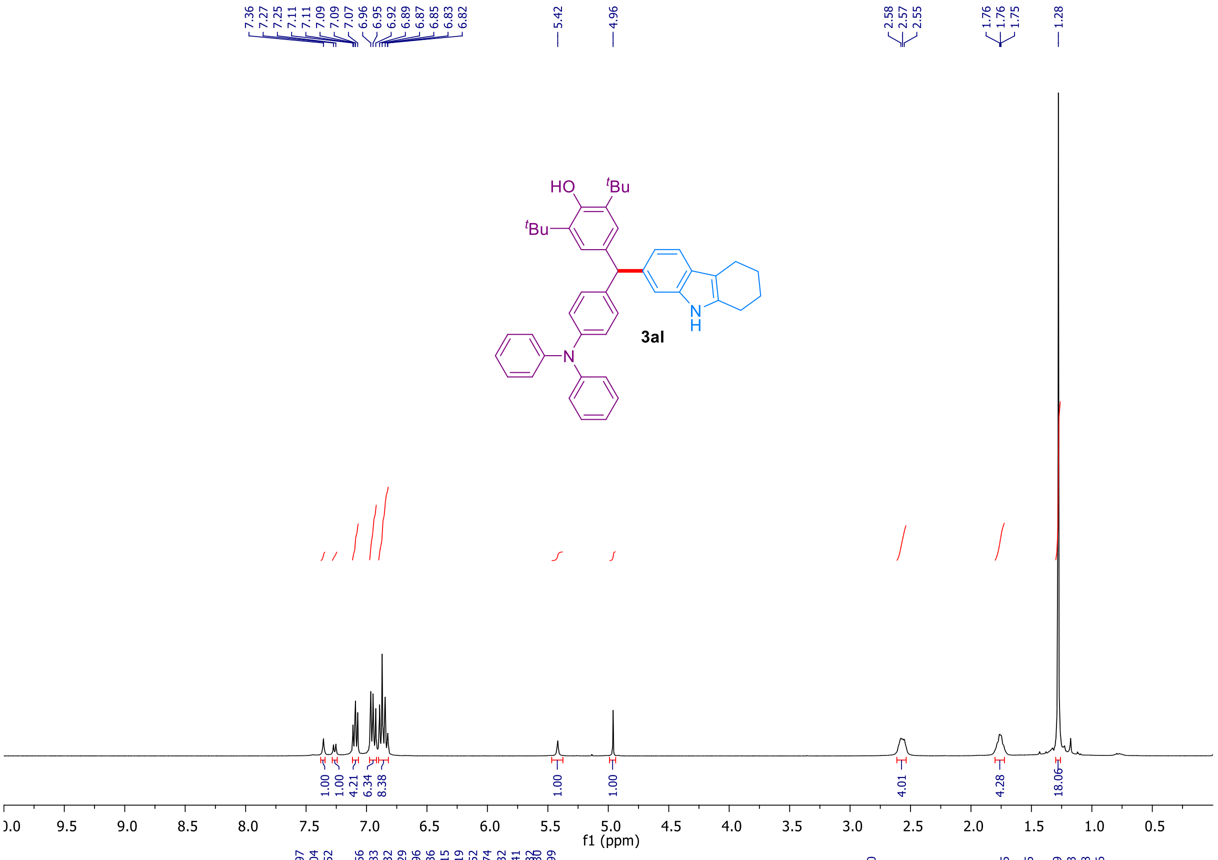

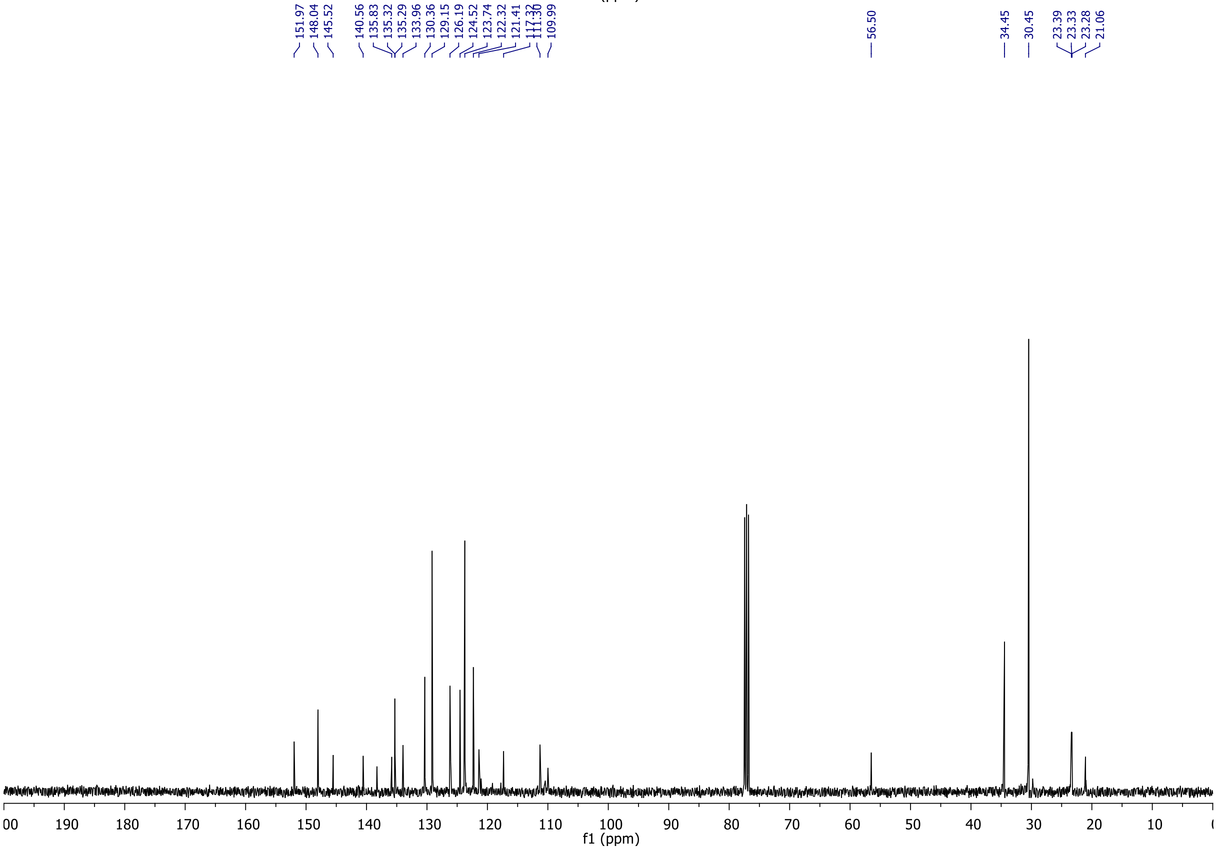

^{1}H NMR (400 MHz) and $^{13}C\{^{1}H\}$ NMR (100 MHz) spectra of **3al** ($CDCl_3$)

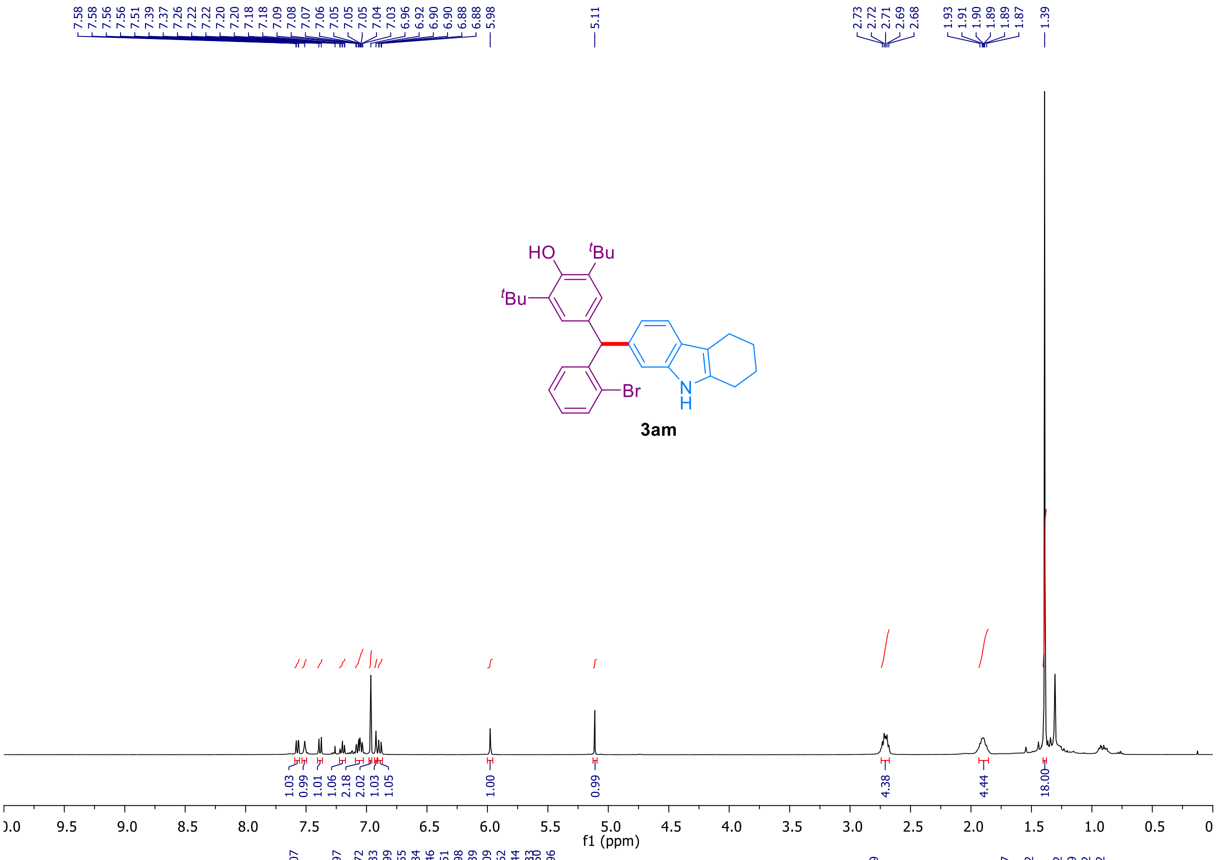

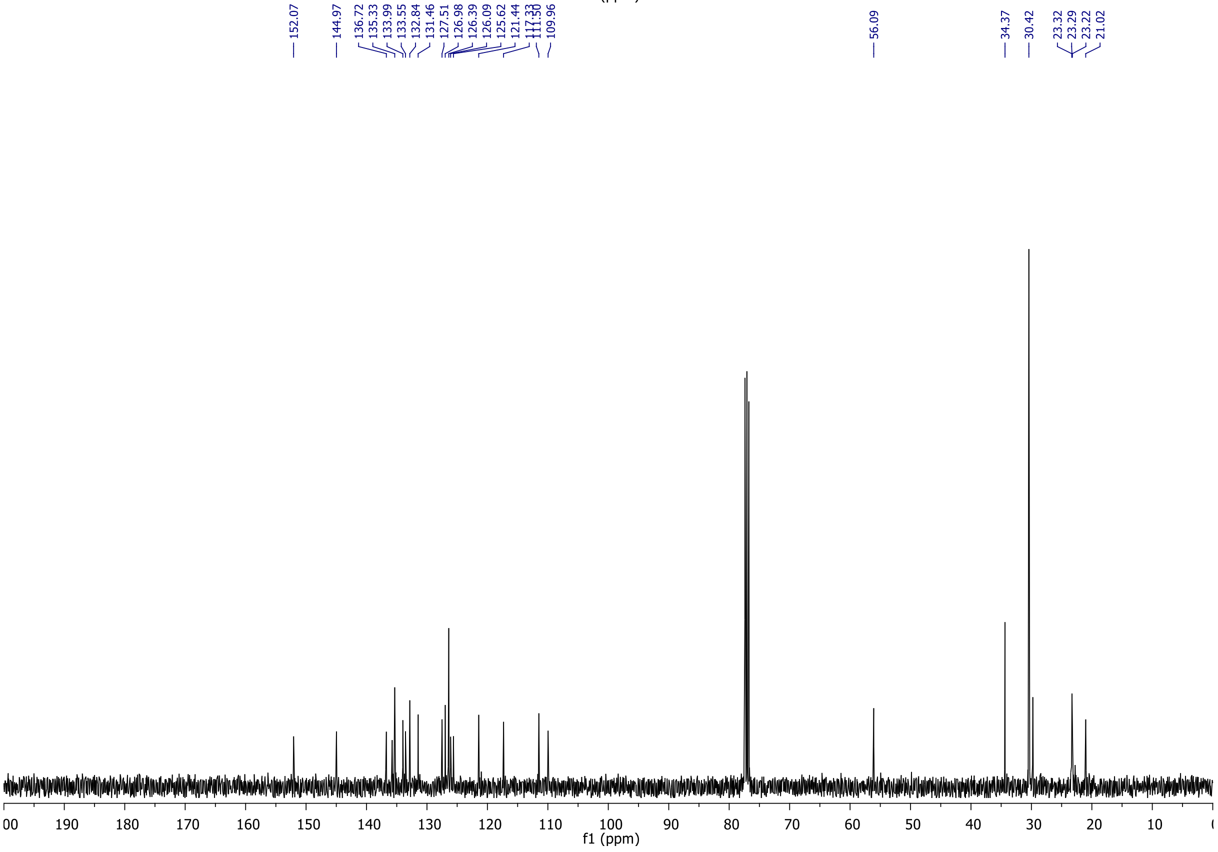

^{1}H NMR (400 MHz) and $^{13}C\{^{1}H\}$ NMR (100 MHz) spectra of **3am** ($CDCl_3$)

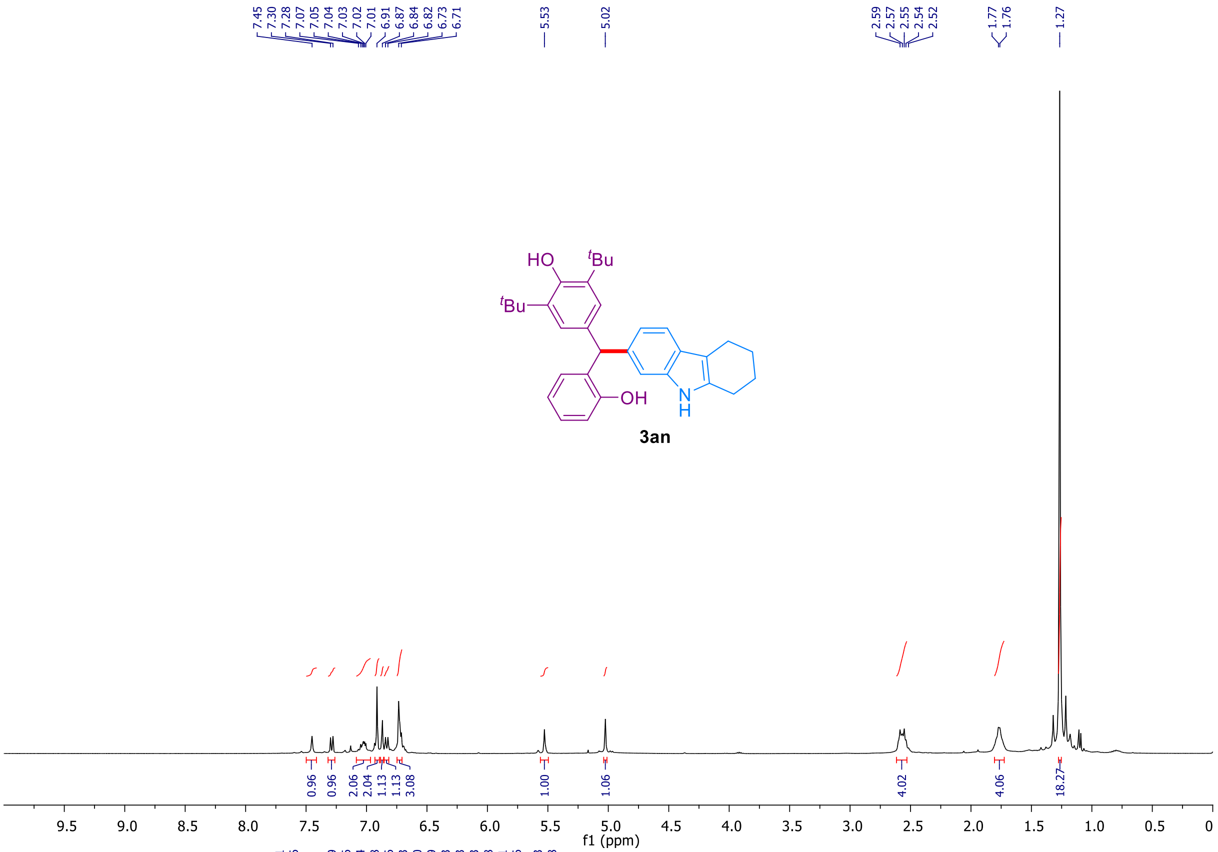

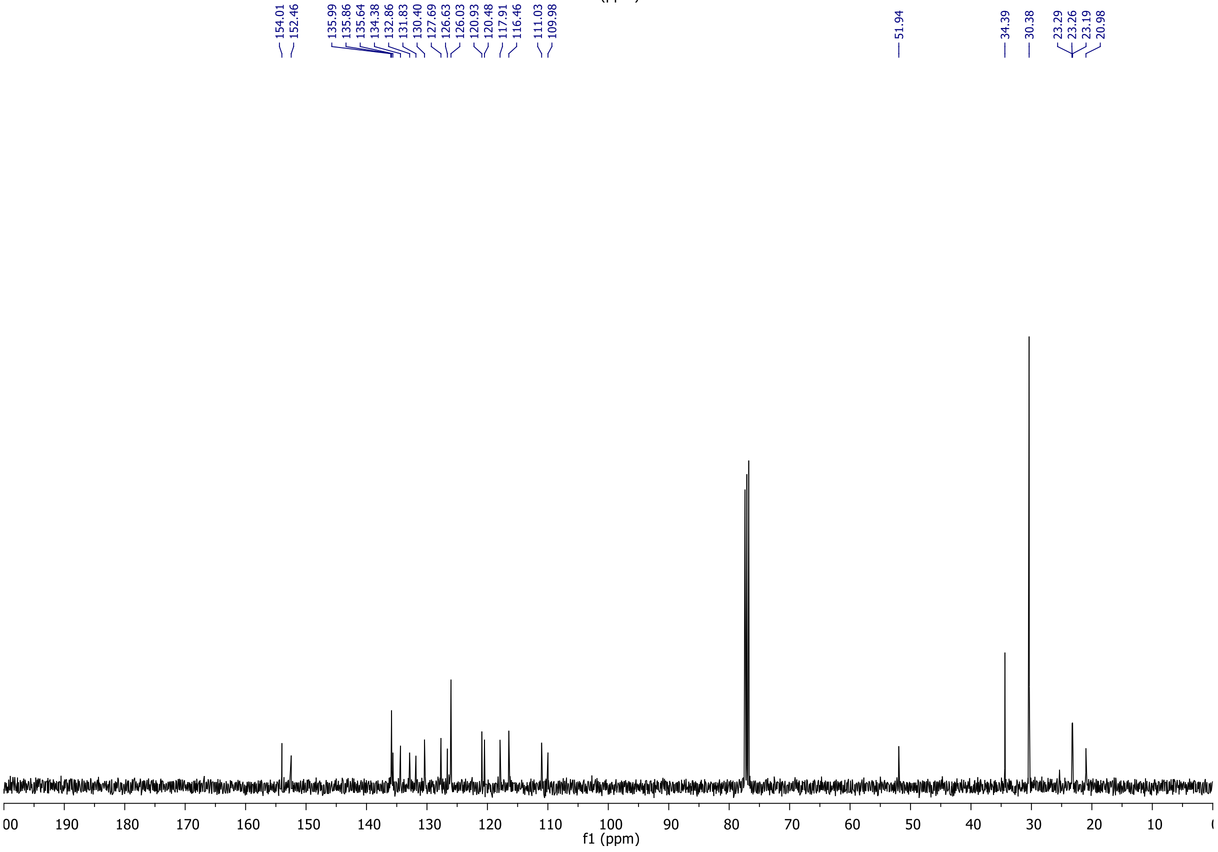

^{1}H NMR (400 MHz) and ^{13}C{^{1}H} NMR (100 MHz) spectra **3an** (CDCl$_3$)

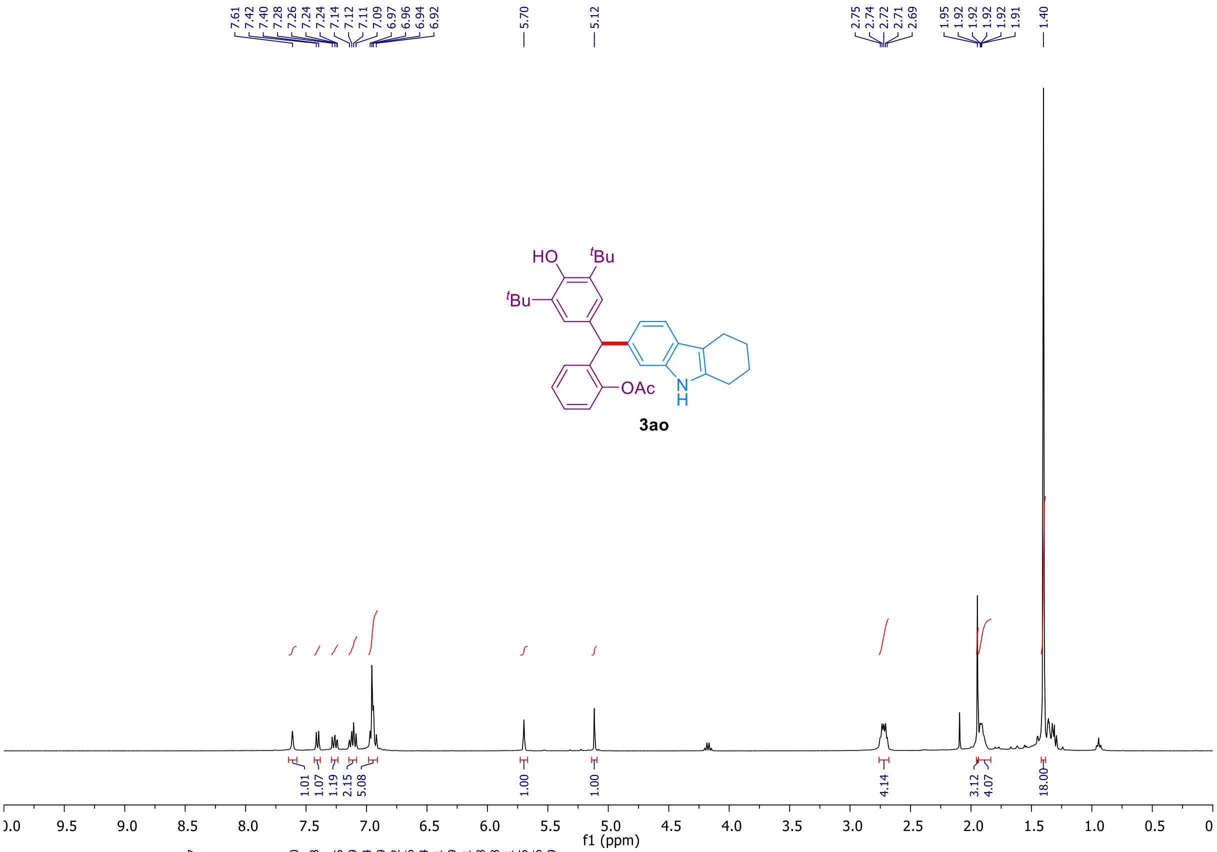

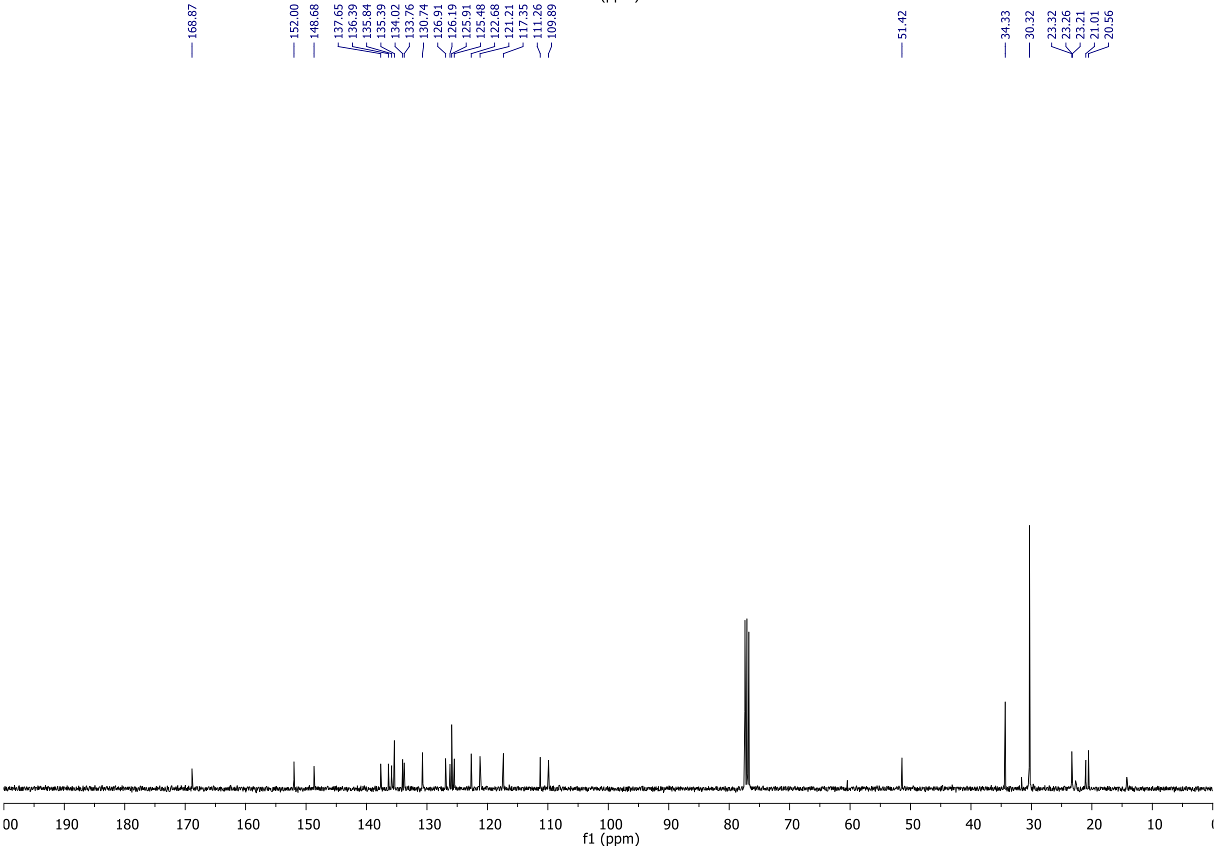

^{1}H NMR (400 MHz) and ^{13}C{^{1}H} NMR (100 MHz) spectra of **3ao** ($CDCl_3$)

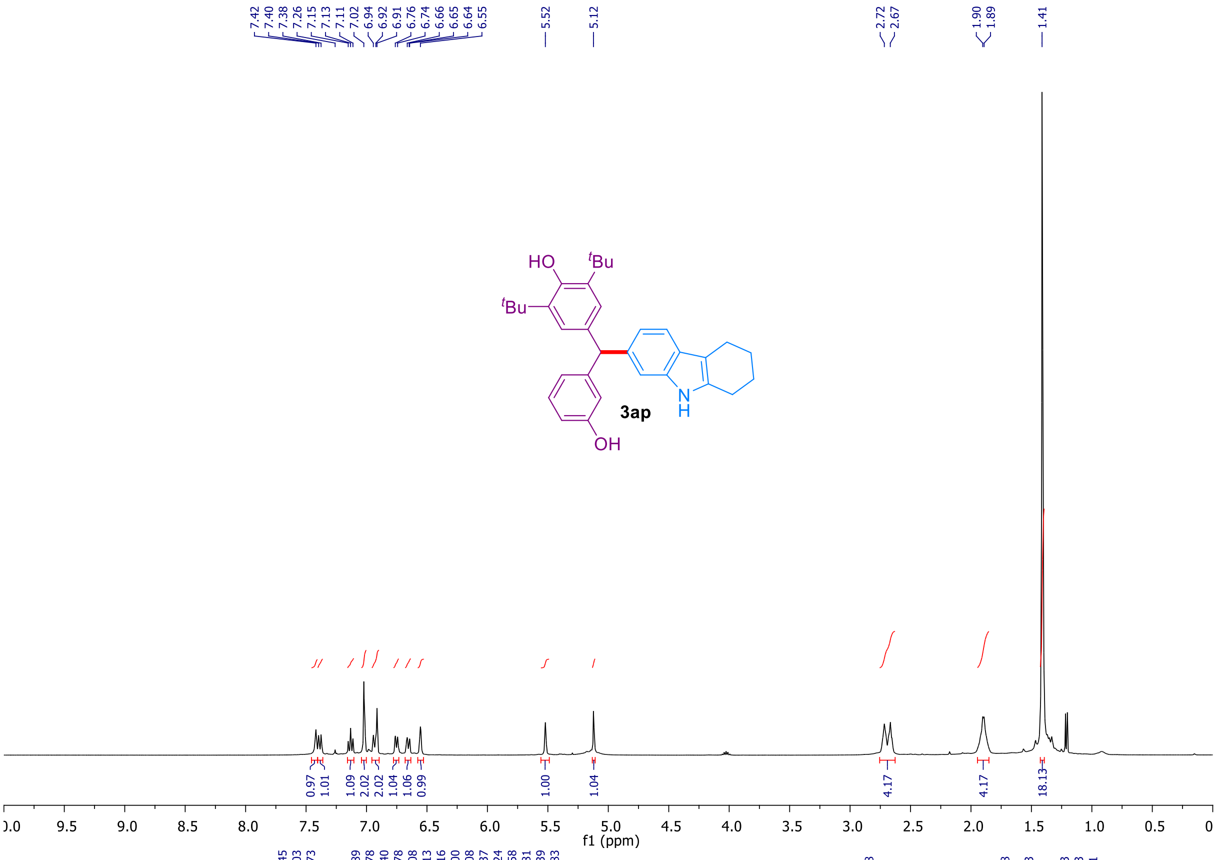

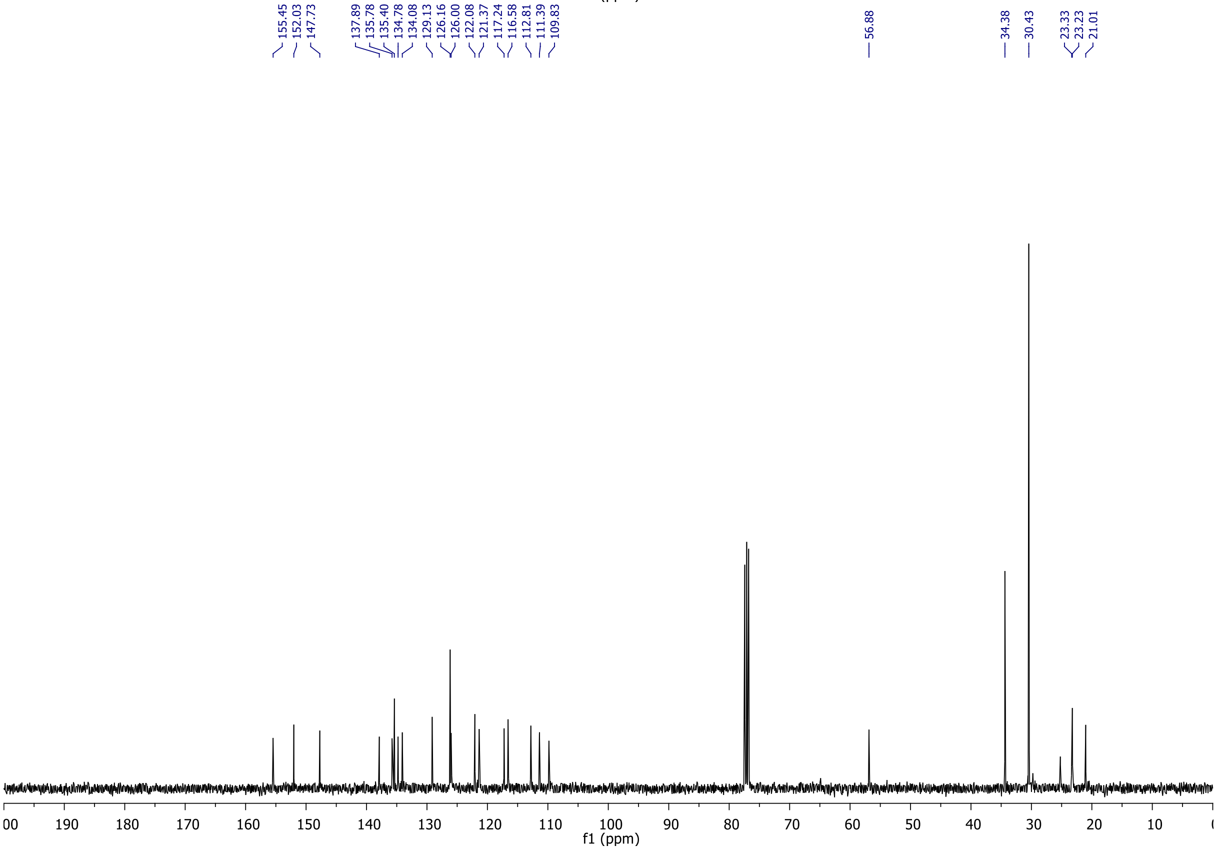

^{1}H NMR (400 MHz) and ^{13}C{^{1}H} NMR (100 MHz) spectra of **3ap** ($CDCl_3$)

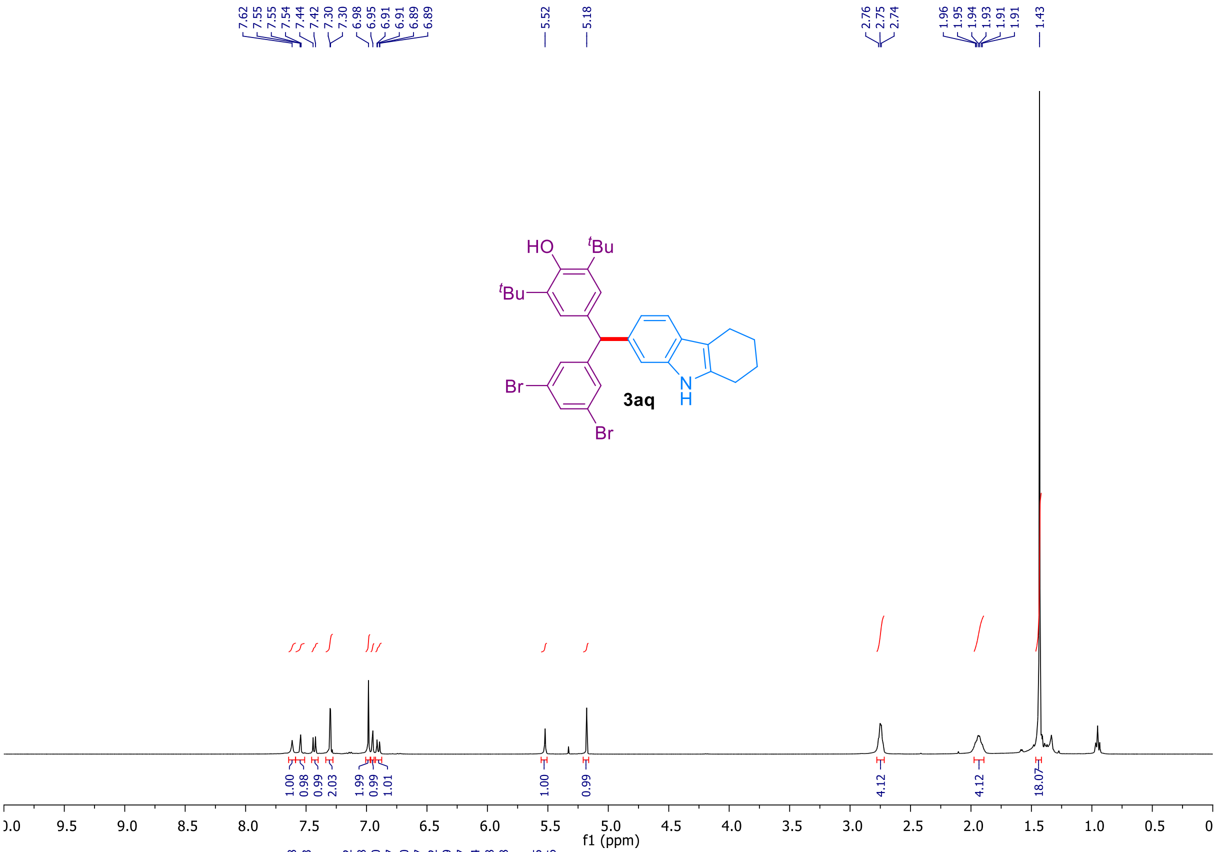

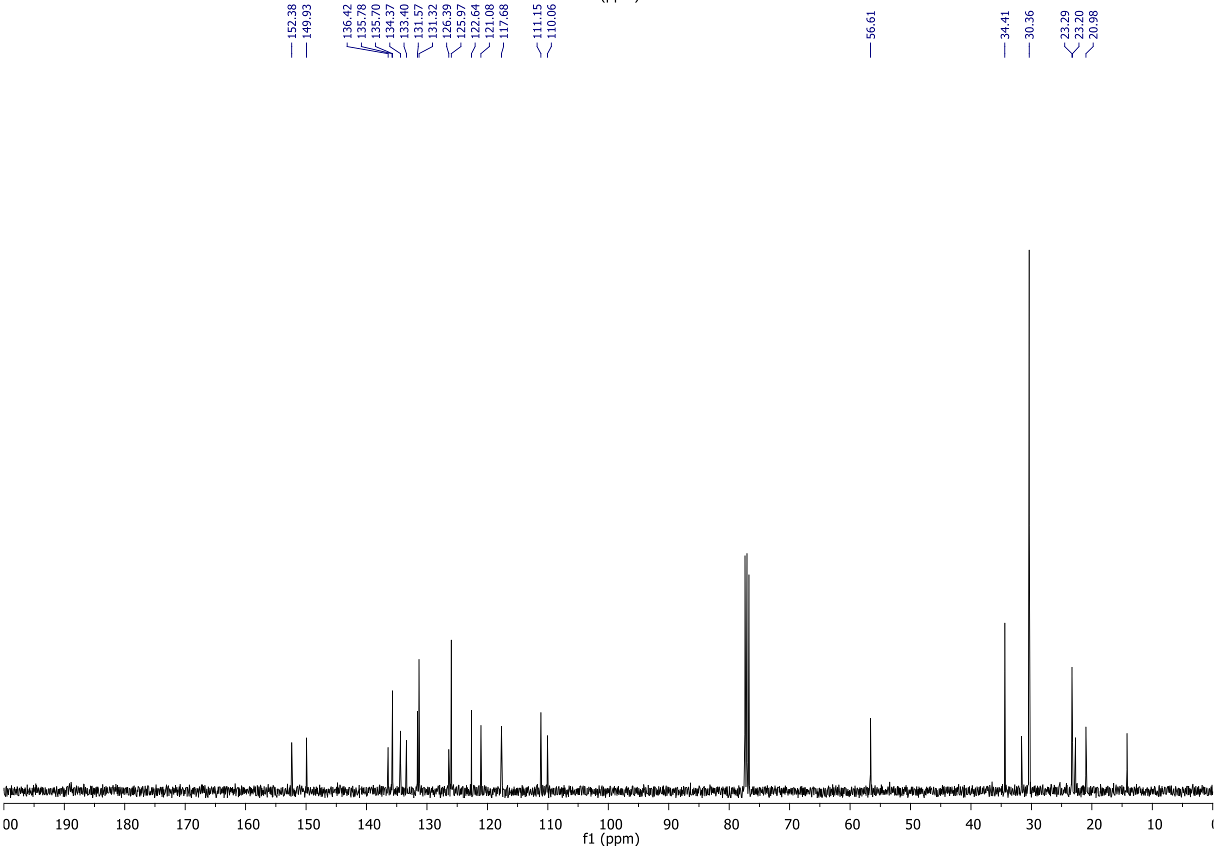

^{1}H NMR (400 MHz) and $^{13}C\{^{1}H\}$ NMR (100 MHz) spectra of **3aq** ($CDCl_3$)

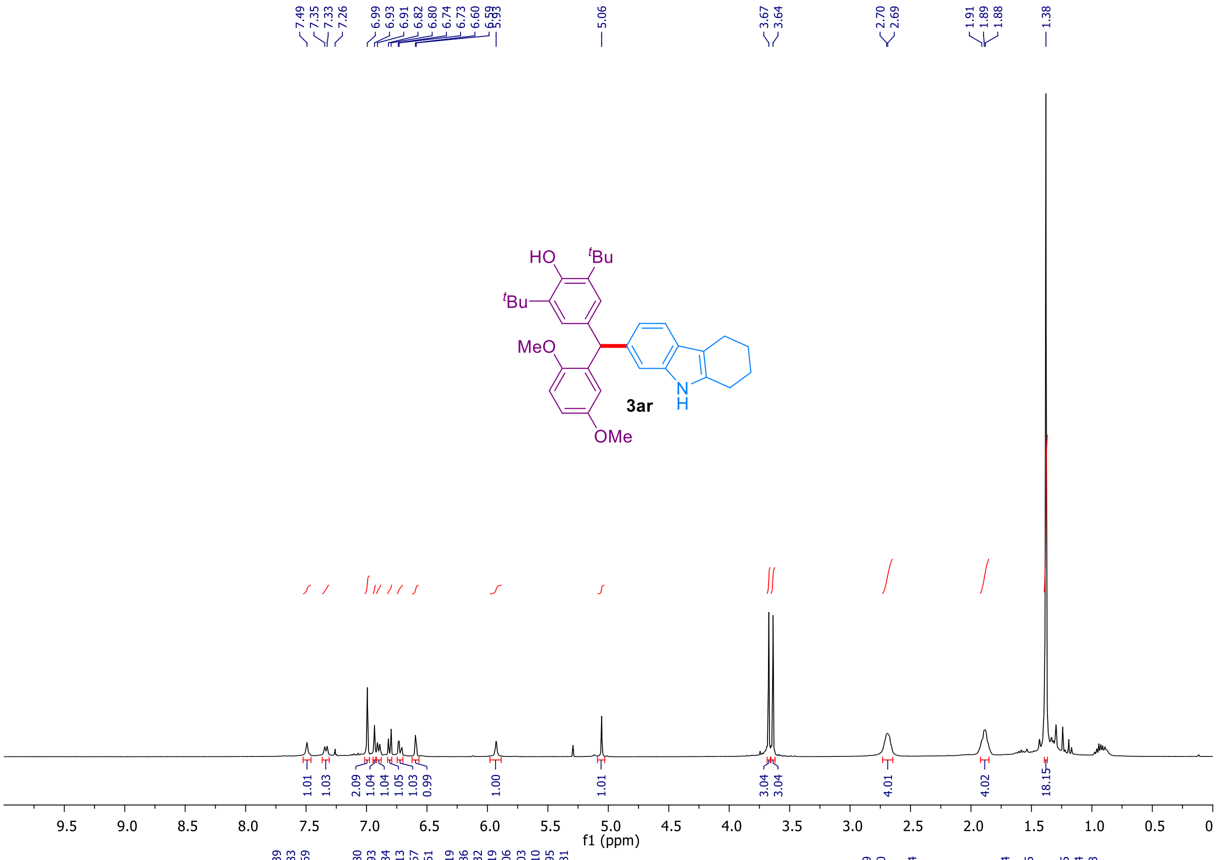

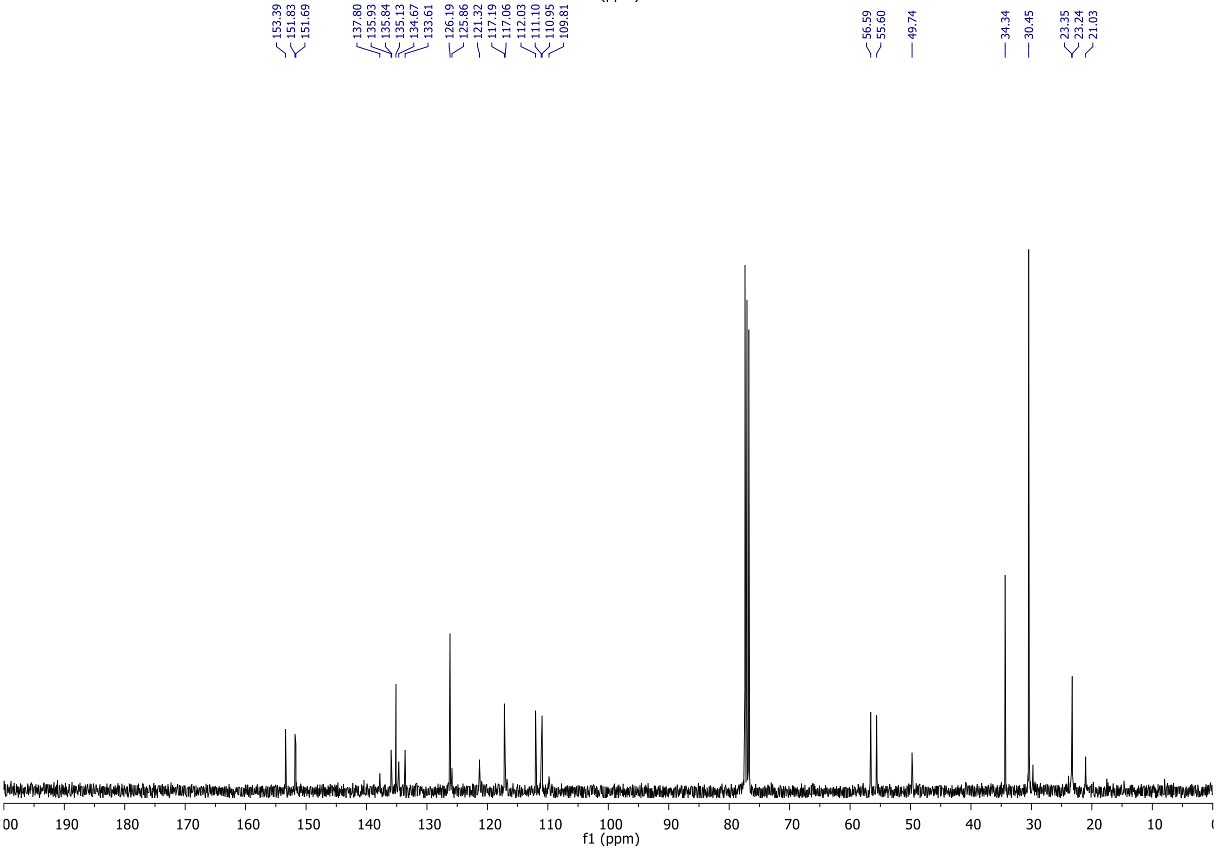

^{1}H NMR (400 MHz) and $^{13}C\{^{1}H\}$ NMR (100 MHz) spectra of **3ar** ($CDCl_3$)

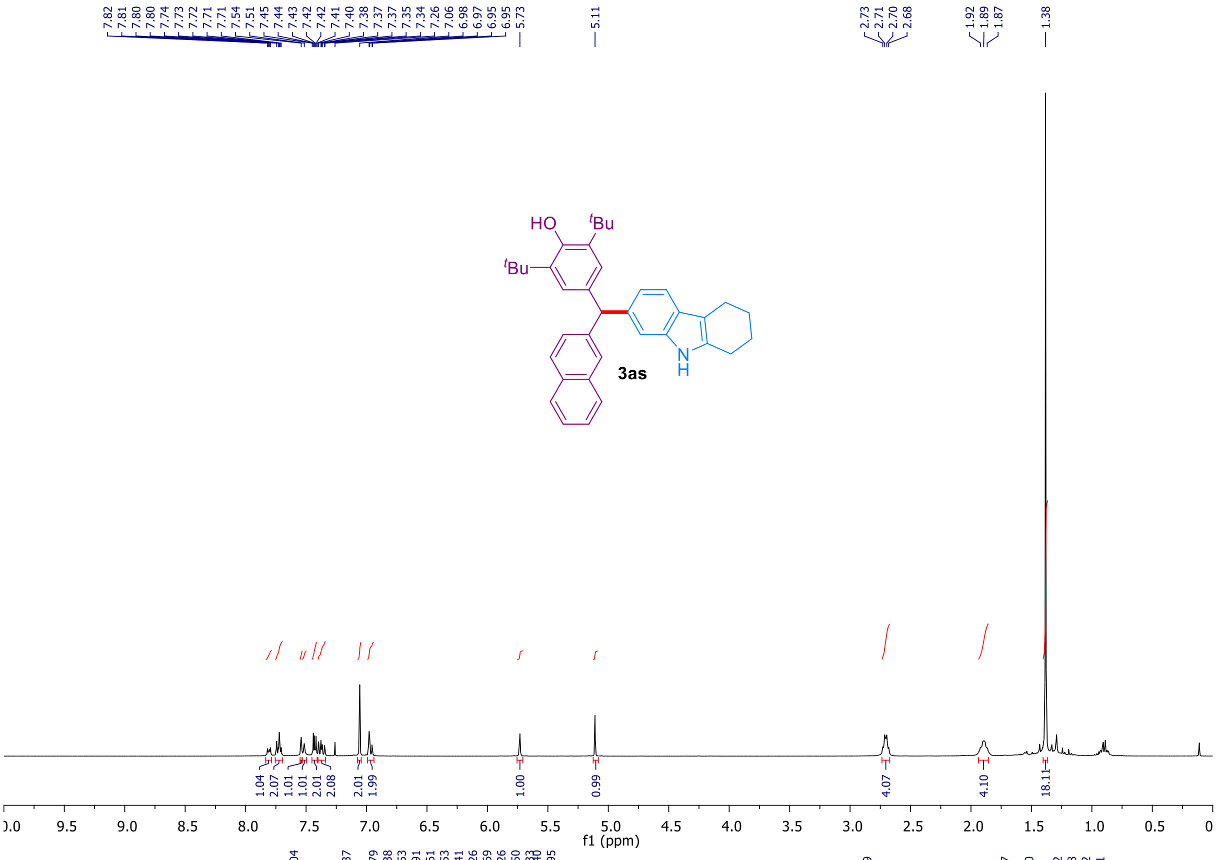

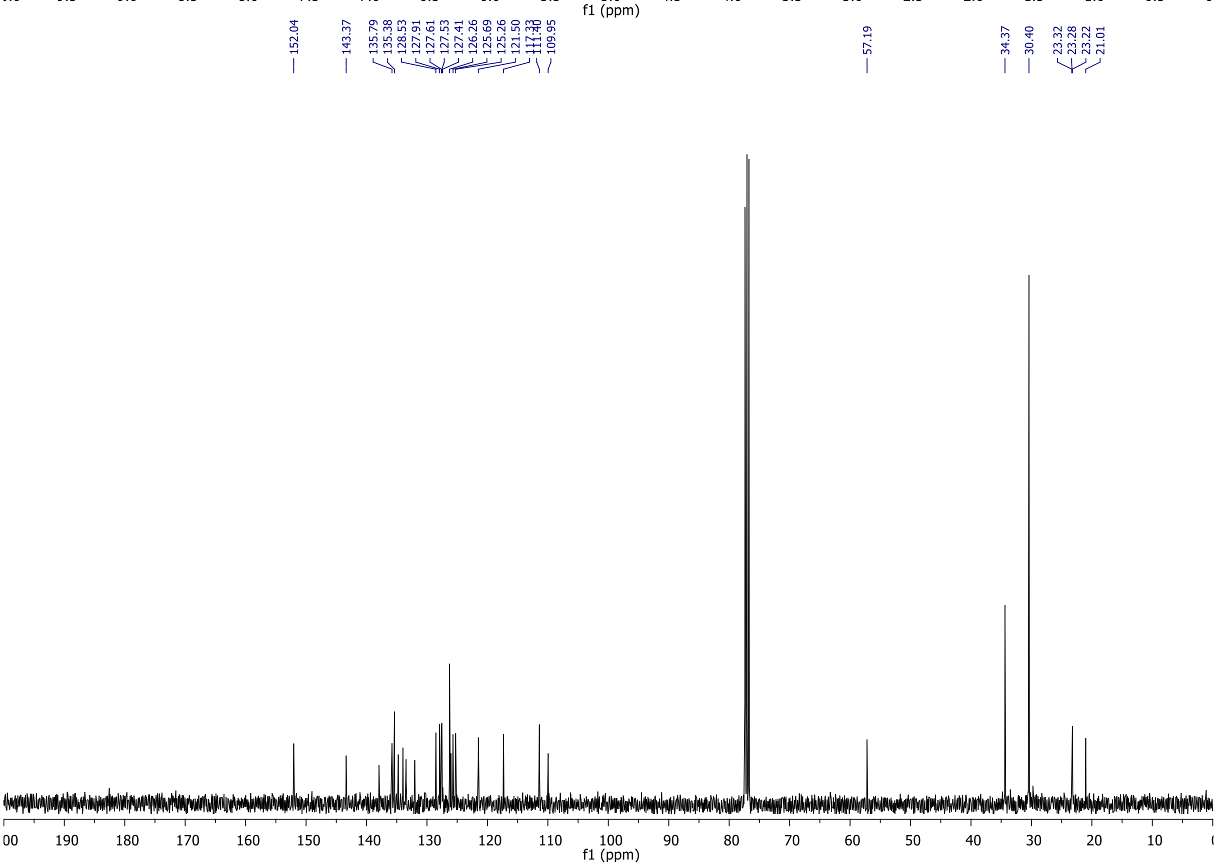

^{1}H NMR (400 MHz) and $^{13}C\{^{1}H\}$ NMR (100 MHz) spectra of **3as** ($CDCl_3$)

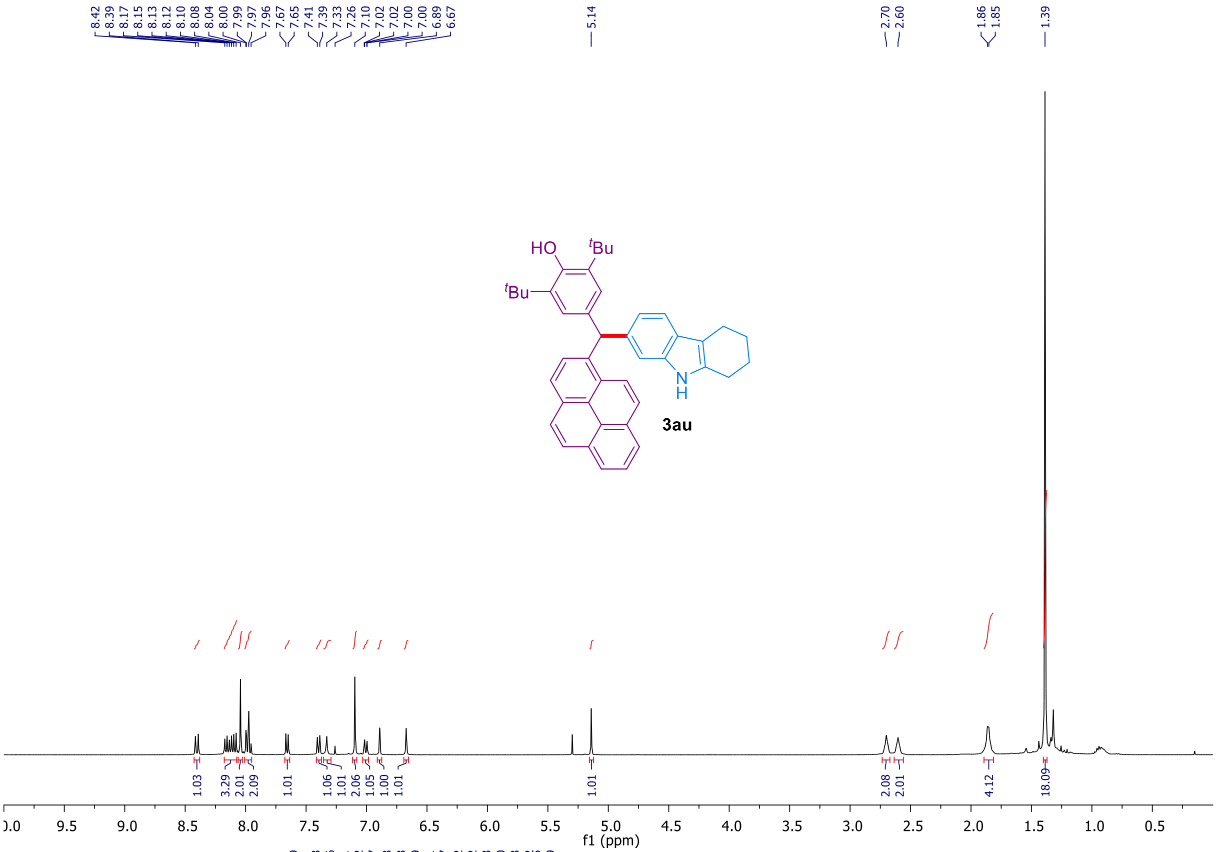

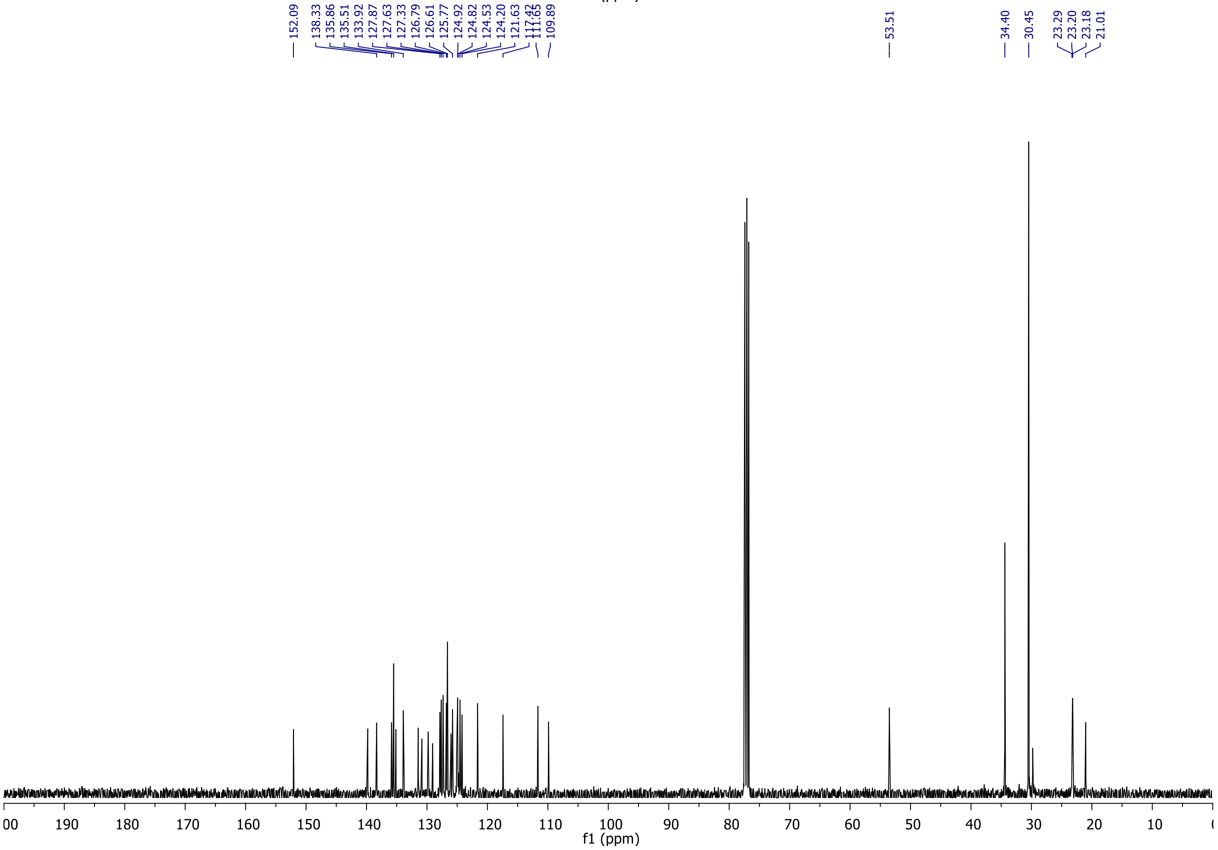

^{1}H NMR (400 MHz) and ^{13}C{^{1}H} NMR (100 MHz) spectra of **3au** ($CDCl_3$)

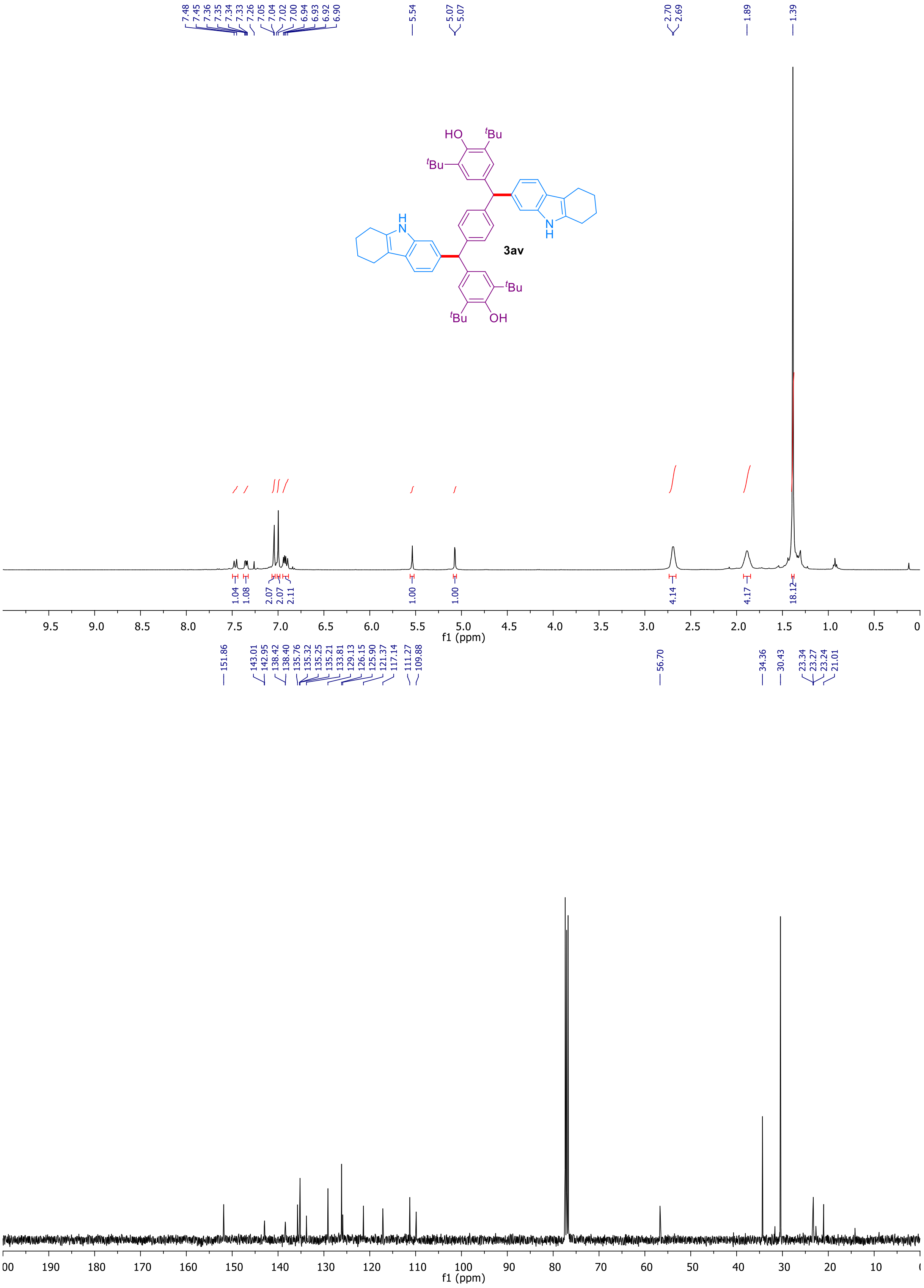

^{1}H NMR (400 MHz) and ^{13}C{^{1}H} NMR (100 MHz) spectra of **3av** ($CDCl_3$)

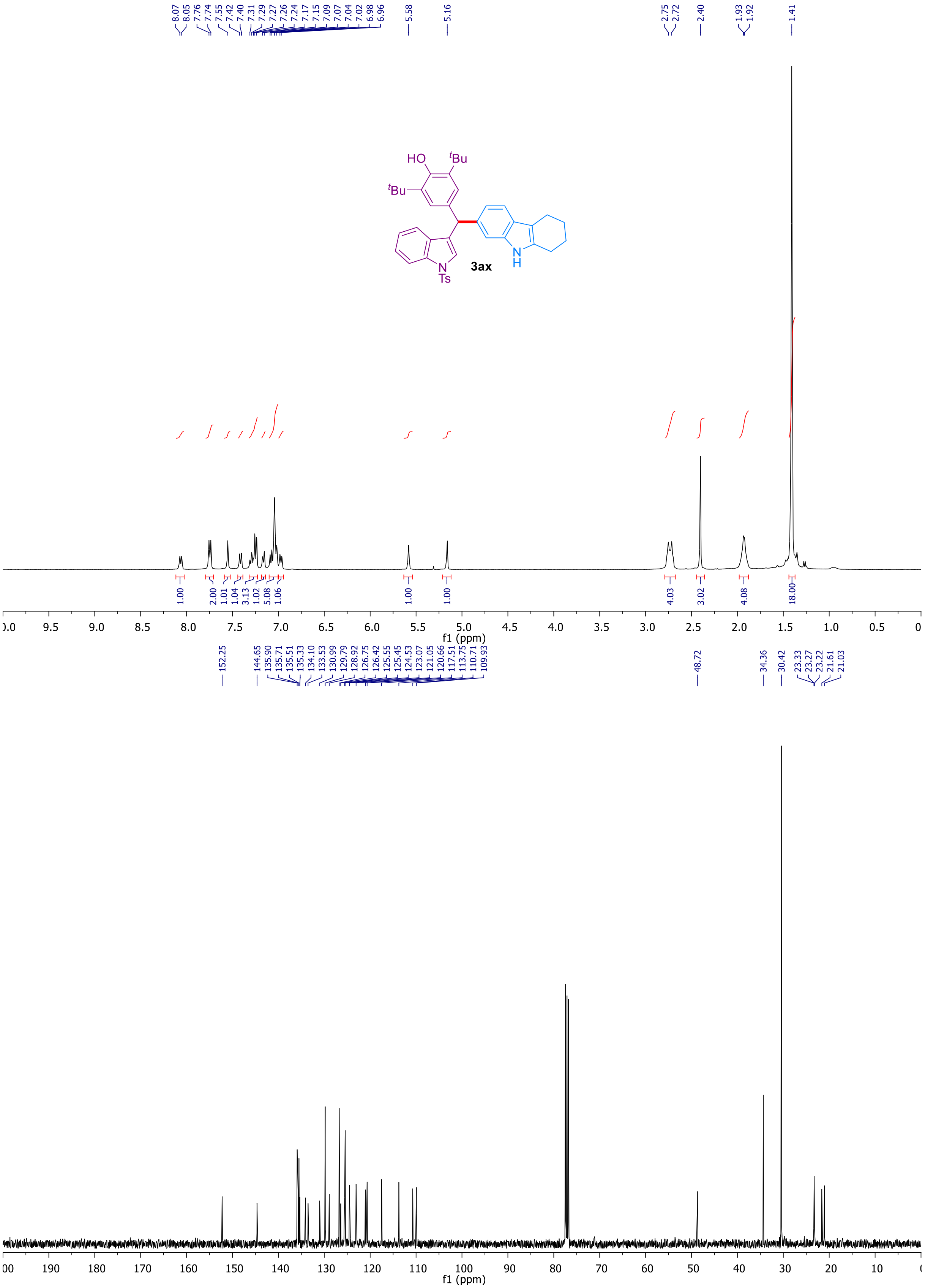

^{1}H NMR (400 MHz) and $^{13}C\{^{1}H\}$ NMR (100 MHz) spectra of **3ax** ($CDCl_3$)

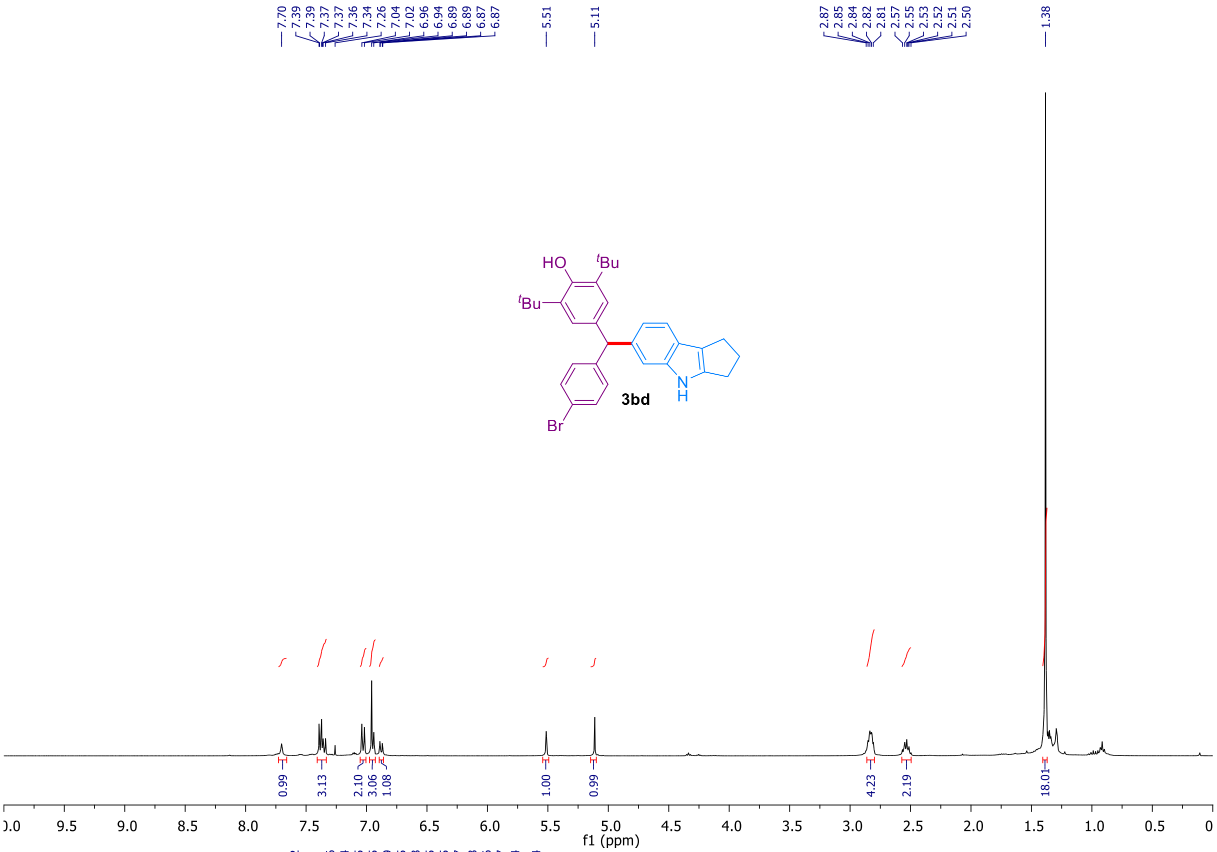

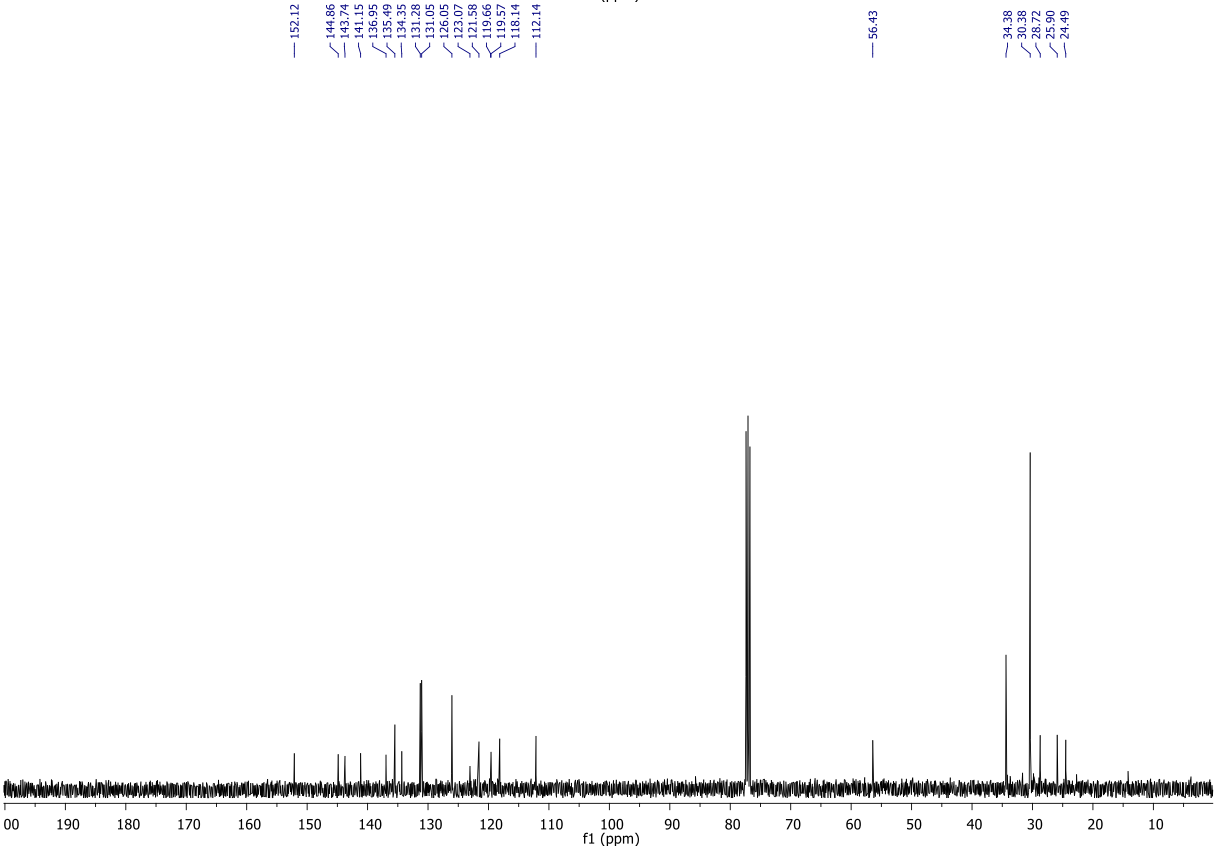

^{1}H NMR (400 MHz) and $^{13}C\{^{1}H\}$ NMR (100 MHz) spectra of **3bd** ($CDCl_3$)

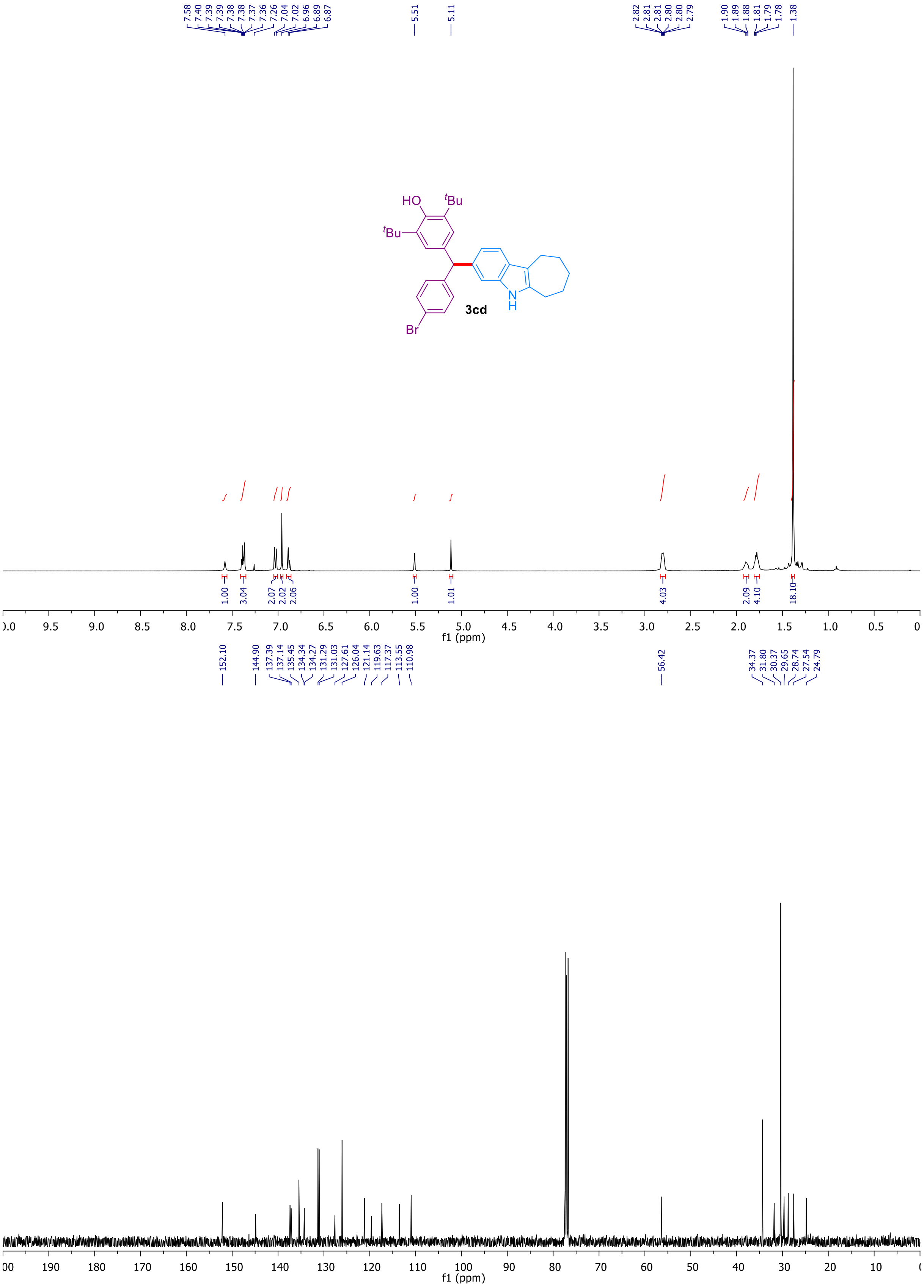

^{1}H NMR (400 MHz) and ^{13}C{^{1}H} NMR (100 MHz) spectra of **3cd** ($CDCl_3$)

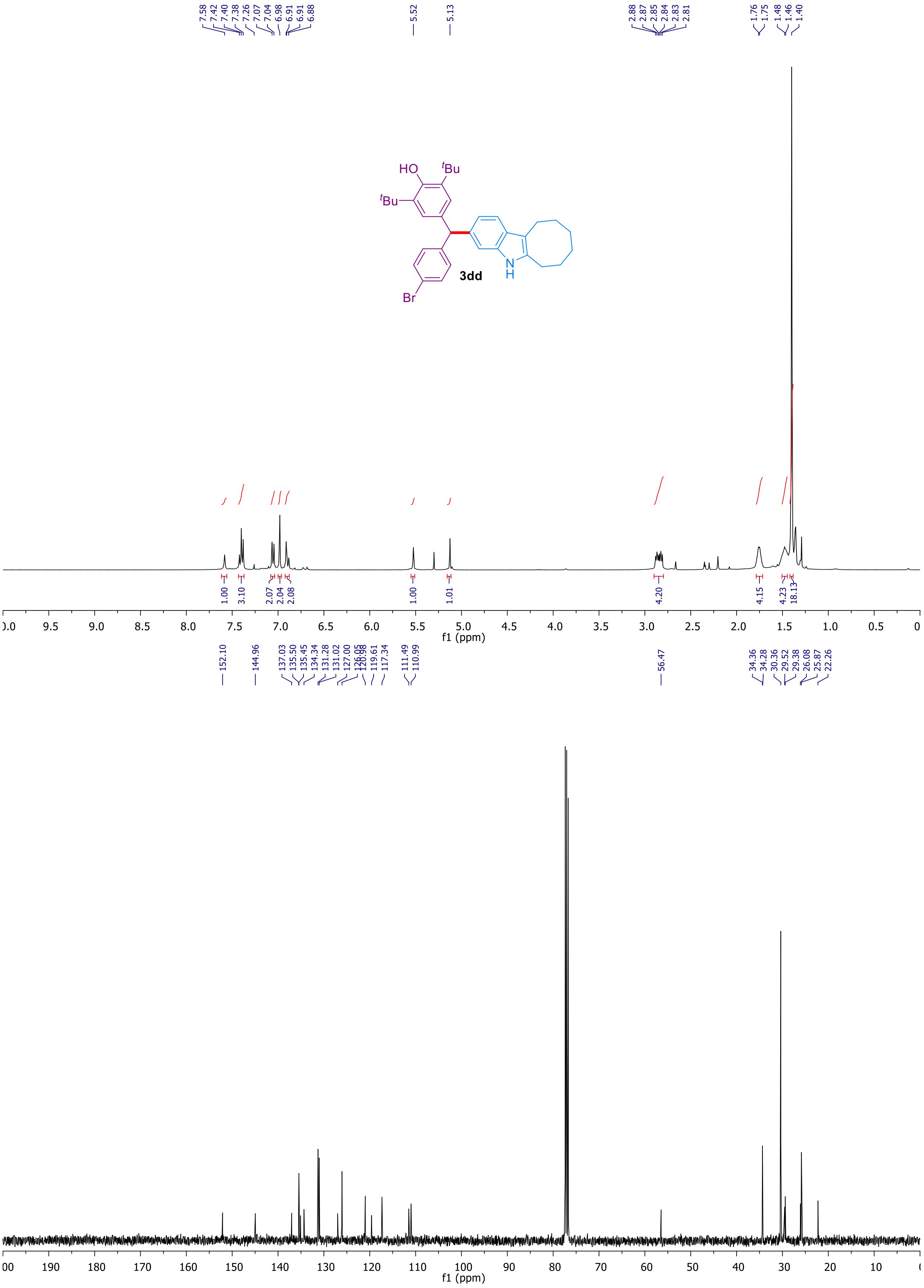

^{1}H NMR (400 MHz) and ^{13}C{^{1}H} NMR (100 MHz) spectra of **3dd** ($CDCl_3$)

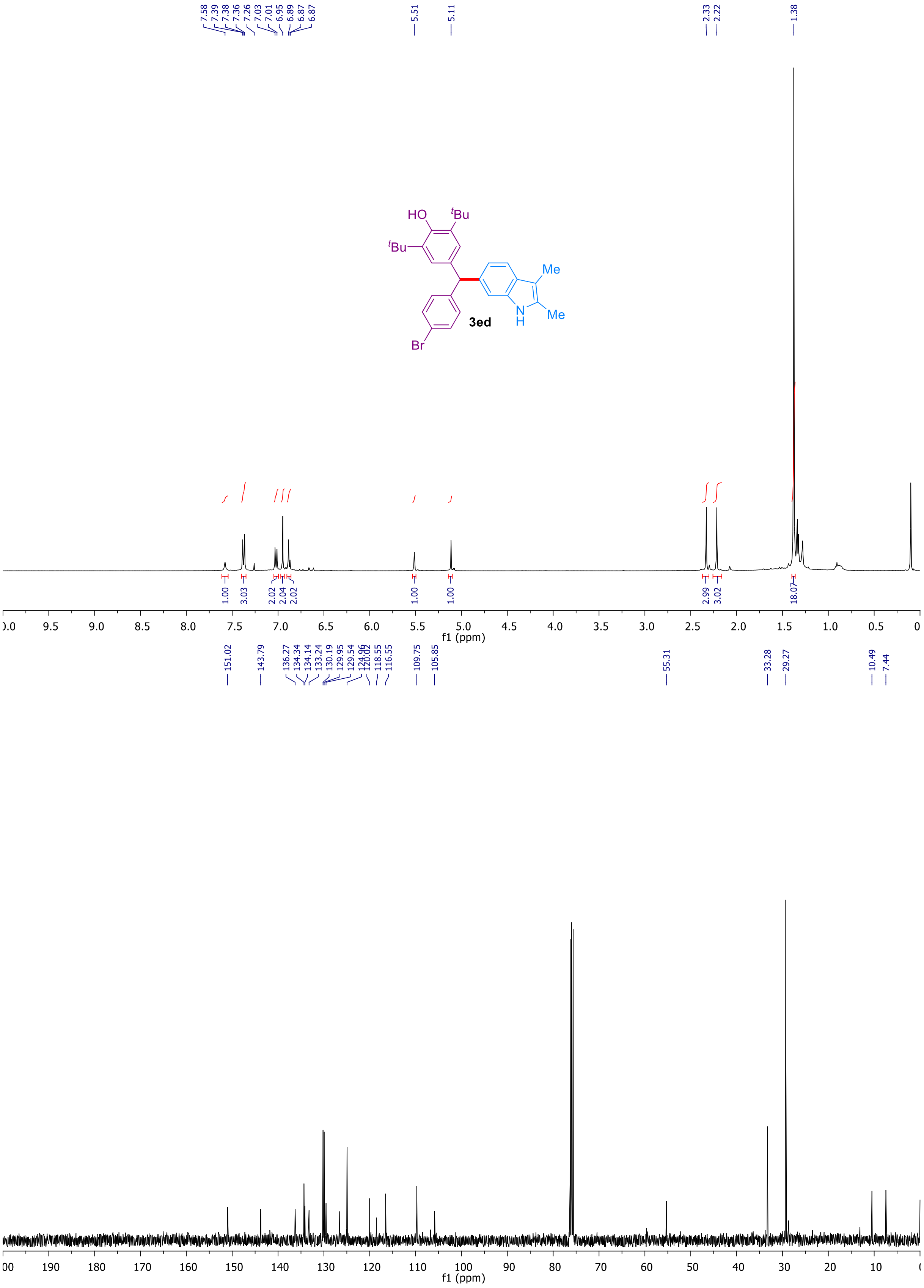

^{1}H NMR (400 MHz) and ^{13}C{^{1}H} NMR (100 MHz) spectra of **3ed** ($CDCl_3$)

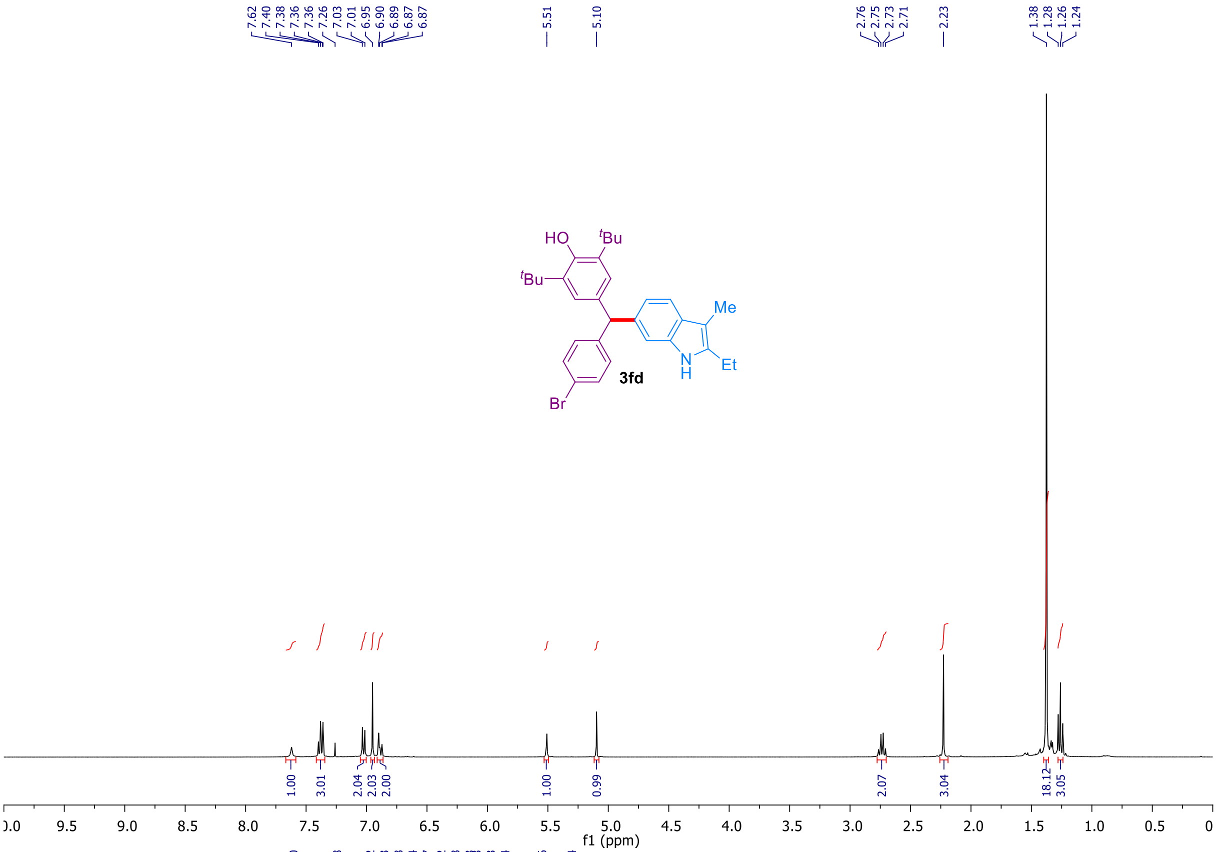

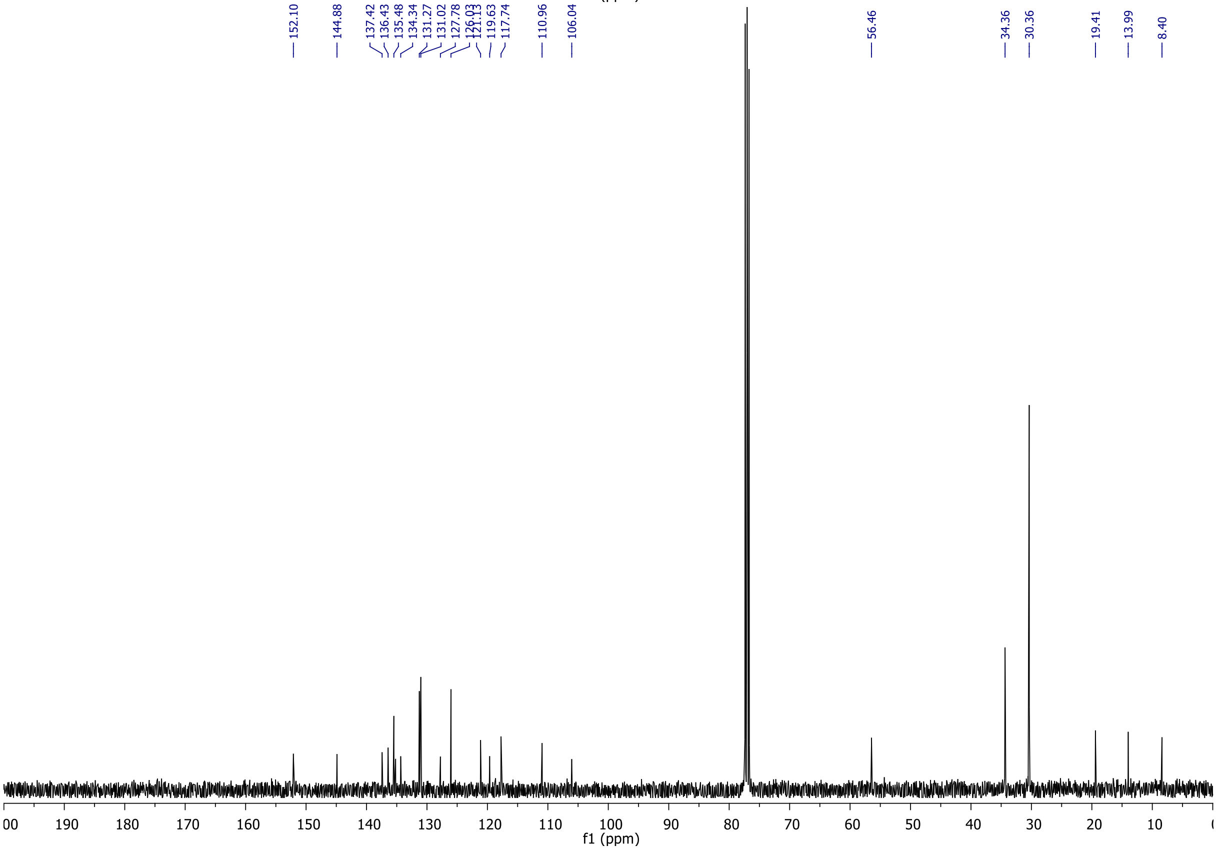

^{1}H NMR (400 MHz) and $^{13}C\{^{1}H\}$ NMR (100 MHz) spectra of **3fd** ($CDCl_3$)

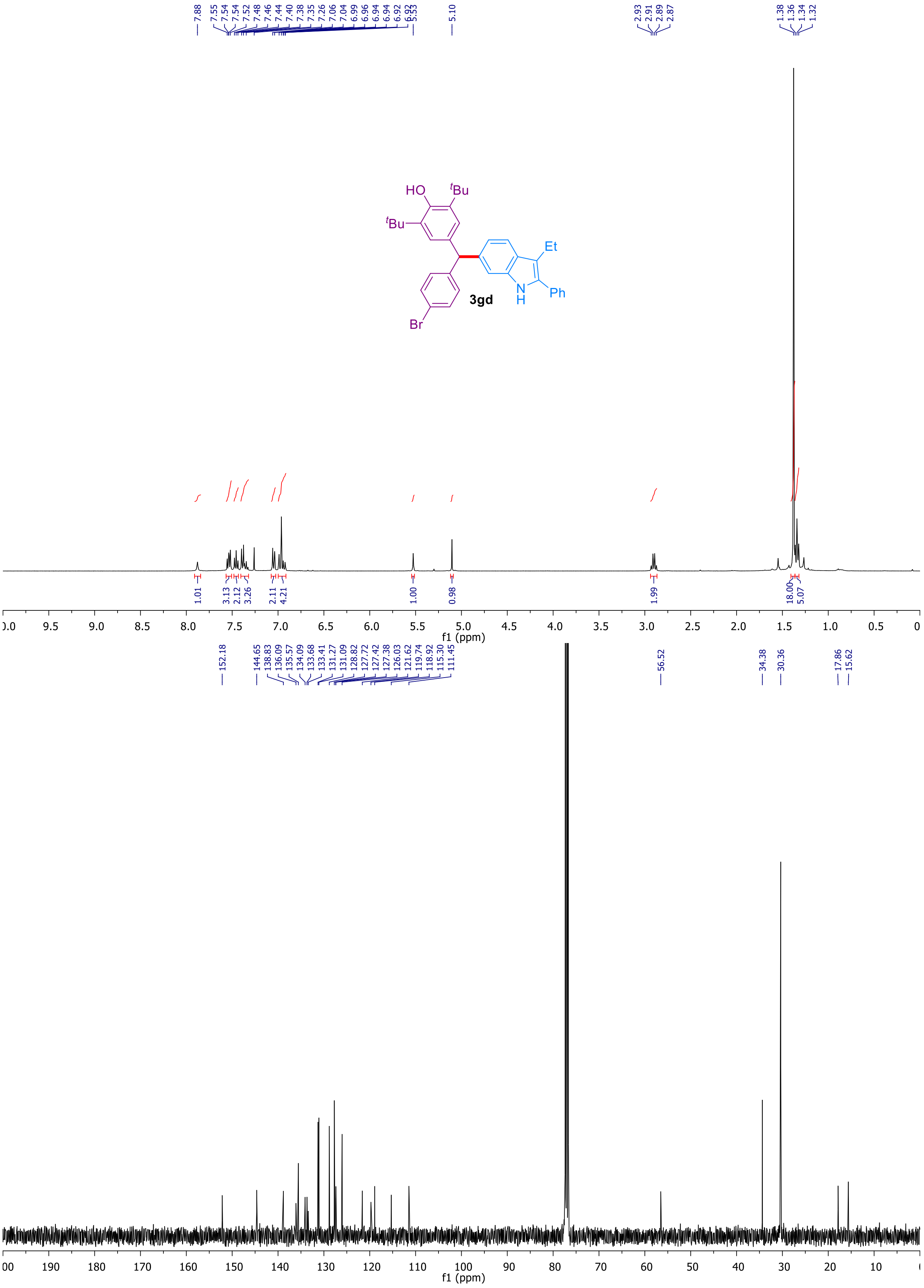

^{1}H NMR (400 MHz) and ^{13}C{^{1}H} NMR (100 MHz) spectra of **3gd** ($CDCl_3$)

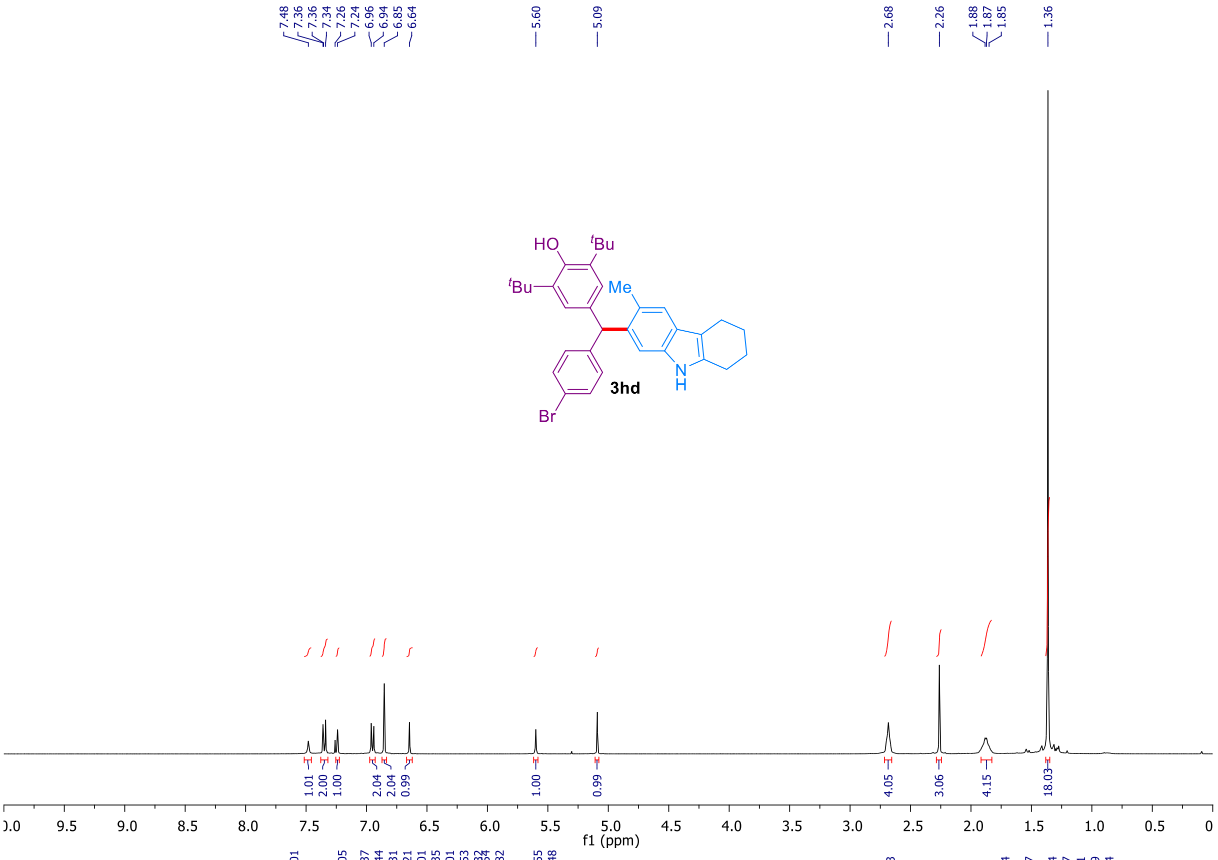

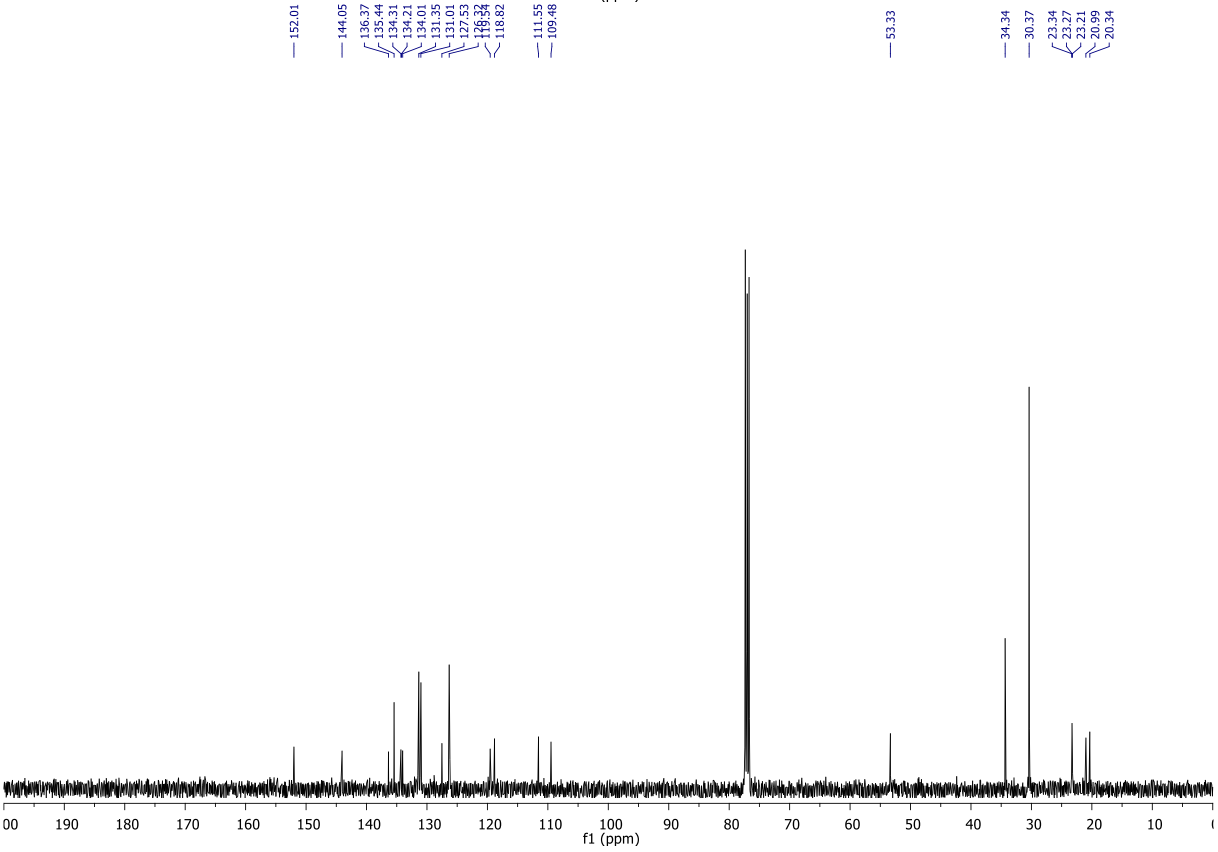

^1H NMR (400 MHz) and $^{13}C\{^1H\}$ NMR (100 MHz) spectra of **3hd** ($CDCl_3$)

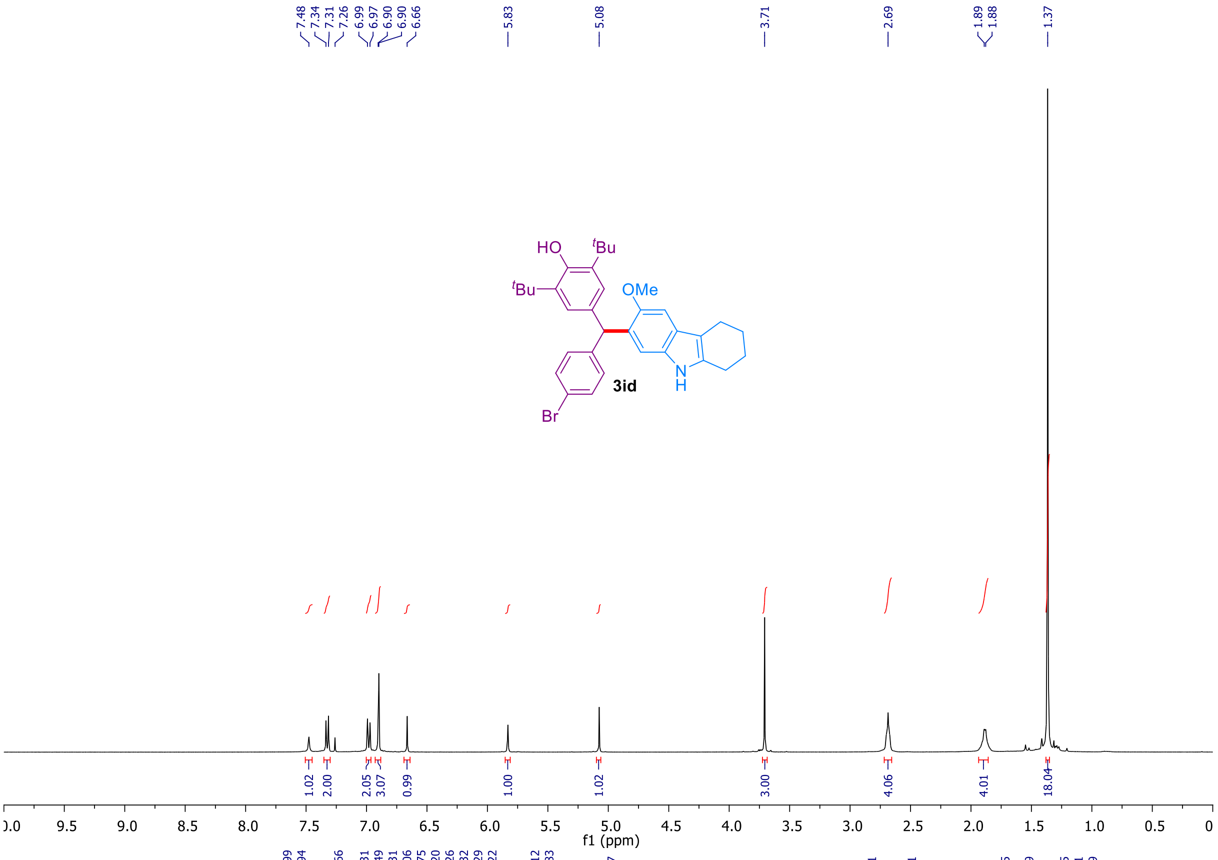

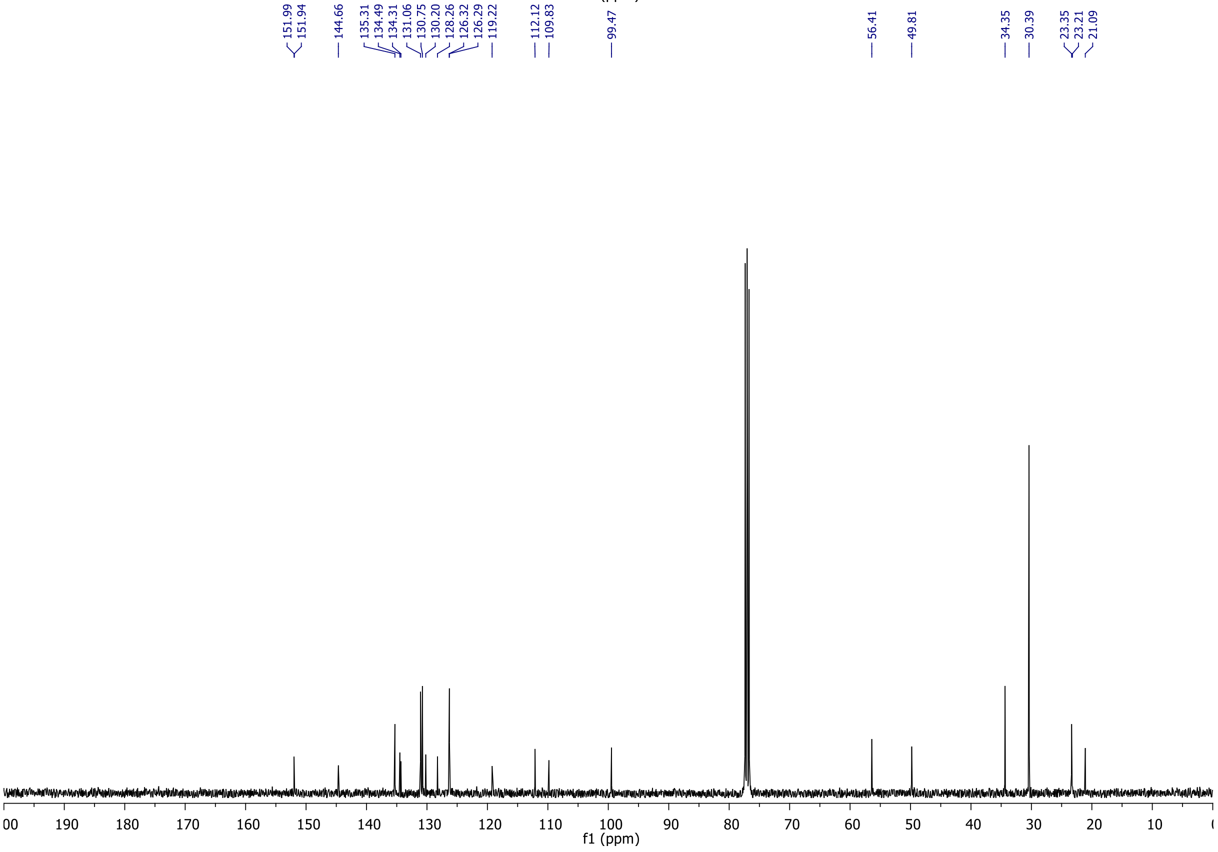

^{1}H NMR (400 MHz) and $^{13}C\{^{1}H\}$ NMR (100 MHz) spectra of **3id** ($CDCl_3$)

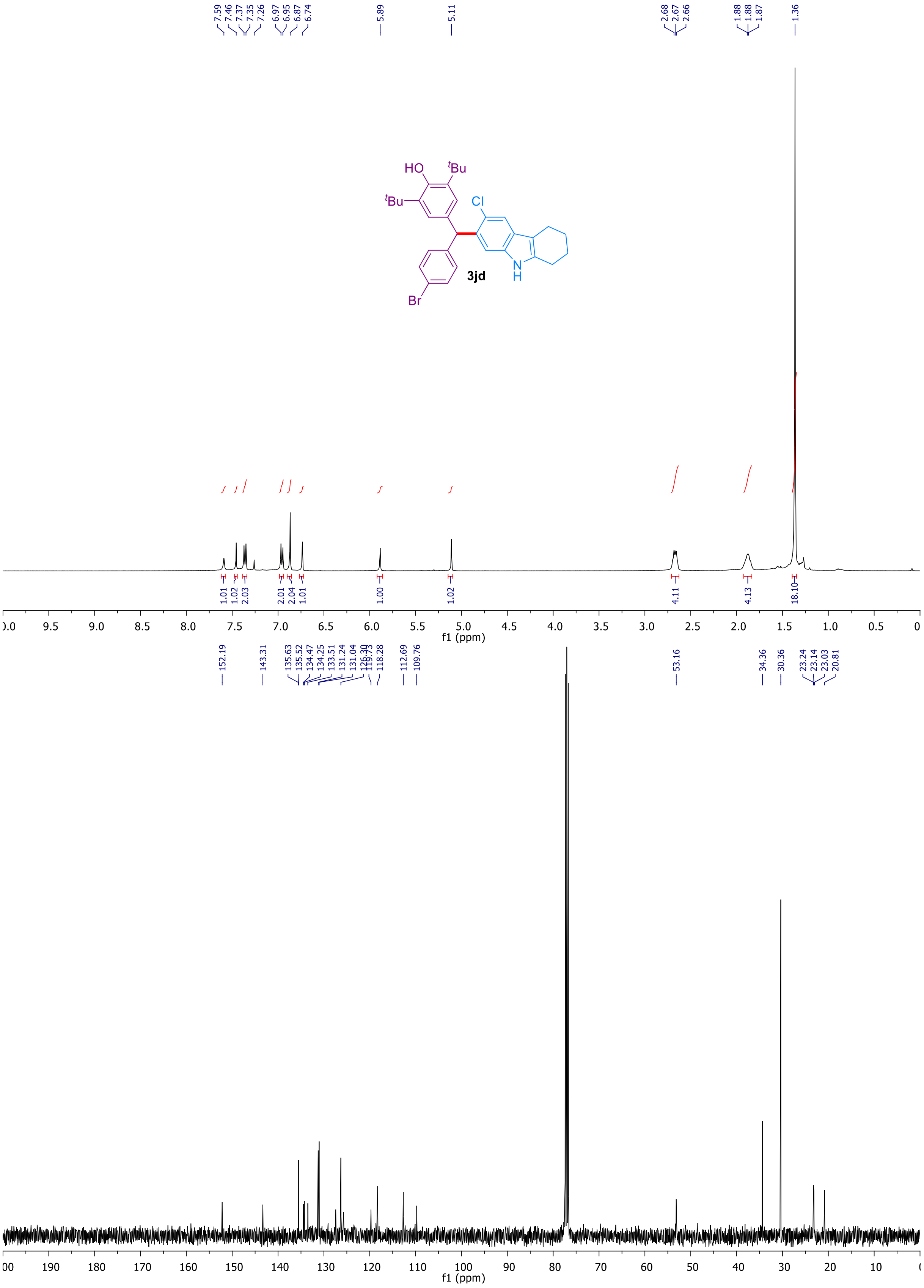

^{1}H NMR (400 MHz) and $^{13}C\{^{1}H\}$ NMR (100 MHz) spectra of **3jd** ($CDCl_3$)

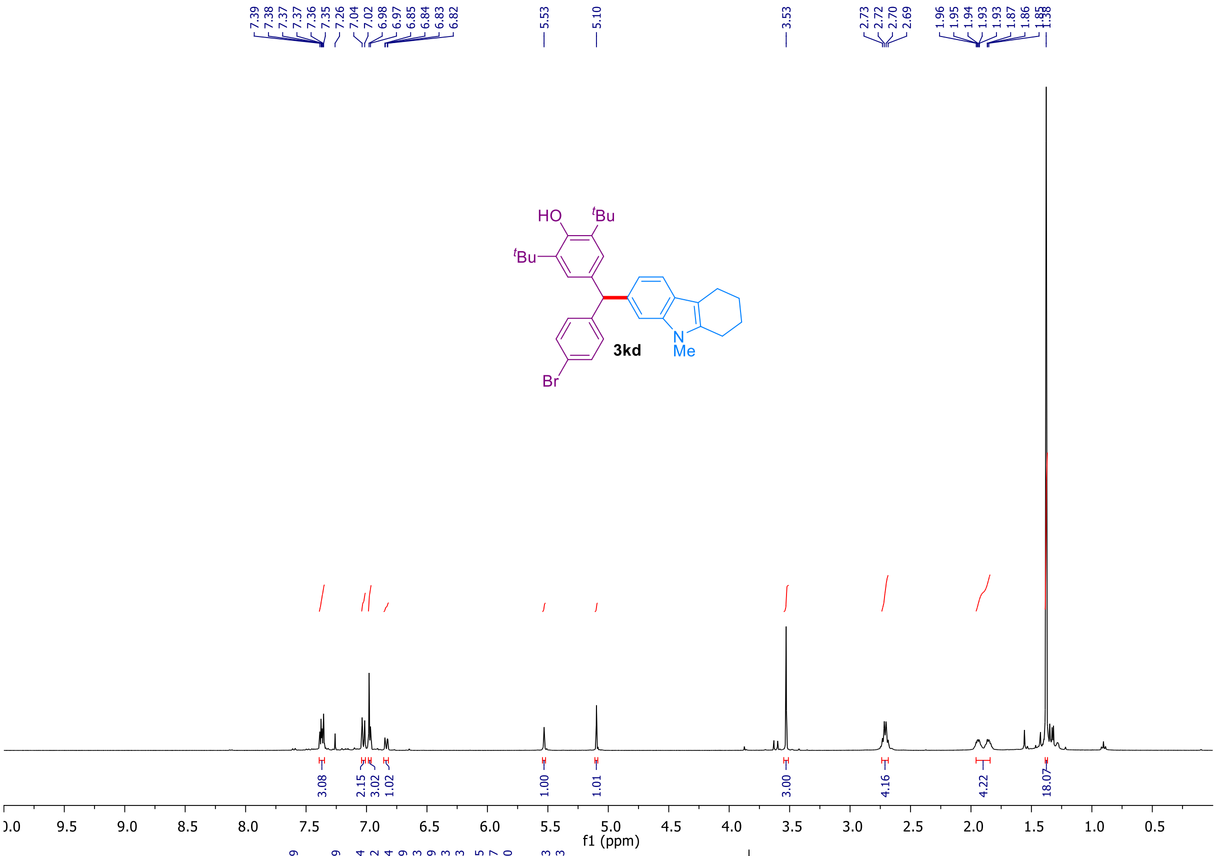

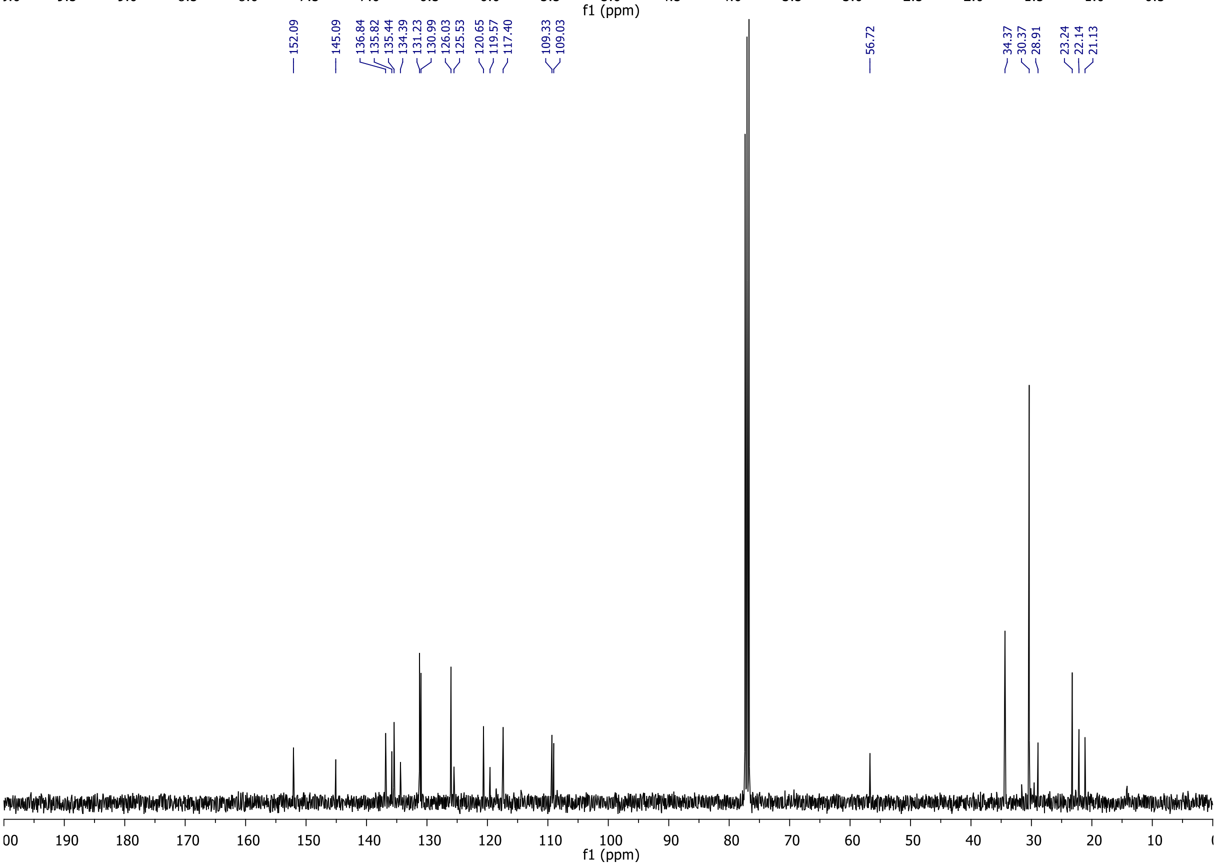

^{1}H NMR (400 MHz) and ^{13}C{^{1}H} NMR (100 MHz) spectra of **3kd** ($CDCl_3$)

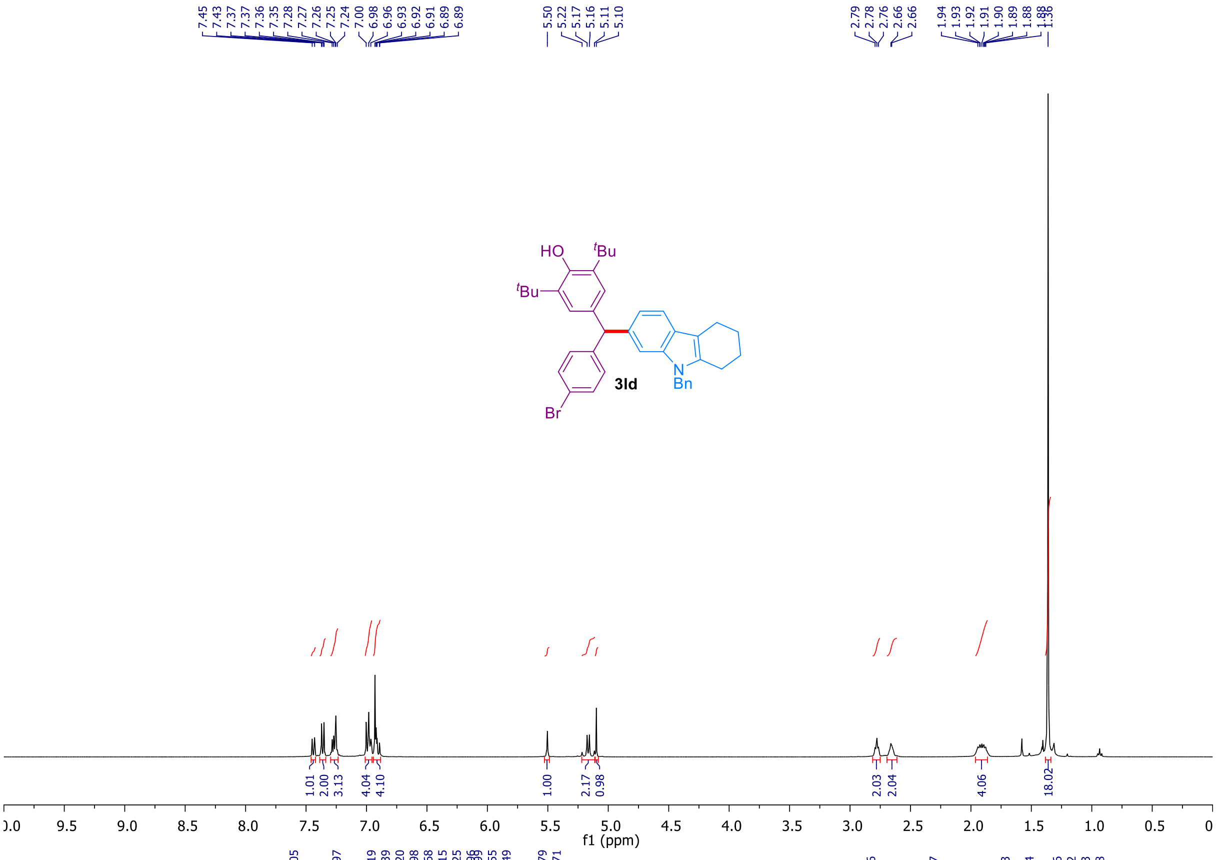

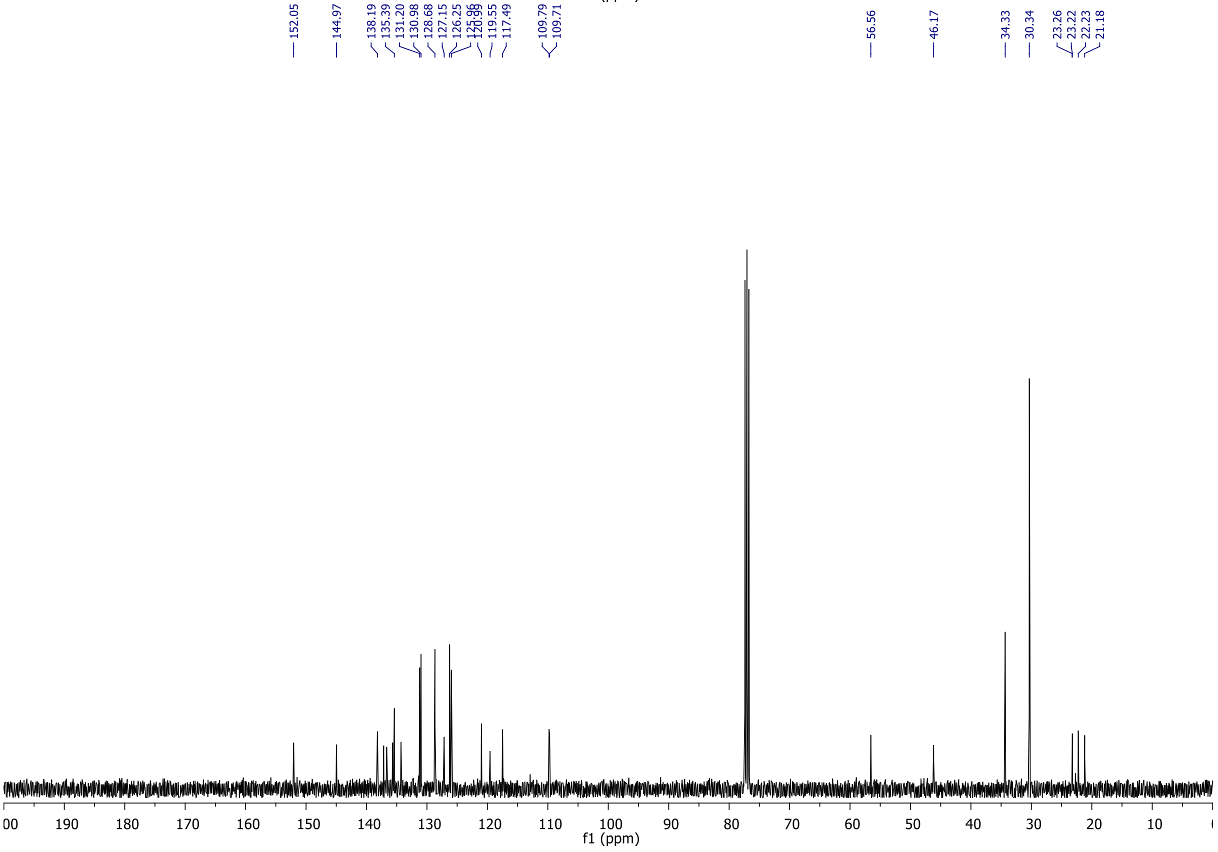

^{1}H NMR (400 MHz) and ^{13}C{^{1}H} NMR (100 MHz) spectra of **3ld** ($CDCl_3$)

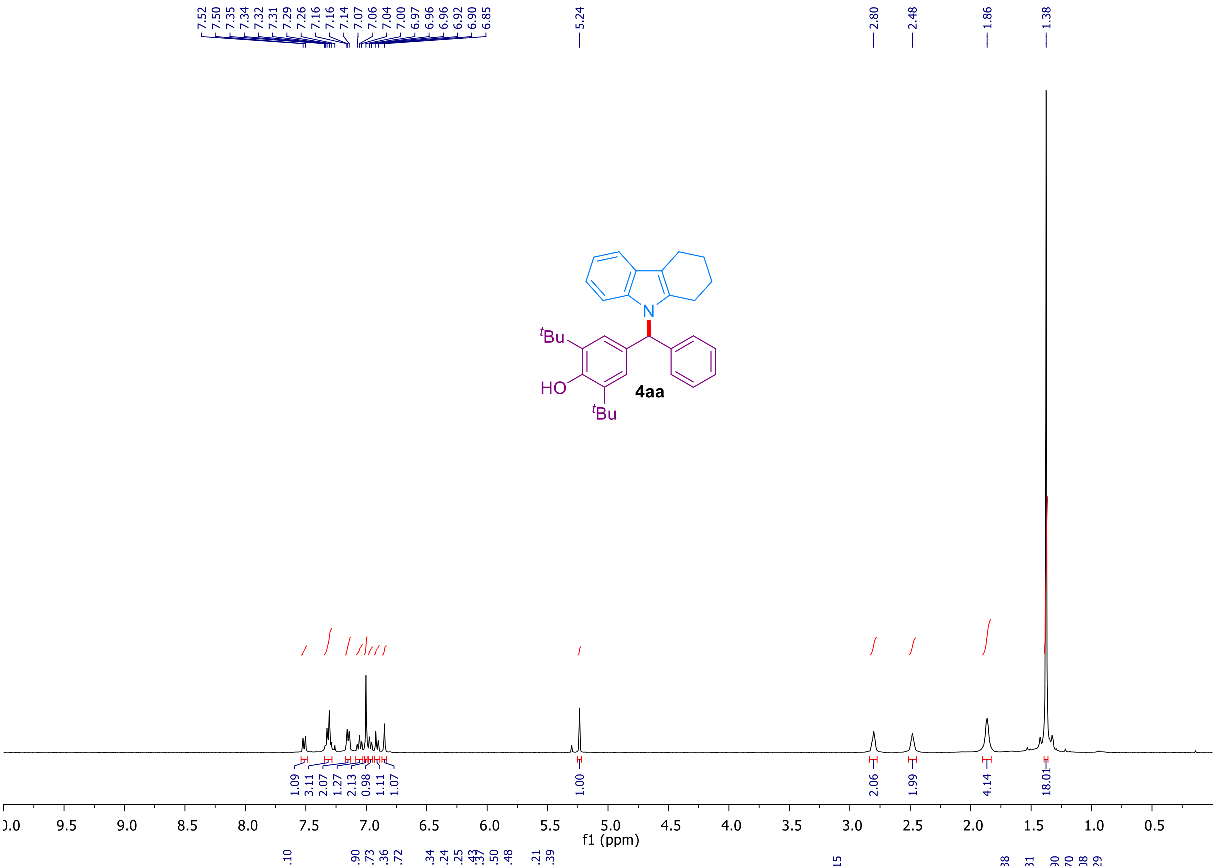

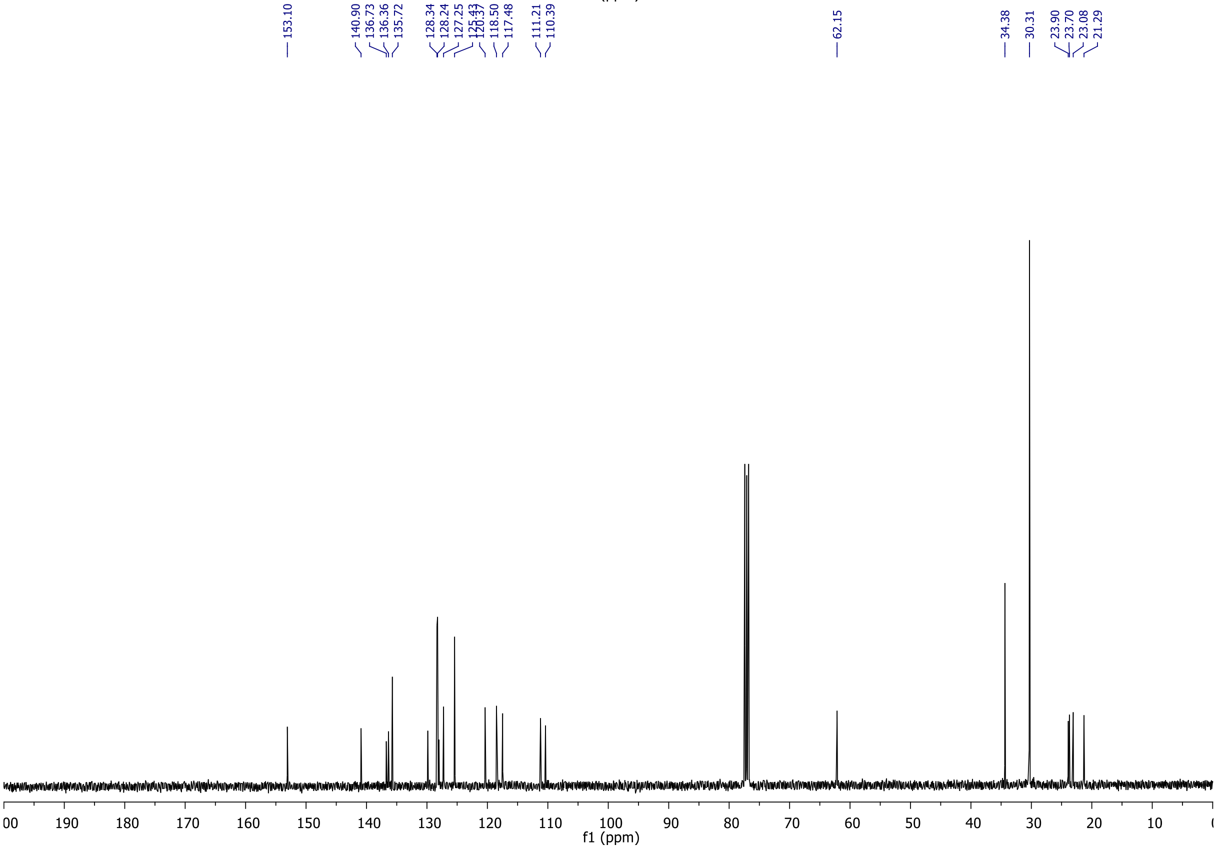

^{1}H NMR (400 MHz) and $^{13}C\{^{1}H\}$ NMR (100 MHz) spectra of **4aa** ($CDCl_3$)

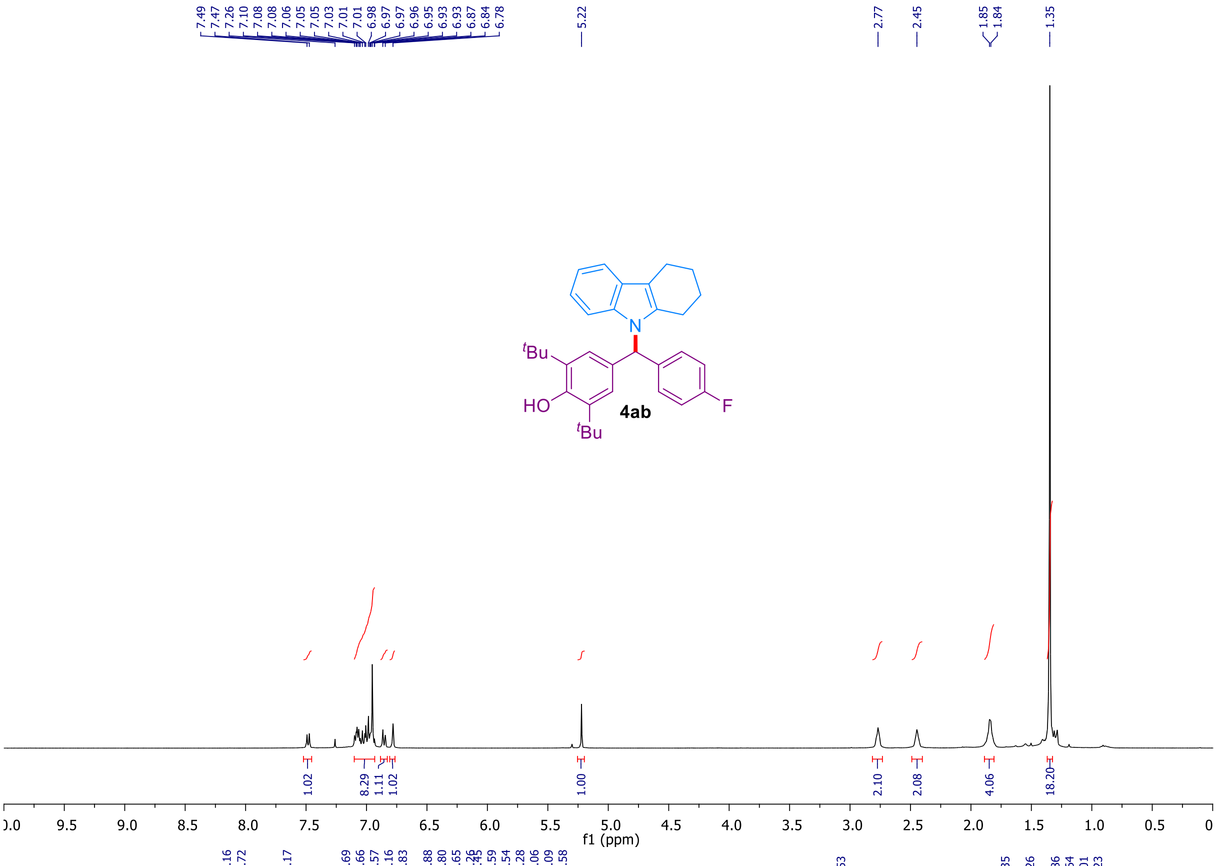

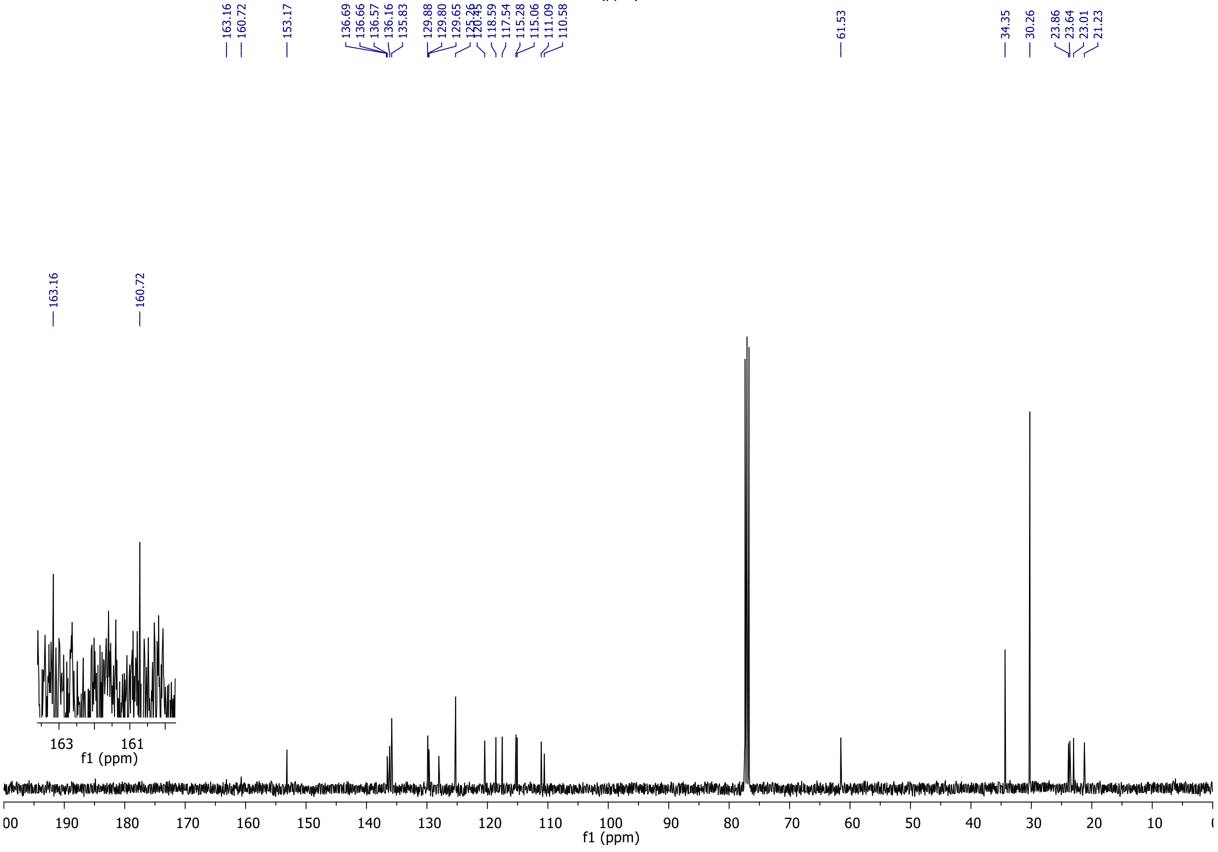

^{1}H NMR (400 MHz) and ^{13}C{^{1}H} NMR (100 MHz) spectra of **4ab** ($CDCl_3$)

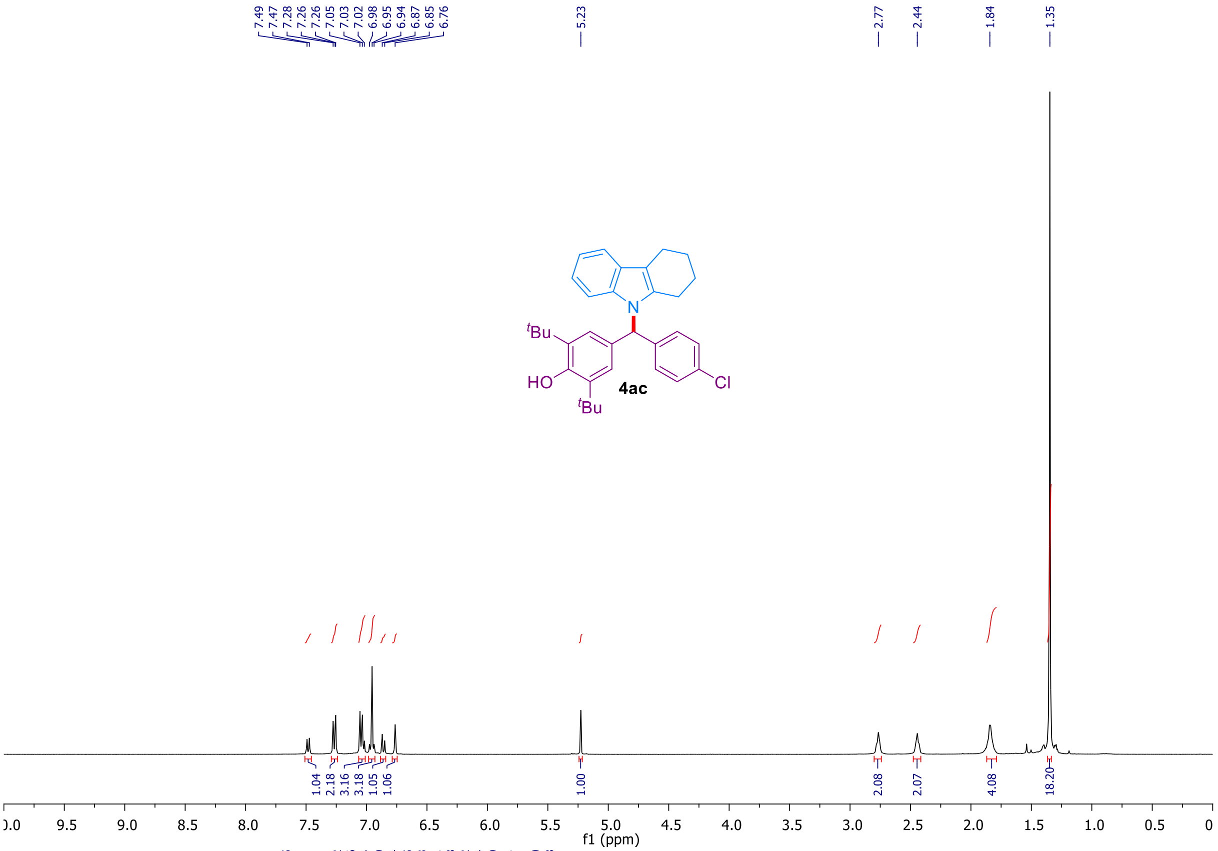

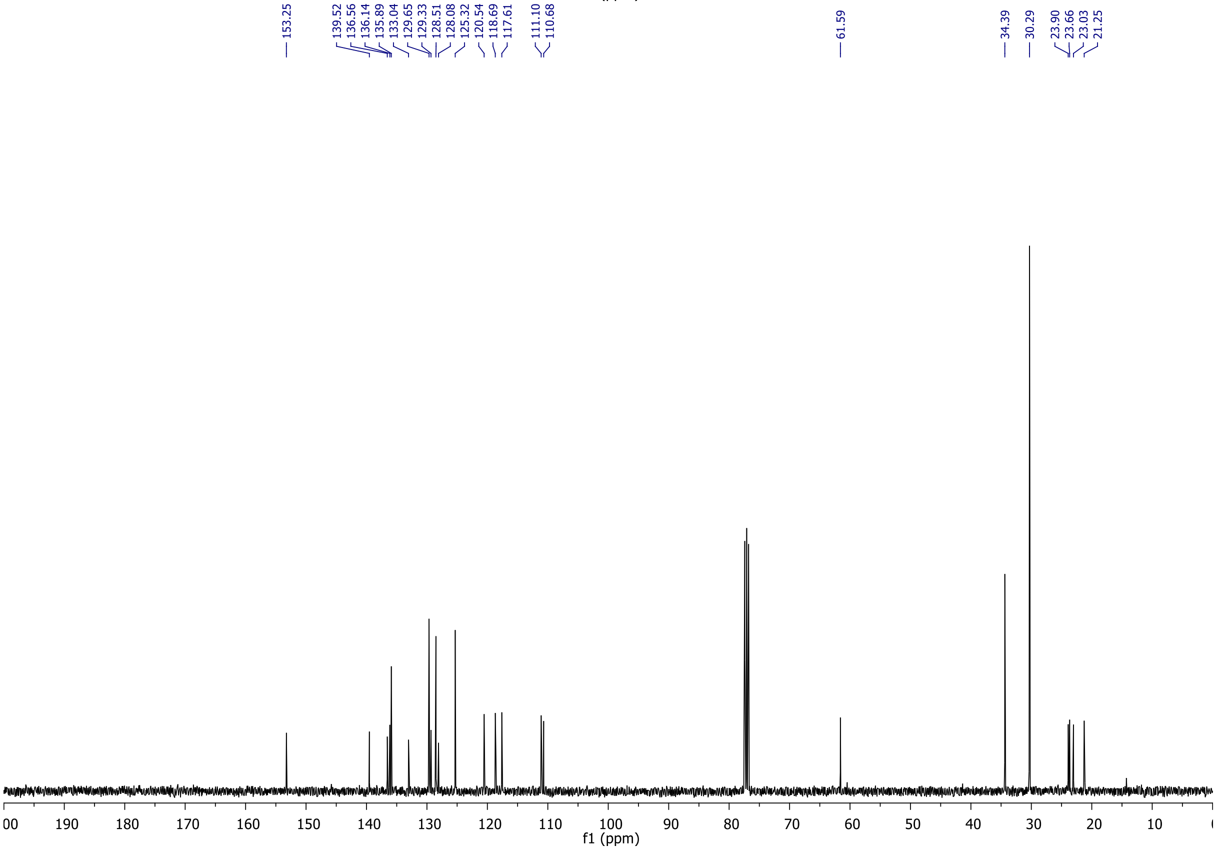

^{1}H NMR (400 MHz) and ^{13}C{^{1}H} NMR (100 MHz) spectra of **4ac** ($CDCl_3$)

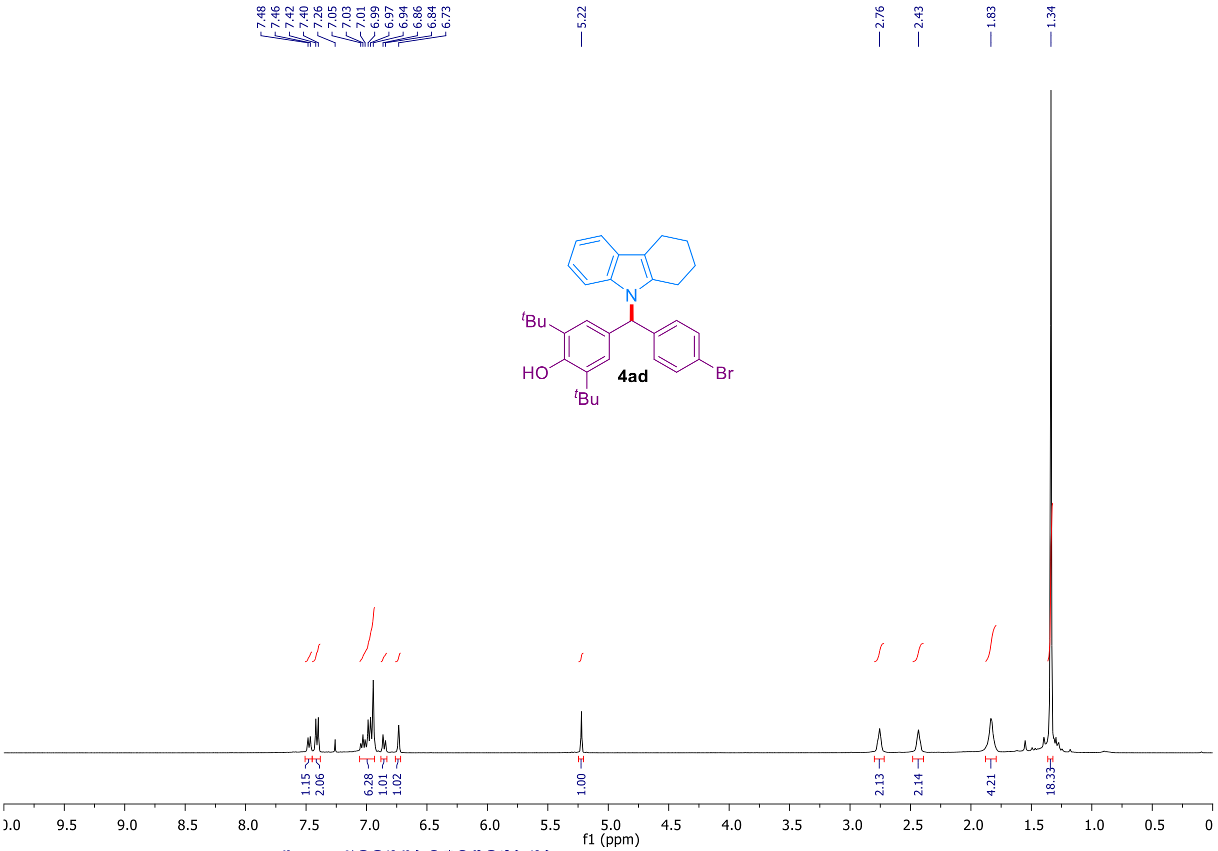

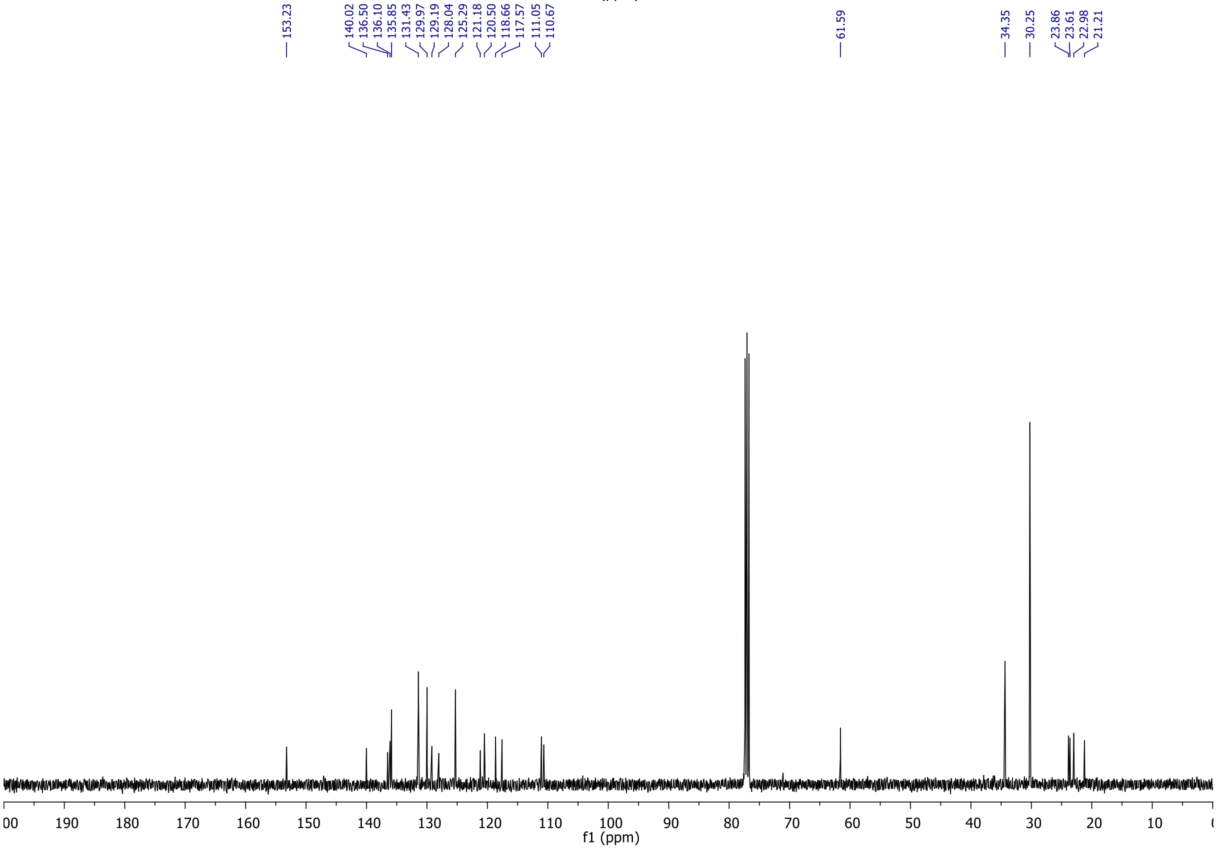

^{1}H NMR (400 MHz) and ^{13}C{^{1}H} NMR (100 MHz) spectra of **4ad** ($CDCl_3$)

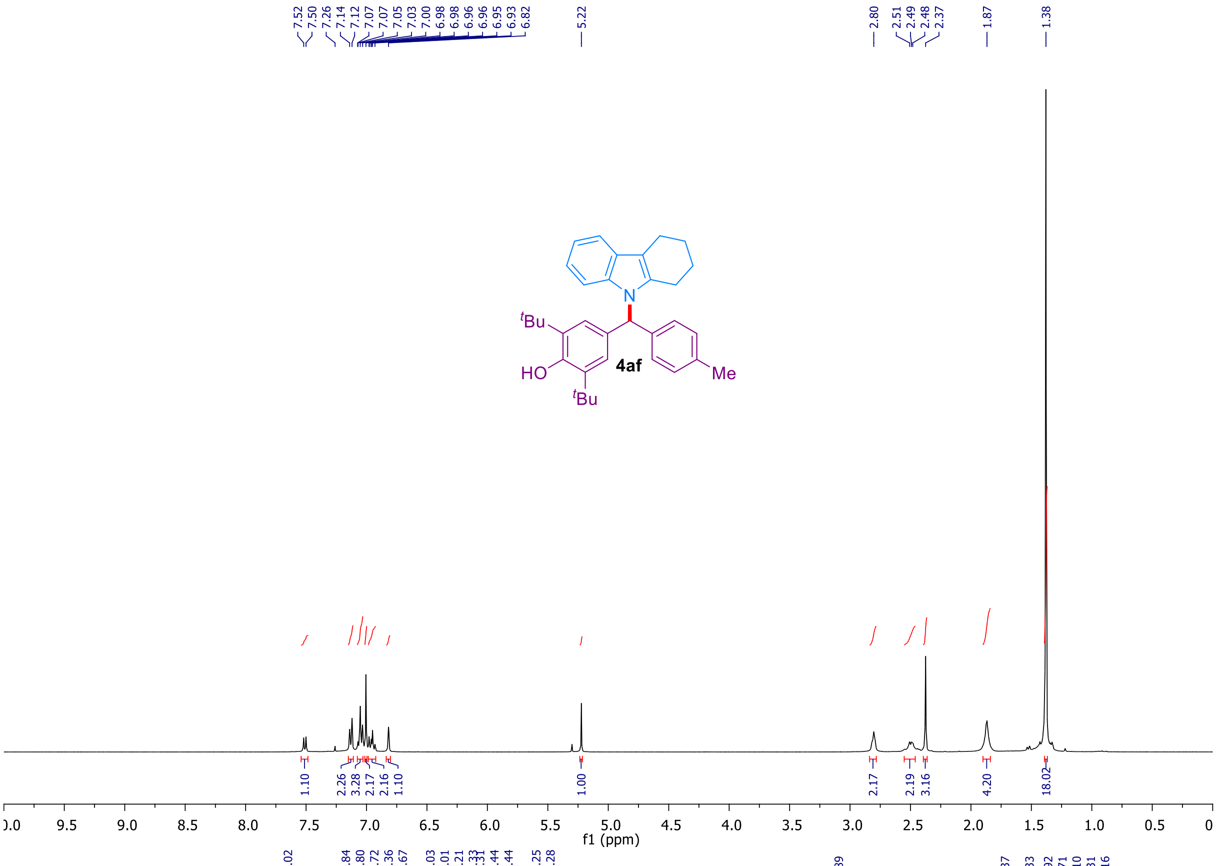

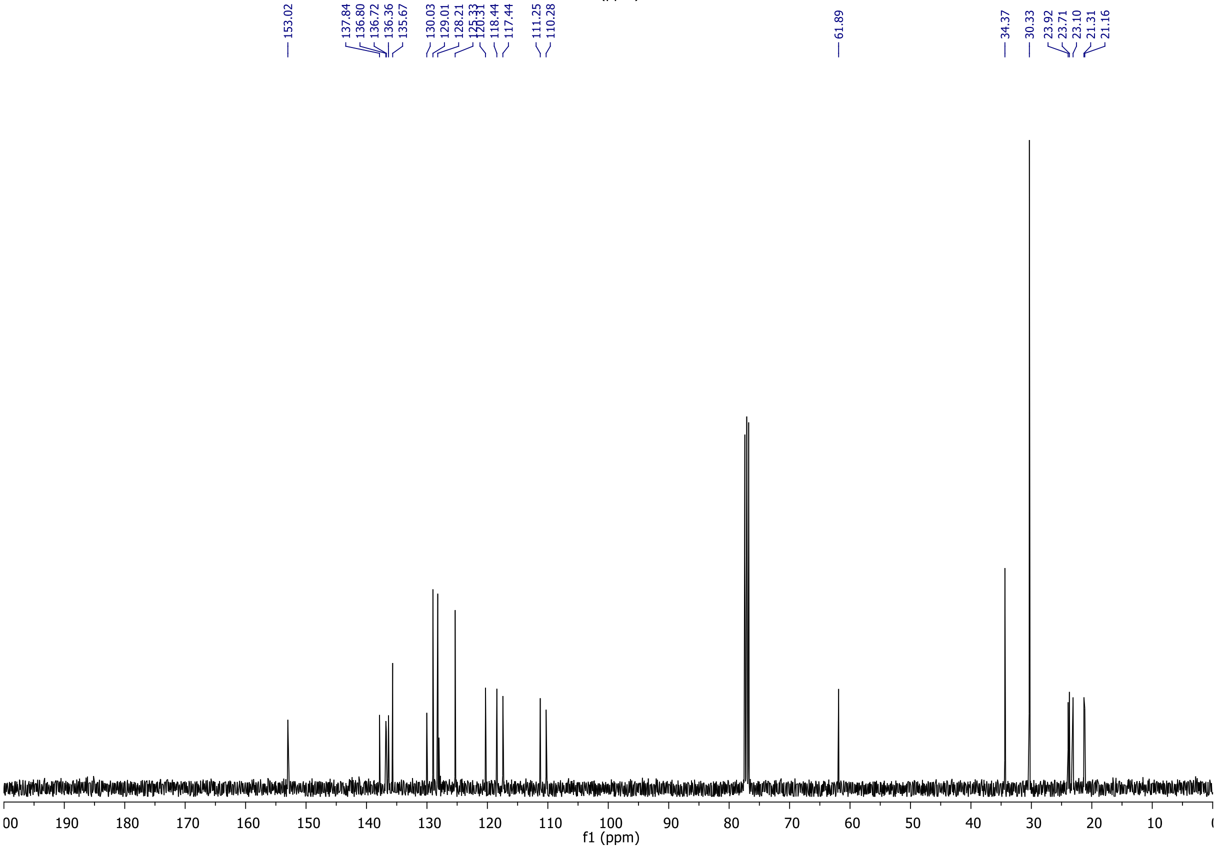

^{1}H NMR (400 MHz) and $^{13}C\{^{1}H\}$ NMR (100 MHz) spectra of **4af** ($CDCl_3$)

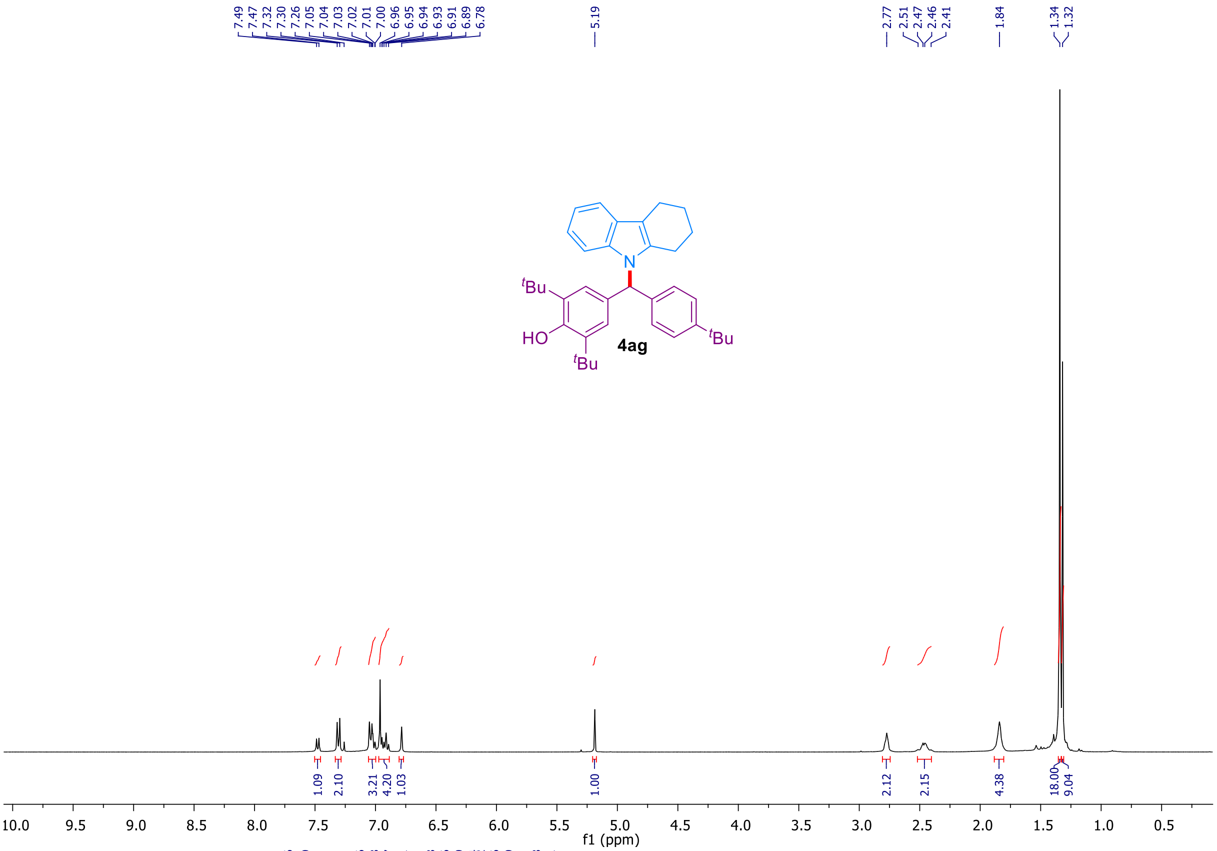

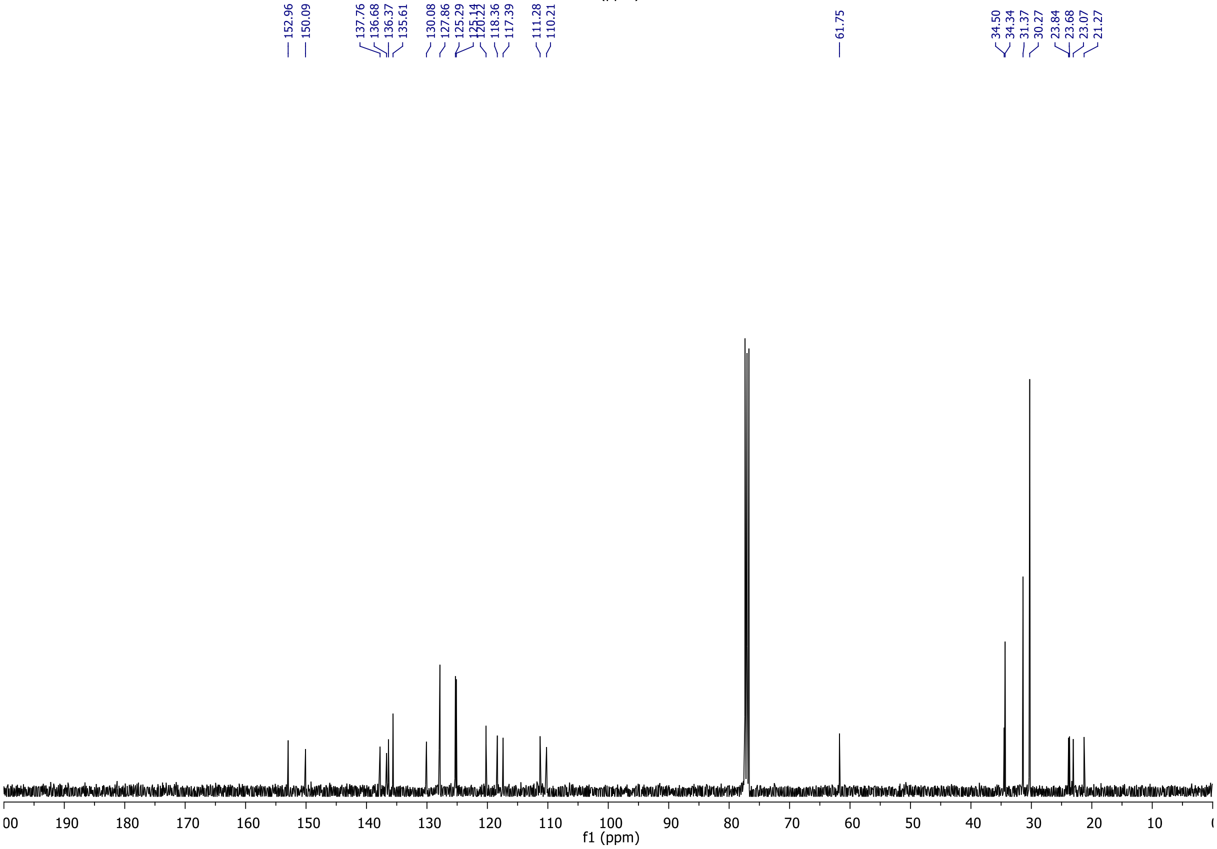

^{1}H NMR (400 MHz) and ^{13}C{^{1}H} NMR (100 MHz) spectra of **4ag** ($CDCl_3$)

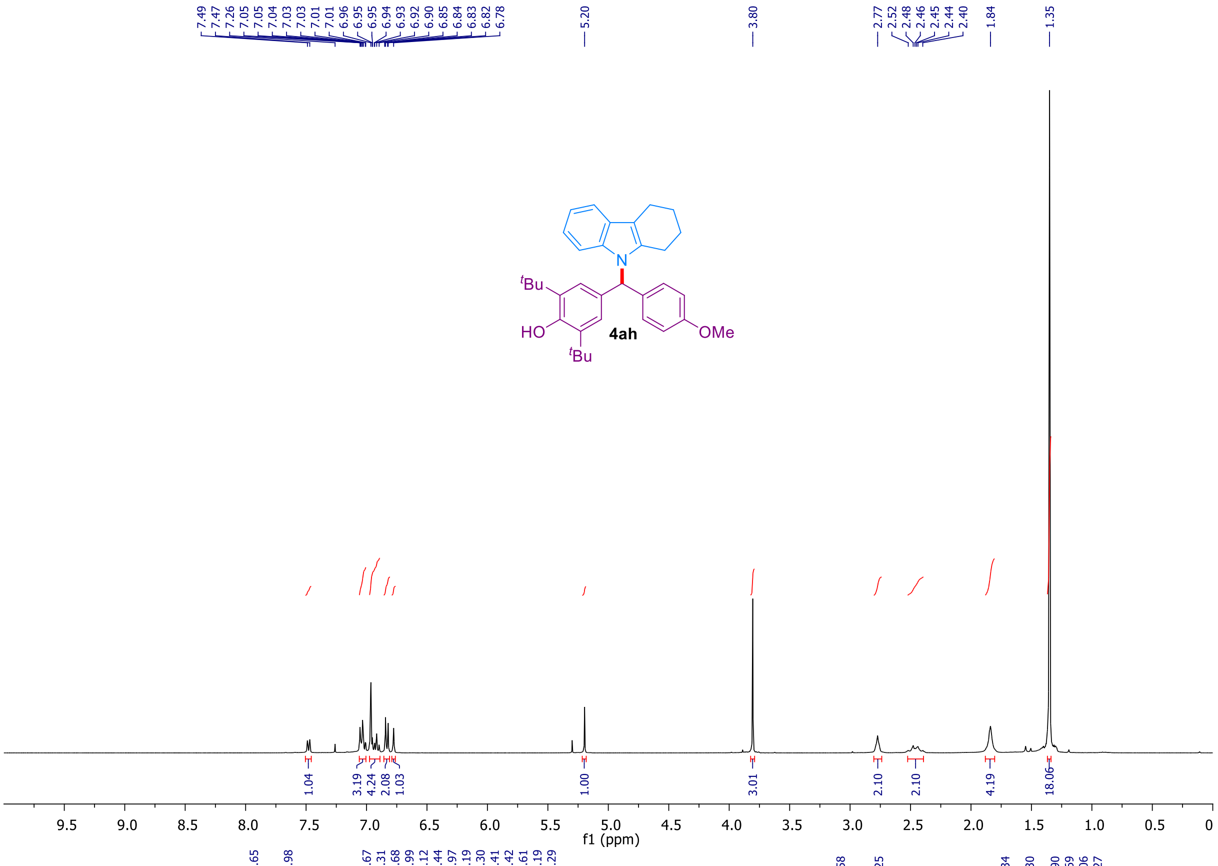

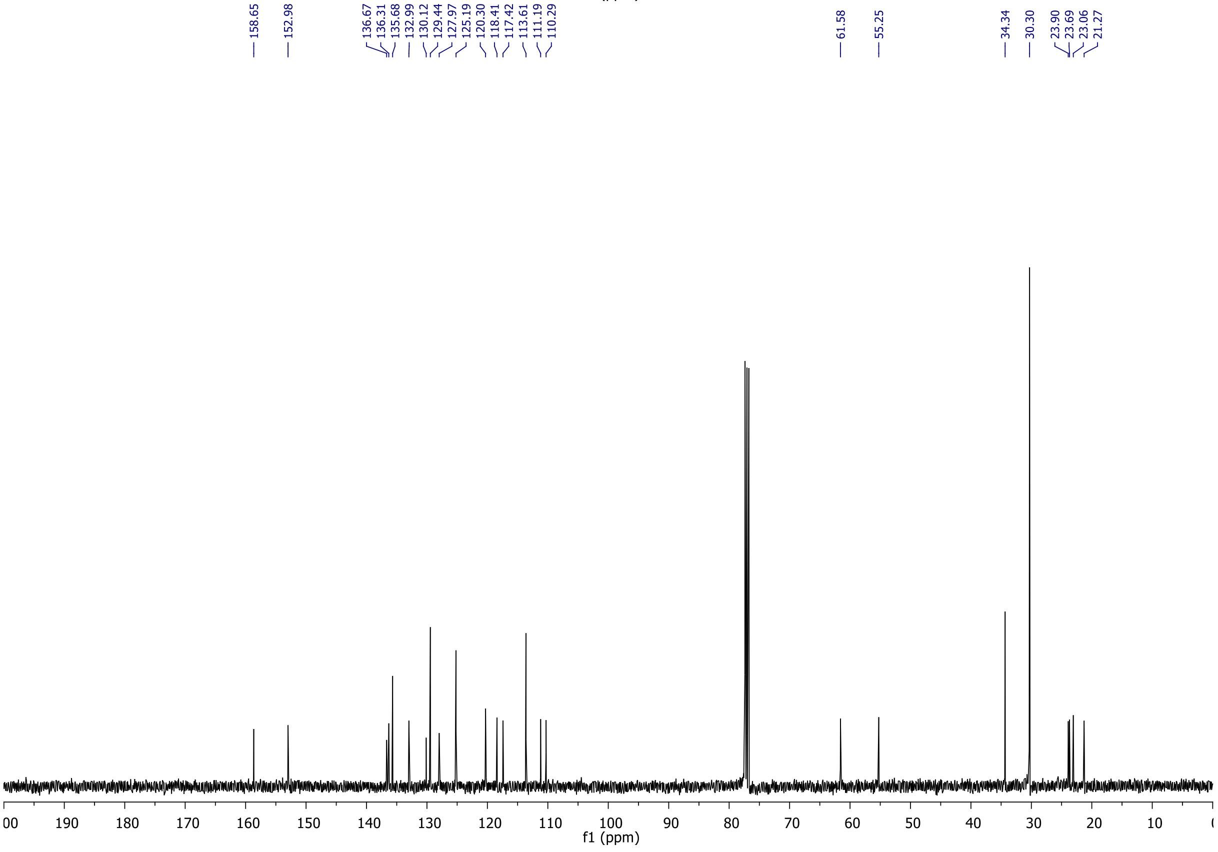

^{1}H NMR (400 MHz) and ^{13}C{^{1}H} NMR (100 MHz) spectra of **4ah** ($CDCl_3$)

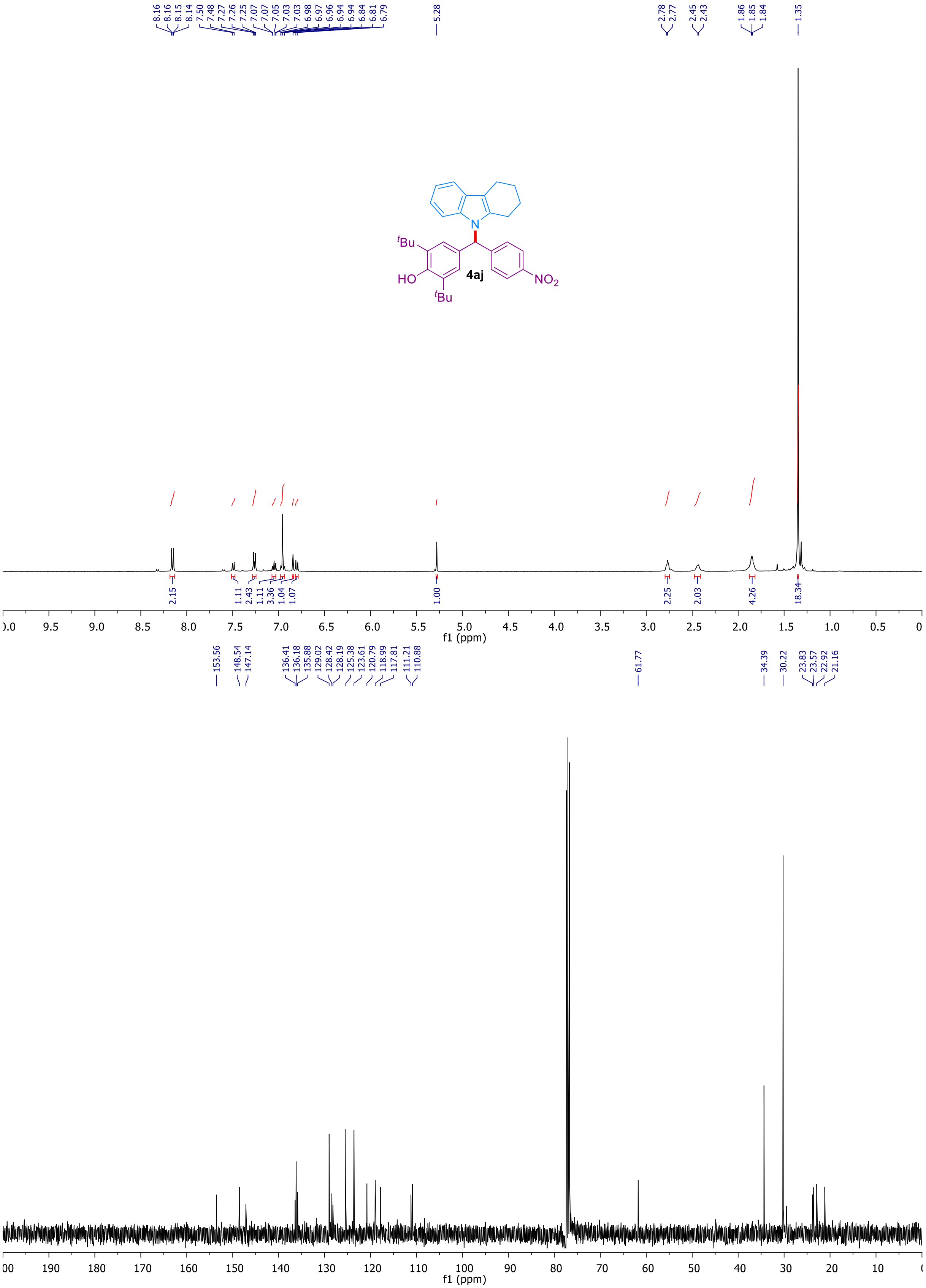

^1H NMR (400 MHz) and $^{13}C\{^1H\}$ NMR (100 MHz) spectra of **4aj** ($CDCl_3$)

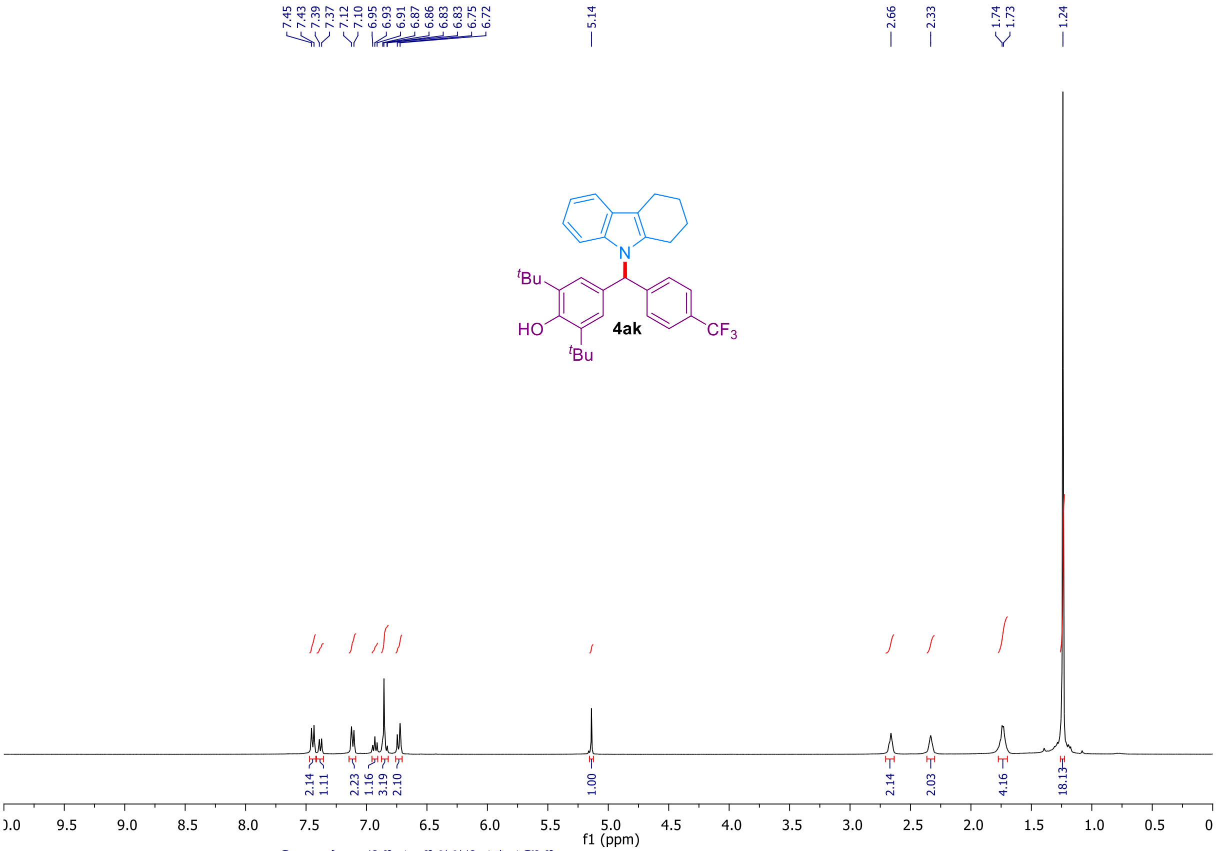

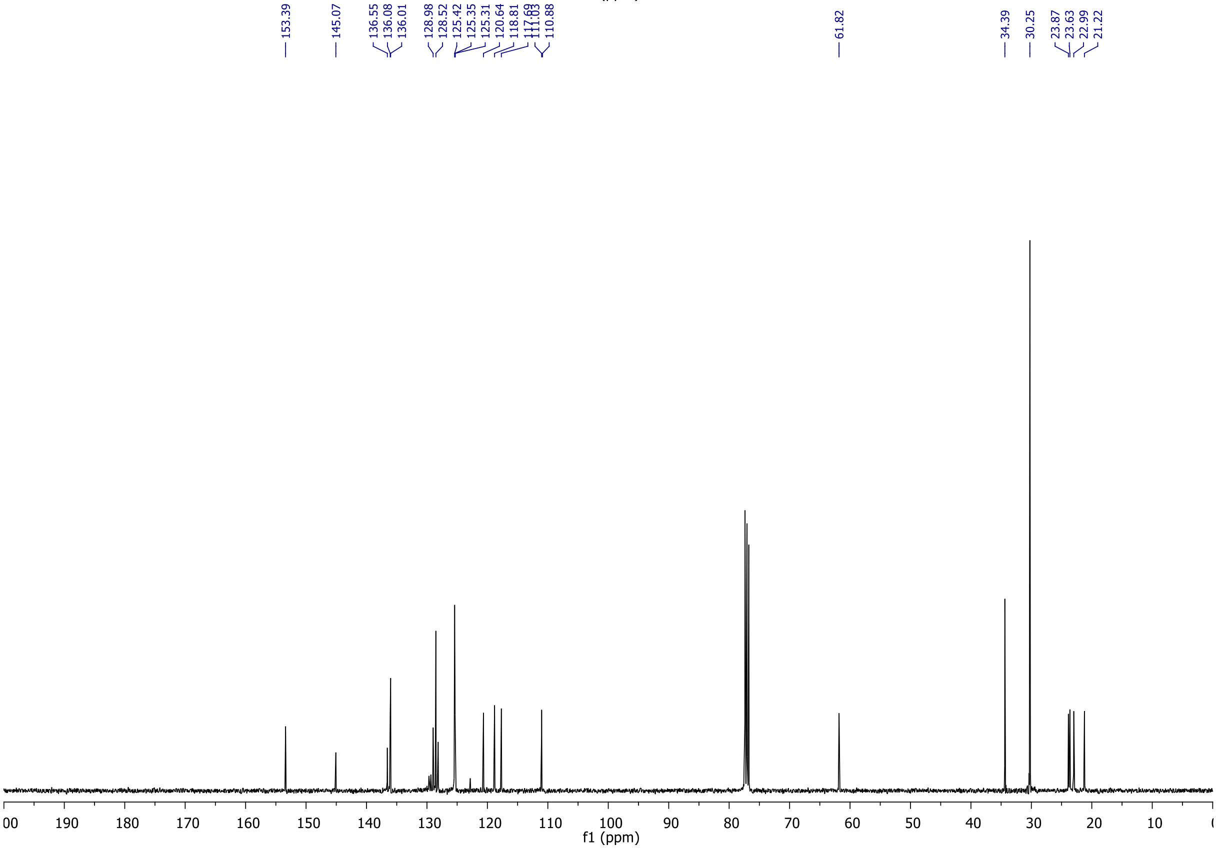

^{1}H NMR (400 MHz) and $^{13}C\{^{1}H\}$ NMR (100 MHz) spectra of **4ak** ($CDCl_3$)

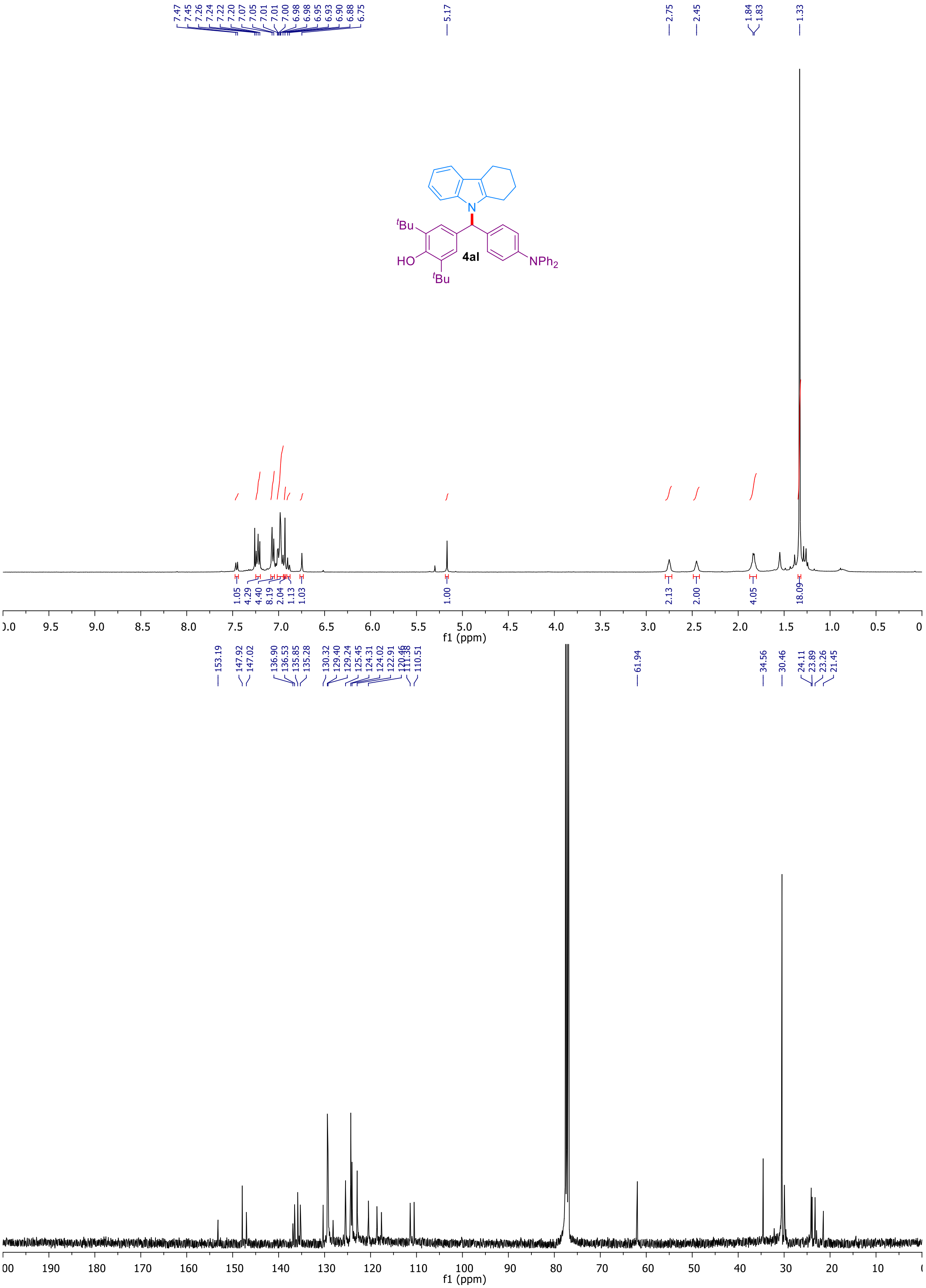

^1H NMR (400 MHz) and $^{13}C\{^1H\}$ NMR (100 MHz) spectra of **4al** ($CDCl_3$)

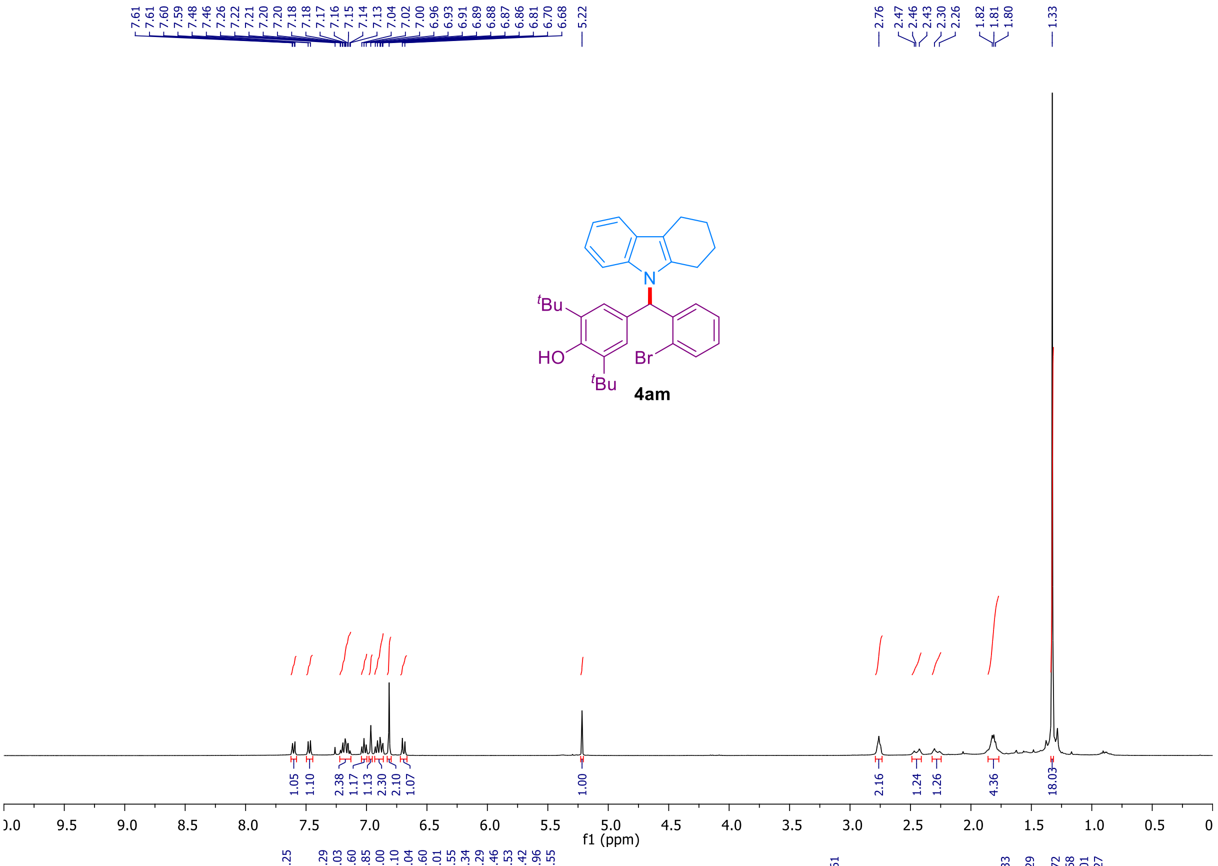

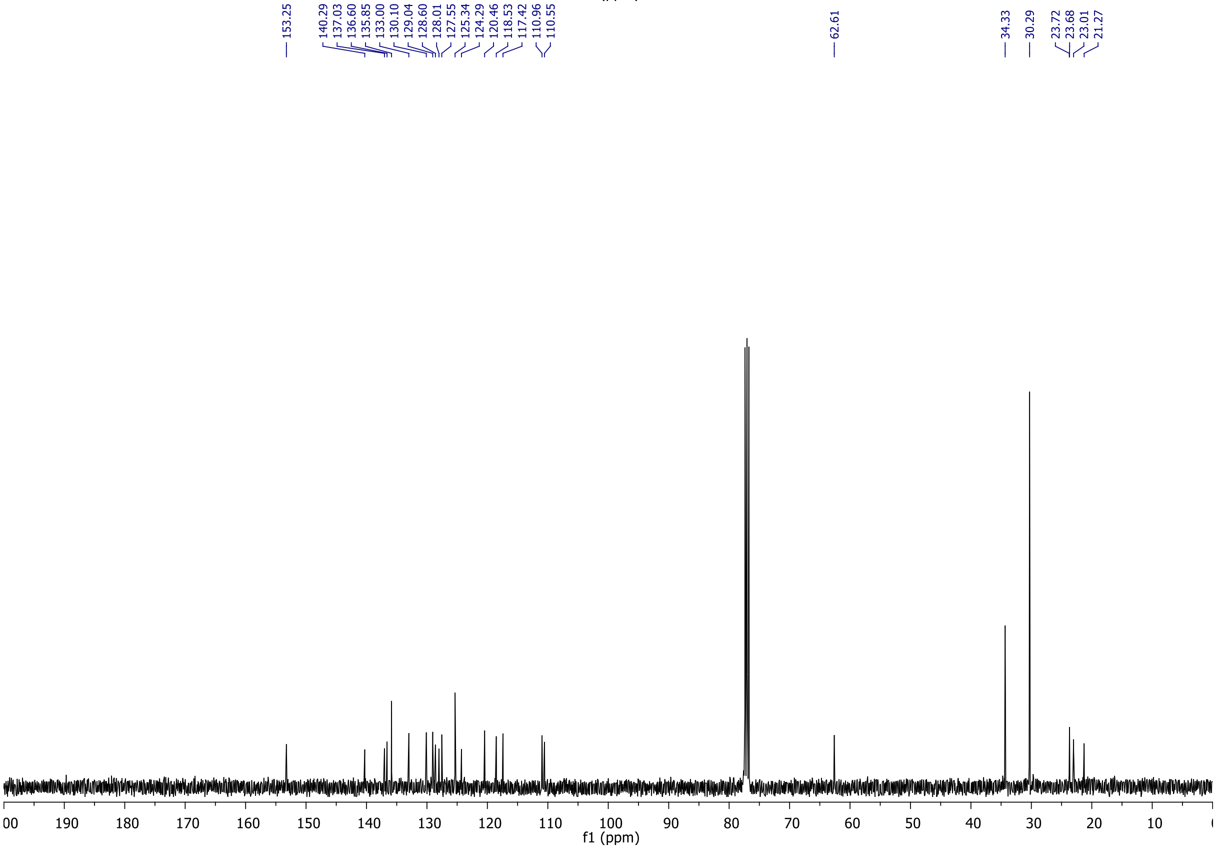

^{1}H NMR (400 MHz) and $^{13}C\{^{1}H\}$ NMR (100 MHz) spectra of **4am** ($CDCl_3$)

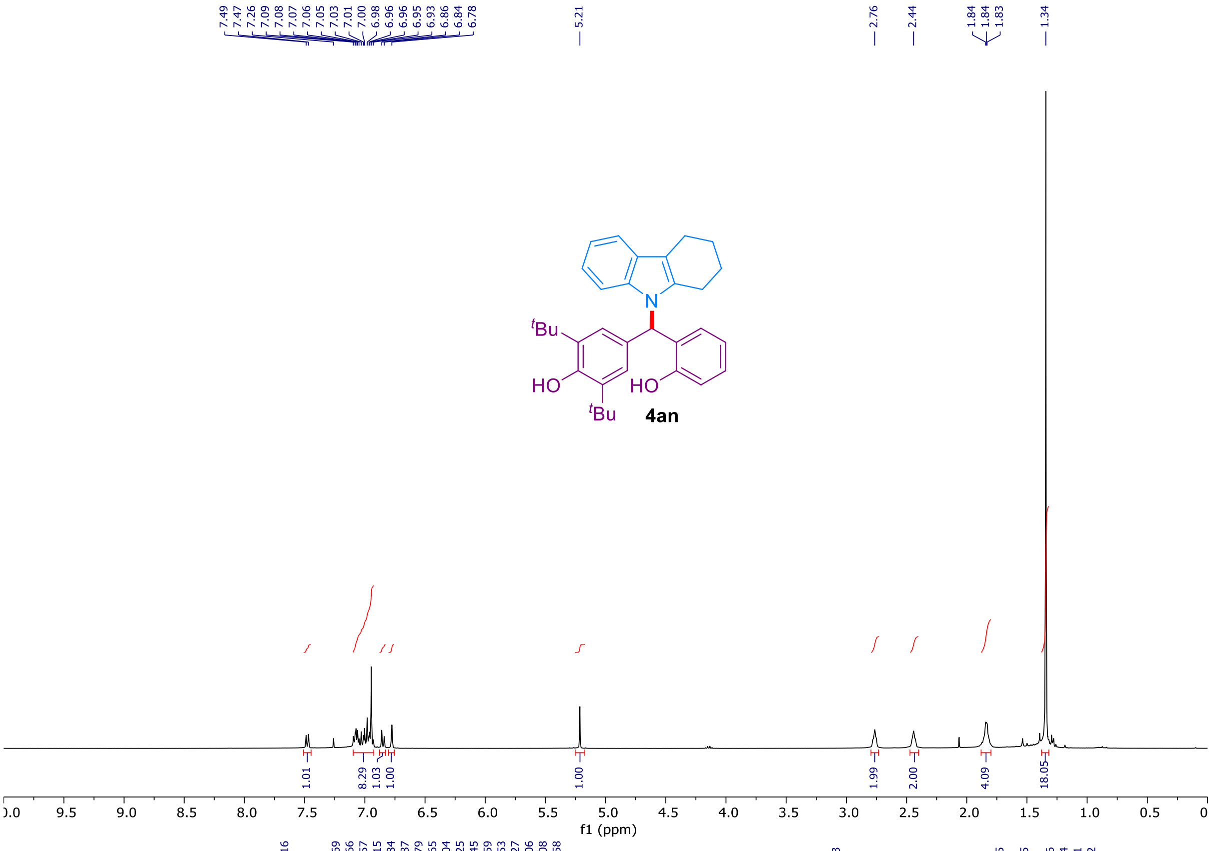

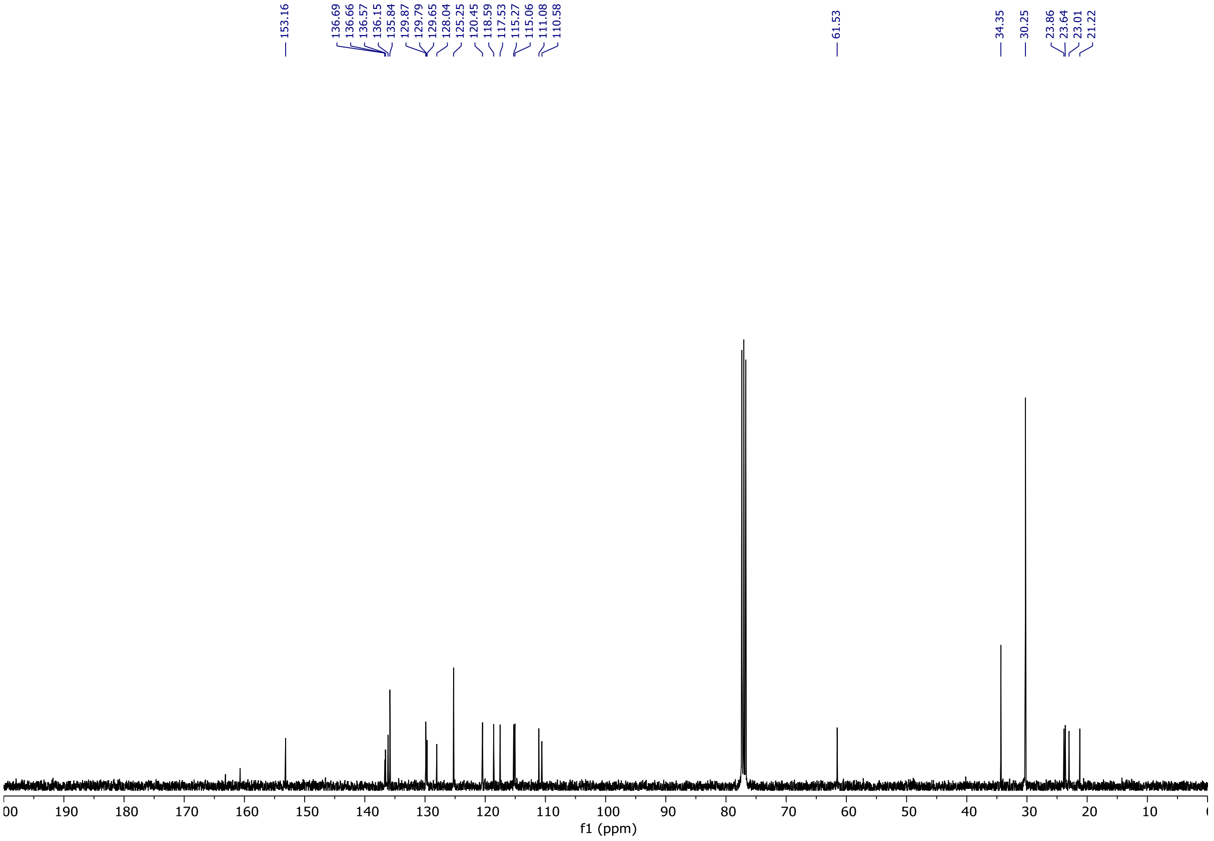

^{1}H NMR (400 MHz) and ^{13}C{^{1}H} NMR (100 MHz) spectra of **4an** ($CDCl_3$)

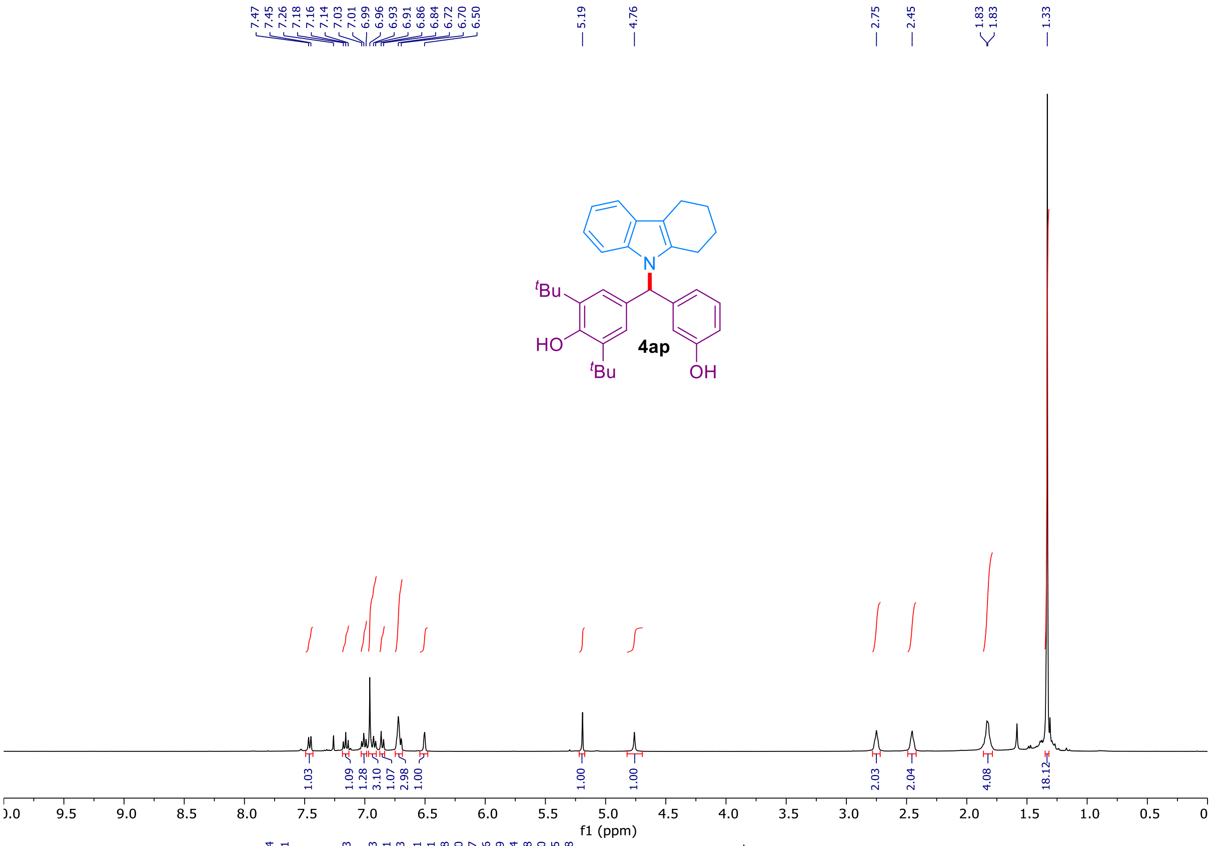

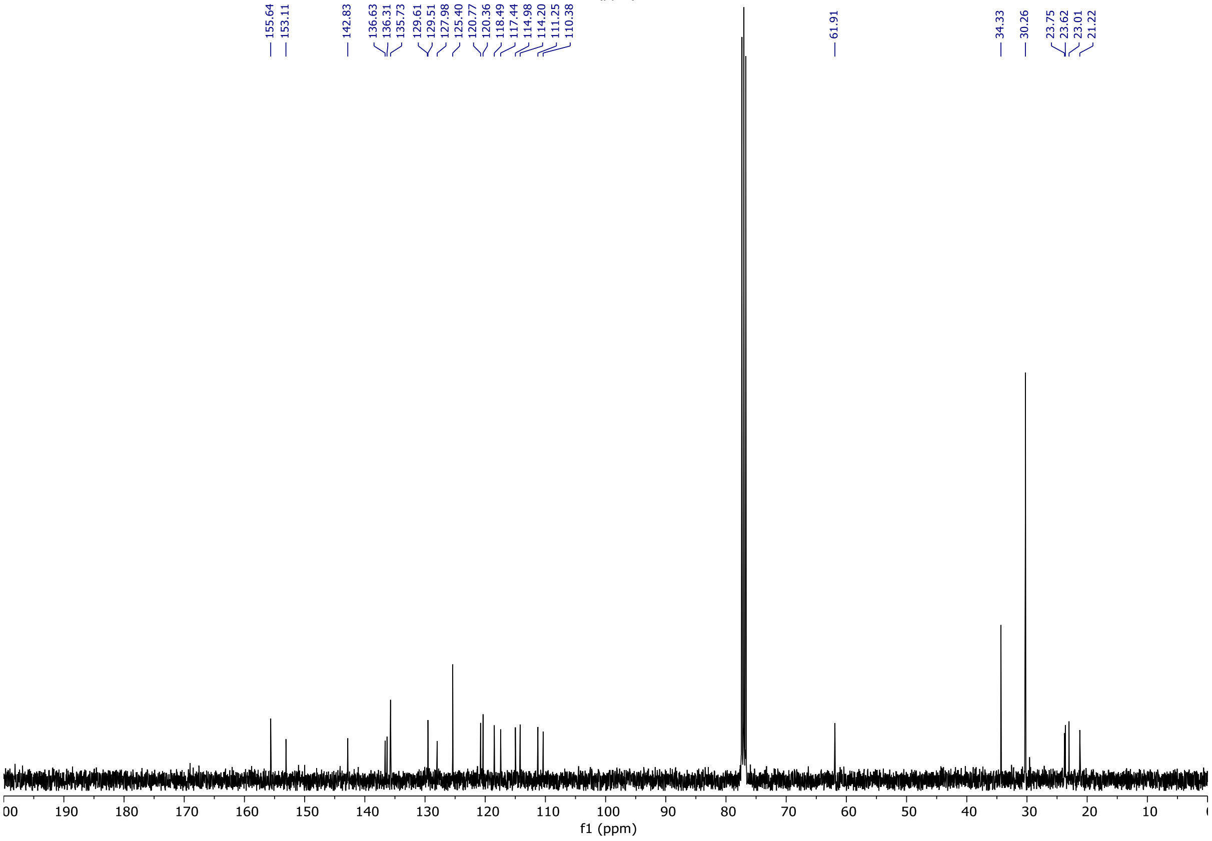

^{1}H NMR (400 MHz) and $^{13}C\{^{1}H\}$ NMR (100 MHz) spectra of **4ap** ($CDCl_3$)

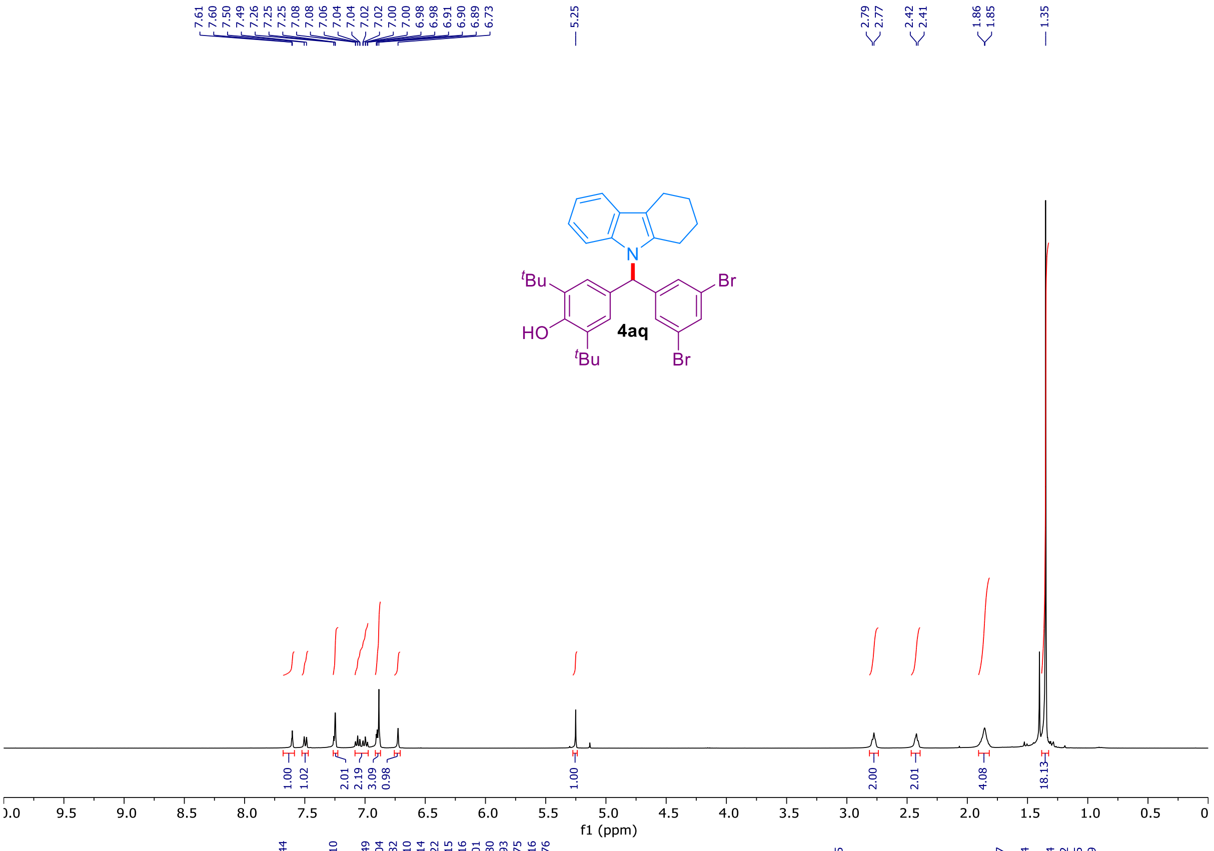

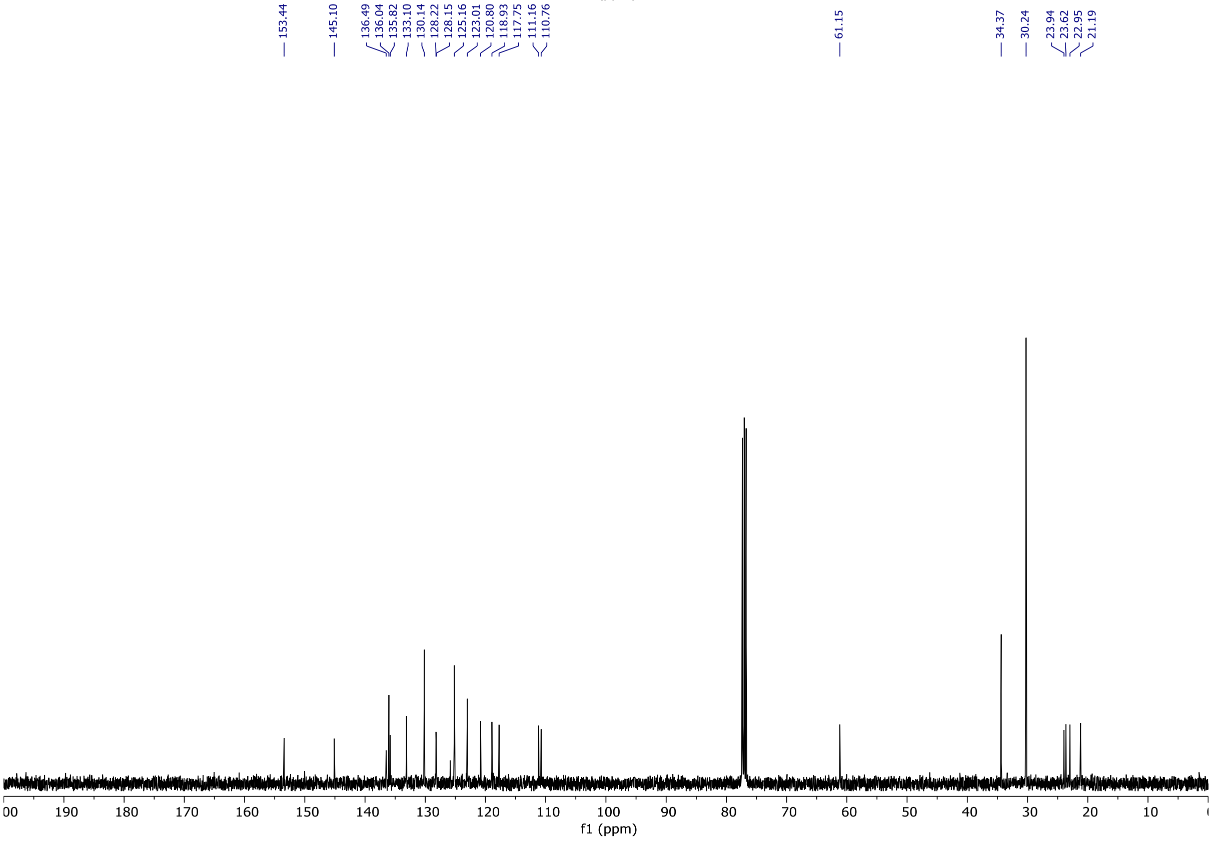

^{1}H NMR (400 MHz) and $^{13}C\{^{1}H\}$ NMR (100 MHz) spectra of **4aq** ($CDCl_3$)

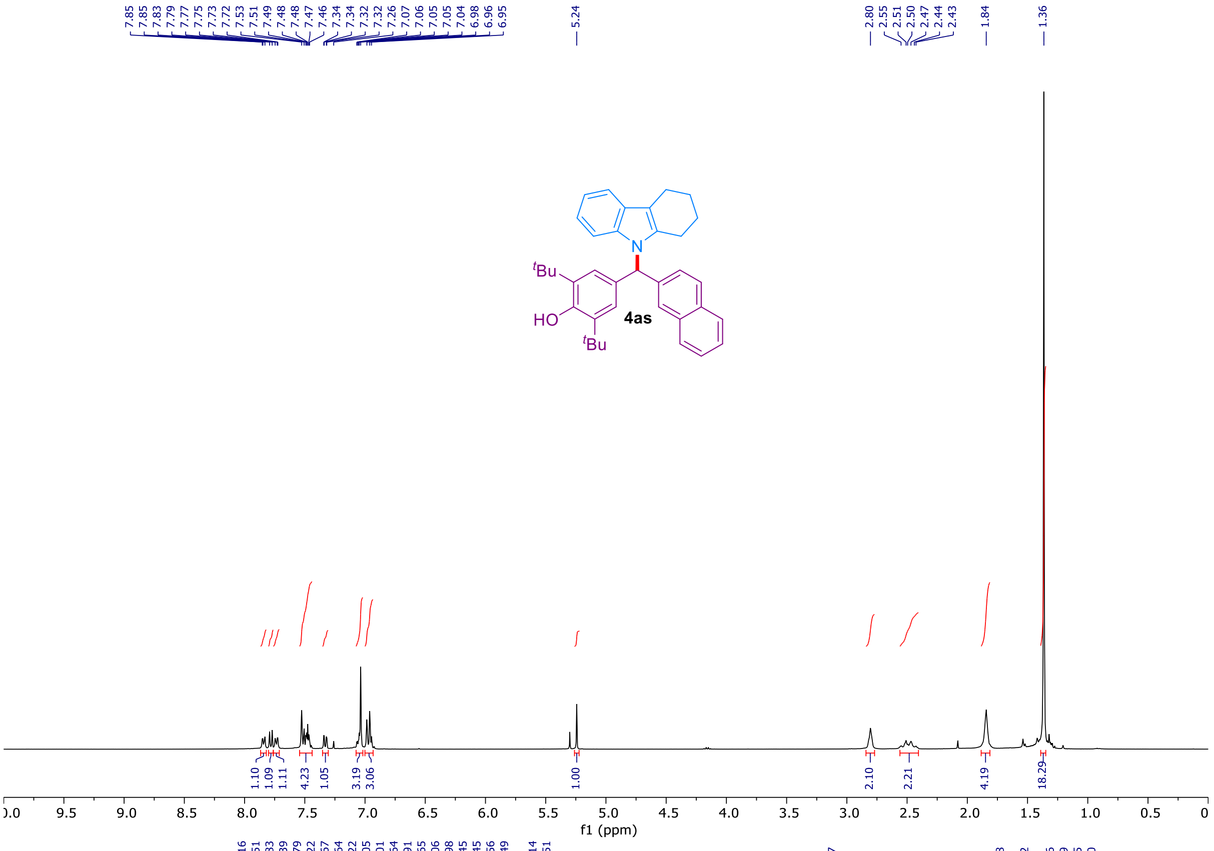

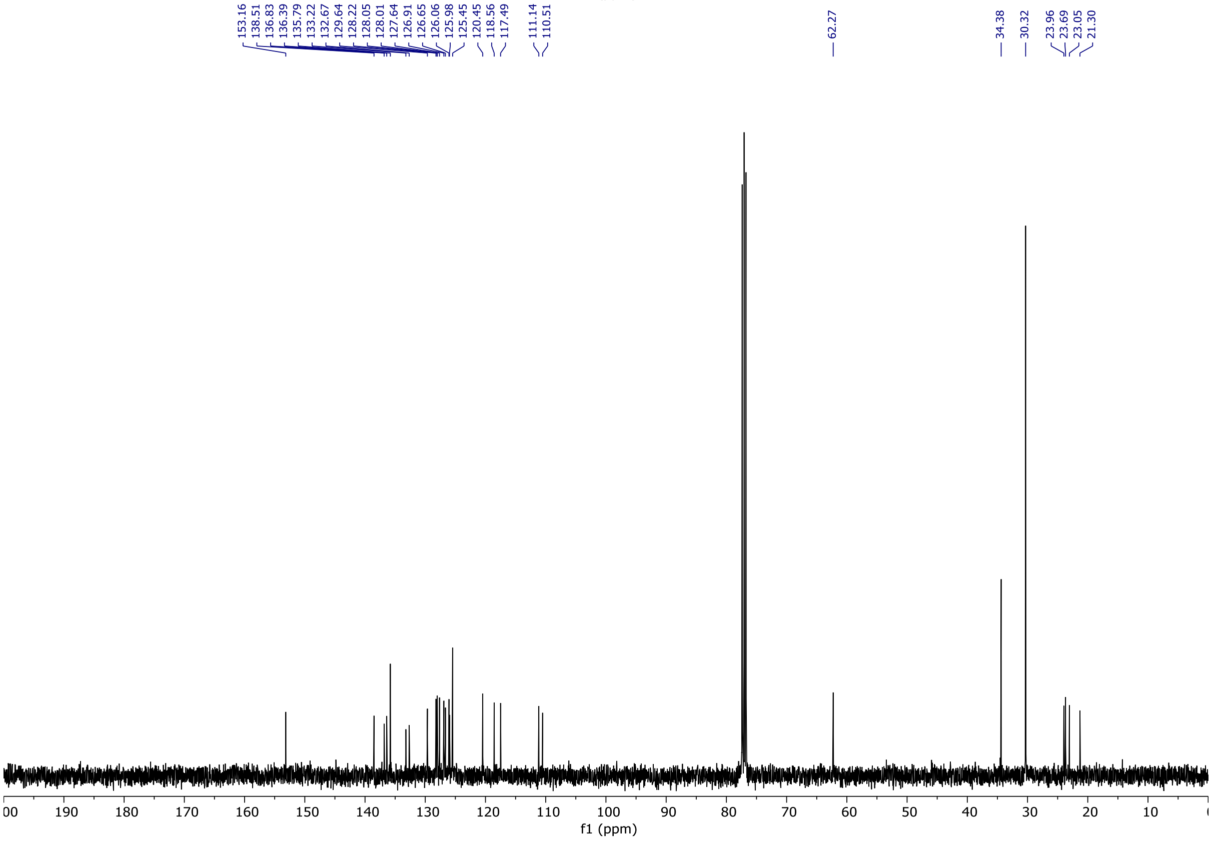

^{1}H NMR (400 MHz) and ^{13}C{^{1}H} NMR (100 MHz) spectra of **4as** ($CDCl_3$)

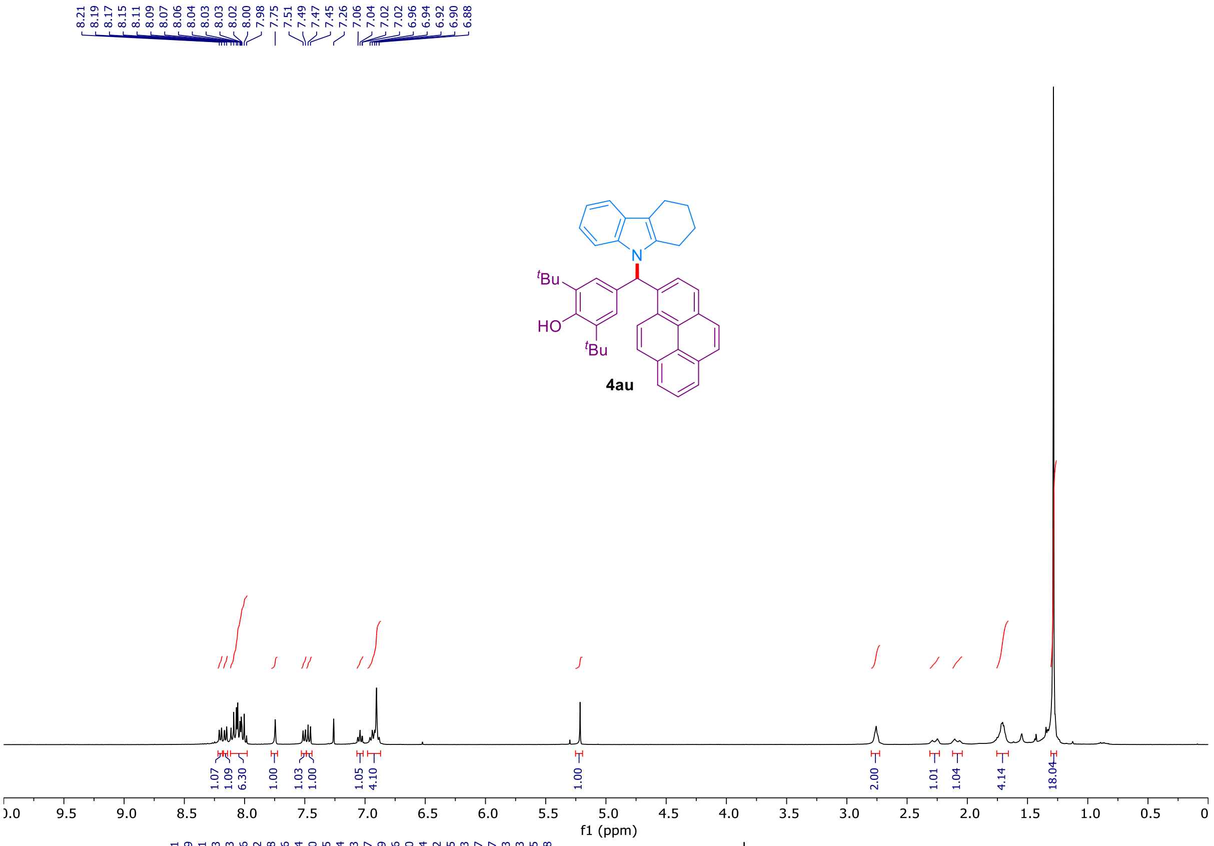

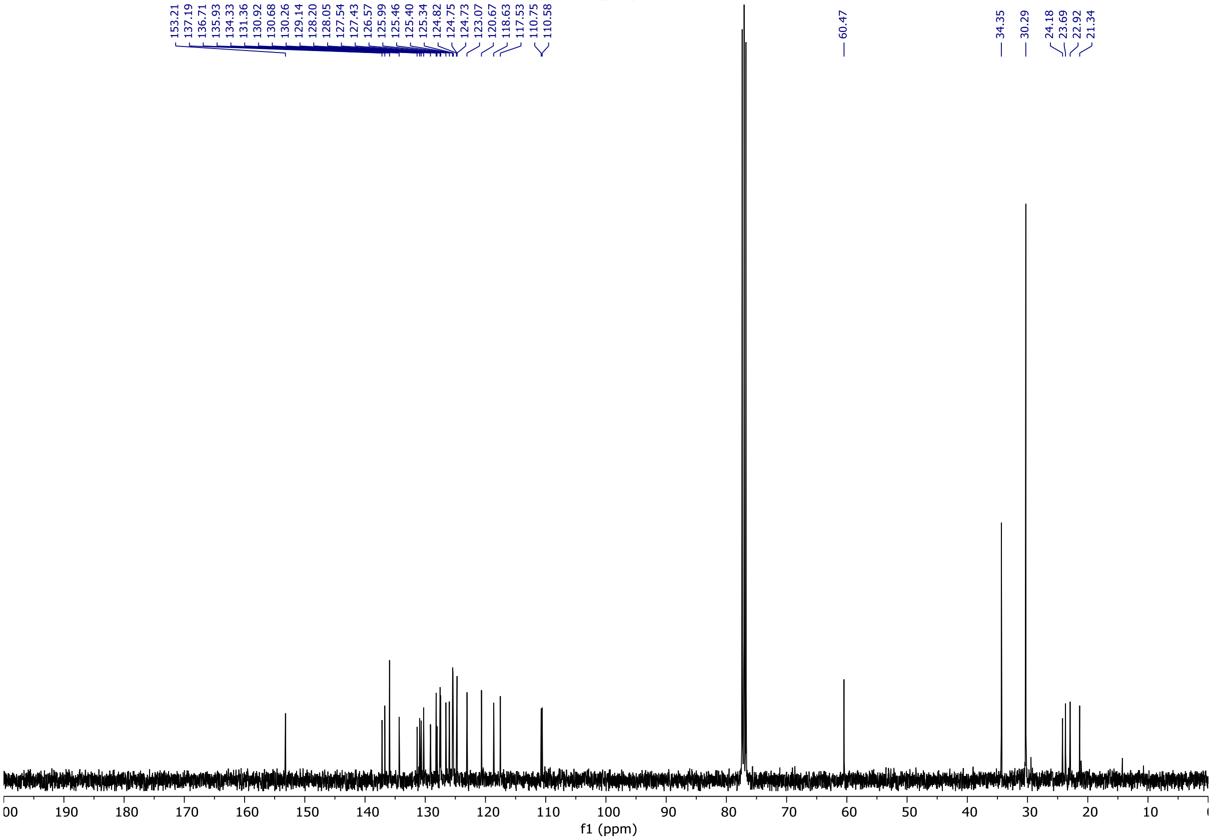

^{1}H NMR (400 MHz) and $^{13}C\{^{1}H\}$ NMR (100 MHz) spectra of **4au** ($CDCl_3$)

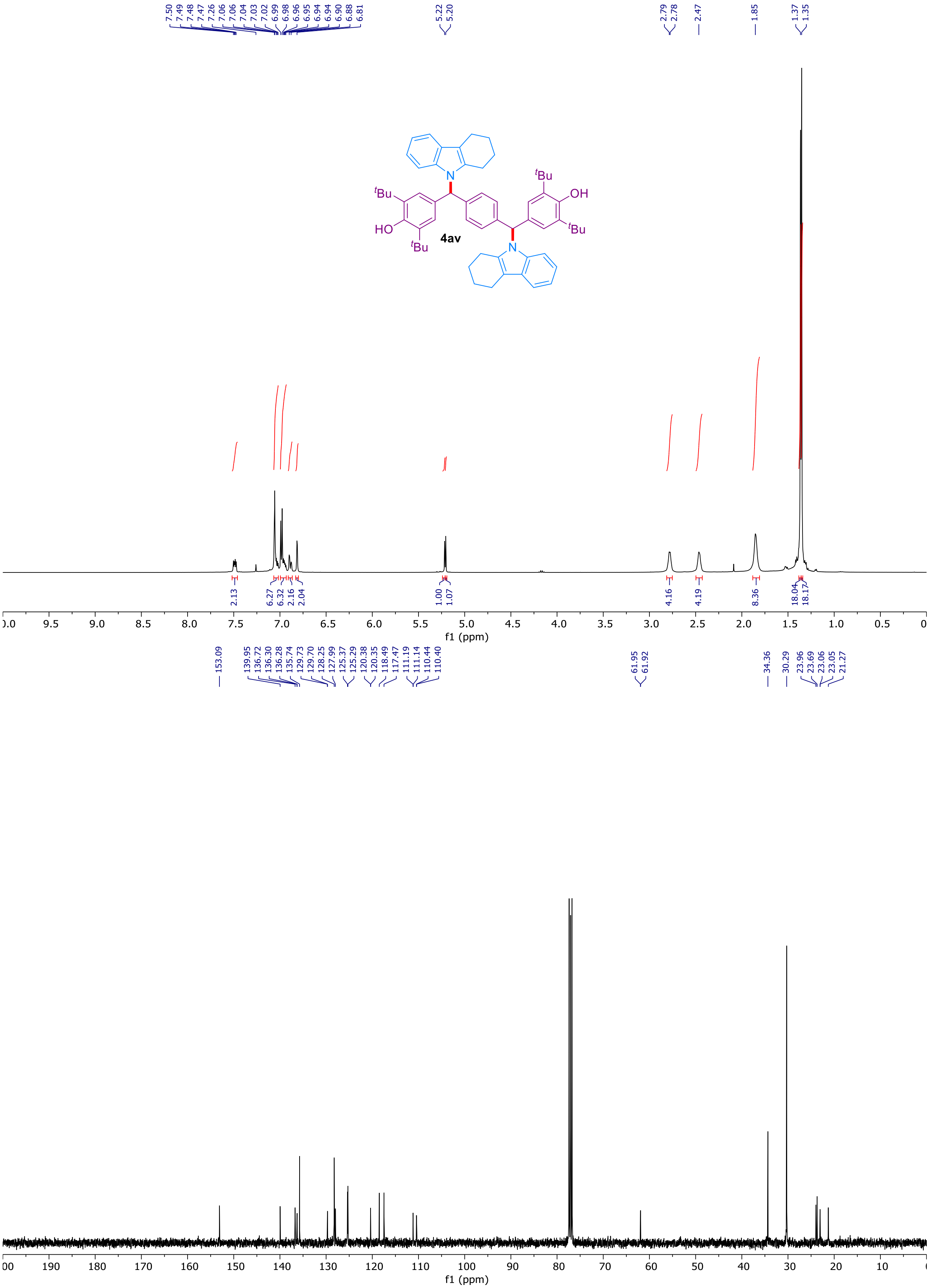

^{1}H NMR (400 MHz) and $^{13}C\{^{1}H\}$ NMR (100 MHz) spectra of **4av** ($CDCl_3$)

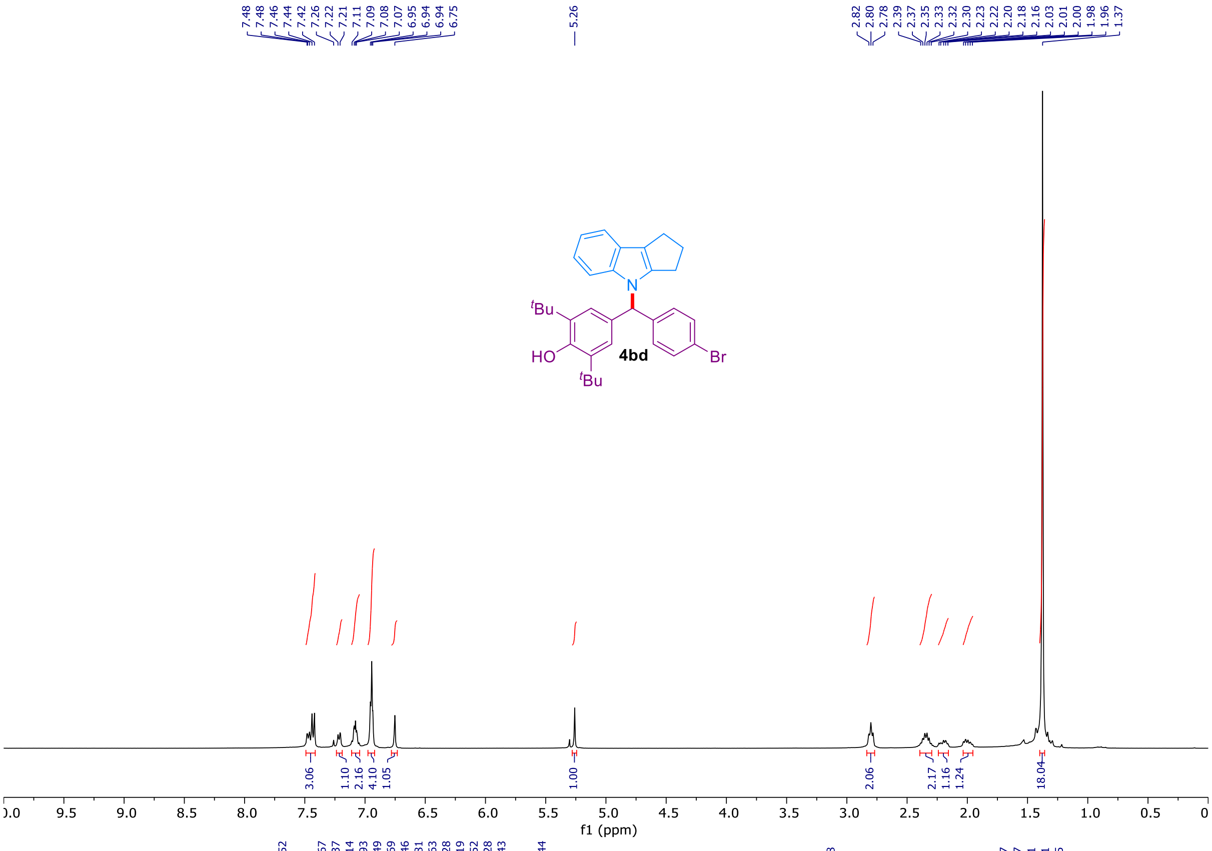

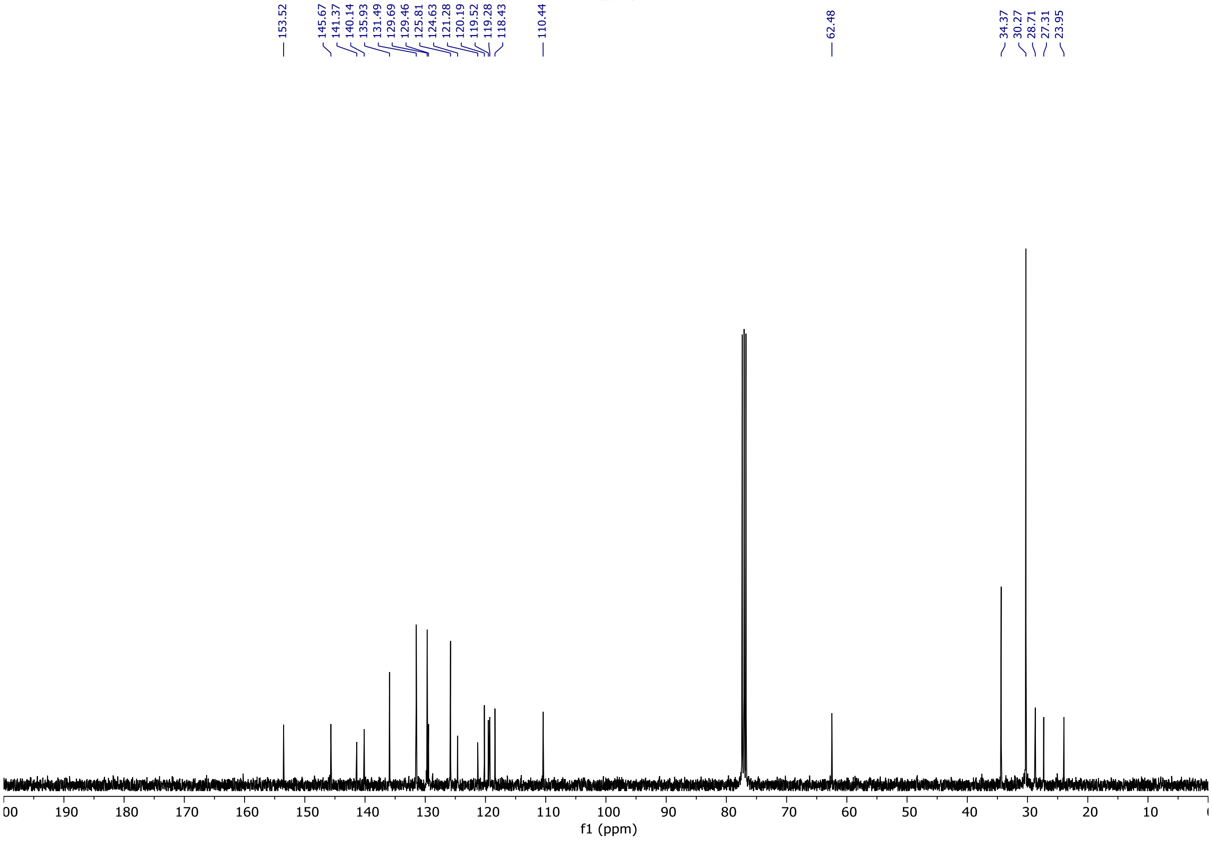

^{1}H NMR (400 MHz) and ^{13}C{^{1}H} NMR (100 MHz) spectra of **4bd** ($CDCl_3$)

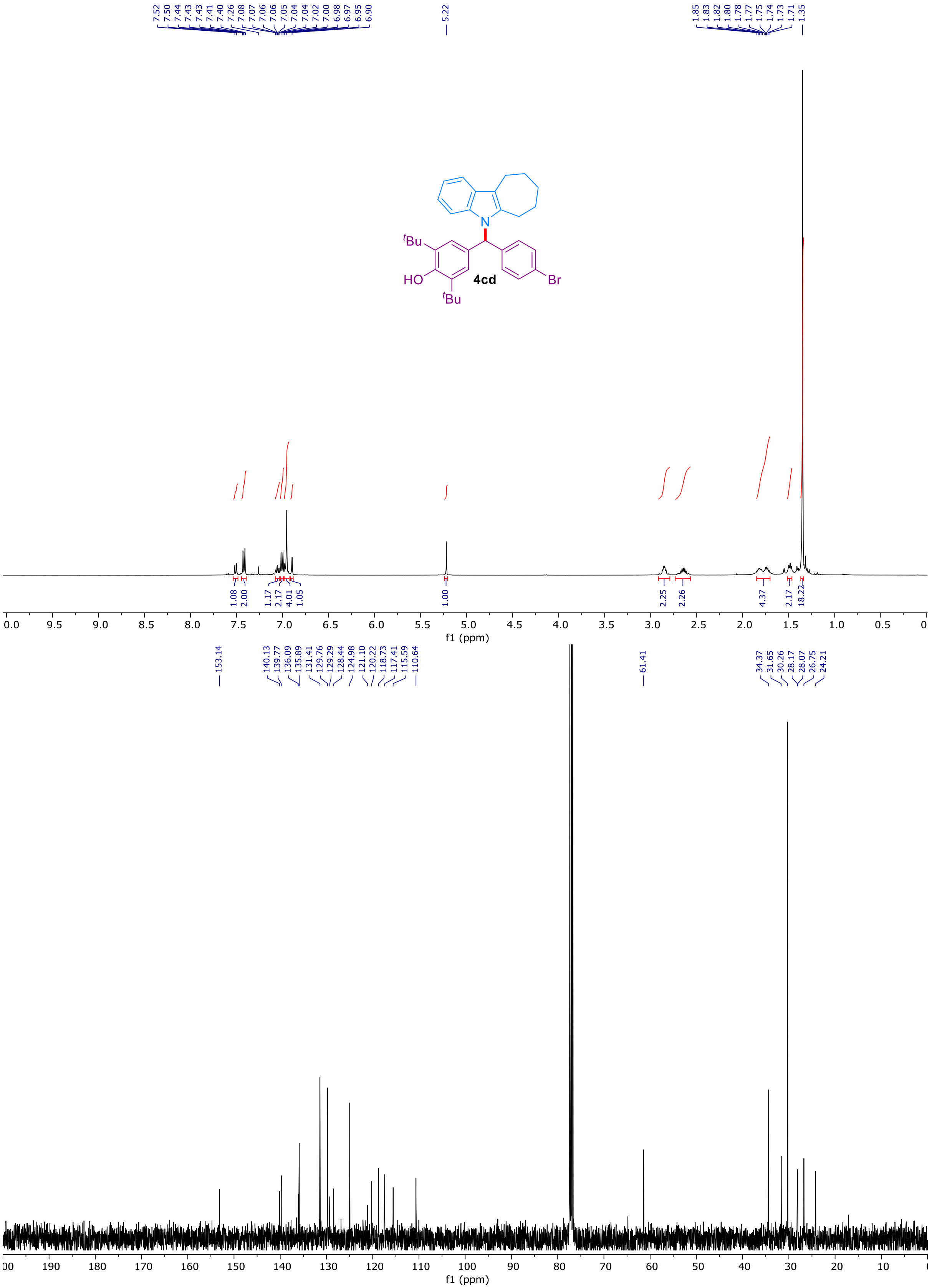

^{1}H NMR (400 MHz) and $^{13}C\{^{1}H\}$ NMR (100 MHz) spectra of **4cd** ($CDCl_3$)

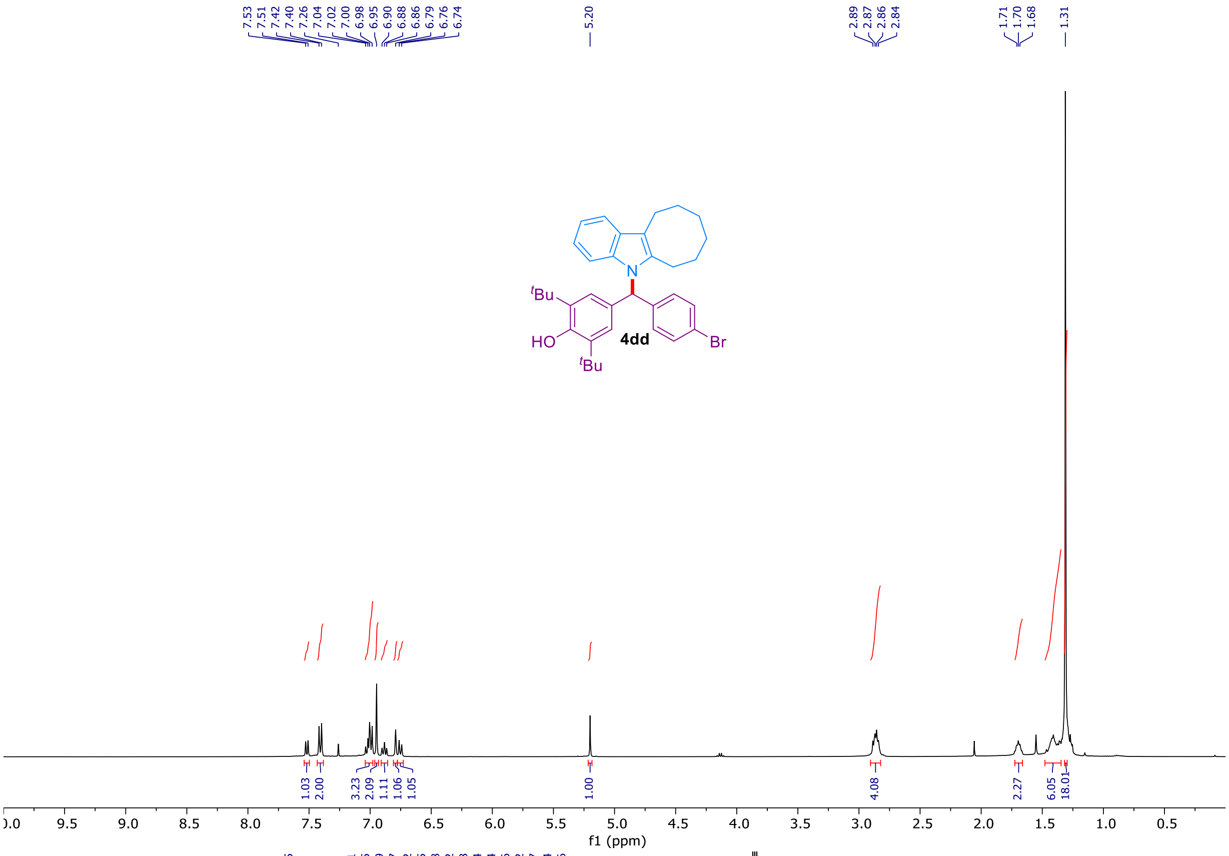

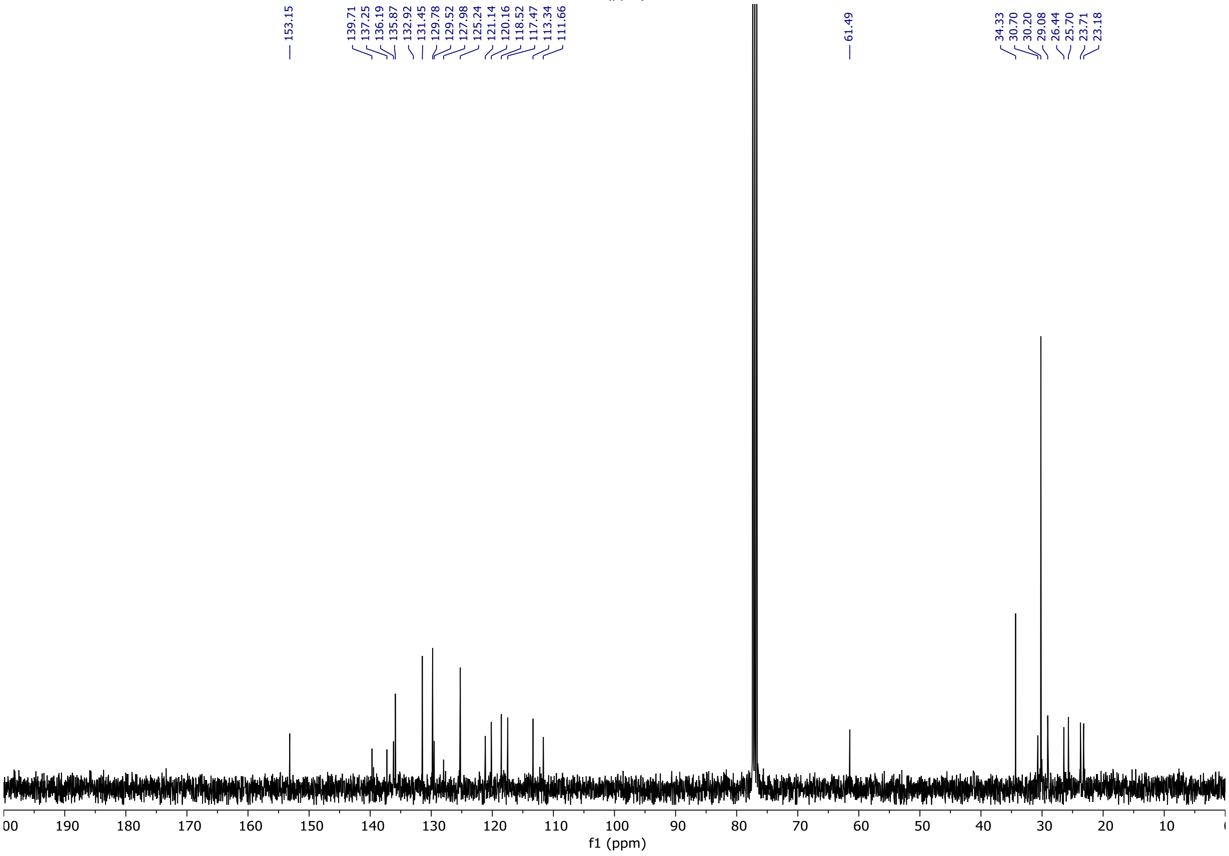

^{1}H NMR (400 MHz) and ^{13}C{^{1}H} NMR (100 MHz) spectra of **4dd** ($CDCl_3$)

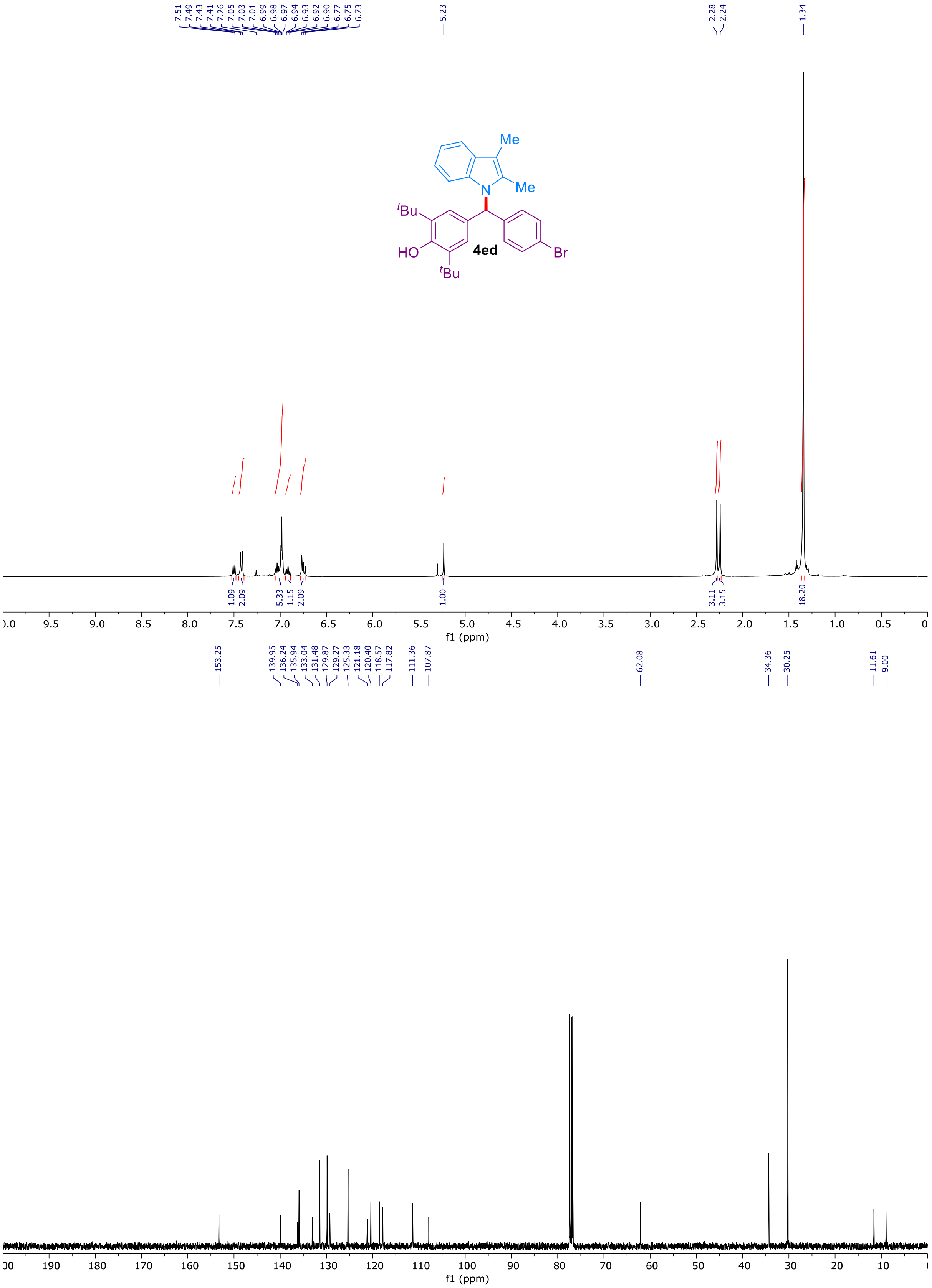

^{1}H NMR (400 MHz) and $^{13}C\{^{1}H\}$ NMR (100 MHz) spectra of **4ed** ($CDCl_3$)

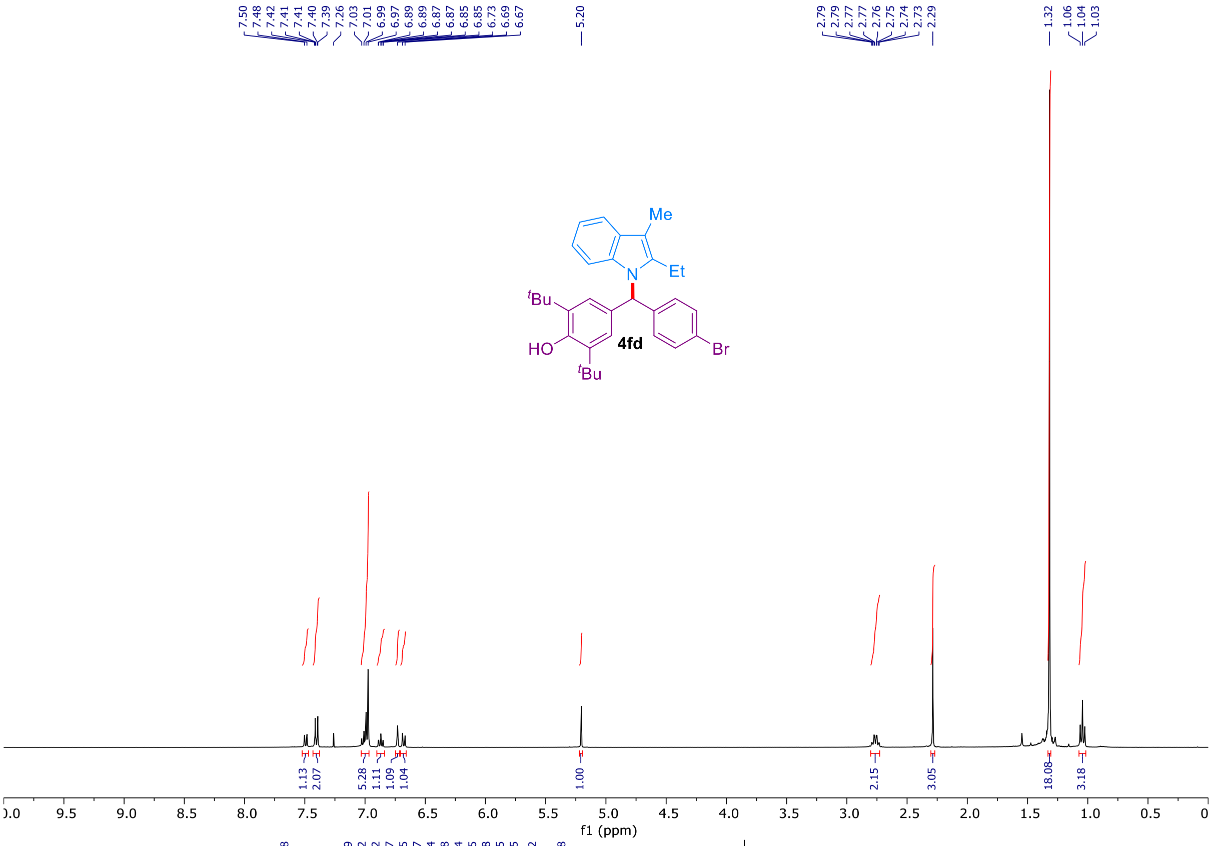

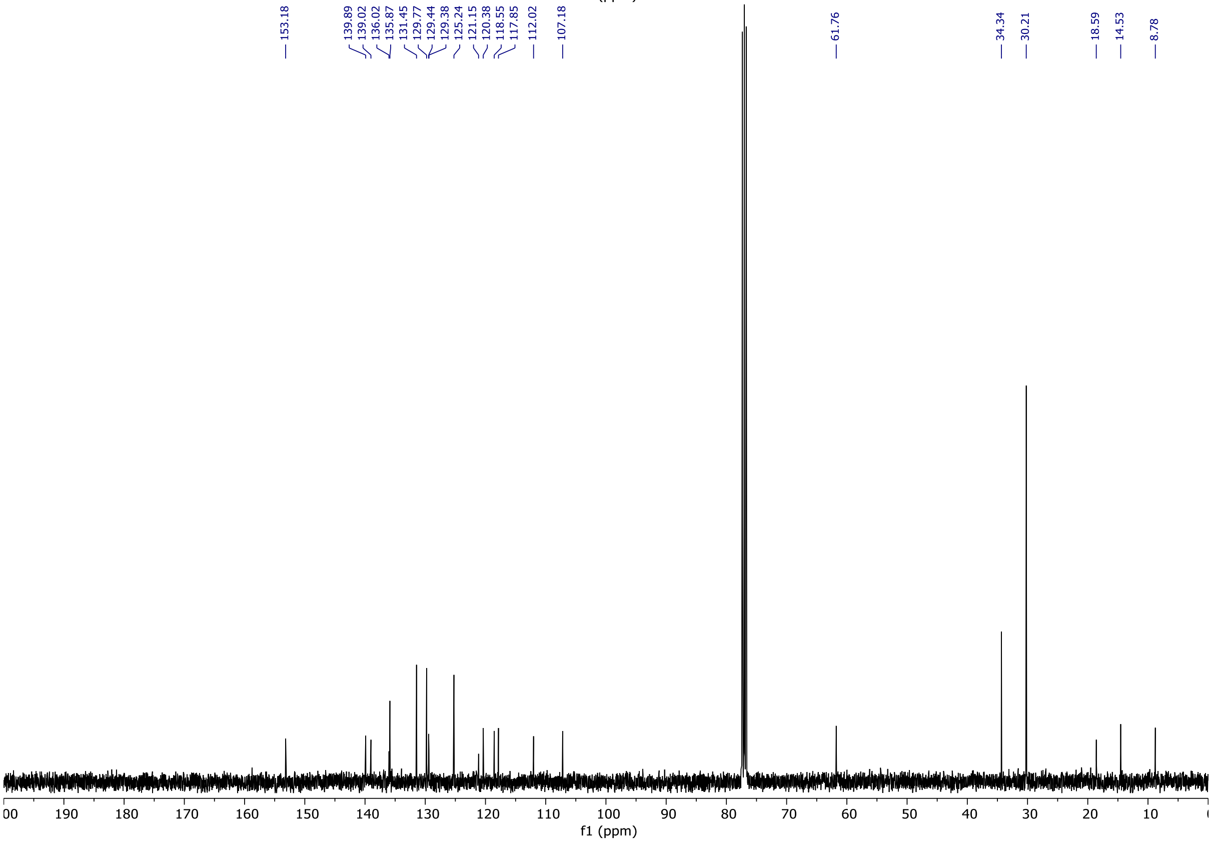

^{1}H NMR (400 MHz) and ^{13}C{^{1}H} NMR (100 MHz) spectra of **4fd** ($CDCl_3$)

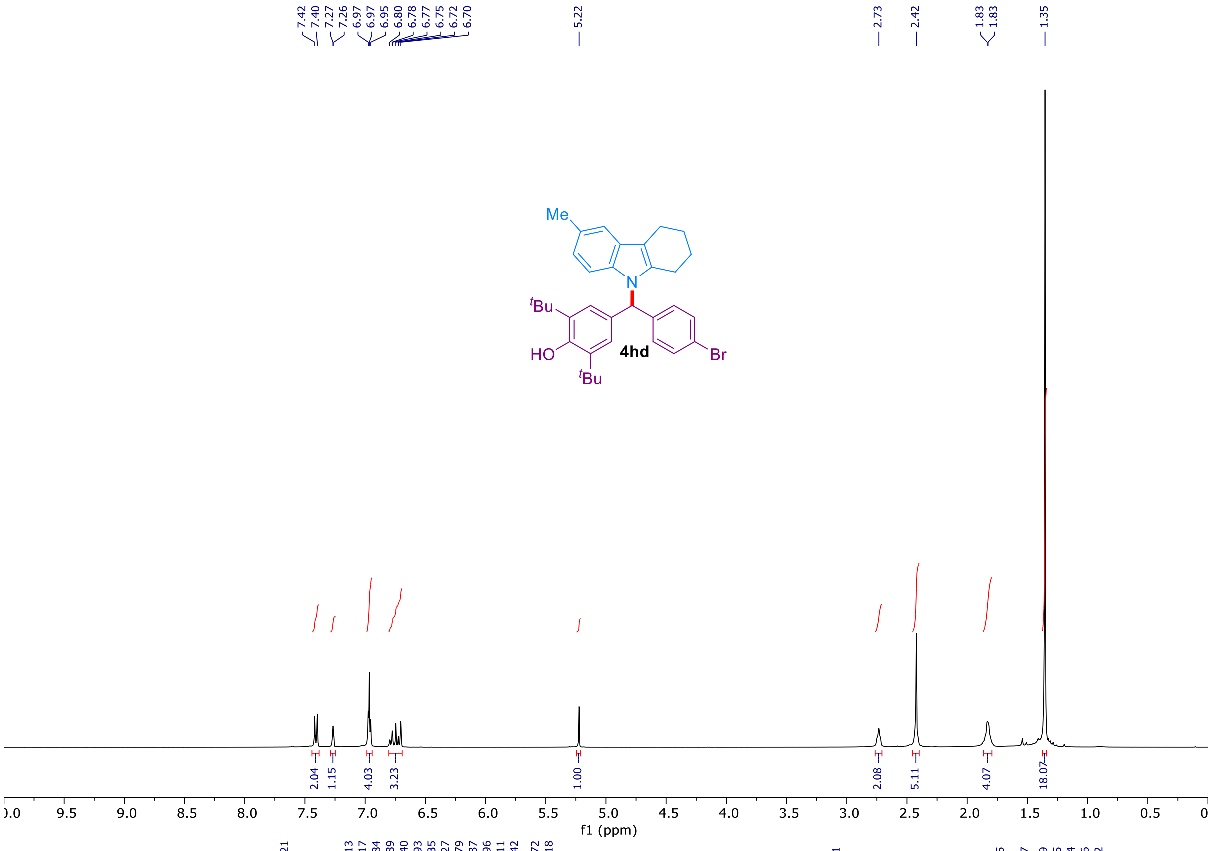

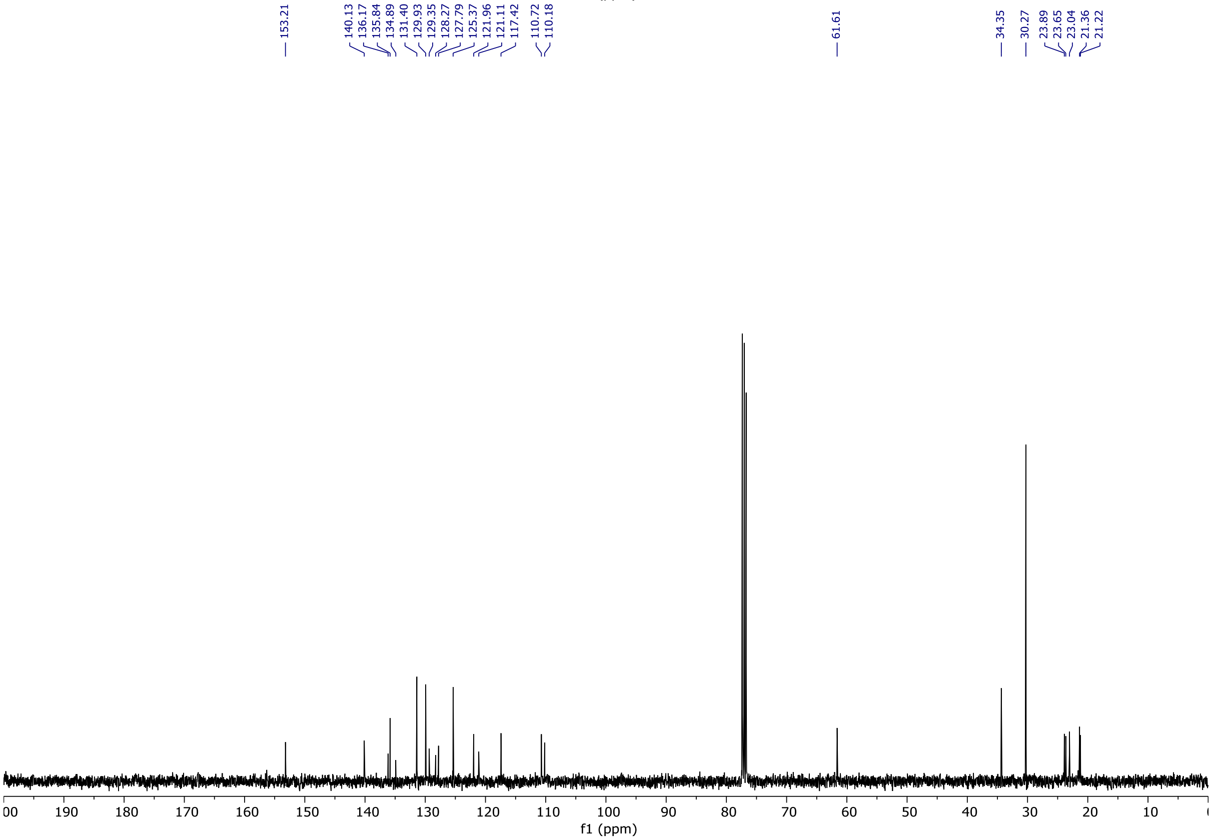

^{1}H NMR (400 MHz) and ^{13}C{^{1}H} NMR (100 MHz) spectra of **4hd** ($CDCl_3$)

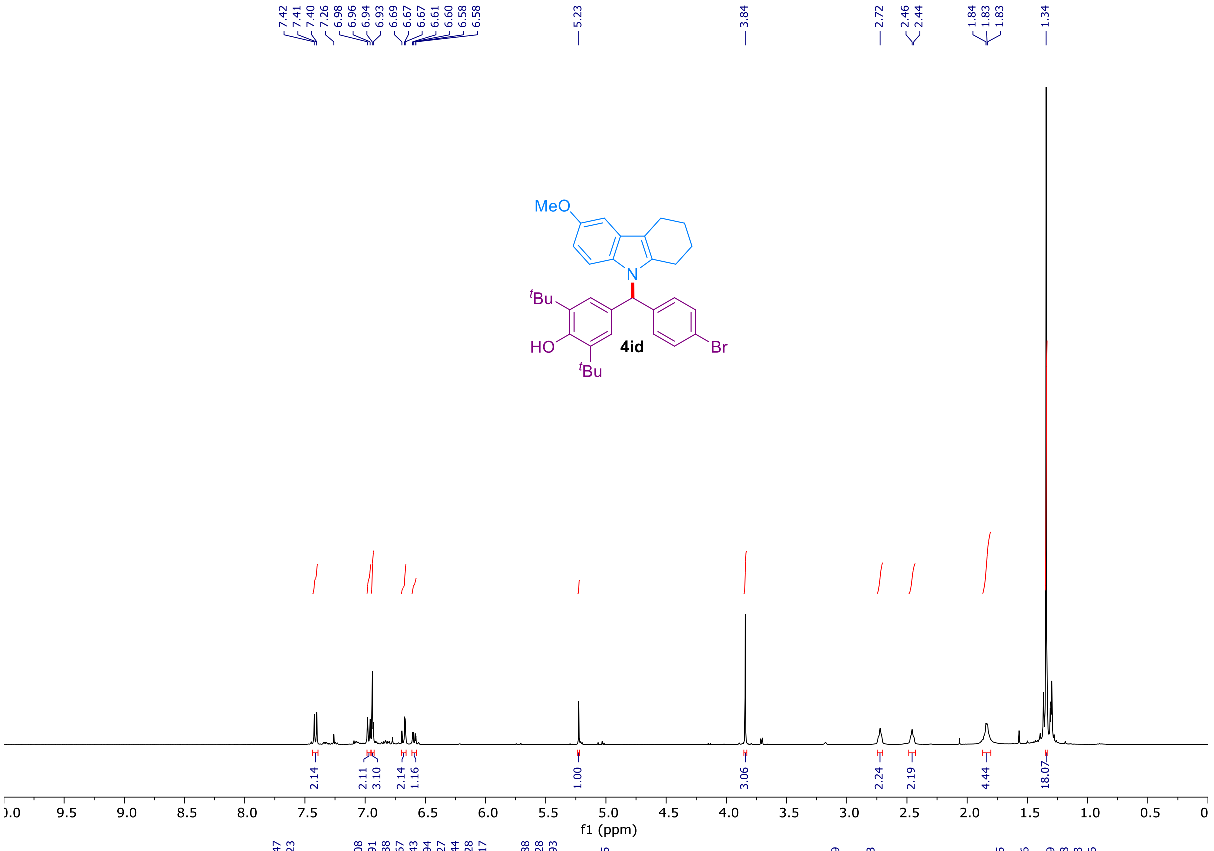

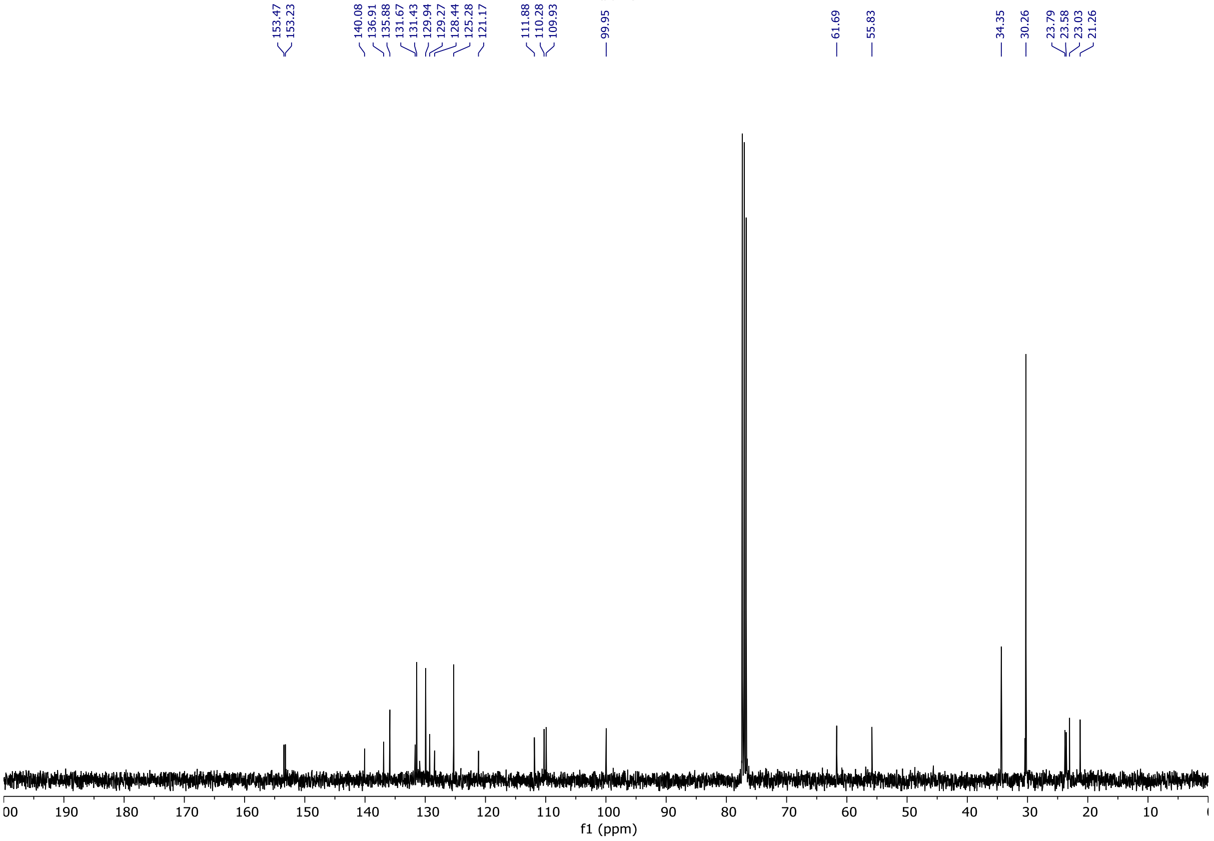

^{1}H NMR (400 MHz) and $^{13}C\{^{1}H\}$ NMR (100 MHz) spectra of **4id** ($CDCl_3$)

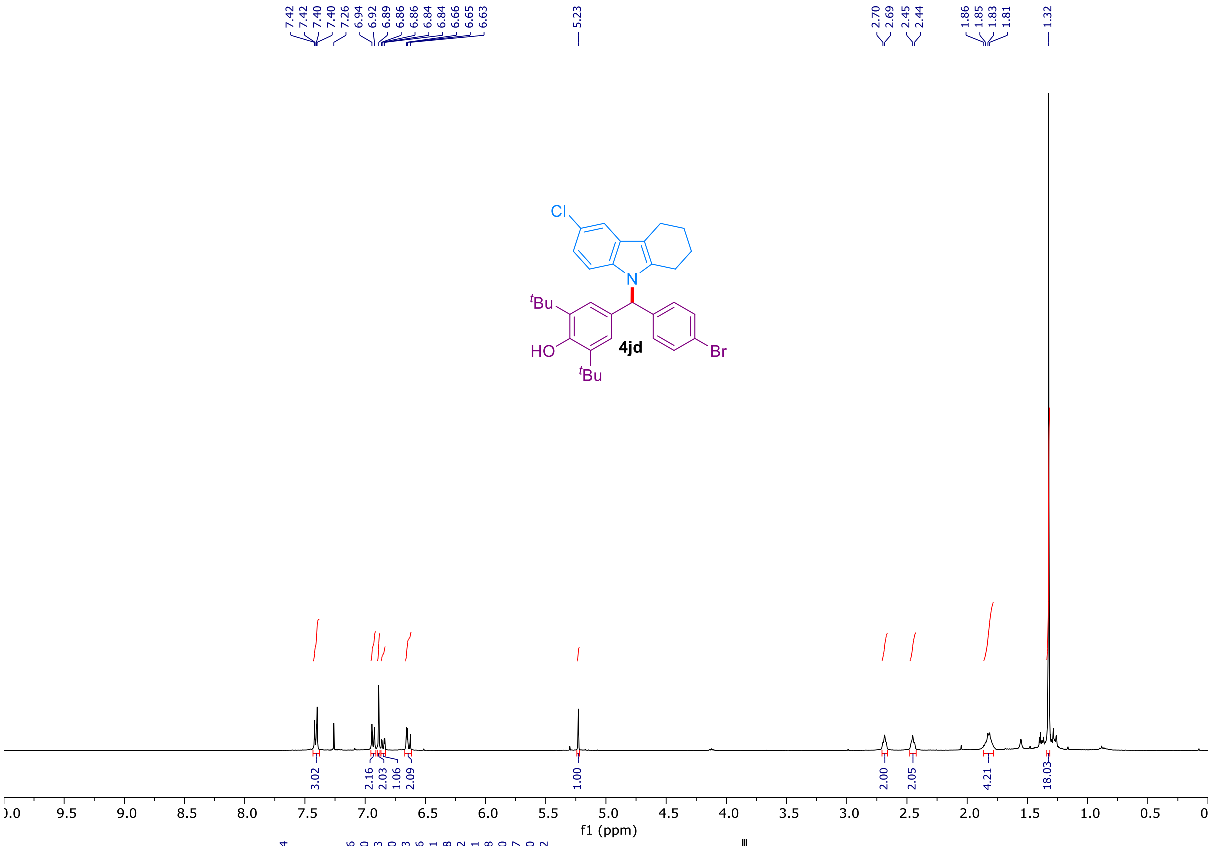

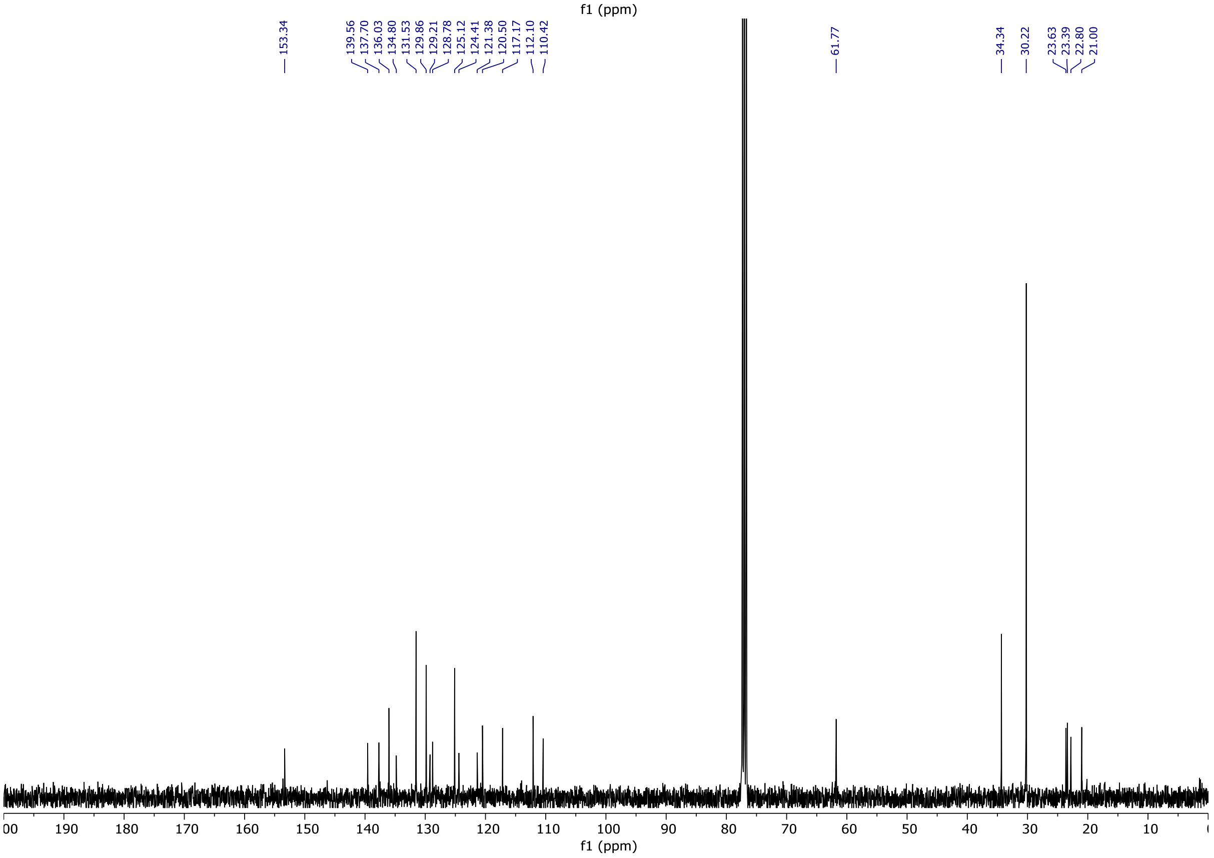

^{1}H NMR (400 MHz) and ^{13}C{^{1}H} NMR (100 MHz) spectra of **4jd** ($CDCl_3$)

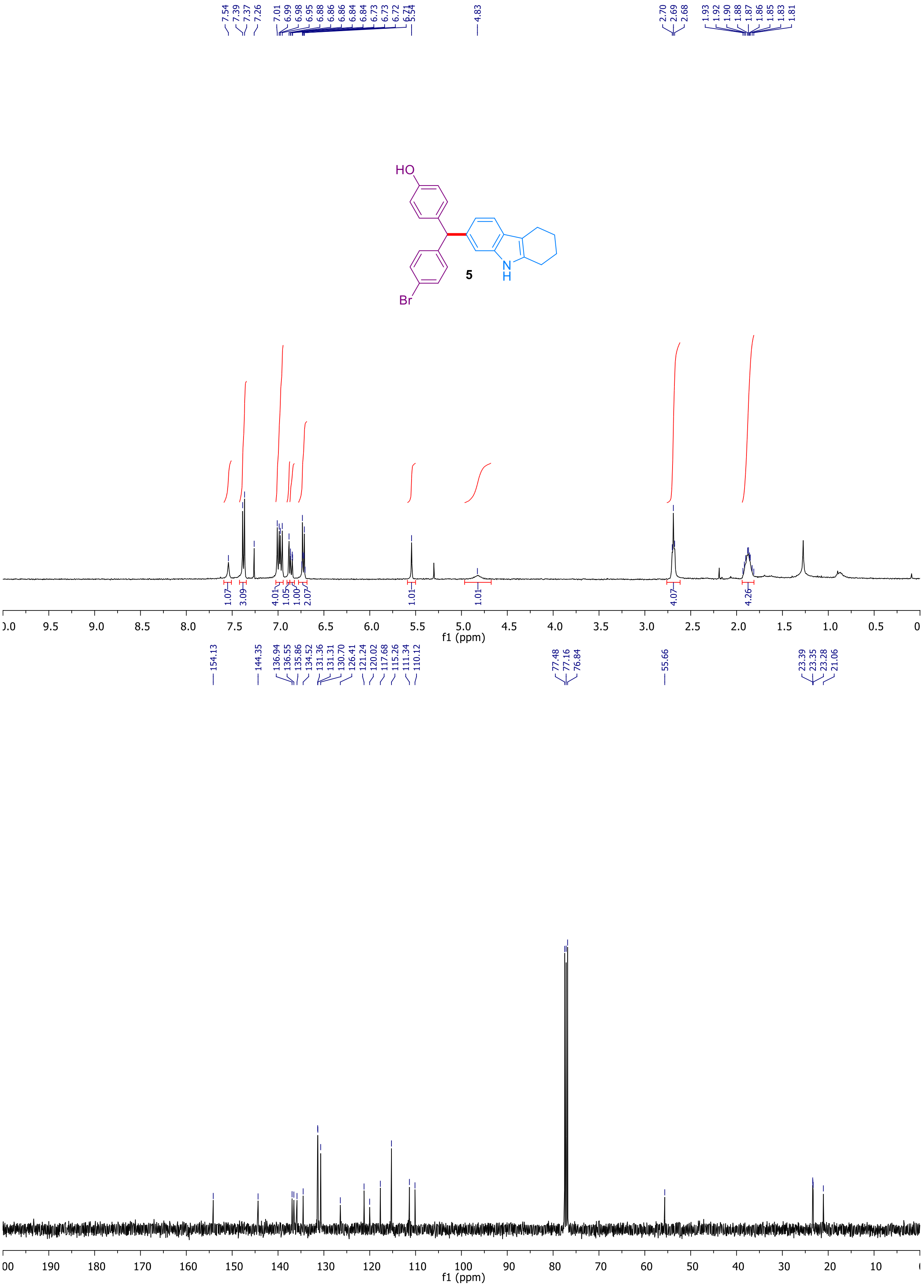

^{1}H NMR (400 MHz) and $^{13}C\{^{1}H\}$ NMR (100 MHz) spectra of **5** ($CDCl_3$)

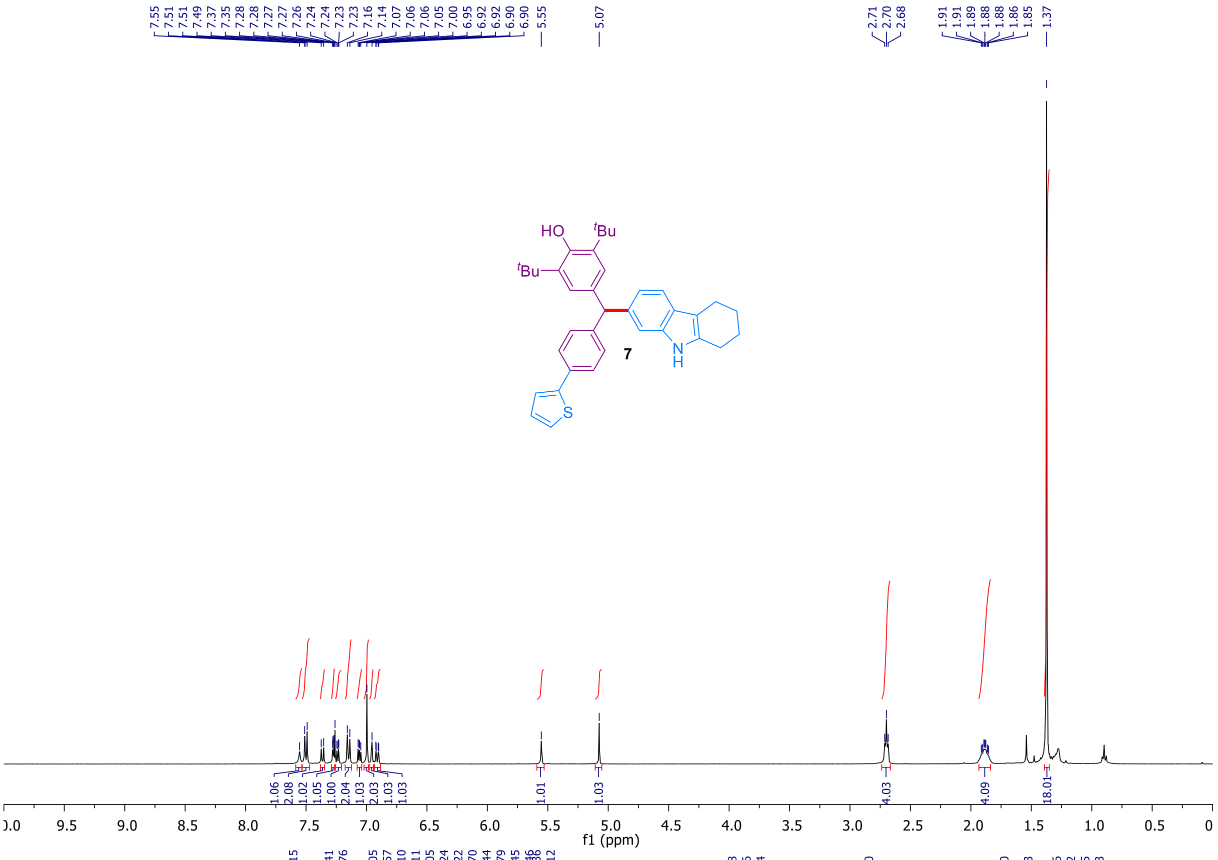

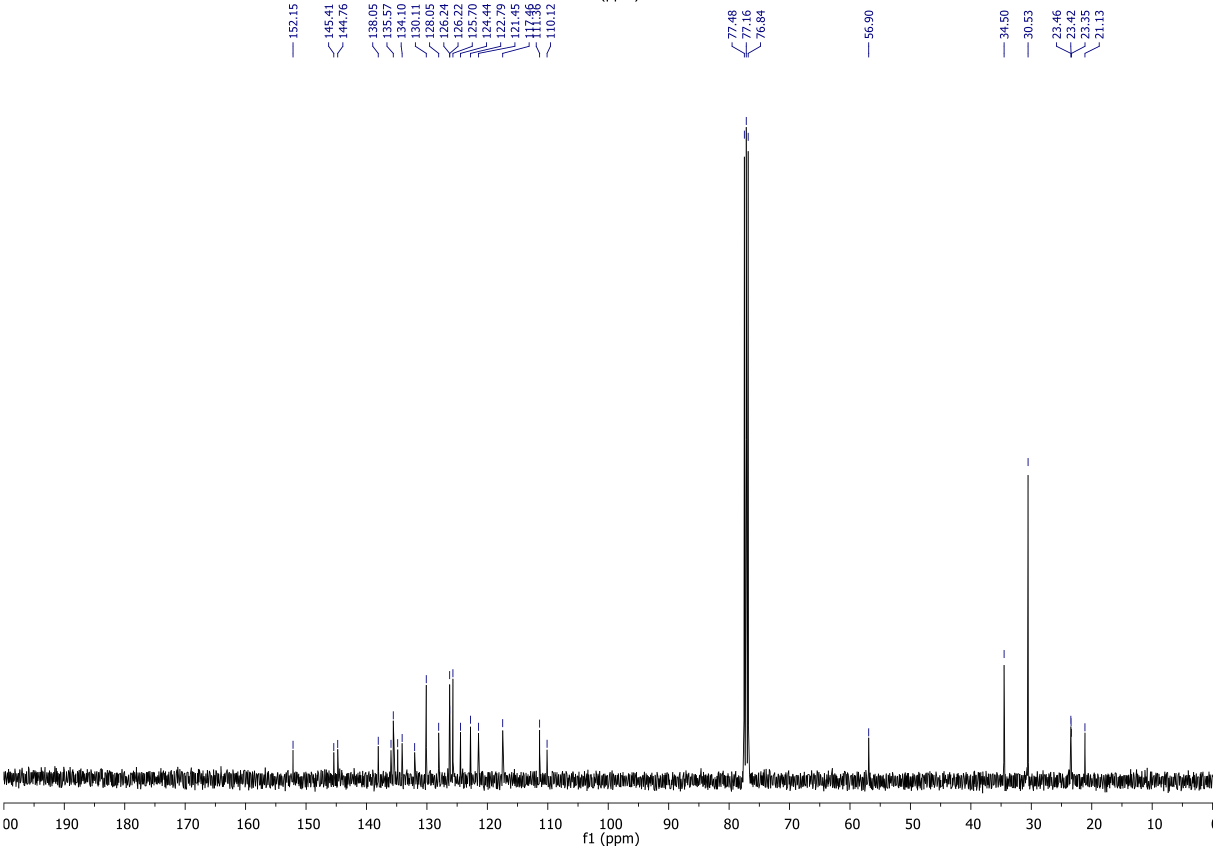

^1H NMR (400 MHz) and $^{13}C\{^1H\}$ NMR (100 MHz) spectra of **7** ($CDCl_3$)

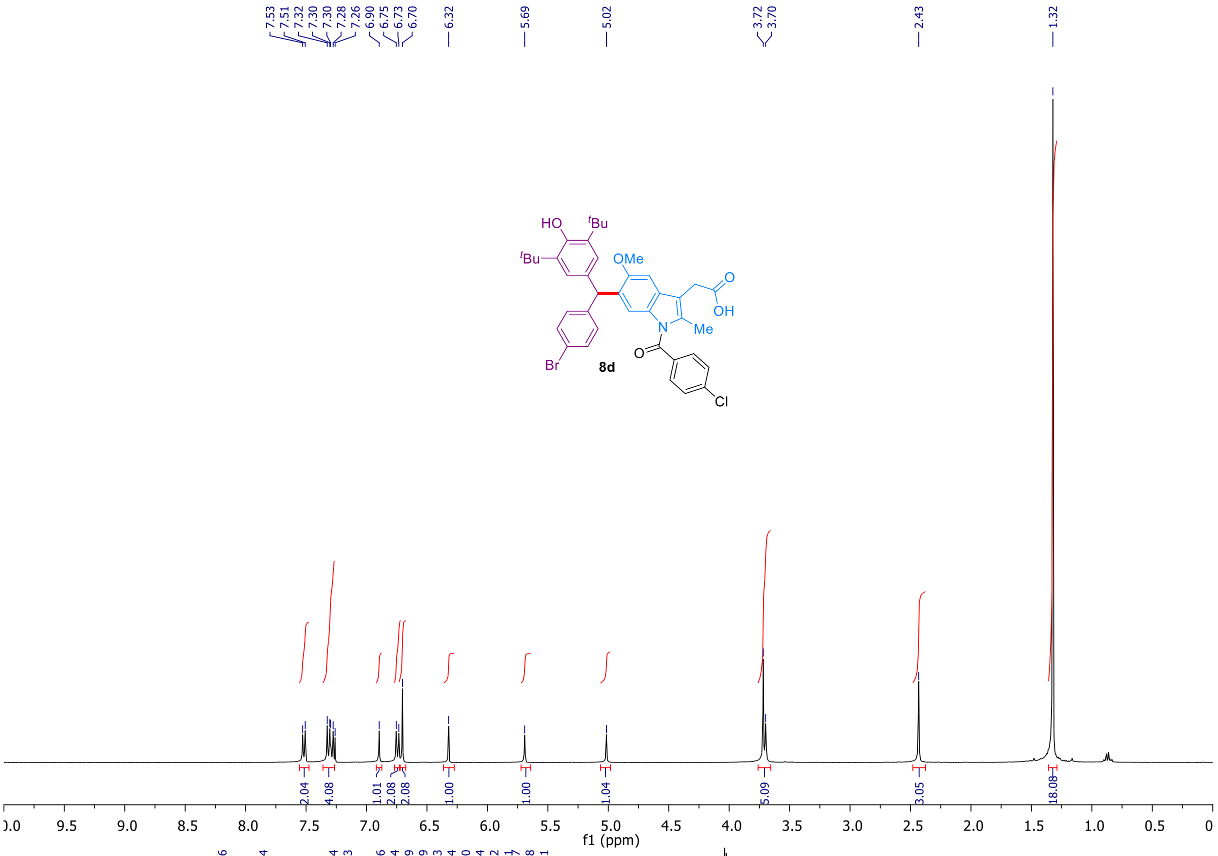

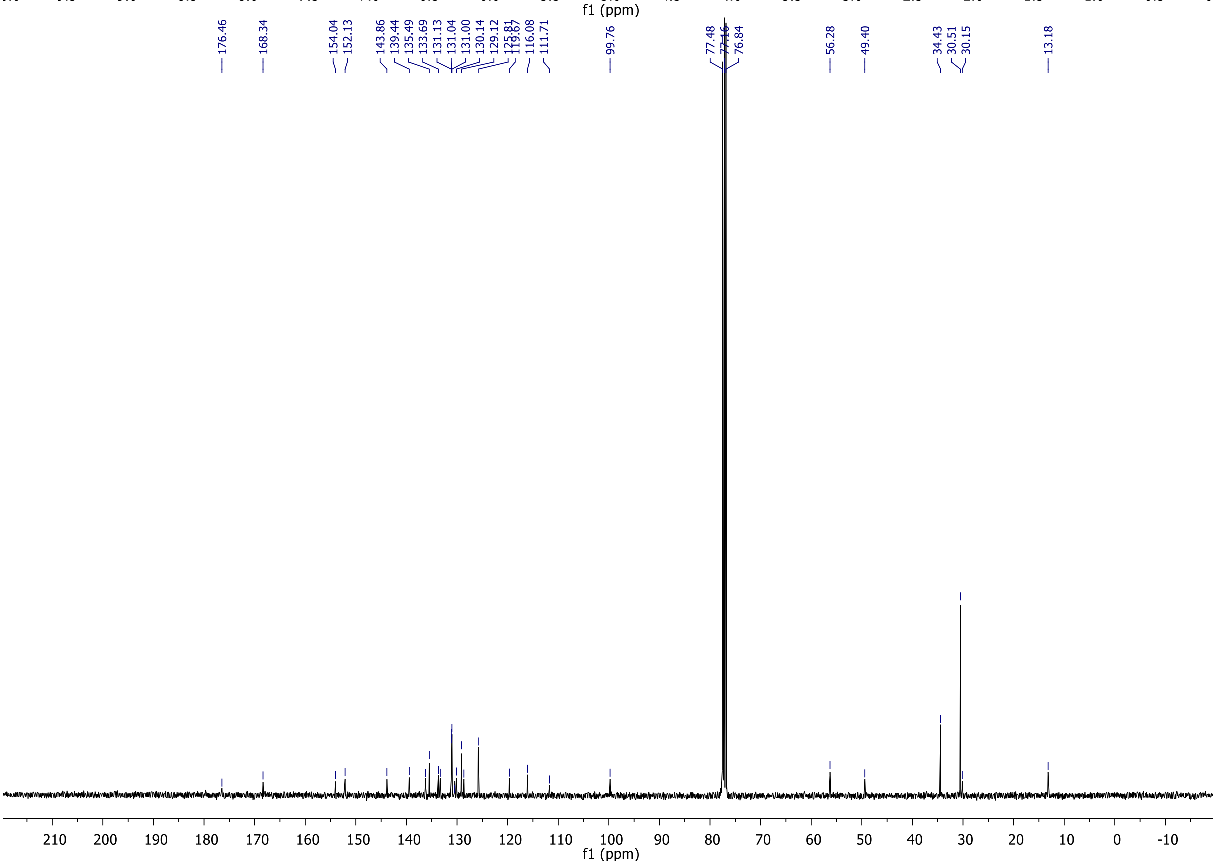

^{1}H NMR (400 MHz) and ^{13}C{^{1}H} NMR (100 MHz) spectra of **8d** ($CDCl_3$)

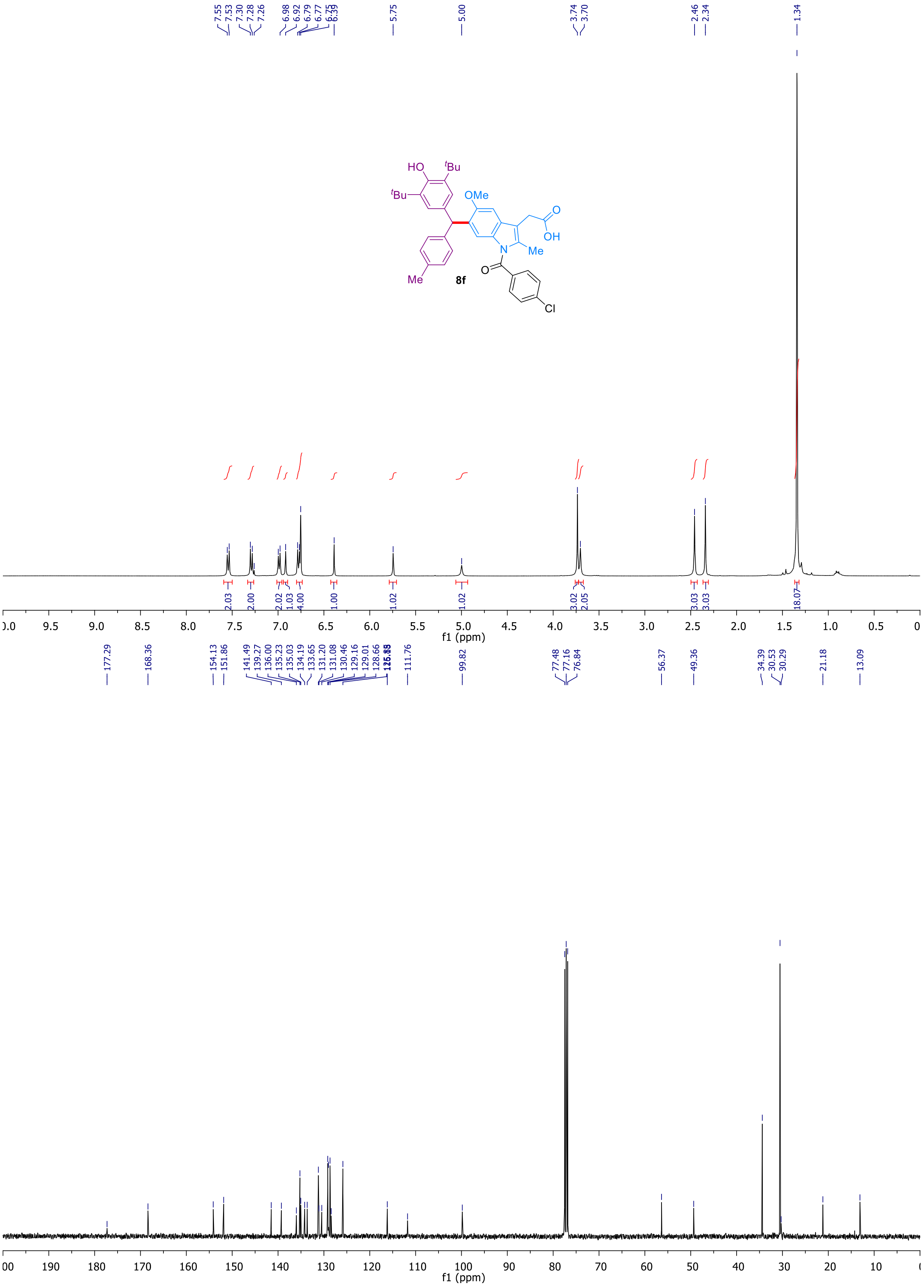

^{1}H NMR (400 MHz) and $^{13}C\{^{1}H\}$ NMR (100 MHz) spectra of **8f** ($CDCl_3$)

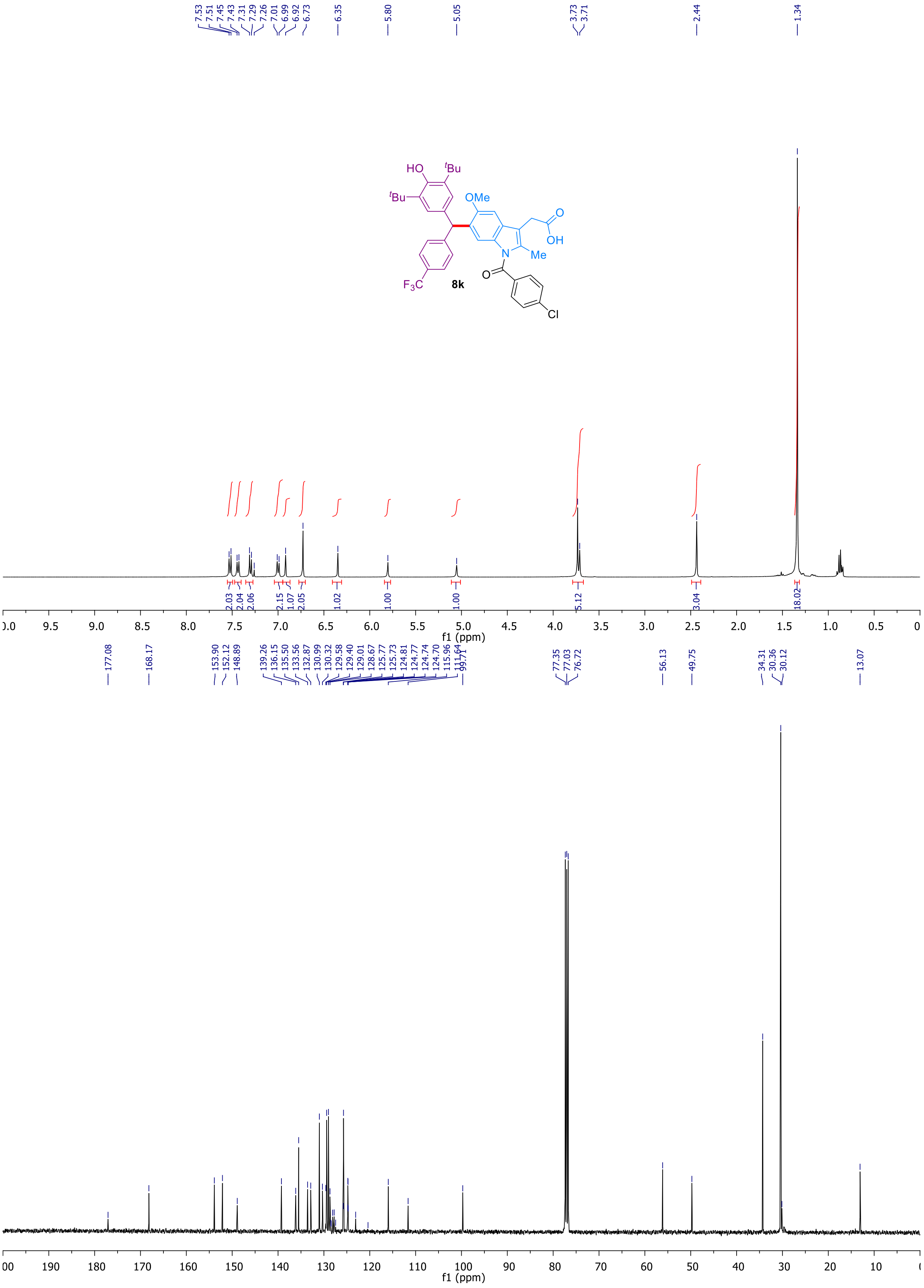

^{1}H NMR (400 MHz) and $^{13}C\{^{1}H\}$ NMR (100 MHz) spectra of **8k** ($CDCl_3$)

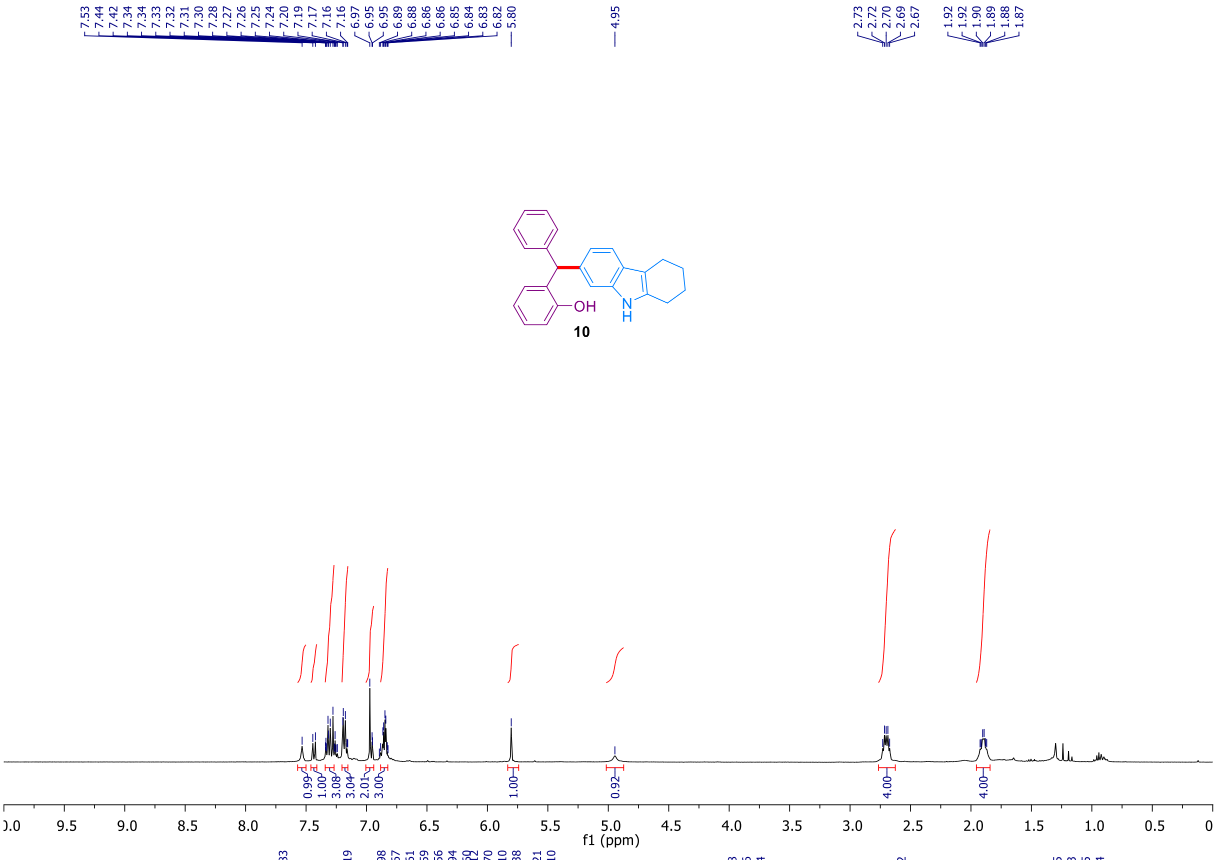

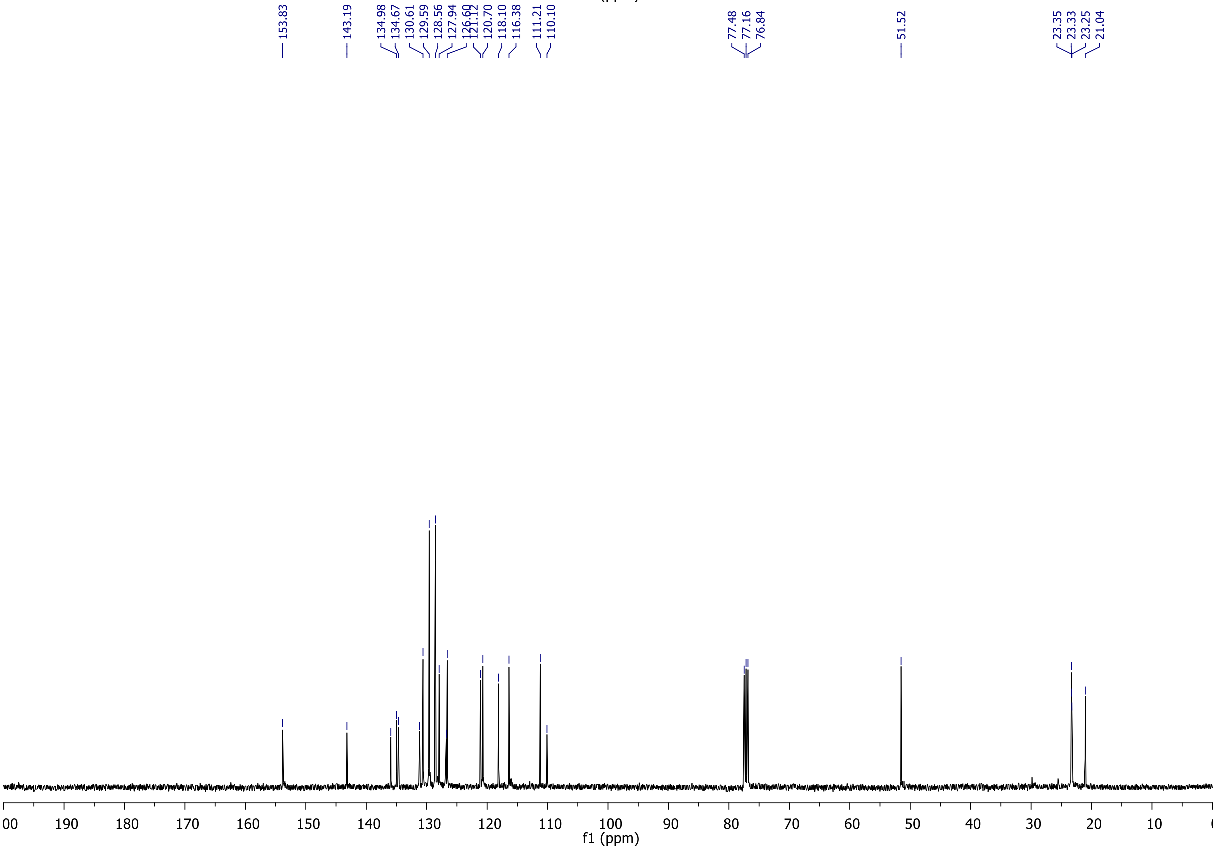

^{1}H NMR (400 MHz) and ^{13}C{^{1}H} NMR (100 MHz) spectra of **10** ($CDCl_3$)

3. HRMS Spectra of Compounds

HRMS spectrum of **3aa**

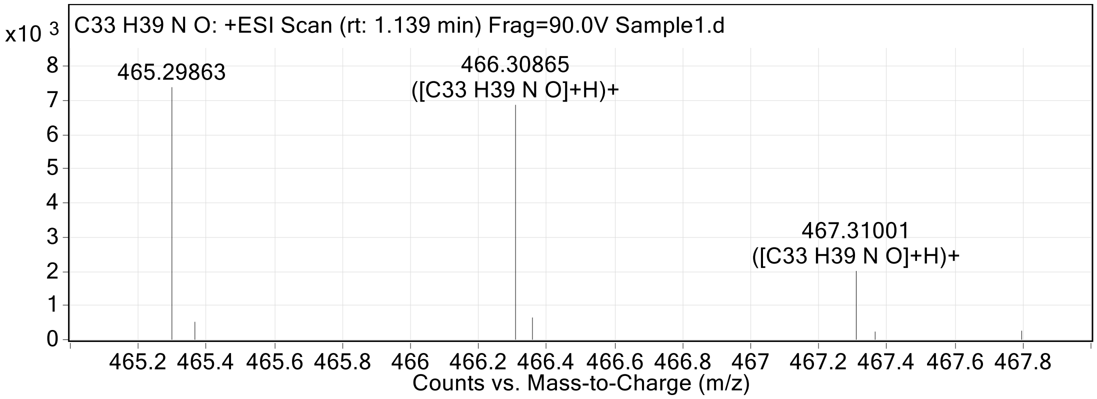

HRMS spectrum of **3ab**

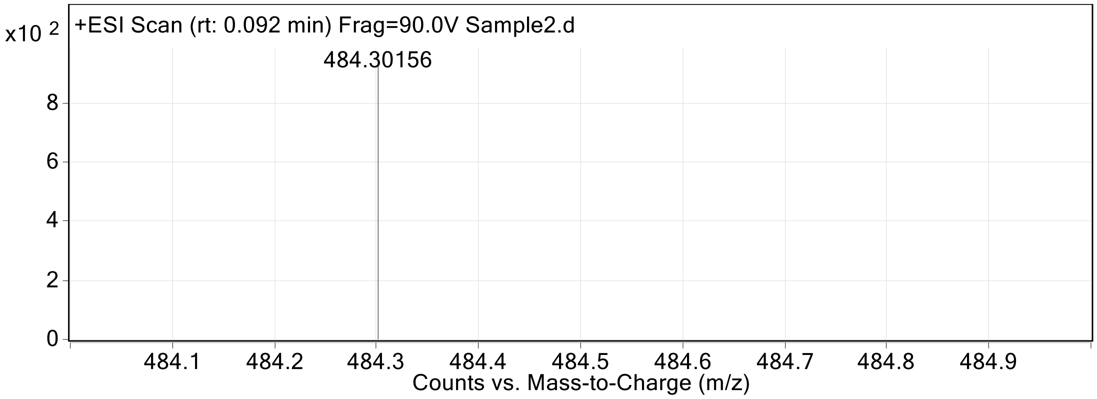

HRMS spectrum of **3ac**

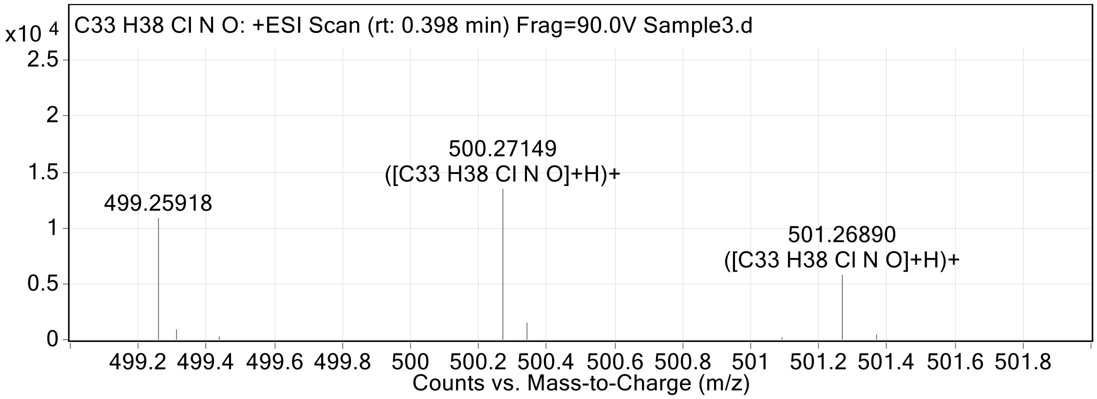

HRMS spectrum of **3ad**

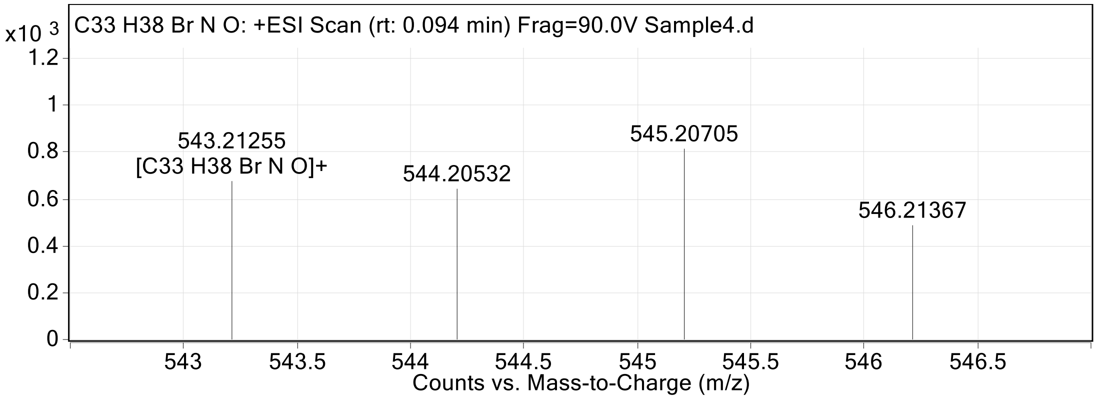

HRMS spectrum of **3ae**

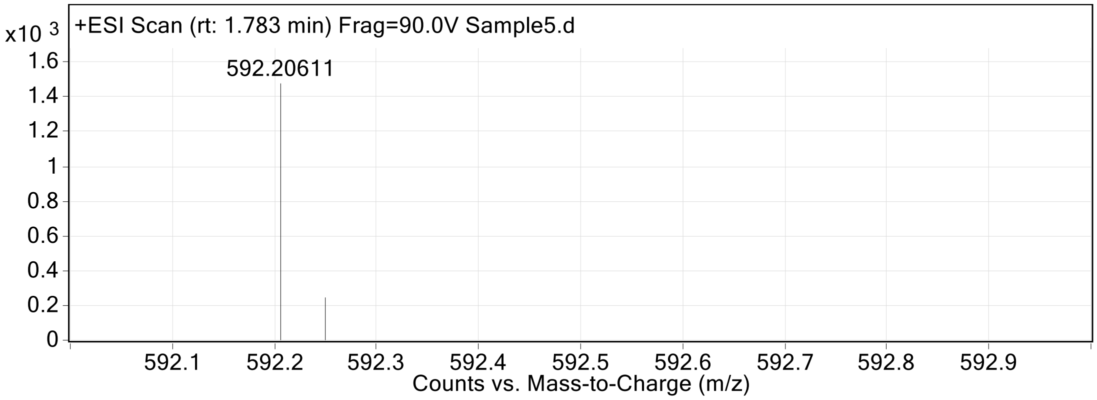

HRMS spectrum of **3af**

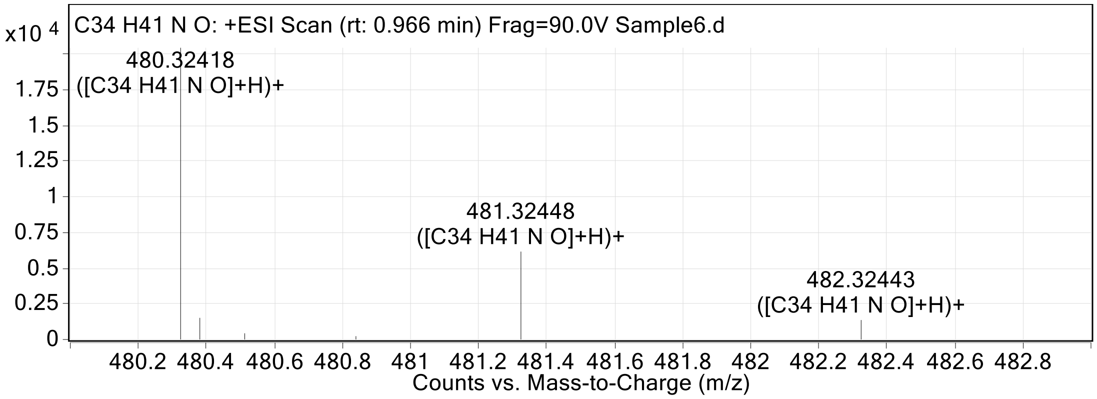

HRMS spectrum of **3ag**

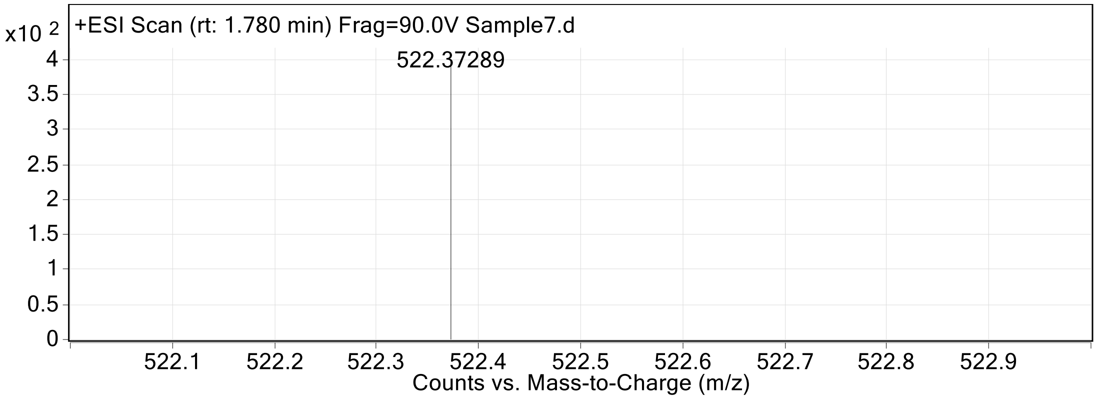

HRMS spectrum of **3ah**

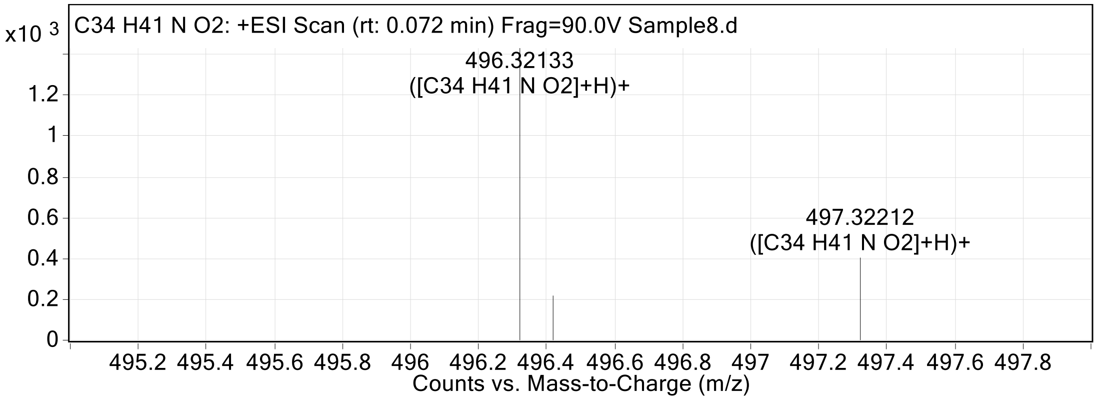

HRMS spectrum of **3ai**

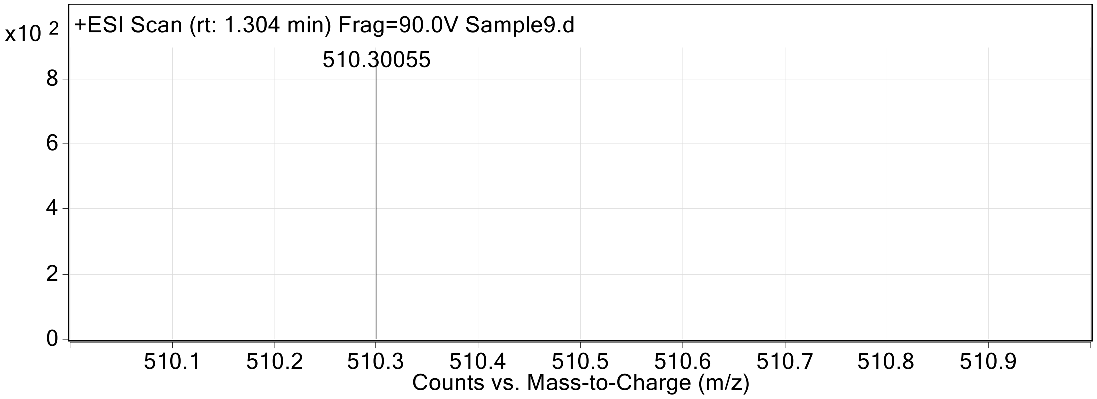

HRMS spectrum of **3aj**

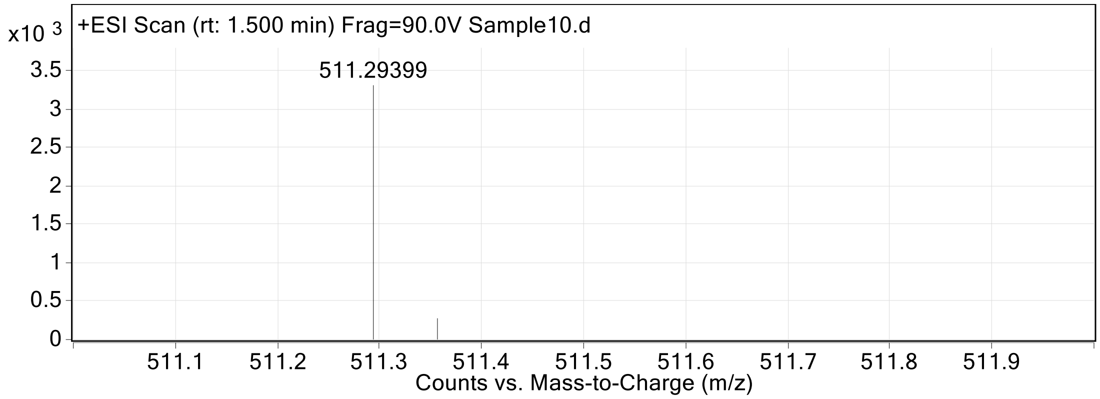

HRMS spectrum of **3ak**

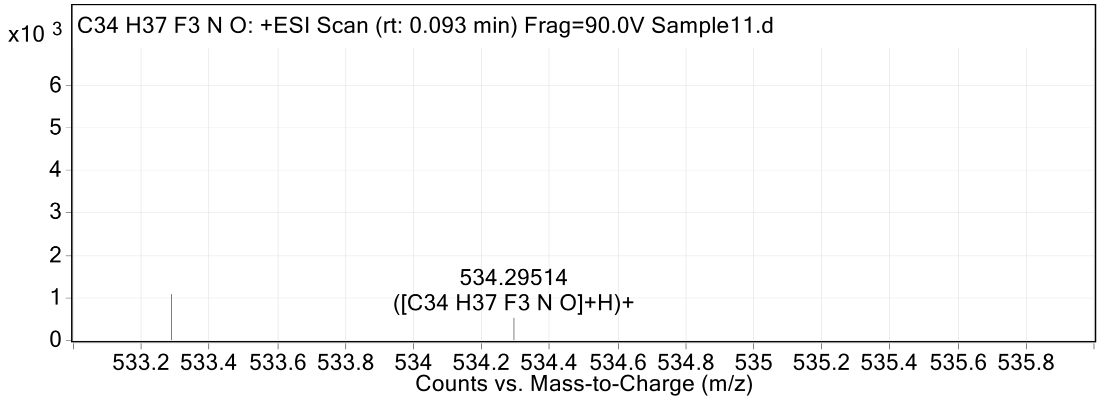

HRMS spectrum of **3al**

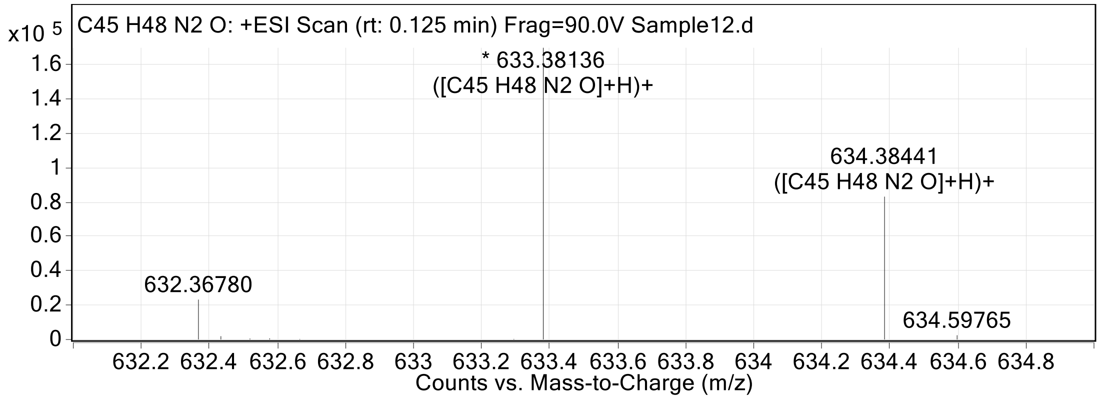

HRMS spectrum of **3am**

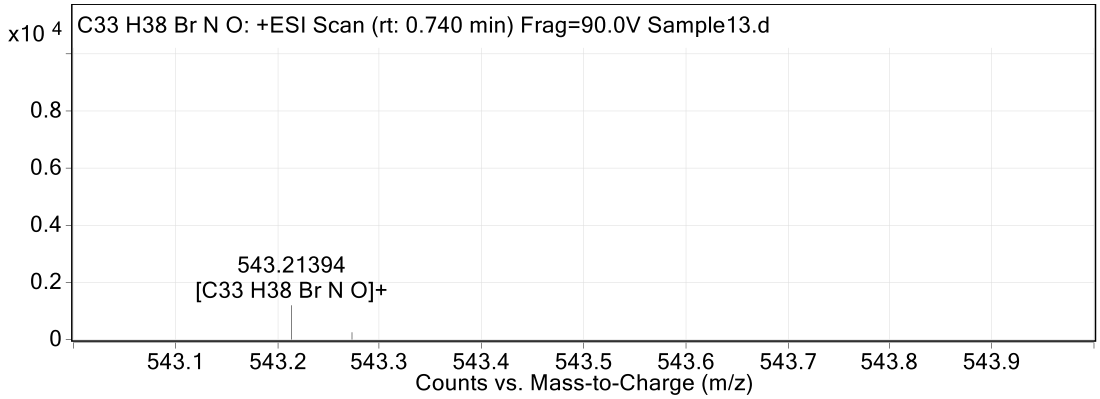

HRMS spectrum of **3an**

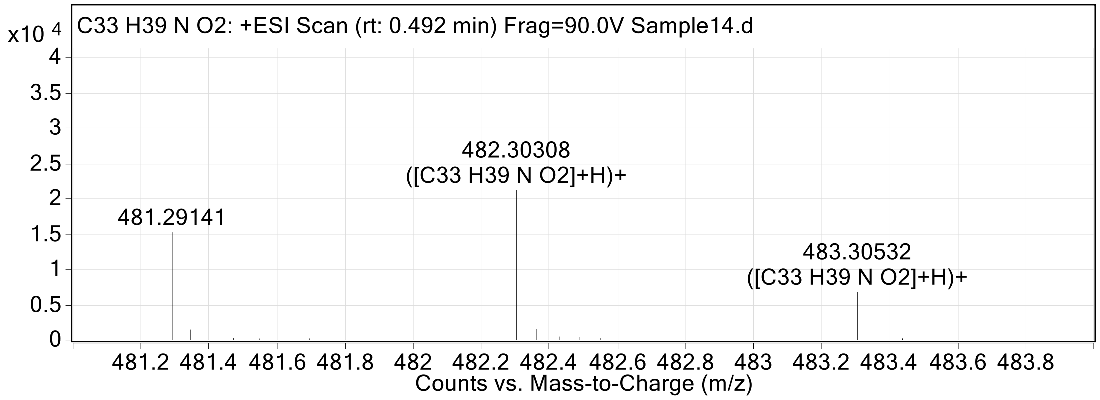

HRMS spectrum of **3ao**

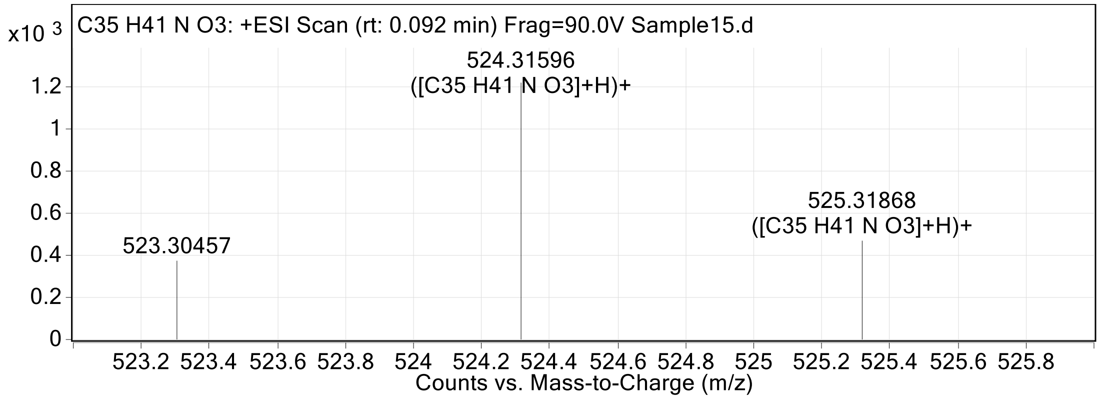

HRMS spectrum of **3ap**

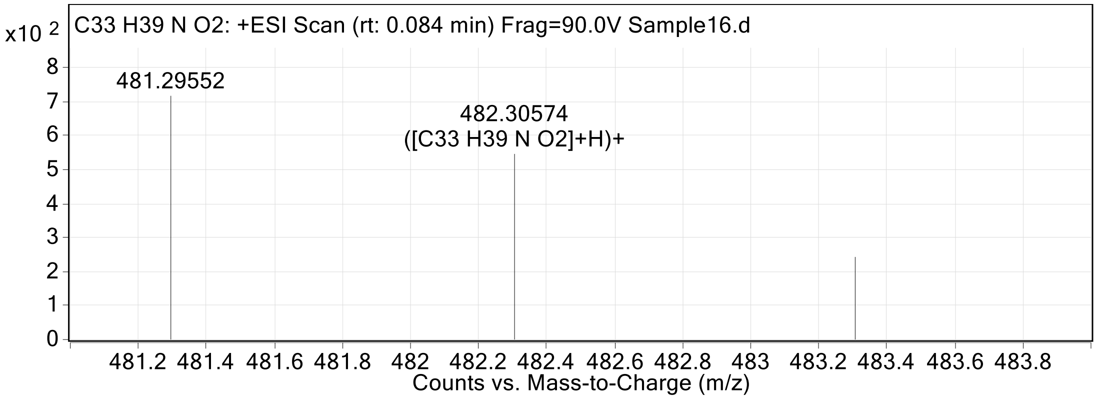

HRMS spectrum of **3aq**

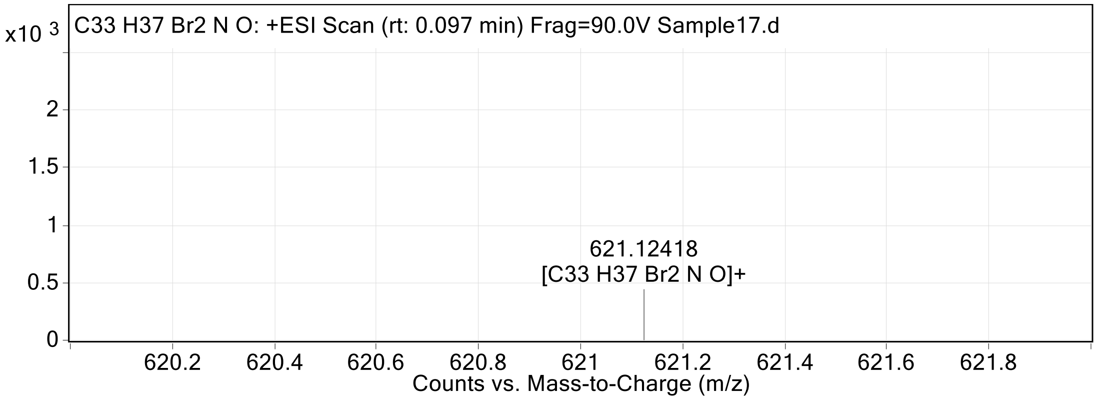

HRMS spectrum of **3ar**

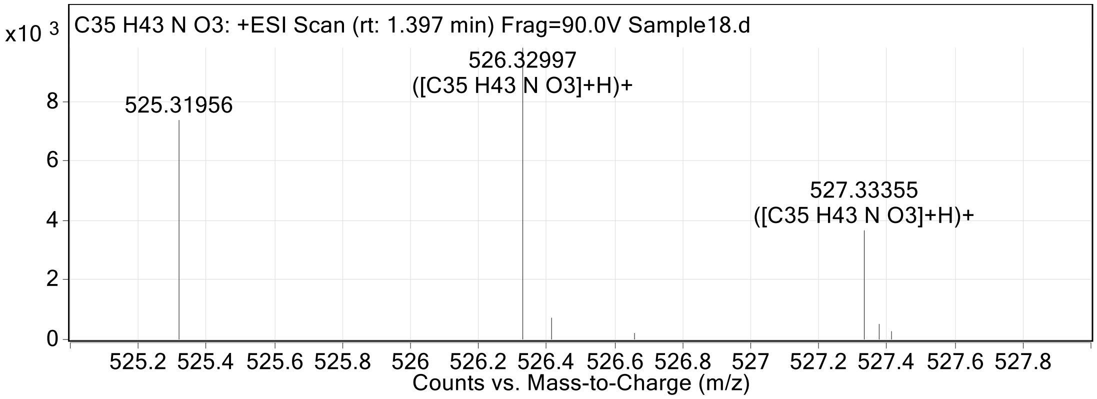

HRMS spectrum of **3as**

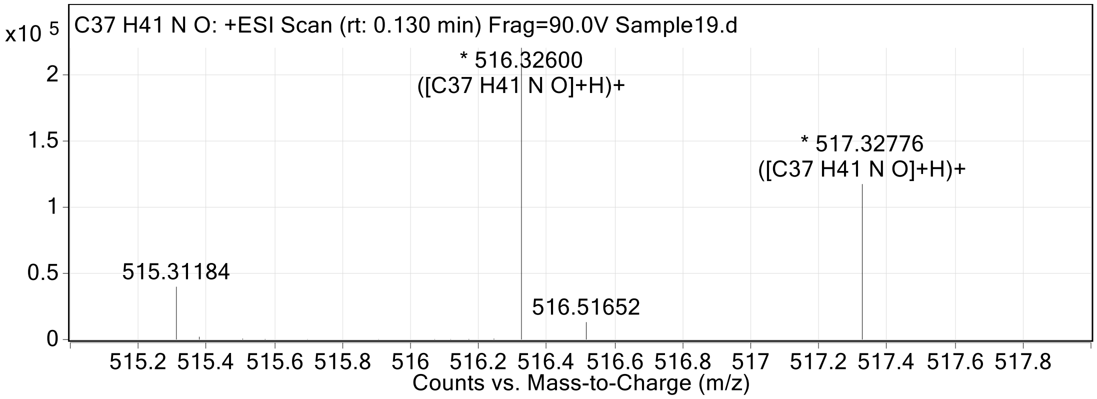

HRMS spectrum of **3au**

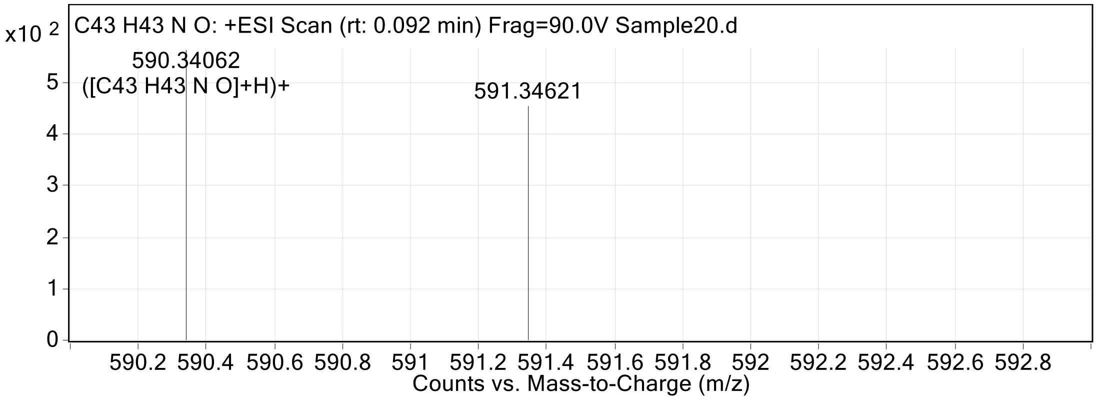

HRMS spectrum of **3av**

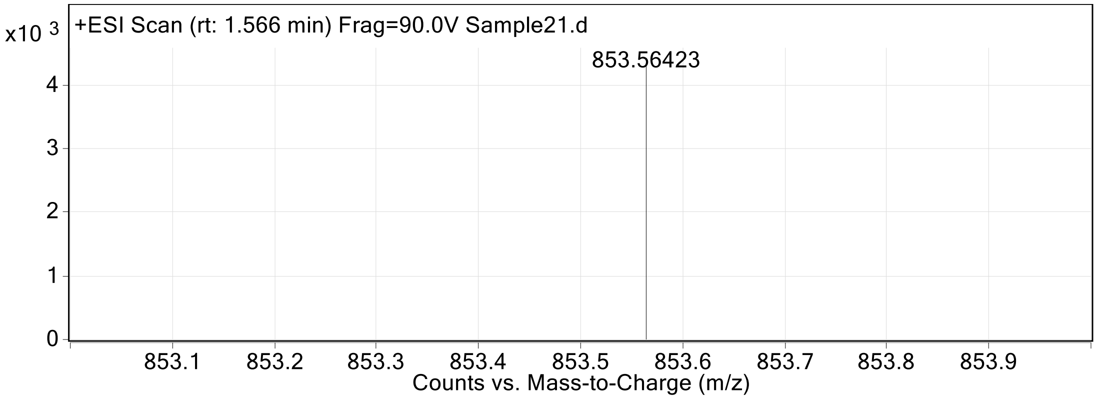

HRMS spectrum of **3ax**

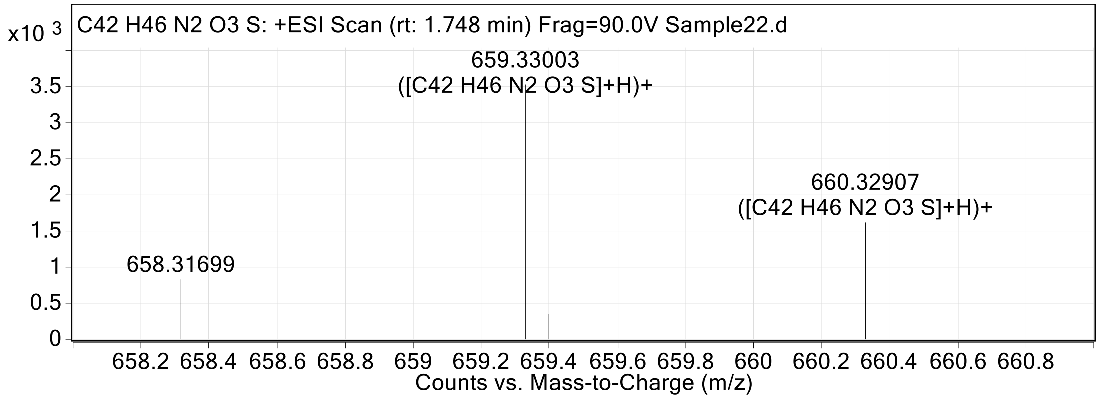

HRMS spectrum of **3bd**

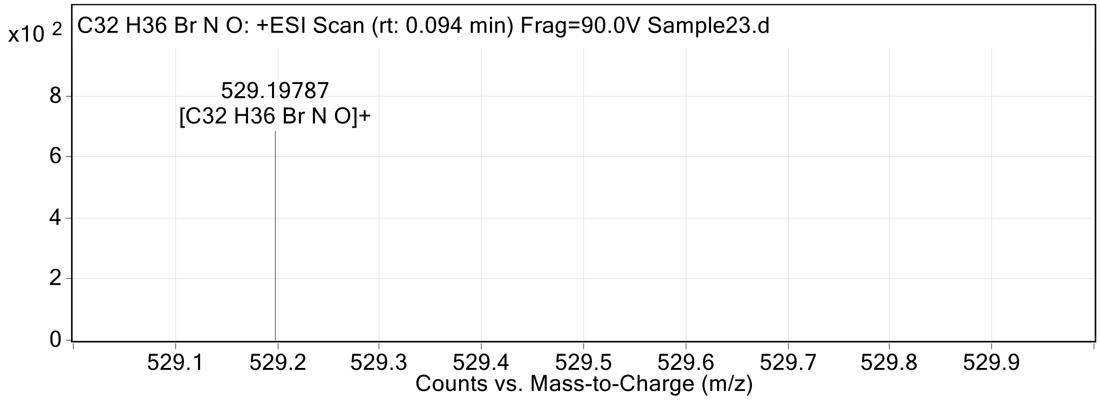

HRMS spectrum of **3cd**

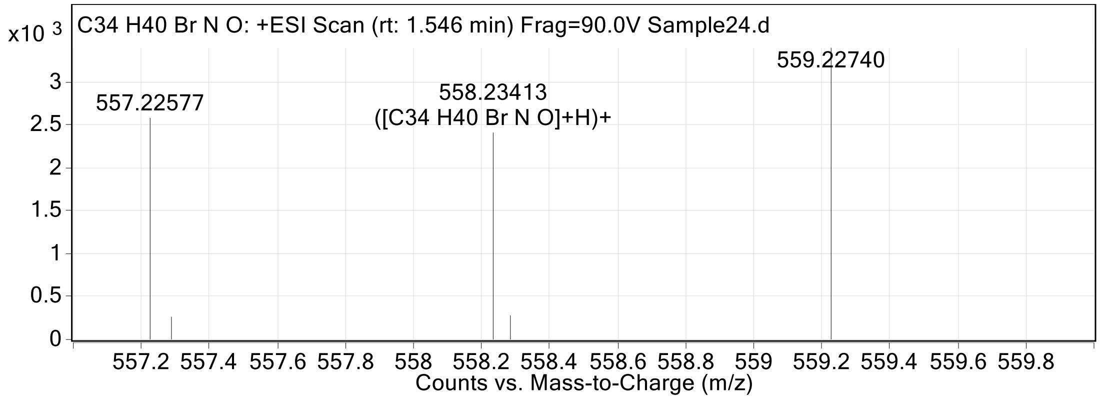

HRMS spectrum of **3dd**

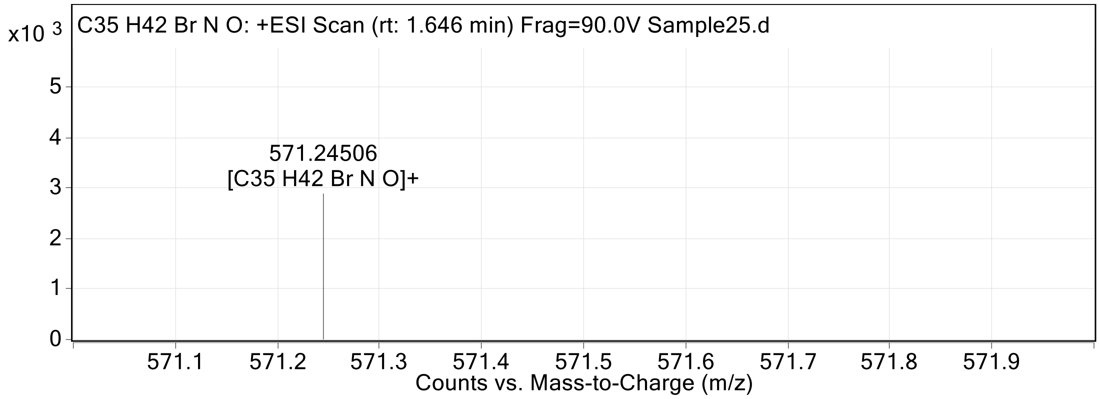

HRMS spectrum of **3ed**

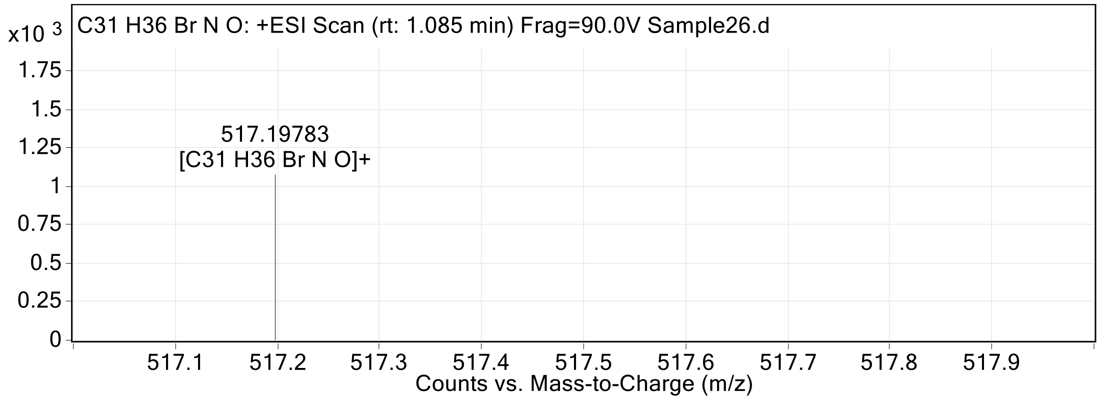

HRMS spectrum of **3fd**

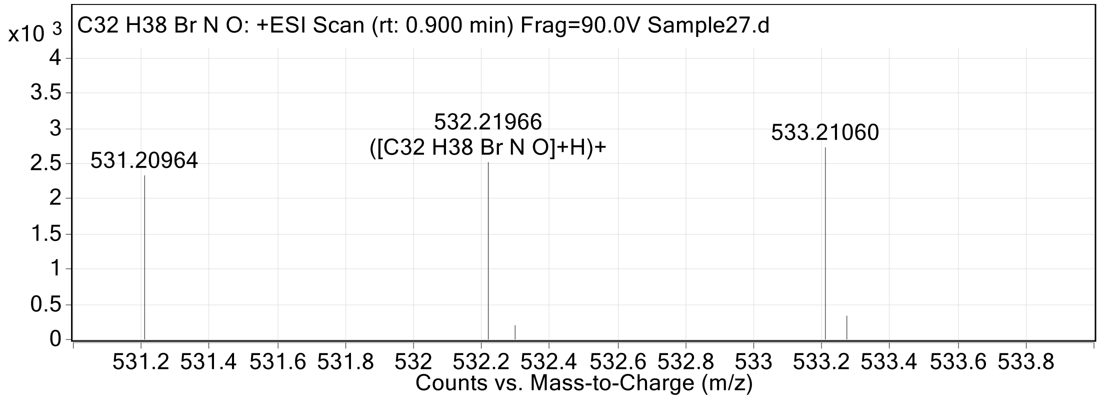

HRMS spectrum of **3gd**

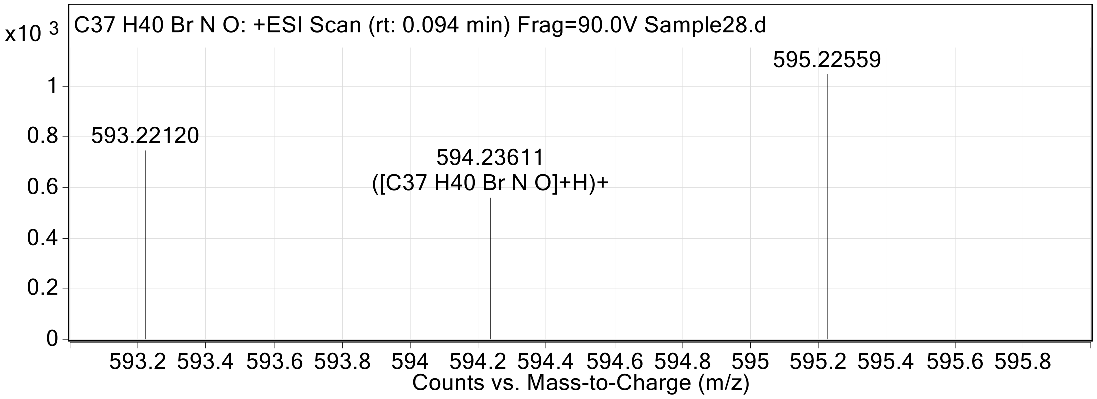

HRMS spectrum of **3hd**

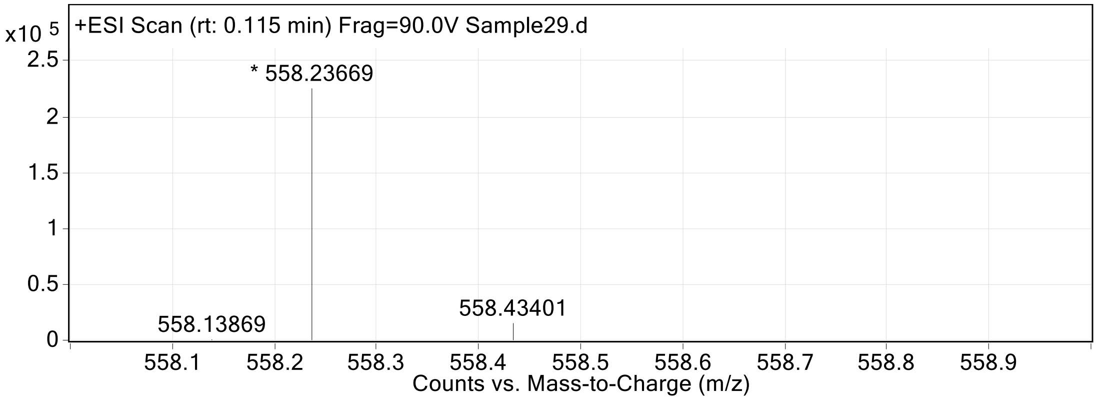

HRMS spectrum of **3id**

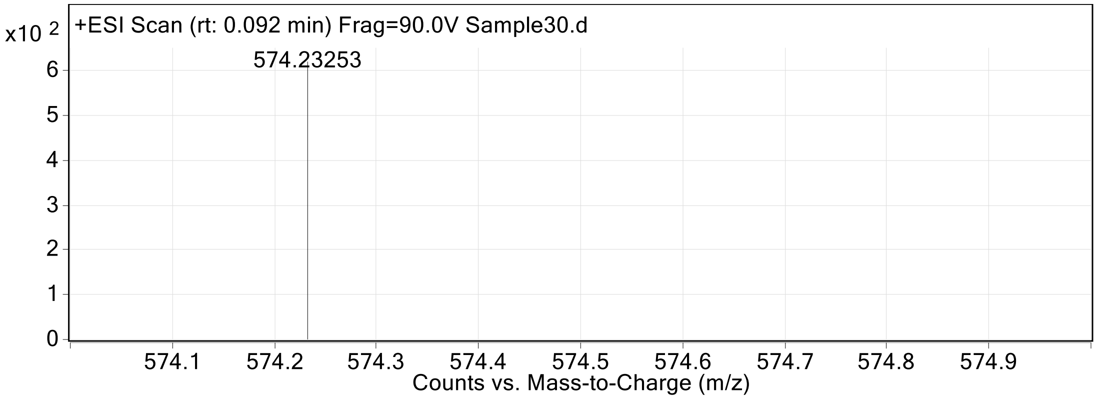

HRMS spectrum of **3jd**

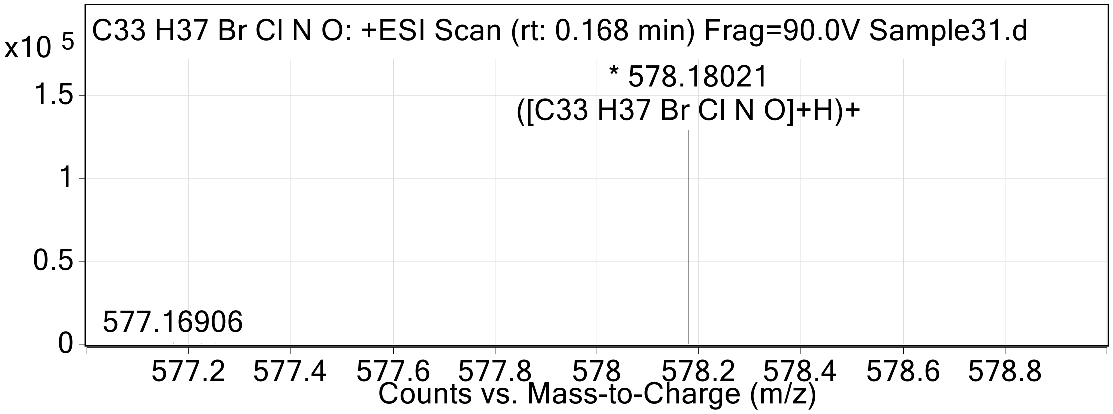

HRMS spectrum of **3kd**

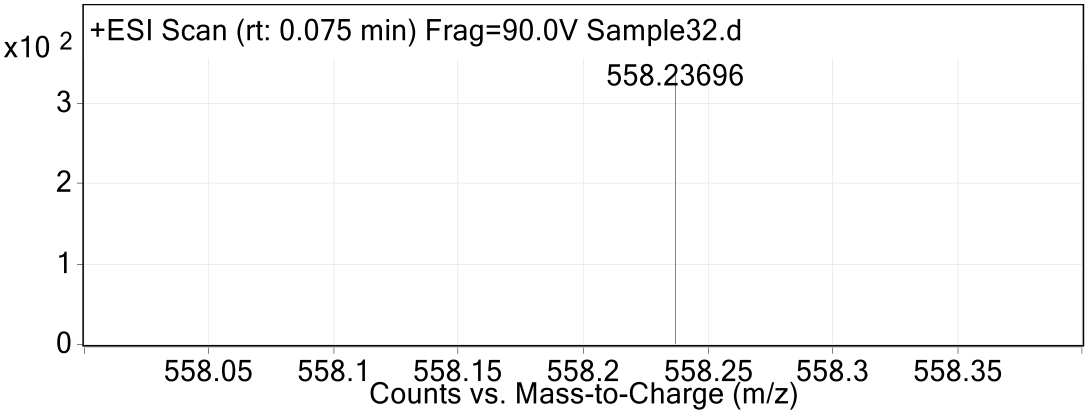

HRMS spectrum of **3ld**

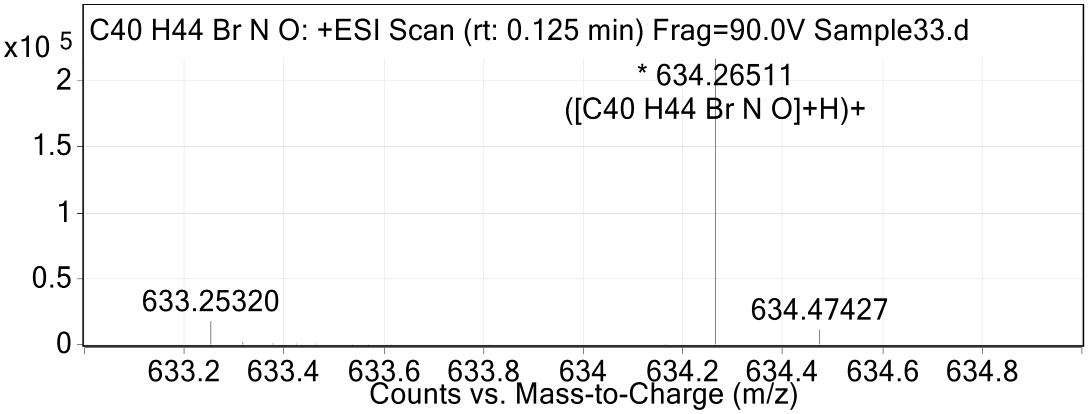

HRMS spectrum of **4aa**

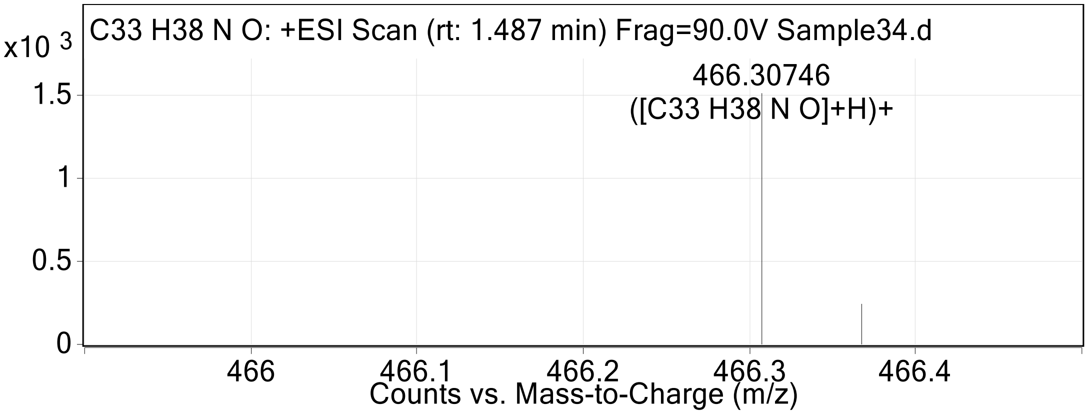

HRMS spectrum of **4ab**

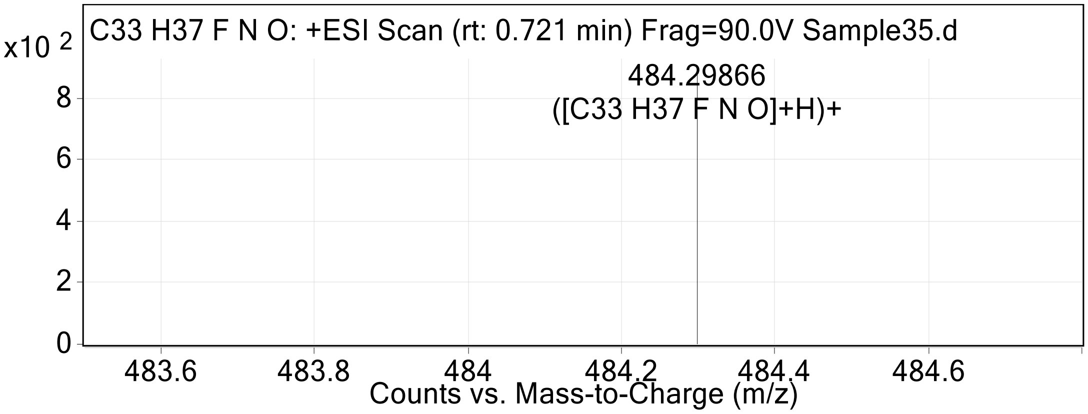

HRMS spectrum of **4ac**

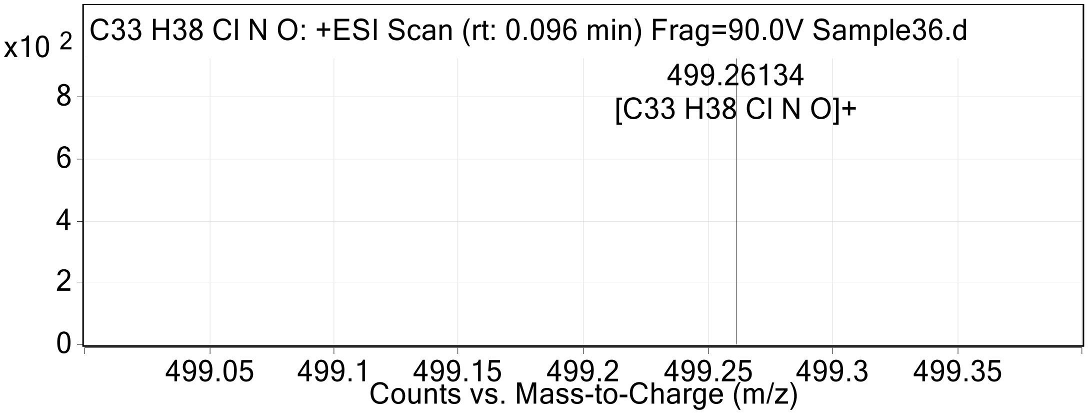

HRMS spectrum of **4ad**

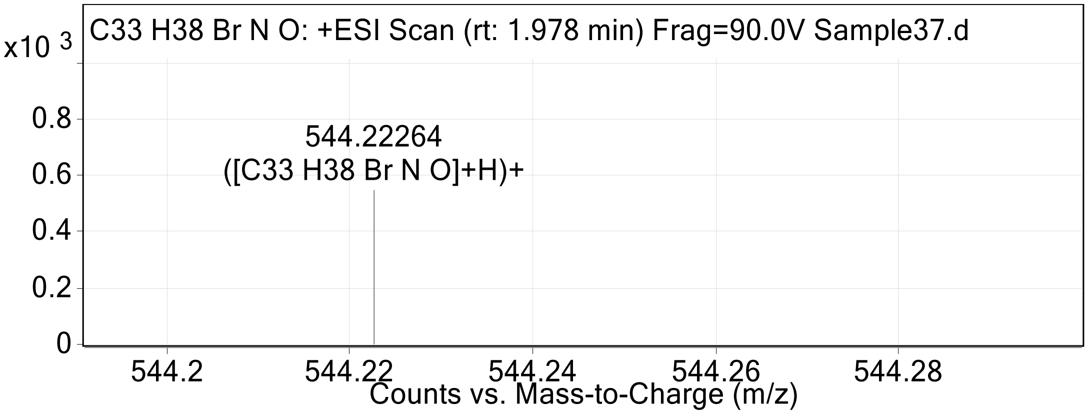

HRMS spectrum of **4af**

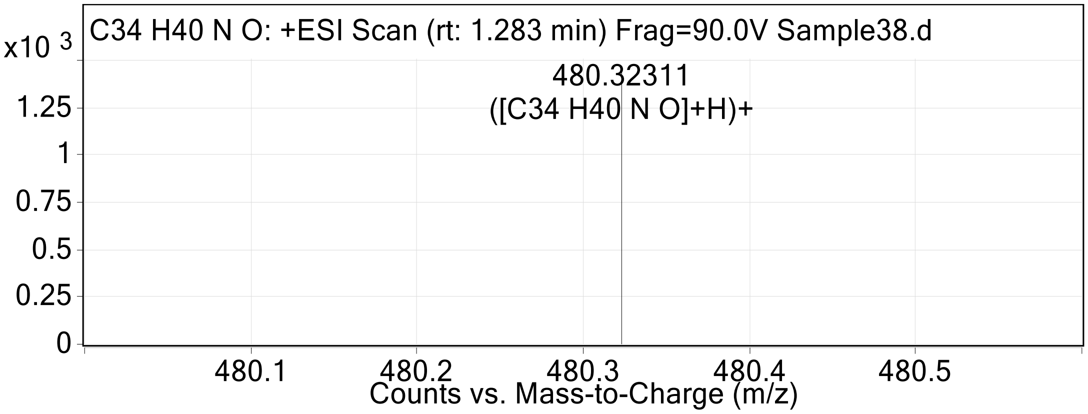

HRMS spectrum of **4ag**

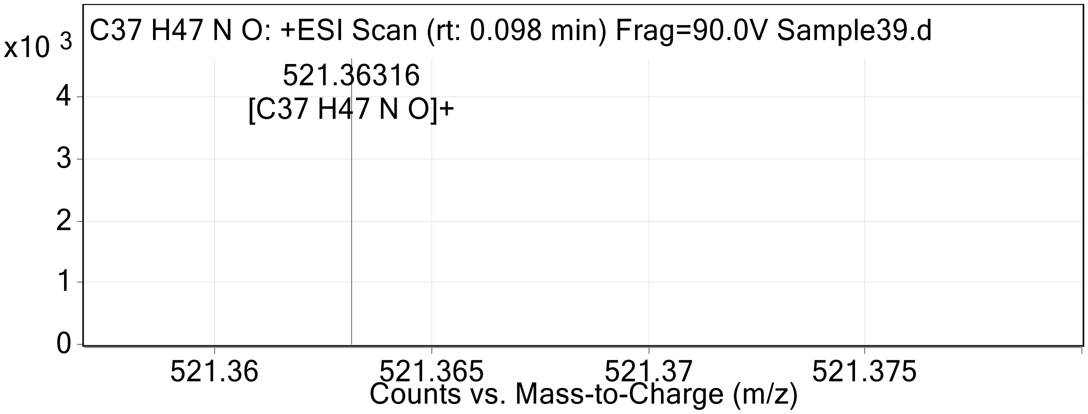

HRMS spectrum of **4ah**

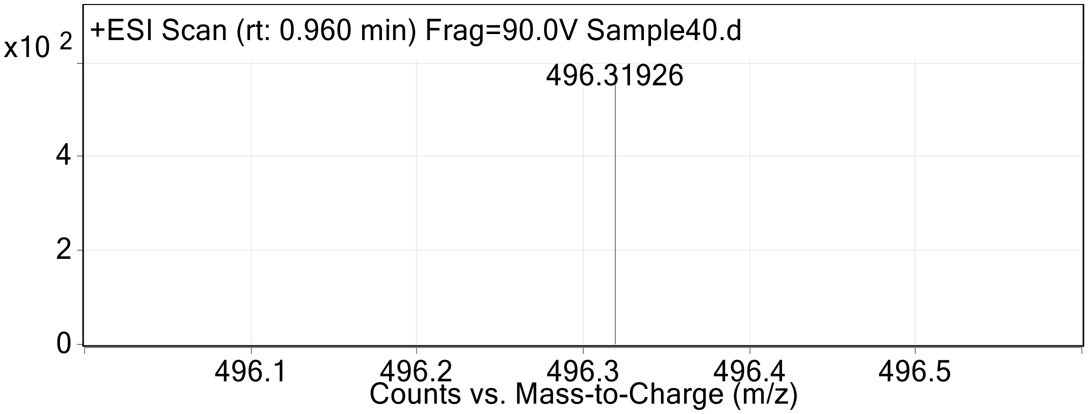

HRMS spectrum of **4aj**

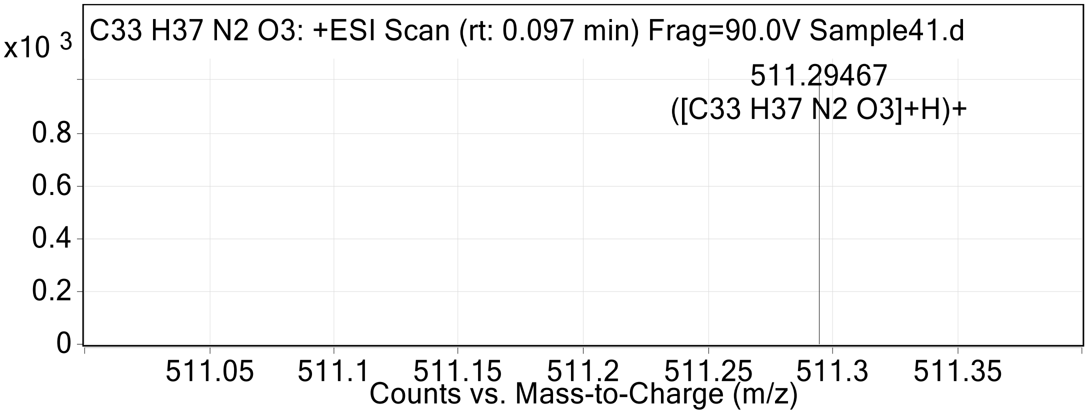

HRMS spectrum of **4ak**

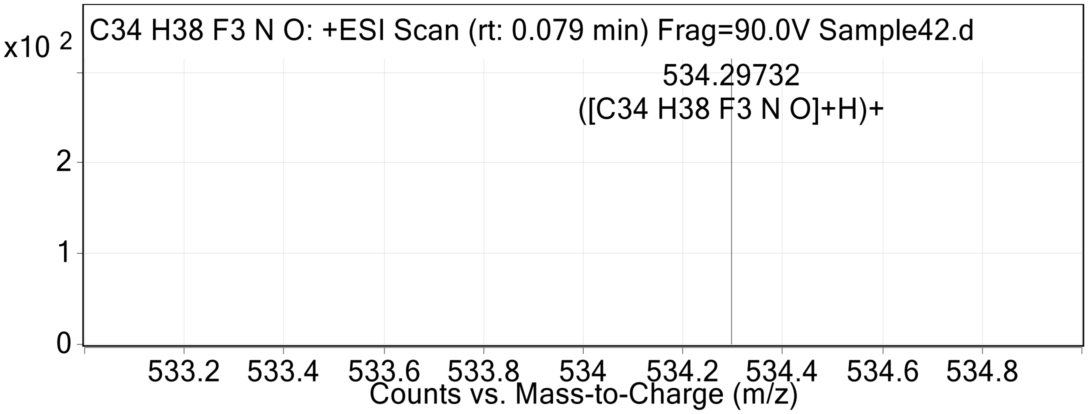

HRMS spectrum of **4al**

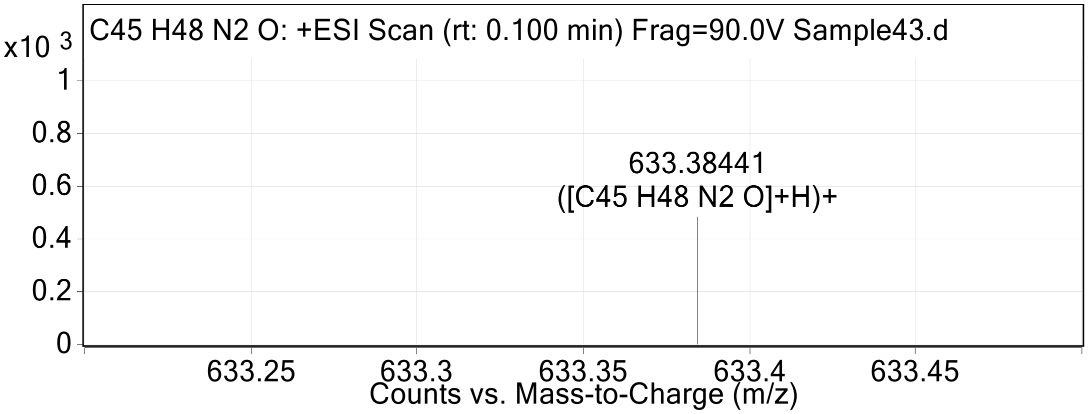

HRMS spectrum of **4am**

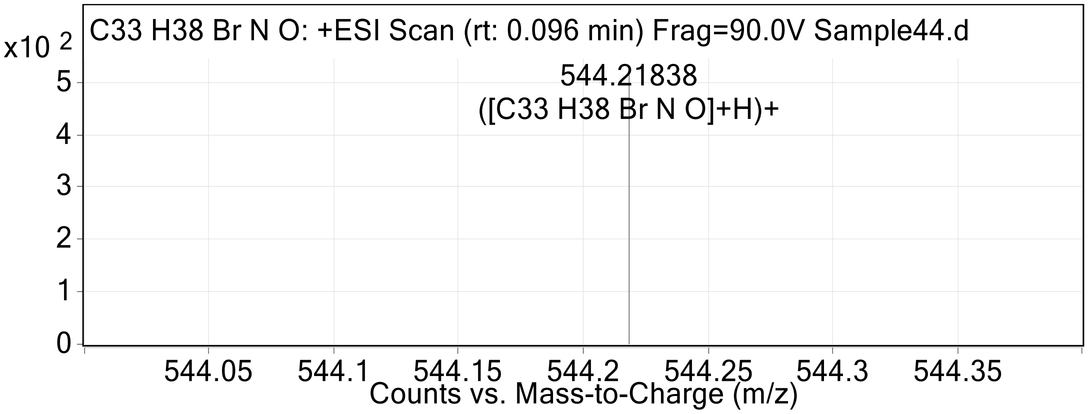

HRMS spectrum of **4an**

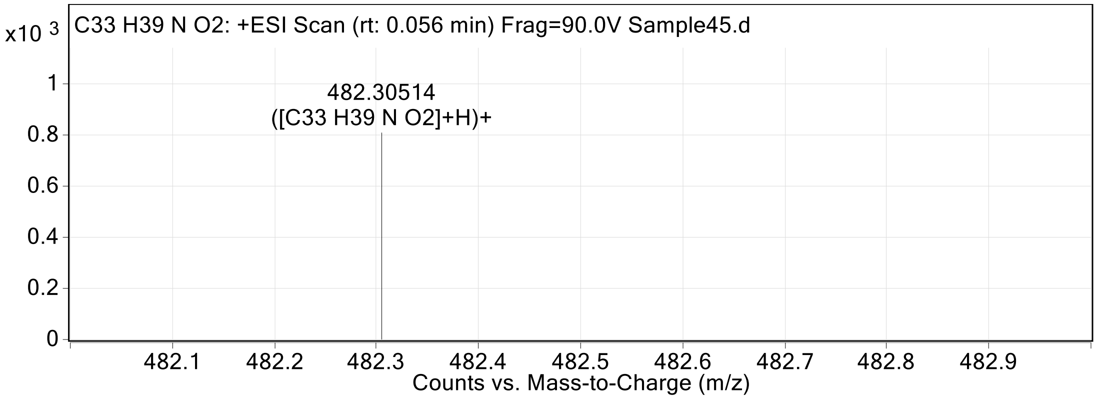

HRMS spectrum of **4ap**

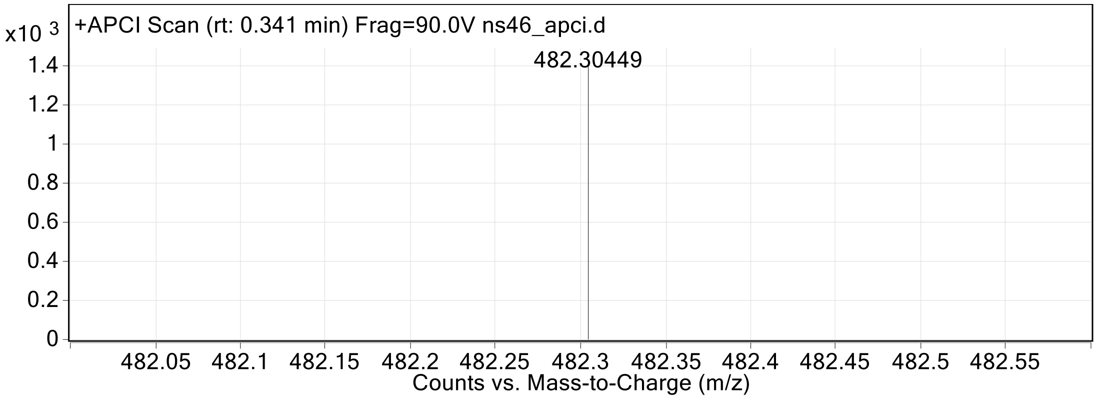

HRMS spectrum of **4aq**

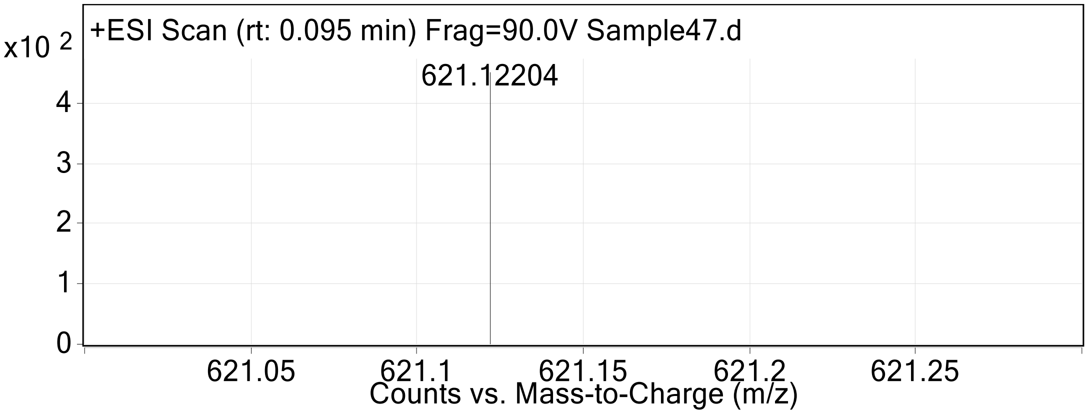

HRMS spectrum of **4as**

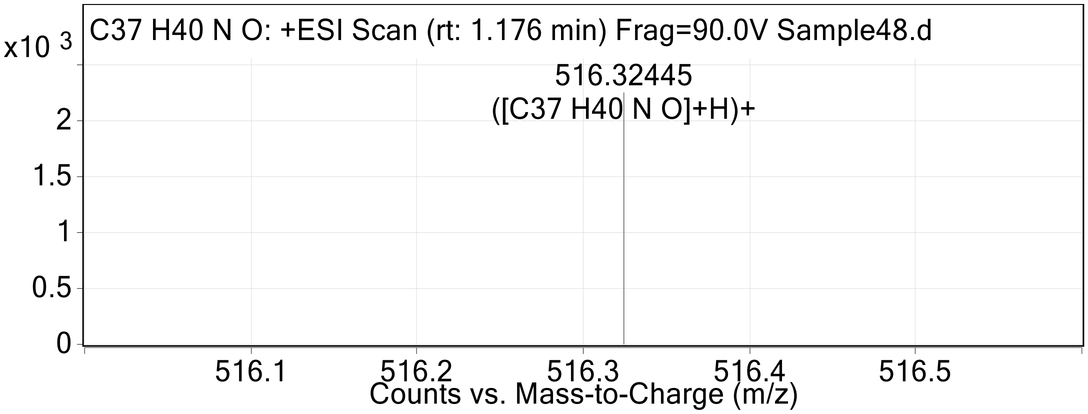

HRMS spectrum of **4au**

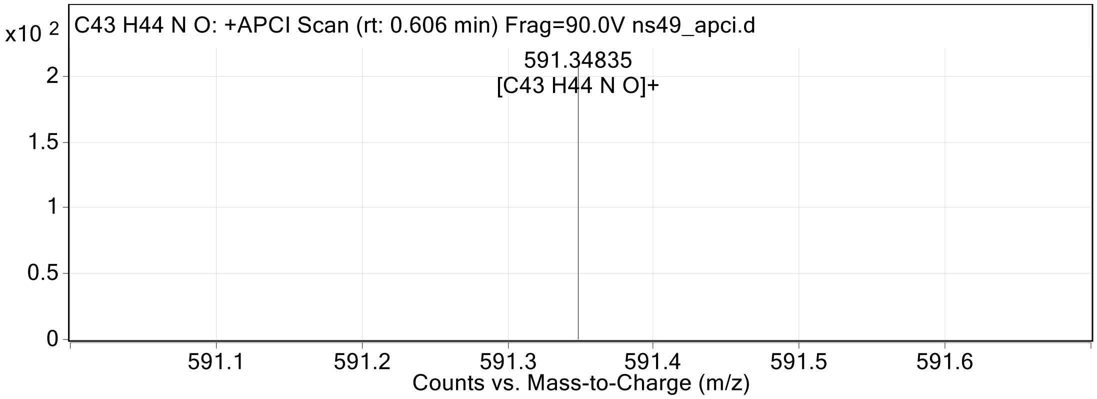

HRMS spectrum of **4av**

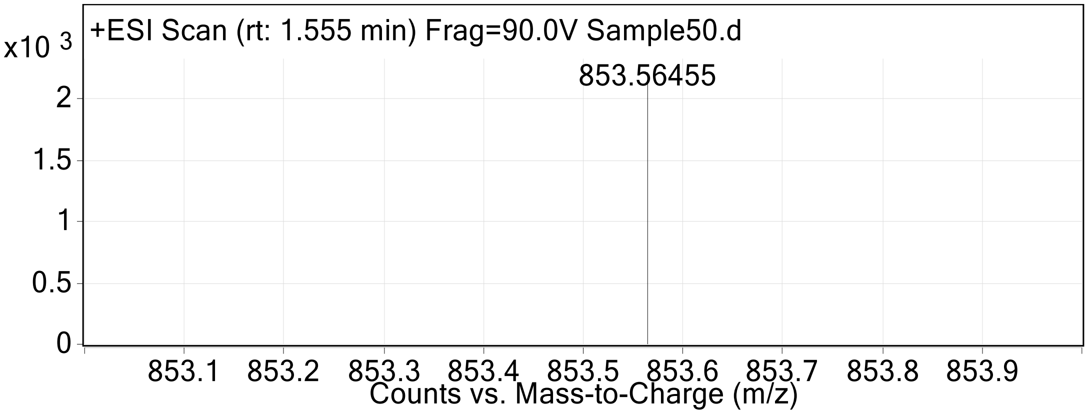

HRMS spectrum of **4bd**

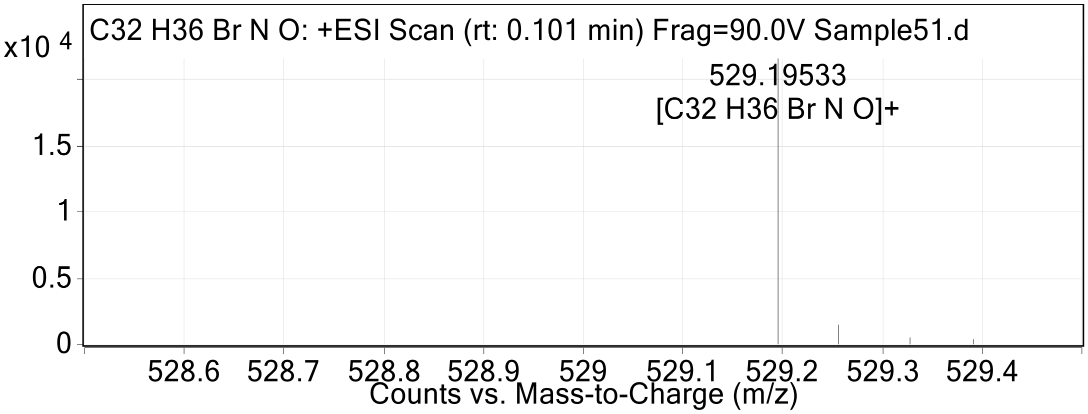

HRMS spectrum of **4cd**

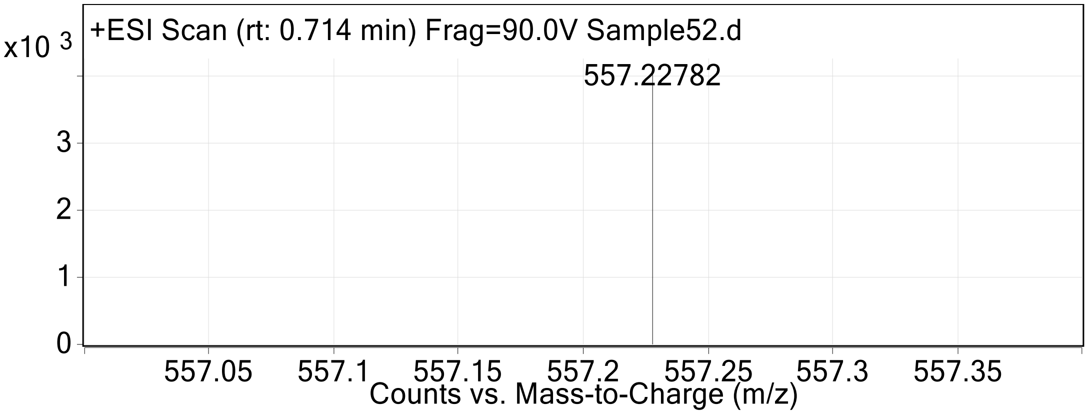

HRMS spectrum of **4dd**

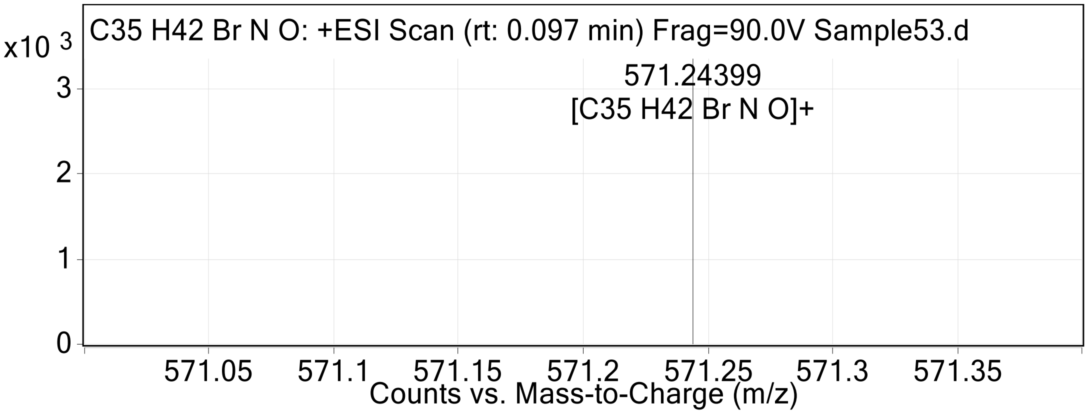

HRMS spectrum of **4ed**

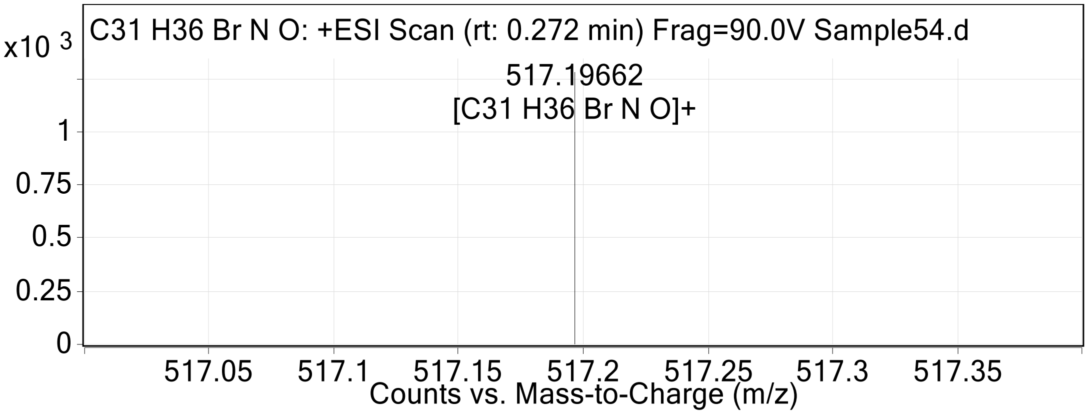

HRMS spectrum of **4fd**

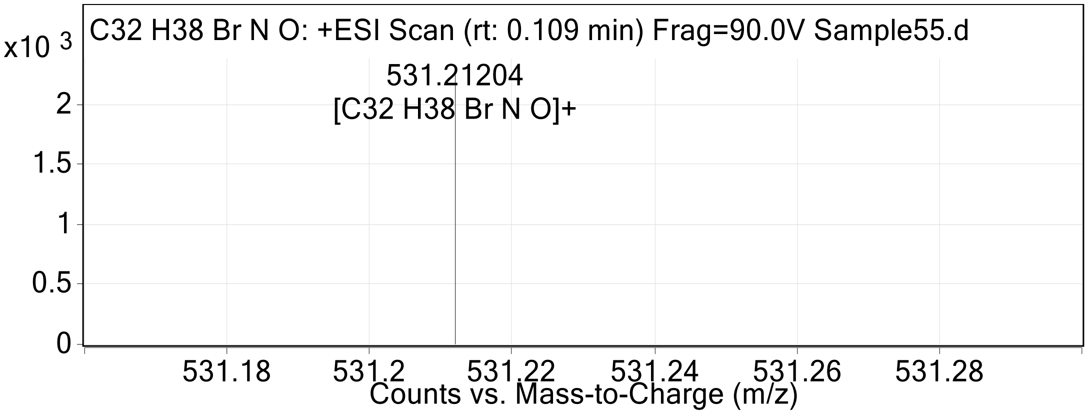

HRMS spectrum of **4hd**

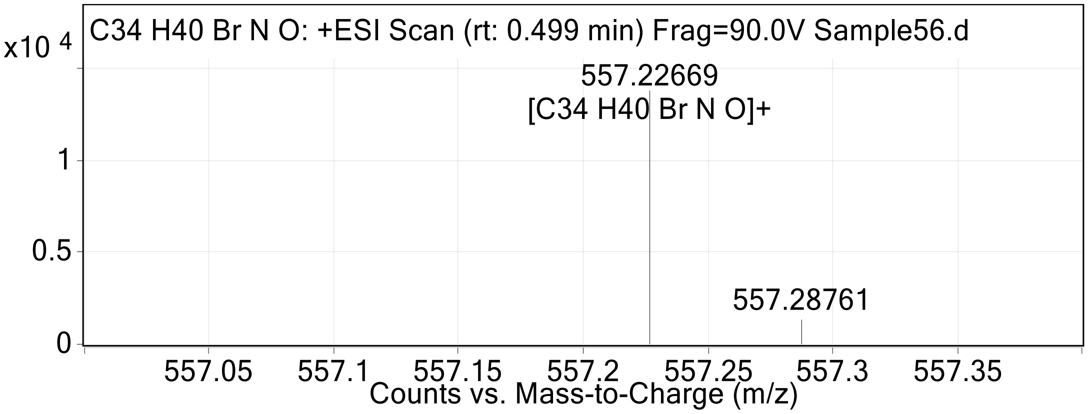

HRMS spectrum of **4id**

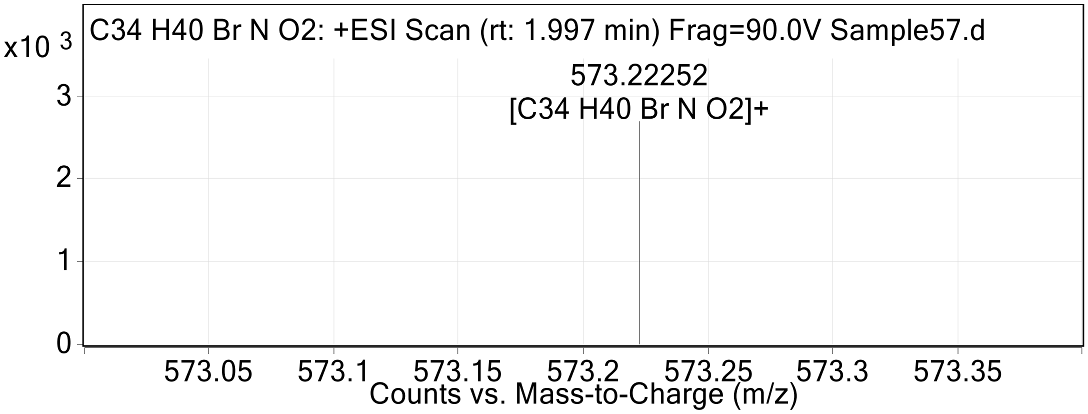

HRMS spectrum of **4jd**

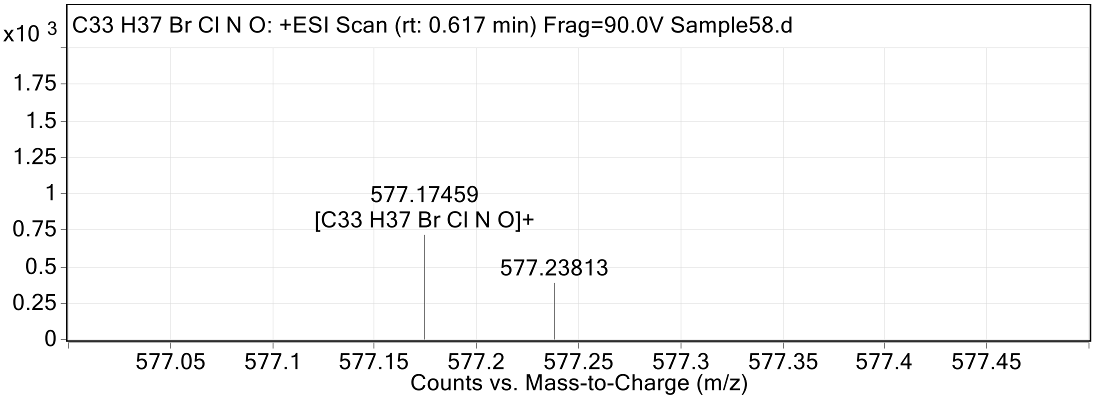

HRMS spectrum of **5**

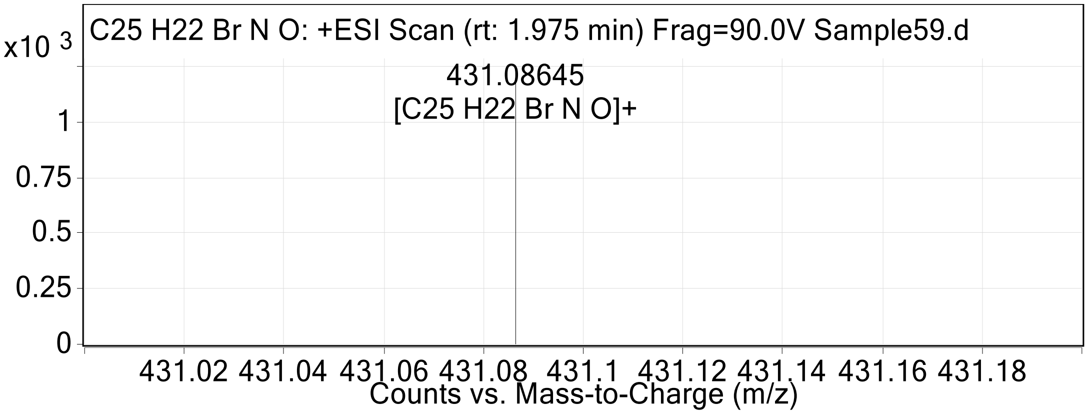

HRMS spectrum of **7**

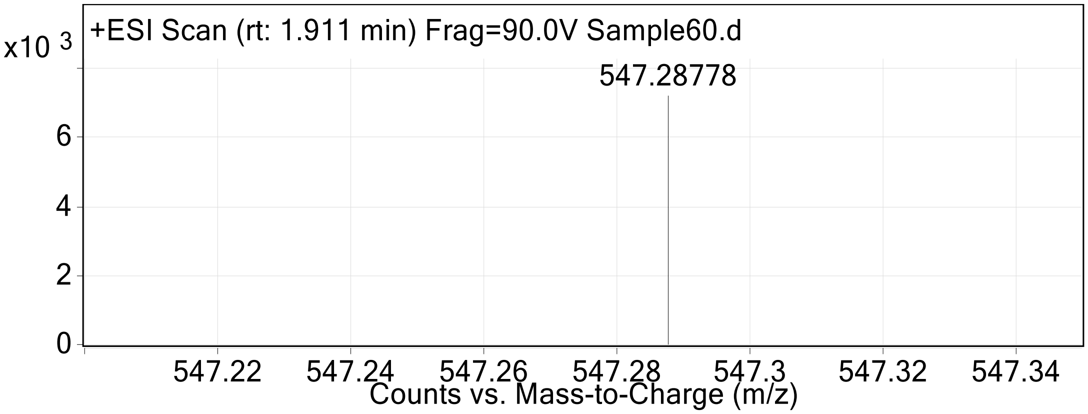

HRMS spectrum of **8d**

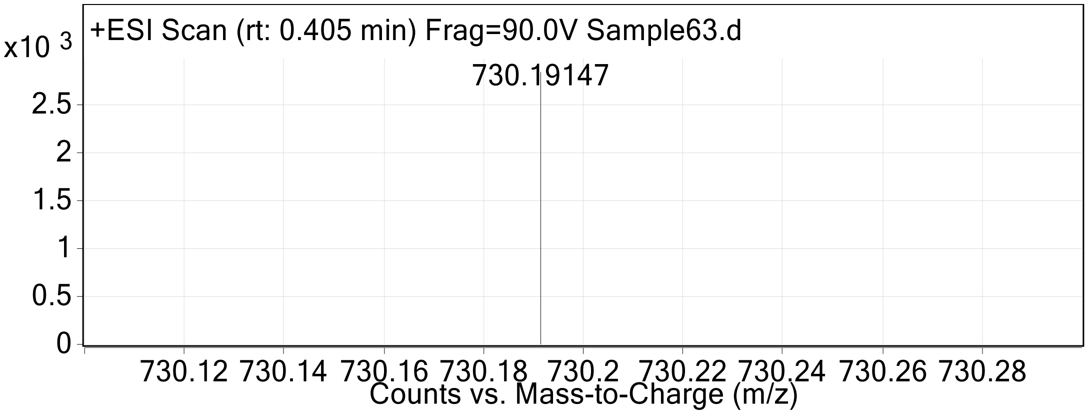

HRMS spectrum of **8f**

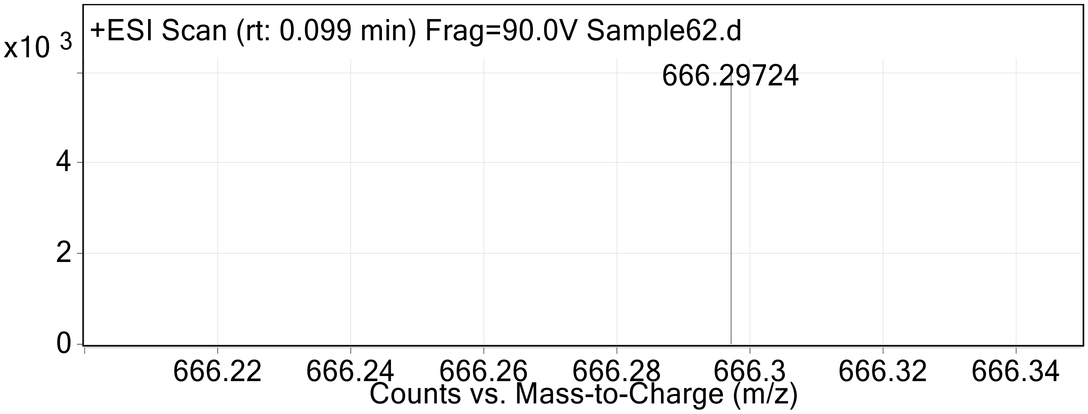

HRMS spectrum of **8k**

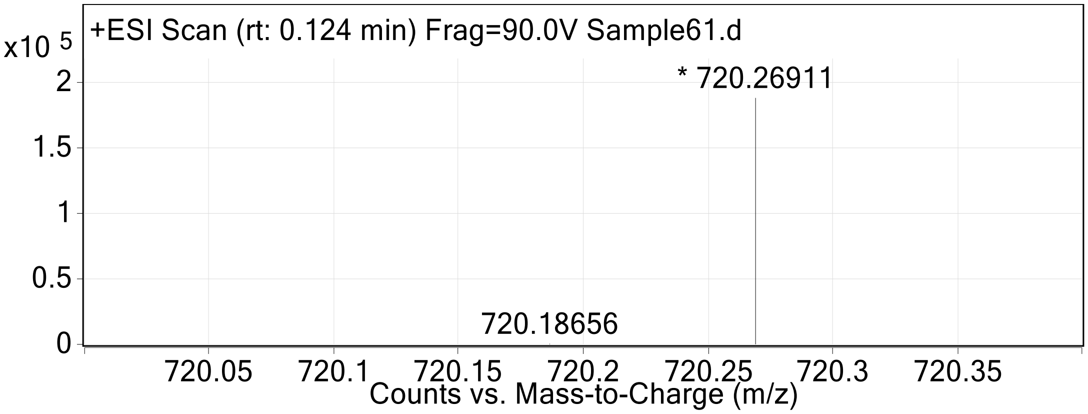

4. Computational Details

4.1. The Optimized Geometries of Computed Structures with Selected Interatomic Distances

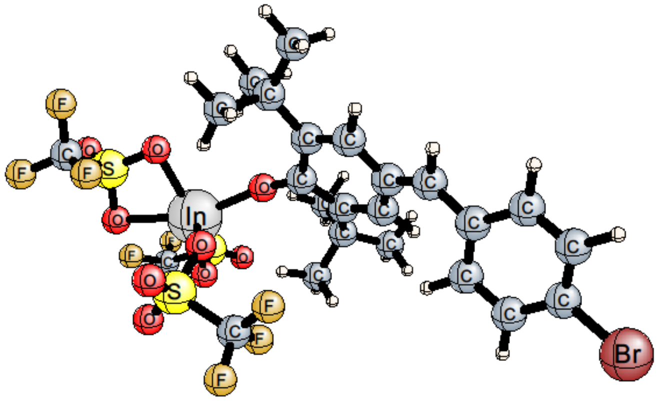

Figure S1. Computed structure of **H** at the B3LYP/def2-TZVP level.

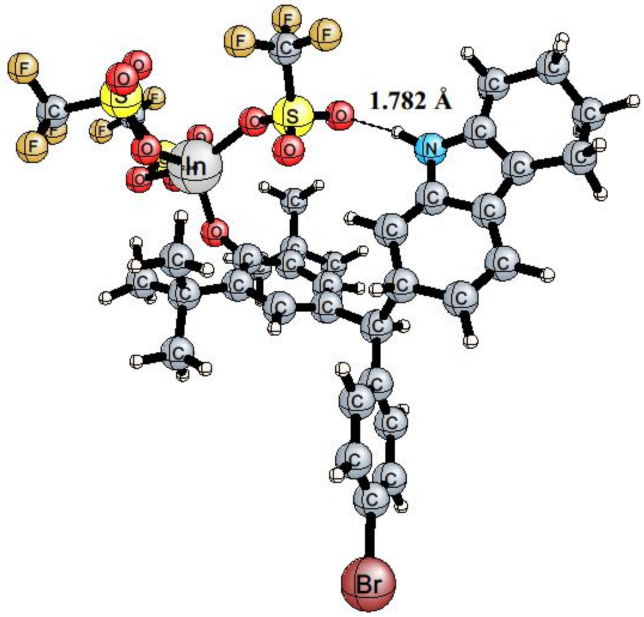

Figure S2. Computed structure of **D** at the B3LYP/def2-TZVP level.

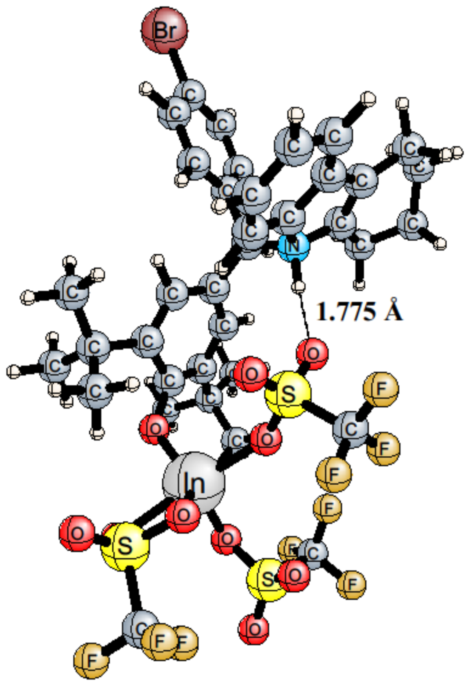

Figure S3. Computed structure of **B** at the B3LYP/def2-TZVP level.

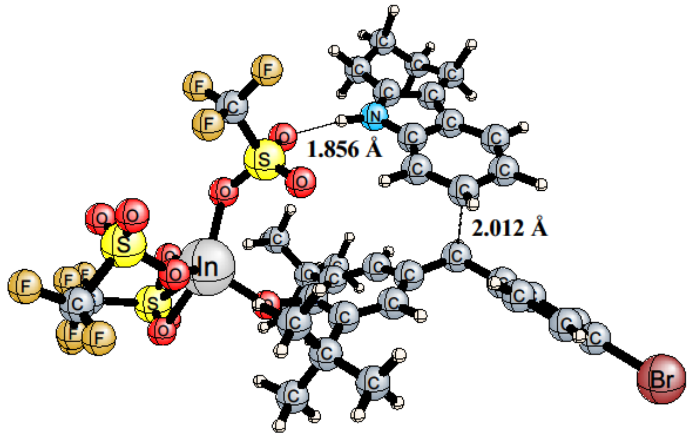

Figure S4. Computed structure of **C (TS2)** at the B3LYP/def2-TZVP level (im. freq.= 347*i*).

4.2. Single Point Energy Computations

Table S1. Single point energies (**E**) at B3LYP/def2-TZVP level in a.u.

**Molecule**	**E** (Toluene)	**E** (THF)
**In(OTf)$_3$**	-3075.5622895	-3075.5661853
**1a**	-520.0825504	-520.0834205
**2d**	-3465.0367248	-3465.0358353

**3ad**	-3985.1297260	-3985.1298317
**4ad**	-3985.1191319	-3985.1182491
**H**	-6540.6172957	-6540.6238845
**D**	-7060.6895584	-7060.6980083
**B**	-7060.6768654	-7060.6850592
**C (TS2)**	-7060.6772273	-7060.6834348

4.3. Cartesian Coordinates for Optimized Structures

In(OTf)$_3$

E= -3075.5576129 a.u., number of negative frequencies = 0

0 1

In 0.22887200 -0.27655100 -0.06745600

O 2.06370200 -0.51019900 -1.27289400

S 2.86621900 -1.19292400 -0.20135700

O 3.50370600 -2.41844000 -0.53067700

O 1.94486000 -1.18069700 0.98585000

C 4.21229800 0.03261000 0.23253300

F 4.89908300 -0.43054500 1.26861500

F 3.66811100 1.20455400 0.54414300

F 5.01659500 0.17712600 -0.81220500

O -0.74235600 1.30494900 -1.26247600

S -0.30421400 2.44784700 -0.39137200

O 0.35159000 3.54229900 -1.01544600

O 0.39918100 1.76862400 0.74865900

C -1.88753400 3.10750600 0.35594100

F -1.58765500 4.05235200 1.23700900

F -2.54249600 2.12206700 0.96305000

F -2.64204000 3.61547300 -0.60966500

O -1.39405900 -1.00768000 1.23536300

S -1.77665900 -2.17423300 0.36825100

O	-1.85060300	-3.45490300	0.97728600
O	-0.89176900	-2.01956300	-0.83525200
C	-3.49254900	-1.75877300	-0.25145300
F	-3.48141700	-0.56545900	-0.83922700
F	-3.87133300	-2.68187500	-1.12485600
F	-4.33080000	-1.74071500	0.77664300

1a

E= -520.0671955 a.u., number of negative frequencies = 0

0 1

C	3.14835500	1.15527500	0.04178700
C	3.55172100	-0.18833900	-0.00028100
C	2.62075700	-1.21558600	-0.03424000
C	1.27330100	-0.86713200	-0.02484000
C	0.84173900	0.48472500	0.01876200
C	1.80634200	1.49896700	0.05176200
N	0.14345700	-1.65587300	-0.05143200
C	-0.97759400	-0.84756600	-0.02252800
C	-0.59492000	0.46488300	0.01545400
C	-2.38165500	-1.35137600	-0.04427000
C	-3.34119000	-0.21626100	0.34994200
C	-2.98029600	1.10259100	-0.34399400
C	-1.57781600	1.59556800	0.04712300
H	3.90134300	1.93288600	0.06717500
H	4.60761700	-0.42735400	-0.00688900
H	2.93557500	-2.25180800	-0.06738900
H	1.50896100	2.54014700	0.08561100
H	0.13404500	-2.65979300	-0.06791600
H	-2.50211100	-2.20084900	0.63634900
H	-2.63456800	-1.72289200	-1.04547500

H	-4.36716200	-0.50278800	0.10831300
H	-3.29963700	-0.07410200	1.43449600
H	-3.02028900	0.95678800	-1.42867800
H	-3.72421100	1.86541000	-0.10231200
H	-1.26584300	2.39698100	-0.62941100
H	-1.61038100	2.04398500	1.04808800

2d

E= -3465.0157999 a.u., number of negative frequencies = 0

0 1

C	-3.26907300	-1.00350400	0.03653400
C	-2.05803100	-1.59221100	-0.04457300
C	-0.81040800	-0.86046100	-0.07831900
C	-0.88819600	0.58117500	-0.12863000
C	-2.05560300	1.25787500	-0.07316400
C	-3.32778100	0.48619800	0.07339900
O	-4.40051800	1.06928100	0.20236700
C	-2.11955400	2.78851300	-0.16494500
C	-4.57407600	-1.80833900	0.10242300
C	-4.31303000	-3.32118000	0.02167500
C	-5.30240800	-1.53062300	1.43675200
C	-5.49069500	-1.43001900	-1.08264900
C	-0.72588500	3.40686900	-0.36205200
C	-2.99060600	3.21387300	-1.36841200
C	-2.70810400	3.37236500	1.13982600
C	0.35307600	-1.57448100	-0.09923100
C	1.73871300	-1.13038400	-0.03549600
C	2.71798600	-1.90560100	-0.67901300
C	4.05635900	-1.54917100	-0.66174300
C	4.44420000	-0.41068200	0.03384900

C	3.50984300	0.35819200	0.71593300
C	2.17141000	-0.00232000	0.67995800
Br	6.28406200	0.08573900	0.07615600
H	-1.97398600	-2.67050700	-0.05971300
H	0.03645200	1.11874900	-0.26363200
H	-5.26654100	-3.85019100	0.06141100
H	-3.82003200	-3.60430100	-0.91104000
H	-3.70612500	-3.67973600	0.85592600
H	-6.21659000	-2.12718400	1.48608600
H	-5.56926300	-0.48166700	1.53371700
H	-4.67455700	-1.81143900	2.28579700
H	-4.99716000	-1.63654600	-2.03541900
H	-6.40447400	-2.02779300	-1.04412400
H	-5.76596700	-0.37909000	-1.05423300
H	-0.82391500	4.49142100	-0.43019400
H	-0.05774000	3.19307500	0.47515600
H	-0.24787100	3.06269500	-1.28175500
H	-3.00458400	4.30382200	-1.44229800
H	-4.01400600	2.86358800	-1.26463100
H	-2.57950700	2.82199200	-2.30190400
H	-2.09249600	3.09638200	1.99944100
H	-2.72389200	4.46318400	1.07749200
H	-3.72173100	3.02080800	1.31135000
H	0.24070300	-2.65094800	-0.19547600
H	2.41799400	-2.79679200	-1.21692900
H	4.79099800	-2.15192000	-1.17689000
H	3.82537500	1.22360500	1.28167700
H	1.46205400	0.57718400	1.25306600

3ad

E= -3985.0965893 a.u., number of negative frequencies = 0

0 1

C	-1.18295900	1.62700900	-0.53894100
C	-0.35434200	0.41677600	-0.96976700
C	1.12536400	0.57057200	-0.62098400
C	-0.95326300	-0.91407200	-0.51495000
C	-2.05206900	-1.43145200	-1.20050000
C	-2.66770500	-2.62294500	-0.84506900
C	-2.13671900	-3.32376600	0.26804400
C	-1.02852800	-2.84473300	0.98919900
C	-0.46687300	-1.63373100	0.55823700
C	-2.01663100	1.61518800	0.57699200
C	-2.73895500	2.74428200	0.95346000
C	-2.63099300	3.90051200	0.19797700
C	-1.81415900	3.94310600	-0.92412800
C	-1.09873200	2.80821600	-1.27975400
C	2.07505700	0.04750000	-1.49312300
C	3.42038000	0.15105600	-1.15835000
C	3.85743800	0.77361100	0.03927000
C	2.89045300	1.29843500	0.90228000
C	1.54955200	1.19525300	0.56702800
N	4.54523000	-0.26912400	-1.83422500
C	5.66922600	0.06880100	-1.10221500
C	5.29245600	0.70342500	0.04906500
Br	-3.62598100	5.45618400	0.69984800
C	7.06928900	-0.23387700	-1.51892000
C	8.04913500	0.58414600	-0.66128800
C	7.66071500	0.56750800	0.82198600
C	6.27894300	1.19602800	1.06377800
C	-3.87932300	-3.15491100	-1.64233500

C	-0.32615700	-3.50626100	2.21050500
O	-2.77557600	-4.49728700	0.57805200
C	-4.25987300	-2.21393300	-2.79951000
C	-3.55476000	-4.52827400	-2.27157600
C	-5.12208500	-3.26597000	-0.73081800
C	-0.92832700	-4.82410300	2.74130400
C	1.13829400	-3.82023800	1.83084600
C	-0.34800300	-2.51856400	3.39886400
H	-0.40251500	0.40456500	-2.06312500
H	-2.43219400	-0.86941400	-2.04113600
H	0.39556800	-1.24617100	1.08049800
H	-2.11659500	0.71066600	1.16219500
H	-3.38140900	2.71692400	1.82265600
H	-1.73881200	4.84701000	-1.51245500
H	-0.45608300	2.84583500	-2.15184600
H	1.76514300	-0.43744000	-2.41242700
H	3.18112600	1.79142900	1.82215500
H	0.80793800	1.61849900	1.23165800
H	4.55075700	-0.74030900	-2.72095100
H	7.21773300	-0.00851700	-2.58024900
H	7.27534600	-1.30542700	-1.40172600
H	9.06201100	0.19678400	-0.79168300
H	8.05798400	1.61885700	-1.01823300
H	7.65091200	-0.46885000	1.17595300
H	8.41690900	1.09280400	1.41004200
H	5.93895900	0.96275800	2.07733200
H	6.35777600	2.28947300	1.01849300
H	-2.36747000	-4.90470900	1.34450200
H	-5.11871400	-2.63233200	-3.32730300
H	-4.54398700	-1.22047700	-2.44766500

H	-3.45163500	-2.10476200	-3.52531300
H	-4.40982700	-4.87823300	-2.85555800
H	-3.32605800	-5.27828600	-1.51943900
H	-2.70024900	-4.44675500	-2.94748200
H	-5.37968700	-2.28925600	-0.31482500
H	-5.97751100	-3.61599100	-1.31419100
H	-4.96466700	-3.95828700	0.09163400
H	-0.34376300	-5.15464300	3.60146900
H	-1.95400500	-4.70618400	3.10366300
H	-0.87342400	-5.63964000	2.01386500
H	1.66345200	-4.26036100	2.68252900
H	1.68273900	-2.92693700	1.52876000
H	1.17966800	-4.53086100	1.00267900
H	0.14755800	-1.57869900	3.16148300
H	-1.37418700	-2.29002600	3.69437500
H	0.16348300	-2.95488400	4.26051100

4ad

E= -3985.0868229 a.u., number of negative frequencies = 0

0 1

C	-3.36292800	-0.67393700	-0.71611100
C	-3.19198000	-1.99702200	-0.22919600
C	-1.92515500	-2.51523300	0.09227600
C	-0.82789200	-1.65842100	-0.08197300
C	-0.95041800	-0.36530400	-0.54565100
C	-2.22494900	0.10292900	-0.86188500
C	0.24616900	0.56268000	-0.77105600
C	1.60922900	-0.09400400	-0.57243300
N	0.11158600	1.83671300	-0.06151700
C	0.48530700	3.06434500	-0.61014000

C	0.35446500	4.05649200	0.32000800
C	-0.12496500	3.44426000	1.52186000
C	-0.26058900	2.05470100	1.25981600
C	-0.44112200	3.94232000	2.79112100
C	-0.87487100	3.06734600	3.77161400
C	-0.99833000	1.69703300	3.50143600
C	-0.69547000	1.17448700	2.25282900
C	2.36964700	0.07777200	0.58157700
C	3.60994600	-0.53776800	0.72194700
C	4.09557500	-1.33217600	-0.30518900
C	3.36346000	-1.51400800	-1.47070300
C	2.12852900	-0.89103900	-1.59387600
C	0.96219500	3.26328200	-2.01332600
C	0.96862800	4.76181700	-2.36060900
C	1.53428400	5.61740900	-1.22356000
C	0.68448000	5.49259500	0.04845400
O	-4.34541200	-2.72296600	-0.10172500
C	-4.75549300	-0.10742600	-1.07091800
C	-1.58191600	-3.93699400	0.62437000
C	-2.75664000	-4.91913700	0.81534000
C	-0.60998700	-4.61904600	-0.36472000
C	-0.90144100	-3.80400200	2.00511700
C	-5.40788700	-0.93625600	-2.19994300
C	-5.66691700	-0.09165200	0.17681500
C	-4.66812500	1.34386900	-1.57632600
Br	5.79974600	-2.17892300	-0.12179700
H	0.15755600	-2.02673300	0.16061500
H	-2.31697400	1.11774800	-1.21855700
H	0.20639600	0.83305800	-1.82810100
H	-0.34534600	5.00047300	3.00279900

H	-1.12163800	3.44057200	4.75758900
H	-1.33912900	1.02886300	4.28238100
H	-0.80232700	0.11706800	2.06453100
H	2.00272200	0.70307100	1.38381200
H	4.18991200	-0.39376900	1.62283900
H	3.75318200	-2.12483700	-2.27301300
H	1.55972800	-1.03147300	-2.50610600
H	0.32673700	2.73180900	-2.72877400
H	1.96944100	2.84627000	-2.13559200
H	1.54230300	4.91787500	-3.27695800
H	-0.05638900	5.08301400	-2.57195000
H	2.55837300	5.29567800	-1.00692500
H	1.59166700	6.66281100	-1.53588300
H	1.21879500	5.92848800	0.89800100
H	-0.23427000	6.08237200	-0.06251500
H	-4.15366300	-3.59550400	0.24825700
H	-2.36247800	-5.86888400	1.18032400
H	-3.47103500	-4.58434500	1.57393200
H	-3.27438800	-5.14882400	-0.12040300
H	-0.33437300	-5.61096200	0.00170700
H	0.30823200	-4.04920800	-0.49822800
H	-1.07484600	-4.73699800	-1.34574500
H	0.00530400	-3.20257600	1.95975900
H	-1.57526800	-3.33757700	2.72675500
H	-0.62627500	-4.79031700	2.38675600
H	-6.37756900	-0.50456900	-2.45999300
H	-4.78447000	-0.91928600	-3.09704700
H	-5.56347500	-1.97184500	-1.91041600
H	-6.63588900	0.34286500	-0.08127000
H	-5.22527600	0.52160600	0.96526600

H	-5.83626700	-1.08946800	0.57202300
H	-4.25240000	2.01832300	-0.82569800
H	-4.07089000	1.42842200	-2.48648800
H	-5.67366500	1.69633600	-1.81215700

H

E= -6540.5878106 a.u., number of negative frequencies = 0

0 1

C	-2.96248000	-0.18199500	-1.50109400
C	-1.69476200	-0.64147000	-1.65828700
C	-0.61098000	0.35745400	-1.65848700
C	-0.93461100	1.74801800	-2.00041900
C	-2.23408700	2.11872900	-1.86082100
C	-3.28257200	1.21503800	-1.48279600
O	0.58027700	-0.00501800	-1.41044200
C	-1.41654200	-2.14821700	-1.80803100
C	-4.52423600	1.73479400	-1.21528000
C	-5.71064800	1.09875100	-0.68876900
C	-6.96028400	1.67388100	-0.98528200
C	-8.13968200	1.11334000	-0.52987700
C	-8.08404800	-0.02391400	0.26973000
C	-6.86312700	-0.59064900	0.62273800
C	-5.68945600	-0.03328500	0.14619300
Br	-9.69514500	-0.79315100	0.91661800
C	0.12262700	2.77578800	-2.44809900
In	2.26410900	-0.16414000	-0.08613500
O	1.06760200	0.74776500	1.33881900
O	3.46373400	1.43553700	-1.05552800
S	4.68346100	1.30970300	-0.18765700
O	4.29309100	0.31398000	0.84516100

O	5.93526100	1.15238100	-0.84652000
C	4.76668400	2.94005200	0.72014800
F	3.62467500	3.16601800	1.36165000
F	5.76747900	2.90601500	1.59234100
F	4.97877900	3.91943000	-0.15825200
S	1.12221000	0.74478800	2.86881000
O	1.11525300	2.07960400	3.38058100
O	2.04646200	-0.23074800	3.36499200
C	-0.58070000	0.07003900	3.22398400
F	-0.72441800	-1.15460500	2.70310600
F	-0.76523300	-0.00088900	4.54093800
F	-1.52597600	0.86080600	2.70007200
O	2.34124000	-2.27455100	0.60088000
S	2.97283400	-2.85667500	-0.62803400
O	3.22957100	-1.67429500	-1.49921000
O	2.33272500	-3.98768700	-1.21370700
C	4.65703300	-3.45419200	-0.07334900
F	4.49457200	-4.45002600	0.79269900
F	5.32232800	-3.89724300	-1.13718200
F	5.33783000	-2.47291400	0.49919800
H	-3.78009900	-0.88241200	-1.45303000
H	-2.51650900	3.15116900	-2.00830400
H	-4.64404000	2.79318600	-1.43017900
H	-7.00178600	2.56445600	-1.60037500
H	-9.09220900	1.55716800	-0.78216600
H	-6.83178400	-1.44984300	1.27769400
H	-4.74584900	-0.44798300	0.46996100
C	-0.53954800	3.94209900	-3.21107300
H	-1.12874900	3.59048100	-4.06068900
H	-1.17779000	4.55661000	-2.57474700

H	0.24280000	4.59694500	-3.59650600
C	0.82455900	3.37793800	-1.21460200
H	1.26532900	2.62636200	-0.56901900
H	1.62091400	4.05452500	-1.52992400
H	0.11295000	3.94532700	-0.61190400
C	1.13410200	2.14280100	-3.42671800
H	1.82893400	2.90966800	-3.77195900
H	1.72102700	1.34681100	-2.98374800
H	0.61951800	1.74182100	-4.30341200
C	-0.93870800	-2.73469600	-0.46528600
H	-0.69430200	-3.79136300	-0.58479100
H	-0.05492600	-2.23958600	-0.07851100
H	-1.72156400	-2.64724200	0.29036200
C	-0.38847300	-2.40302400	-2.93113000
H	0.57458500	-1.94108000	-2.74054400
H	-0.22353900	-3.47579100	-3.03547300
H	-0.76434900	-2.02673500	-3.88585600
C	-2.69602700	-2.90924900	-2.20755800
H	-3.45309300	-2.90378500	-1.42161000
H	-3.14045100	-2.51081000	-3.12202400
H	-2.43863200	-3.95307100	-2.39054800

D

E= -7060.6461667 a.u., number of negative frequencies = 0

0 1

C	2.19970900	-1.64610600	-0.09472400
C	0.86257000	-2.01245800	0.08432900
C	0.15669200	-1.39391800	1.14889900
C	0.86810100	-0.69257800	2.16754100
C	2.20547500	-0.39591600	1.92801200

C	2.87083900	-0.80397700	0.77532200
O	-1.18317400	-1.52085100	1.26274600
C	0.29626400	-3.07378300	-0.89687100
C	5.30094700	-1.33263100	0.18532200
C	6.08988400	-1.82919000	1.22507400
C	7.02296300	-2.83586600	1.01568100
C	7.17997500	-3.35834300	-0.26040400
C	6.41588400	-2.88459000	-1.31634800
C	5.48544200	-1.87711300	-1.08621300
Br	8.46515700	-4.73510200	-0.56715800
C	0.22692300	-0.30552800	3.52099700
In	-2.69013700	-0.50939500	0.38379300
O	-4.10726400	0.24473100	1.87846400
O	-3.07466900	-1.04362300	-1.58600100
S	-4.17071800	-0.46635000	-2.48288100
O	-4.99288700	0.45257400	-1.74283700
O	-3.65716300	-0.11373700	-3.76926700
C	-5.20933300	-1.99070600	-2.76897500
F	-4.48896800	-2.94070100	-3.36889500
F	-5.67665700	-2.47096600	-1.61574800
F	-6.24059000	-1.67204800	-3.55312300
S	-4.77828000	-1.05364700	2.24250400
O	-4.74678100	-1.41617200	3.62117300
O	-4.27919400	-2.02289200	1.24265700
C	-6.57913200	-0.76937300	1.83390700
F	-7.06353100	0.17735500	2.63639800
F	-6.72373000	-0.39370000	0.57180000
F	-7.24916700	-1.90235000	2.03983800
O	-2.12536900	1.55122600	0.06126800
S	-1.02941300	2.30846600	-0.60315300

O	-0.12660200	1.48672200	-1.36653500
O	-0.42379500	3.27841200	0.29463700
C	-1.95627100	3.35914100	-1.83350700
F	-2.54982700	2.60616400	-2.74575700
F	-2.86339300	4.11131100	-1.21429000
F	-1.07301800	4.16827700	-2.44134800
C	-0.81750200	0.81720500	3.37015800
C	-0.42158100	-1.55045100	4.16586000
C	1.28092500	0.20938600	4.52084300
C	1.32154900	-4.22864000	-1.02147200
C	-1.01633700	-3.74687900	-0.44783600
C	0.09161000	-2.44567500	-2.29052000
C	4.26577100	-0.26663600	0.50258800
C	5.54236500	1.61705800	-0.65002800
C	3.04712100	1.75884700	-0.43228700
C	5.66598500	2.95066500	-0.49034800
C	3.20780500	3.08275900	-0.26701100
C	4.49195600	3.74387800	-0.27984400
N	2.24308500	4.06490000	-0.06082000
C	4.25971400	5.09482500	-0.07941900
C	2.85366500	5.24494600	0.05787400
C	5.19277000	6.26720900	-0.01692400
C	2.16595900	6.53659000	0.32400300
C	3.10537200	7.71850900	0.03720300
C	4.50346100	7.48512100	0.61950300
C	4.23882800	0.89392400	-0.60217900
H	2.72994600	-2.04236100	-0.94858500
H	2.75724000	0.18318900	2.65451400
H	5.96899600	-1.42855100	2.22455700
H	7.62138800	-3.20622900	1.83621200

H	6.54156700	-3.29403700	-2.30887500
H	4.90277200	-1.52654000	-1.92891200
H	-1.14842300	1.14853200	4.35726300
H	-0.40280200	1.68060100	2.84703200
H	-1.70745000	0.49187700	2.84125600
H	-0.87412200	-1.27873800	5.12219800
H	0.33151000	-2.31845500	4.35685400
H	-1.19467700	-1.97968300	3.53477800
H	2.08318400	-0.51340900	4.68107200
H	1.72488600	1.15729800	4.20845600
H	0.79641500	0.38282900	5.48293100
H	0.92321900	-4.99337200	-1.69079700
H	2.28028400	-3.91239800	-1.43022200
H	1.50715400	-4.69307900	-0.05088800
H	-1.18247300	-4.62918800	-1.06971300
H	-1.88854400	-3.11380000	-0.57219900
H	-0.97524600	-4.06974400	0.59170900
H	1.03077100	-2.06113700	-2.69372100
H	-0.29121500	-3.19422100	-2.98853700
H	-0.62195700	-1.62301100	-2.26558200
H	4.59124700	0.22862700	1.41924800
H	6.42504300	1.00902800	-0.80134200
H	2.06509400	1.30652200	-0.45189500
H	6.63930800	3.42339600	-0.51945400
H	1.23650200	3.87210300	0.03961600
H	6.09603500	6.00818600	0.54032600
H	5.52275800	6.52519400	-1.02967300
H	1.86008900	6.54657200	1.37711500
H	1.24318800	6.60050900	-0.25716800
H	3.18026200	7.86831600	-1.04406200

H	2.66943100	8.62935600	0.44930800
H	5.12226200	8.37155300	0.47209400
H	4.42398100	7.33483600	1.70100900
H	4.09850600	0.36912000	-1.55856800

B

E= -7060.6355151 a.u., number of negative frequencies = 0

0 1

C	-1.42260100	0.02844000	2.19032600
C	-0.16510800	-0.49518800	2.46322400
C	0.33224400	-1.46581500	1.55294900
C	-0.57305600	-2.16681400	0.70367700
C	-1.83482400	-1.61398800	0.51342300
C	-2.23789500	-0.45267700	1.16761800
C	-3.46743100	0.34461000	0.85203900
C	-4.81748600	-0.31985500	0.73758600
O	1.64865400	-1.76703800	1.53257500
C	-5.81005500	0.09169600	1.63149200
C	-7.06963700	-0.49242500	1.64817900
C	-7.35183800	-1.50701600	0.74627000
C	-6.38972800	-1.93797400	-0.15708300
C	-5.13327800	-1.34798900	-0.15539200
Br	-9.07586200	-2.31865600	0.74694500
N	-3.17382900	1.33597800	-0.41186900
C	-3.50006100	0.85352100	-1.76280500
C	-4.49316100	1.67148300	-2.30293300
C	-4.75283100	2.74698800	-1.35110800
C	-3.95653700	2.58393700	-0.28728200
C	-2.89338500	-0.15809500	-2.47265100
C	-3.37230200	-0.39855800	-3.76236900

C	-4.40229300	0.36707300	-4.30190700
C	-4.96509400	1.42019900	-3.58453200
C	-5.66833700	3.91792900	-1.50940800
C	-5.76803900	4.72090100	-0.20368000
C	-4.40211200	4.88553800	0.47076900
C	-3.78768400	3.52715100	0.85404500
C	0.59840900	-0.06179000	3.73467800
C	-0.21519300	-3.52361400	0.05813400
C	-0.30047600	0.77025600	4.66901900
C	1.02111300	-1.32416000	4.51791200
C	1.83949700	0.79209100	3.42625300
C	0.20903200	-4.50458800	1.17375000
C	-1.42391500	-4.14774300	-0.66224000
C	0.91938800	-3.40539400	-0.97511100
O	3.67238300	-1.20395400	-1.65499500
S	4.46629100	-2.44891200	-1.36129300
O	4.17010700	-3.59274900	-2.15920600
O	4.41616600	-2.58173600	0.11422200
C	6.23233500	-1.99474500	-1.77324800
F	7.01024600	-3.04571900	-1.51937100
F	6.31487800	-1.68948300	-3.06561400
F	6.63483600	-0.96324200	-1.04571000
O	1.75705200	0.93277800	-0.22033600
S	0.67453800	1.22499500	-1.20531000
O	0.34678400	0.11425500	-2.05635600
O	-0.43168800	1.93193900	-0.57891100
C	1.47139300	2.50989900	-2.29654400
F	2.52932600	2.00001000	-2.91222000
F	0.57779600	2.90140900	-3.21411100
F	1.83939400	3.56836600	-1.57900600

O	4.43695400	0.34653200	1.37497600
S	5.36173800	1.43928800	0.83784300
O	6.71538800	1.20611800	1.23164600
O	5.03764400	1.77171500	-0.52092500
C	4.80219200	2.89420500	1.86344800
F	4.93152800	2.63660100	3.16759600
F	5.55125300	3.95498600	1.55938500
F	3.52197600	3.19093100	1.61360800
H	-1.78668700	0.85143600	2.78893600
H	-2.51123300	-2.09883900	-0.16878200
H	-3.55195900	1.08271900	1.64302600
H	-5.59563400	0.87703700	2.34622400
H	-7.81766200	-0.16102500	2.35443000
H	-6.61440900	-2.73091500	-0.85633500
H	-4.40957500	-1.70039100	-0.87252900
H	-2.14793700	1.53313800	-0.36786700
H	-2.05914400	-0.71614300	-2.07655200
H	-2.91679900	-1.18080800	-4.35451100
H	-4.74990900	0.15908800	-5.30548300
H	-5.73266900	2.04136300	-4.02715900
H	-6.65826400	3.57766000	-1.82611400
H	-5.29449000	4.55101100	-2.32219200
H	-6.20641600	5.69930200	-0.40654000
H	-6.44973300	4.20878900	0.48244600
H	-4.49212400	5.50578200	1.36367700
H	-3.71958600	5.40431700	-0.20812900
H	-2.72790700	3.64429000	1.09842800
H	-4.27400600	3.14664000	1.75993800
H	0.23684300	0.95537100	5.60021900
H	-0.55100100	1.74509700	4.24475400

H	-1.22737700	0.25048400	4.91957200
H	1.56025800	-1.03435500	5.42275300
H	1.67211300	-1.96318700	3.92515900
H	0.14714800	-1.90589800	4.81909800
H	1.60945100	1.60294500	2.73451600
H	2.22205500	1.23773500	4.34719400
H	2.65181000	0.19919900	3.01640900
H	0.47157900	-5.47132800	0.73791400
H	1.07469300	-4.13452900	1.71960700
H	-0.60648400	-4.66438100	1.88254400
H	-1.14411000	-5.13818900	-1.02358600
H	-1.73410200	-3.56484100	-1.53287800
H	-2.28331400	-4.27075800	-0.00003800
H	1.87677900	-3.27554300	-0.48112600
H	0.99737700	-4.32625500	-1.55617700
H	0.75398000	-2.58259200	-1.67229300
In	2.97867800	-0.73654700	0.37163400

C (TS2)

E= -7060.6374673 a.u., im. freq.= 346.55*i*

0 1

C	2.25945200	-1.83355900	0.17270800
C	0.90700400	-2.07931700	0.34426700
C	0.16083000	-1.15684000	1.14828200
C	0.85249100	-0.22032700	1.98603300
C	2.19421100	-0.01420600	1.72453500
C	2.91856100	-0.76313800	0.78656100
O	-1.16759500	-1.23383900	1.19537300
C	0.27964700	-3.32779900	-0.31889900
C	5.36564200	-1.38826000	0.25719800

C	6.42340900	-1.48361800	1.16837000
C	7.43399300	-2.42289500	1.01478100
C	7.40527600	-3.27457700	-0.08013700
C	6.38208300	-3.19026600	-1.01582500
C	5.37423500	-2.25236600	-0.84282700
Br	8.79091300	-4.56143000	-0.31263500
C	0.18898400	0.50085100	3.18348300
In	-2.77487400	-0.37416400	0.29846600
O	-4.03769100	0.70784600	1.71958500
O	-3.46973300	-1.34234300	-1.39211700
S	-4.62543900	-0.88022300	-2.28495000
O	-5.31929600	0.22110400	-1.67481700
O	-4.23414000	-0.82724800	-3.65796200
C	-5.74992900	-2.36416400	-2.14819700
F	-5.14087300	-3.45440400	-2.61773800
F	-6.09938500	-2.58119500	-0.87924800
F	-6.85094700	-2.14100100	-2.86696200
S	-4.61326000	-0.46439100	2.47240900
O	-4.40820600	-0.46819200	3.88356800
O	-4.19001600	-1.64778200	1.69205800
C	-6.45980000	-0.30623700	2.22944100
F	-6.88358600	0.79710200	2.84274100
F	-6.76386100	-0.24093600	0.94259300
F	-7.05330300	-1.36676900	2.77460800
O	-2.18444700	1.49091000	-0.55375700
S	-0.99504100	2.01379800	-1.29762500
O	-0.08230300	0.98082300	-1.71335000
O	-0.43199000	3.17905400	-0.64808800
C	-1.78266400	2.68034100	-2.85049300
F	-2.37216900	1.70489600	-3.52800000

F	-2.67761000	3.61926800	-2.55223400
F	-0.82332300	3.22453500	-3.60988900
C	-0.76858900	1.61995100	2.73708900
C	-0.55359100	-0.52683600	4.06539200
C	1.24373000	1.16818300	4.08954200
C	1.36329700	-4.32970900	-0.76860200
C	-0.62167900	-4.09270100	0.67480400
C	-0.51097800	-2.93470200	-1.57985300
C	4.30192700	-0.38516100	0.53810100
C	5.67368700	1.51005800	-0.88909500
C	3.20762300	1.64641100	-0.99466000
C	5.77341000	2.82357200	-0.53707700
C	3.32144800	2.95316500	-0.63266500
C	4.58768400	3.58927900	-0.38451900
N	2.33782200	3.90790300	-0.44582600
C	4.31633700	4.92231600	-0.04377100
C	2.92484900	5.07353600	-0.09777700
C	5.23320900	6.05699900	0.30295100
C	2.19087400	6.33431100	0.20070100
C	3.15986500	7.52724200	0.20244700
C	4.45344600	7.20750200	0.95975700
C	4.39637800	0.84997700	-1.04686800
H	2.82752800	-2.49730600	-0.45535400
H	2.72961000	0.74296500	2.27813400
H	6.45189800	-0.82011100	2.02404900
H	8.23525200	-2.48631200	1.73750600
H	6.37246200	-3.84711200	-1.87418400
H	4.59884800	-2.19262700	-1.59456900
H	-1.13257800	2.16465600	3.61107600
H	-0.26974000	2.33135800	2.07767700

H	-1.64898600	1.24828000	2.22417100
H	-1.02135800	-0.01840400	4.91060300
H	0.14687900	-1.26497600	4.46273300
H	-1.32998400	-1.05713900	3.52264500
H	1.99926000	0.45839800	4.43158400
H	1.74826600	2.00419500	3.60057100
H	0.74268600	1.56850700	4.97202800
H	0.87366100	-5.23445600	-1.13137400
H	1.97270000	-3.94949500	-1.59067500
H	2.02470800	-4.61578000	0.05168100
H	-1.02107100	-4.98538900	0.18909600
H	-1.45657200	-3.49736500	1.02982000
H	-0.04533300	-4.41688200	1.54460200
H	0.12755000	-2.40879400	-2.29183100
H	-0.90151900	-3.82938700	-2.07040800
H	-1.36213600	-2.29723500	-1.36777100
H	4.64218700	0.34592300	1.26310200
H	6.57021700	0.91823100	-1.01977900
H	2.24178900	1.19967000	-1.19485800
H	6.74274200	3.28334200	-0.39307400
H	1.33590500	3.73353000	-0.53929600
H	6.03154800	5.71637200	0.96757900
H	5.72944200	6.42320400	-0.60351300
H	1.71128200	6.23770600	1.18207500
H	1.37981200	6.48145000	-0.51687600
H	3.40136000	7.79788100	-0.83003000
H	2.66511700	8.39292100	0.64551800
H	5.08520700	8.09608500	1.01165000
H	4.20806000	6.93633100	1.99180000
H	4.36600200	0.02138900	-1.74316700
